# Design and Synthesis of Sulfonium and Selenonium Derivatives Bearing 3′,5′-*O*-Benzylidene Acetal Side Chain Structure as Potent α-Glucosidase Inhibitors

**DOI:** 10.3390/molecules30132856

**Published:** 2025-07-04

**Authors:** Xiaosong He, Jiahao Yi, Jianchen Yang, Genzoh Tanabe, Osamu Muraoka, Weijia Xie

**Affiliations:** 1 Department of Medicinal Chemistry, China Pharmaceutical University, 24 Tong Jia Xiang, Nanjing 210009, China; 18182409307@163.com (X.H.); 15173092366@163.com (J.Y.); 2 Department of Biomedical Engineering, The University of Texas at Austin, Austin, TX 78712, USA; 3 School of Pharmacy, Kindai University, 3-4-1 Kowakae, Higashiosaka 577-8502, Osaka, Japan; g-tanabe@phar.kindai.ac.jp (G.T.); osamumuraoka3@gmail.com (O.M.)

**Keywords:** α-glucosidase inhibitor, *Salacia*, sulfonium salts, selenonium salts, anti-hyperglycemic effects

## Abstract

A group of sulfonium and selenonium salts bearing diverse benzylidene acetal substituents on their side chain moiety were designed and synthesized. Compared with our previous study, structural modifications in this study focused on multi-substitution of the phenyl ring and bioisosteric replacements at the sulfonium cation center. In vitro biological evaluation showed that selenonium replacement could significantly improve their α-glucosidase inhibitory activity. The most potent inhibitor **20c** (10.0 mg/kg) reduced postprandial blood glucose by 48.6% (15 min), 52.8% (30 min), and 48.1% (60 min) in sucrose-loaded mice, outperforming acarbose (20.0 mg/kg). Docking studies of **20c** with ntMGAM presented a new binding mode. In addition to conventional hydrogen bonding and electrostatic interaction, amino residue Ala-576 was first identified to contribute to binding affinity through π-alkyl and alkyl interactions with the chlorinated substituent and aromatic ring. The selected compounds exhibited a high degree of safety in cytotoxicity tests against normal cells. Kinetic characterization of α-glucosidase inhibition confirmed a fully competitive inhibitory mode of action for these sulfonium salts.

## 1. Introduction

Diabetes mellitus typically arises due to either insufficient insulin secretion resulting from the destruction of β cells or the body’s ineffective utilization of normally produced insulin. It is projected that by 2045, the number of individuals suffering from diabetes will reach 783 million [1]. Elevated blood glucose levels are closely associated with a range of complications, including vascular damage, kidney failure, nerve damage, and severe foot infections, among others [2]. The most frequently prescribed medications for T2DM, accounting for over 90% of all diabetes cases, are oral antidiabetic agents, encompassing insulin secretagogues (such as sulfonylureas), insulin sensitizers (like metformin), PPARγ agonists (thiazolidinediones), DPP-4 inhibitors (e.g., sitagliptin) and GLP-1 mimetics (e.g., exenatide) [3,4], sodium-glucose cotransporter 2 (SGLT2) inhibitors [5,6], and α-glucosidase inhibitors (e.g., acarbose) [7,8]. Among the various therapeutic strategies available, one particularly effective method involves delaying intestinal glucose absorption by interfering with the breakdown of polysaccharides [9]. This can be accomplished by inhibiting different α-glucosidases located at the brush border of the intestine, a strategy that has garnered significant attention in medicinal chemistry due to its lack of severe side effects. This approach is especially beneficial for Asian patients, whose diets predominantly consist of carbohydrates [10,11], a dietary pattern that contributes to the high prevalence of diabetes in densely populated Asian countries, thereby driving the global diabetes epidemic. The three currently approved α-glucosidase inhibitors for hyperglycemia—acarbose, voglibose, and miglitol—all belong to the azasugar or iminosugar derivative classes [12,13]. While these agents offer moderate therapeutic benefits for T2DM, they are sometimes associated with gastrointestinal disturbances, such as bloating, diarrhea, nausea, liver dysfunction, and skin allergies, among other adverse effects [14]. Consequently, the identification of potent α-glucosidase inhibitors with high safety profiles remains a focal area of research for medicinal chemists [15,16,17,18,19,20,21,22,23,24].

**Figure 1:**
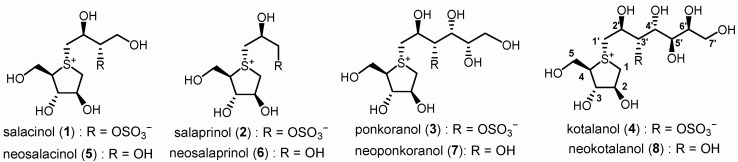
Sulfonium type α-glucosidase inhibitors isolated from Genus *Salacia*.

Since 1997, a group of sulfonium salts characterized by polyhydroxylated side chain structure have been isolated from plants belonging to the genus *Salacia* [25,26,27]. These compounds, featuring distinct five-membered-ring sulfonium derivatives, can be categorized into two groups. Compounds **1**–**4** [28] present a unique zwitterionic sulfonium-sulfate salt structure, while compounds **5**–**8** [29,30,31,32] are identified as the de-*O*-sulfonated derivatives of **1**–**4**, respectively (Figure 1). In vitro biological assessments have demonstrated that these sulfonium salts exhibit potent α-glucosidase inhibitory activity comparable to clinically prescribed anti-diabetic drugs such as acarbose and voglibose. Their unique structural character, coupled with promising biological activities, has established them as a novel class of drug candidates, specifically as potent α-glucosidase inhibitors. Consequently, significant efforts have been directed towards the total synthesis [29,33,34,35,36,37,38,39,40,41,42,43] and structural modification [44,45,46,47,48,49,50,51,52] of this series of compounds.

**Figure 2:**
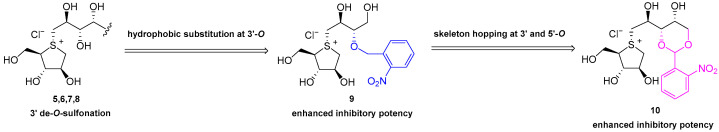
Our previous structural modification at the 3′-*O* and 5′-*O* positions of *Salacia*-derived Sulfonium type α-glucosidase inhibitors.

Natural products. Previous structure–activity relationship studies have unequivocally demonstrated that the five-membered sulfonium ring fragment represents the most structurally conserved region responsible for potent bioactivity, with the cooperative effect of the 2′S-OH and 4′R-OH groups on the side chain being crucial for achieving strong inhibitory effects [29] (Figure 1 and Figure 2). Polyhydroxylated side chains longer than four carbons, such as those found in compounds **7** and **8**, do not significantly enhance the enzyme inhibitory potency [29]. Additionally, the hydrophilic 3′-*O*-sulfate moiety in compounds **1**–**4** is constrained by adjacent hydrophobic residues of the target enzymes [53,54], explaining why de-*O*-sulfonated analogs **5**–**8** (Figure 1) typically exhibit superior inhibitory potency compared to their sulfated counterparts **1**–**4**. As part of a rational drug design approach, a series of hydrophobic substituents were deliberately introduced at the 3′-*O* position of neosalacinol by us [48]. It was discovered that sulfonium salt **9** (Figure 2), which features a substituted benzyl group at the 3′-*O* position, exhibited significantly increased α-glucosidase inhibitory activities. In silico docking studies revealed an effective π-π stacking interaction between the phenyl ring of the amino acid residue Phe450 and the benzyl group. Recently, we reported a group of sulfonium derivatives (**10**) with a five-carbon side chain structure on which 3′-OH and 5′-OH diol were protected by a benzylidene acetal ring [54]. Mono-substitution on the benzene ring resulted in different sulfonium salts with different enzyme inhibitory profiles. Specifically, the introduction of a strong electron withdrawing group at the ortho position was most beneficial to present potent α-glucosidase inhibitory activities. Two sulfonium salts with a nitro- or trifluromethyl group attached at the ortho position on the benzene ring exhibited potent in vitro and in vivo bioactivities. Based on previous investigation, we believe that there remains a potential for further structural modification of the benzylidene acetal moiety. For example, an increased dihedral angle between the phenyl ring and acetal ring, as well as a stronger electron-deficient effect on the phenyl ring, may be more beneficial for further enhancing α-glucosidase inhibitory potency. As a continuous SAR study, this paper describes a more comprehensive structural modification focusing on the benzene ring within the benzylidene group. Multiple substituents were introduced onto the benzene ring. Furthermore, utilizing the principle of “bioisosterism”, we replaced the sulfonium cation core moiety with a selenonium cation structure, and a series of target compounds were designed and synthesized. In vitro enzymatic inhibitory activity against maltase and sucrase was evaluated. The most potent inhibitor was selected for in vivo antihyperglycemic efficacy investigation.

## 2. Results and Discussion

### 2.1. Chemistry

**Scheme 1:**
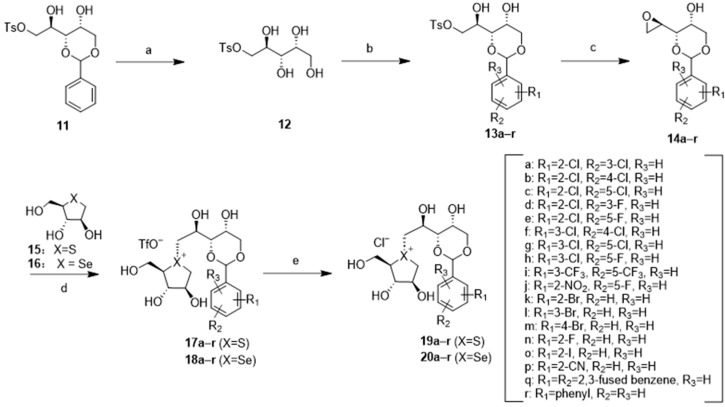
Reagents and conditions: (a) 30% aq TFA, DCM, r.t, 4 h, 73%; (b) R-benzaldehyde, p-TSA, MeCN, r.t, 24 h, 41–60%; (c) DBU, DCM, 0 °C, 2 h, 85–96%; (d) TFA, MeCN, −20 °C-r.t, 8 h; (e) IRA400J (Cl^−^ form), MeOH, r.t, 4 h, 45–62%.

Starting from compound **11** [49], selective removal of the benzylidene protecting group [55] using 30% trifluoroacetic acid (TFA) solution yielded **12** in 73% yield, serving as a pivotal intermediate for diverse downstream chemical reactions (Scheme 1). Compound **12** was subjected to acid-catalyzed acetalization with different substituted benzaldehydes to give **13a**–**r** in 41–60% yield. **13a**–**r** underwent direct vicinal cyclization [56] to produce epoxide **14a**–**r** with a yield of 85–96%. Subsequently, a coupling reaction with the five-membered thiosugar **15** [35] or selenosugar **16** [45] was conducted, following a previously reported procedure [29] but in a modified manner. In this step, **14a**–**r** was coupled with thiosugar **15** and selenosugar **16** in the presence of a catalytic amount of trifluoroacetic acid (TFA), resulting in the formation of sulfonium salts **17a**–**r** and **18a**–**r**. Under these reaction conditions, the acid-sensitive benzylidene moiety remained. Target compounds were obtained in satisfactory yield without any observation of any degradation products. Following a direct ion exchange reaction with IRA 400J (in its chloride form), sulfonium chloride **19a**–**r** and selenonium chloride **20a**–**r** were obtained, respectively.

### 2.2. α-Glucosidase Inhibitory Activity Evaluation

The α-glucosidase inhibitory activity of compounds **19a**–**r** and **20a**–**r** was evaluated in vitro against rat intestinal sucrase and maltase, in comparison with the natural sulfonium salt neoponkoranol (**7**) and the clinical anti-diabetic drugs voglibose and acarbose. IC_50_ values are shown in Table 1 and Table 2. Our previous investigation revealed that an electron withdrawing group attached at the ortho-position on the phenyl ring is the most beneficial way to increase enzyme inhibitory activity of the resulting sulfonium salts. Thus, most of the designed products maintained an electron withdrawing substituent at ortho-position on phenyl ring. Compounds **19a**–**19e** are sulfonium salts featuring a second halogen substitution at either the meta- or para-position on the phenyl ring structure.

Generally, the introduction of a second halogen enhanced the maltase inhibitory activity of sulfonium salts compared to acarbose. Specifically, compounds **19a**, **19c**–**e** exhibited IC_50_ values ranging from 0.50 to 1.0 μM, demonstrating approximately seven-fold greater potency than that of acarbose (IC_50_ value of 3.5 against maltase). For compounds **19f**–**h**, chlorine substituent was moved from the ortho- to the meta-position. The introduction of a second chlorine did not further increase the inhibitory efficacy of the resulting sulfonium salts, which clearly demonstrated the critical role of ortho-substitution by an electron withdrawing group for strong bioactivity. When two strong electron-withdrawing group (EWG) trifluoromethyl groups were introduced at two meta-positions, the resulting sulfonium salt **19i** also showed modest inhibitory potency (IC_50_ = 8.9 μM) against maltase. For sulfonium salt **19j**, a strong EWG nitro group was placed at the ortho-position again and the second halogen (fluorine) atom introduced at the para-position yielded compound **19j** with increased maltase inhibition (IC_50_ = 0.75 μM). In this study, we also synthesized several sulfonium salts with mono-substitution on the benzene ring as a supplementary investigation to our previous study. When bromine was introduced, sulfonium salt **19k** with ortho-Br attachment showed stronger maltase inhibitory activity than that of **19l** and **19m**. It is interesting that **19m** with bromine attached at the para-position is more potent than **19l**, a meta-bromo-substituted sulfonium analog, which is inconsistent with findings from our previous study [57]. The known sulfonium salt **19n** also showed potent α-glucosidase inhibitory activity in the present study. Iodine is a halogen atom with relatively weak electron-withdrawing capacity. Unsurprisingly, compound **19o** with ortho-iodine substitution exhibited slightly decreased inhibitory activity. When ortho-iodine was replaced by an ortho-cyano group, the resulting sulfonium salt **19p** exhibited increased maltase inhibitory activity comparable to that of **19n**.

In this study, we also attempted to replace the phenyl ring with other aromatic functional groups, such as naphthyl and biphenyl groups, resulting in compounds **19q** and **19r**. Unfortunately, both compounds showed decreased inhibitory efficacy when compared with the benzaldehyde analog. In a sucrase enzymatic assay, a similar trend to that discussed above was observed. **19n** presented the strongest inhibitory activity against both maltase and sucrase.

By applying the principle of “bioisosterism”, a group of selenonium analogs (**20a**–**r**) were also synthesized. Their inhibitory activity against maltase and sucrase was evaluated. To our delight, the biological activities of selenonium salts were increased to some extent when compared with their sulfonium counterparts. Especially, selenonium salt **20c** could effectively inhibit maltase with an IC_50_ value of 0.14 μM and sucrase with an IC_50_ value of 0.05 μM, representing 25-fold and 44-fold greater potency than acarbose, respectively. This inhibitory potency is also much superior to that of voglibose, a clinically used strong α-glucosidase inhibitor. Unlike sulfonium salts **19f**–**i**, which only showed modest enzymatic inhibitory activities, the inhibitory efficacy of selenonium salts **20f**–**i** against maltase and sucrase increased from 7-fold to 25-fold. It should be noted that selenonium analog **20m** with para-bromo substitution even showed superior α-glucosidase inhibitory activities compared to its ortho-bromo and meta-bormo counterparts **20k** and **20l**. Equipped with the selenonium core structure, compounds **20q** and **20r**, featuring naphthyl and biphenyl substituents, also showed improved inhibitory activities against both maltase and sucrase. Especially, **20q** was shown to be a stronger maltase inhibitor even compared to voglibose.

### 2.3. Kinetic Enzymatic Studies

**Figure 3:**
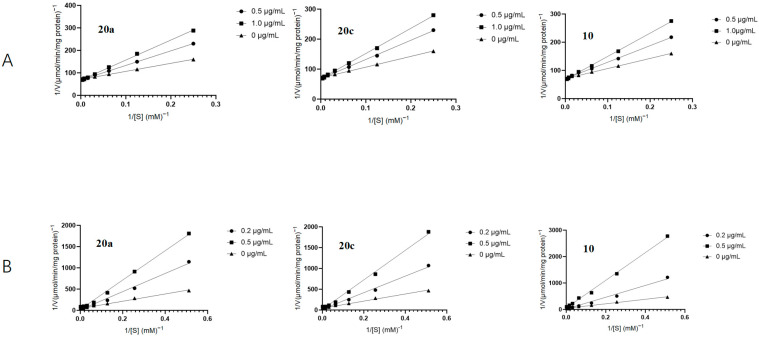
Lineweaver–Burk plots of the inhibition of rat intestinal maltase (**A**) and sucrase (**B**) activities by compounds **20a**, **20c**, and **10** (Maltase concentration: 0.25 mg/mL; Sucrase concentration: 1.31 mg/mL).

In order to explore the inhibitory mechanism of the α-glucosidase by this family of compounds, enzyme kinetic assays were conducted on **20a**, **20c**, and **10**, followed by a detailed kinetic analysis using the Lineweaver–Burk plots. (Figure 3) The small intestinal brush border membrane vesicles were incubated with varying concentrations of maltose (3.27 mg/mL) for maltase assays and sucrose (52.37 mg/mL) for sucrase assays. The plots of 1/V against 1/[S] revealed a series of linear relationships with varying slopes, indicating that this family of compounds competitively inhibits the enzymes by binding to their active sites and competing with the natural substrates. The inhibition constants (*K*i) of all selected inhibitors are listed in Table 3. Compound **10** displayed the highest binding affinity for maltase, as indicated by its *K*i value of 0.42 μg/mL. Similarly, compound **20c** exhibited the highest affinity for sucrase, with a Ki of 0.20 μg/mL.

### 2.4. Cytotoxicity

The cytotoxicity of selected sulfonium salts (**20a**, **20c**, and **10**) was then evaluated via the 3-[4,5-dimethylthiazol-2-yl]-2,5-diphenyl-tetrazolium bromide (MTT) assay against different cell lines. All compounds showed non-toxic behavior, indicating a high level of safety profile.

### 2.5. Hypoglycemic Effect in Normal Rats

**Figure 4:**
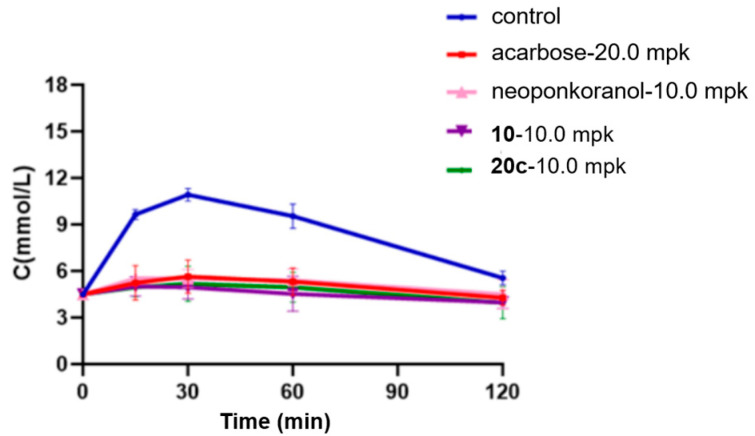
Effects of **20c** (10.0 mpk), **10** (1.0 mpk), neoponkoranol (10.0 mpk), and acarbose (20.0 mpk) on blood glucose levels in the sucrose loading test. The experiments were performed in three independent experiments. Each value represents the mean ± SD (*n* = 8).

**Figure 5:**
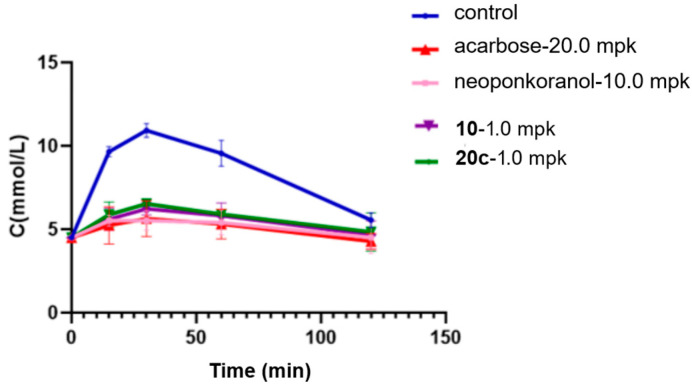
Effects of **20c** (1.0 mpk), **10** (1.0 mpk), neoponkoranol (1.0 mpk), and acarbose (20.0 mpk) on blood glucose levels in the sucrose loading test. The experiments were performed three times independently. Each value represents the mean ± SD (*n* = 8).

Research has shown a strong association between the 2 h postprandial blood glucose level and the risk of cardiovascular events, diabetic microvascular complications such as retinopathy, and the progression of macrovascular diseases [57,58]. Recent clinical data suggest that postprandial hyperglycemia may exacerbate cognitive decline in elderly individuals with type 2 diabetes mellitus (T2DM) [59]. Considering this underlying pathological process, α-glucosidase inhibitors, which efficiently regulate postprandial glucose fluctuations, hold significant therapeutic promise for alleviating diabetes-related complications. In this investigation, selenonium salt derivatives **20c**, identified through rigorous screening as the most potent inhibitor of α-glucosidase, was chosen for in vivo assessment of their modulatory effects on endogenous glucose homeostasis mechanisms. In the sucrose challenge model (2.5 g/kg p.o.), fasting ICR mice experienced a swift surge in blood glucose levels, with rapid elevation peaking at 30 min post-administration (*T*max) followed by a gradual return to baseline by 120 min (Figure 4). Pharmacological intervention with acarbose (20 mg/kg) demonstrated dose-dependent suppression across all measured time points (15, 30, 60, and 120 min). Relative to acarbose, naturally derived neoponkoranol (10.0 mg/kg) showed a similar trend of hypoglycemic effects but with relatively lower efficacy (Table 4). Candidate compound **20c** was initially dosed at 10.0 mg/kg, exhibiting a robust and early-phase suppression of postprandial glucose levels comparable to acarbose but with greater potency (Figure 4). Compound **20c** (10 mg/kg) exhibited superior early-phase glucose modulation compared to acarbose (20 mg/kg), achieving 48.6% reduction at 15 min (vs. 45.7% for acarbose) and peak efficacy of 52.8% at 30 min (vs. 48.4% control) (Table 4). The 46.4% decrease in the AUC for **20c** (10 mg/kg) was also greater than the 40.5% decrease observed for acarbose (20 mg/kg). Sulfonium salt **10**, the best inhibitor identified in our previous investigation, was also tested for hypoglycemic effect assay. Although **20c** showed a slightly higher blood glucose reduction rate than **10** at 15 min post-administration, compound **10** exhibited slightly stronger glucose-lowering efficacy at 30 min and 60 min. In terms of the AUC reduction, both compounds demonstrated almost the same effects. Among all administered compounds, naturally occurring neoponkoranol yielded the least favorable results, further validating our strategy of bridging 3′ and 5′-hydroxyls via benzylidene acetal cyclization of the side chain moiety of these naturally derived sulfonium salts. Subsequently, doses were reduced to 1.0 mg/kg. As expected, the three administered compounds, neoponkoranol, **20c**, and **10**, exhibited decreased blood glucose reduction rates at 15 min, 30 min, and 60 min, respectively. Although compounds **20c** and **10** (1 mg/kg) exhibited lower glucose-lowering efficacy compared to acarbose (20 mg/kg), both derivatives maintained statistically significant attenuation of postprandial hyperglycemia (Table 5 & Figure 5). At this low dose, **20c** still demonstrated antihyperglycemic potency comparable to compound **10** across all time points (15–60 min).

**Figure 6:**
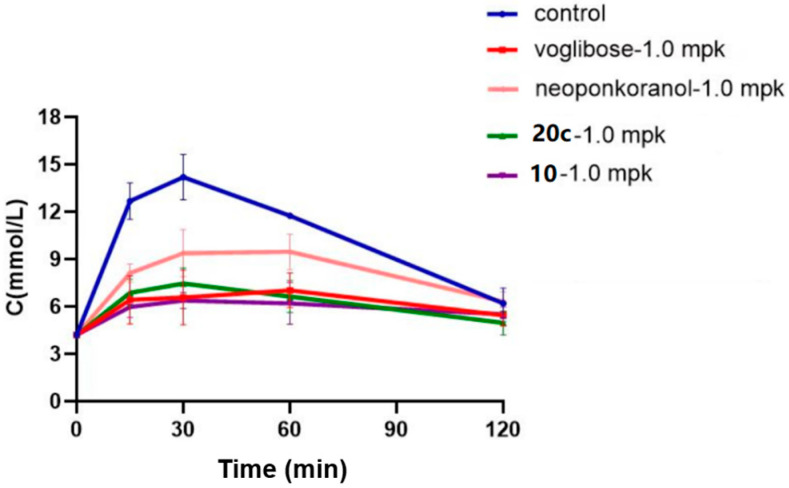
Effects of **20c** (1.0 mpk), **10** (1.0 mpk), neoponkoranol (1.0 mpk), and voglibose (1.0 mpk) on blood glucose levels in the maltose loading test. The experiments were performed in three independent experiments. Each value represents the mean ± SD (*n* = 8).

A maltose loading test on compounds **10**, **20c**, neoponkoranol, and positive control (voglibose) was conducted to further evaluate their in vivo hypoglycemic effects against maltase. As shown in Figure 6, when fasted rats were administered maltose at a dose of 2.0 g/kg, their blood glucose levels swiftly rose from 4.75 mmol/L to a peak of 14.62 mmol/L within 30 min post-ingestion. Subsequently, these levels returned to the pretreatment baseline at 120 min. The clinically used strong α-glucosidase inhibitor voglibose (1.0 mg/kg) showed an evident antihyperglycemic effect across all measured time points. At an equimolar dose, compound 10 presented the strongest capability to delay the hydrolysis of maltose. As shown in Table 6, compound **10** achieved blood glucose reduction rates of 52.8%, 55.0%, and 47.2% at 15, 30, and 60 min, respectively, which are superior to those of voglibose. Compound **20c** also showed evident hypoglycemic effects. Although less potent than **10**, compound **20c** achieved a 43.5% blood glucose reduction rate at 60 min (vs. 40.1% for voglibose). Notably, administration of **20c** led to a 40.2% decrease in AUC, which was also greater than that of voglibose (a 39.8% decrease in AUC). Naturally occurring neoponkoranol showed reduction rates of 42.3%, 33.9%, and 19.4% at 15, 30, and 60 min and a 21.5% decrease in AUC, making it the least effective among the four tested compounds.

### 2.6. Docking Studies on Compound ***20c*** with ntMGAM

**Figure 7:**
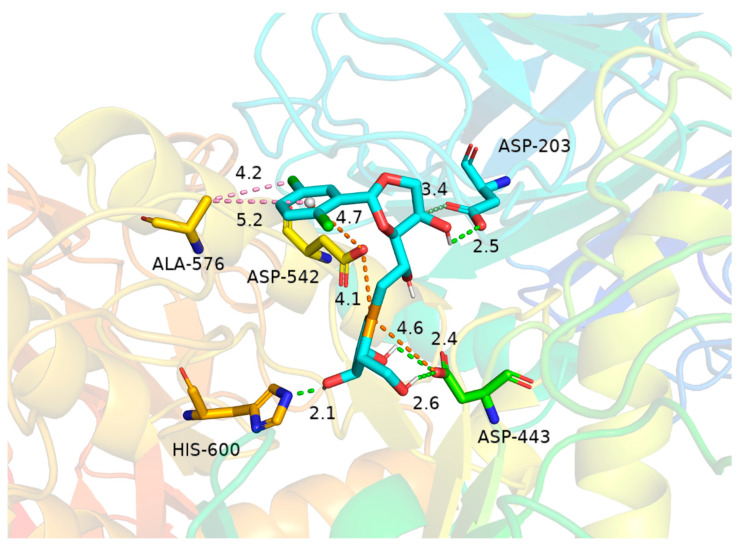
Interactions of **20c** with human N-terminal maltase-glucoamylase (hNtMGAM) (PDB ID: 3L4U). Dotted lines show hydrogen bonding (light green); dotted lines with yellow color show salt bridges. The hydrophobic interaction between ALA-576 and chlorinated substituent and aromatic ring shown in the pink dotted lines were calculated to be 4.2 Å. The electrostatic interaction between two chloride atoms of **20c** and the amino acid residues Asp 542 and ASP 443 was calculated to be 4.1 Å and 4.6 Å, respectively.

In molecular docking studies, binding energy serves as a critical parameter for evaluating the binding stability between ligands and receptors. Generally, a binding energy below 0 kcal/mol indicates spontaneous binding, while values lower than −5 kcal/mol typically indicate strong binding affinity. In this study, molecular docking revealed a binding energy of −6.8 kcal/mol for the ligand–target protein complex, suggesting a thermodynamically favorable and high-affinity interaction. Analysis of the binding mode demonstrated that Asp-203 (2.5 Å), His-600 (2.1 Å), and Asp-443 (2.4 Å and 2.6 Å) stabilize the complex through hydrogen bonding interactions. Notably, Asp-203 also forms a carbon–hydrogen bond with the hydroxyl-bearing carbon atom in the ligand’s dioxane ring. Furthermore, the selenium atom participates in attractive charge interactions with Asp-443 and Asp-542, while Asp-542 engages in a π-anion interaction with the aromatic ring of the ligand, collectively enhancing binding affinity and specificity. Concurrently, Ala-576 contributes to hydrophobic stabilization via π-alkyl and alkyl interactions with the chlorinated substituent and aromatic ring. These synergistic interactions reinforce complex stability. Collectively, the results suggest that this ligand exhibits both favorable binding energetics and a pharmacologically relevant interaction pattern within the target protein’s active site (Figure 7).

## 3. Experimental Section

### 3.1. Materials and Instruments

Reagents and Solvents: All solvents were purified and dried according to standard methods. PE refers to petroleum ether (b.p. 60–90 °C), EA refers to ethyl acetate, DCM refers to methylene dichloride, and DMF refers to dimethyl formamide.

Chromatography: Flash column chromatography was carried out using commercially available 200–300 mesh under pressure and conducted by eluting with PE/EA, which are listed as volume/volume ratios.

Data Collection: The melting point (m.p.) was measured on a microscopic melting point apparatus. ^1^H NMR spectra were collected on a BRUKER AV-300 (Billerica, MA, USA, 300 MHz) spectrometer using CDCl_3_ or DMSO-*d*_6_ or methanol-*d*_4_ as solvent. Chemical shifts of ^1^H NMR were recorded in parts per million (ppm, δ) relative to tetramethylsilane (δ = 0.00 ppm) with the solvent resonance as an internal standard (CDCl_3_, δ = 7.26 ppm, DMSO-*d*_6_, δ = 2.50 ppm, methanol-*d*_4,_ δ = 3.31 ppm). Data are reported as follows: chemical shift in ppm (δ), multiplicity (s = singlet, d = doublet, t = triplet, q = quartet, brs = broad singlet, m = multiplet), coupling constant (Hz), and integration. ^13^C NMR spectra were collected on a BRUKER AV-300 (75 MHz) and BRUKER AVANCE500 (125 MHz) spectrometer using CDCl_3_ or DMSO-*d*_6_ or methanol-*d*_4_ as solvent. Chemical shifts of ^13^C NMR were reported in ppm with the solvent as the internal standard (chloroform-*d*_6_, δ = 77.0 ppm, DMSO-*d*_6_, δ = 39.5 ppm, methanol-*d*_4,_ δ = 47.1 ppm). Low-resolution mass spectra (MS) were measured on Finnigan MAT 95 spectrometer (Finnigan, München, Germany). Highresolution mass measurement was performed on Agilent QTOF 6520 mass spectrometer (Santa Clara, CA, USA) with electron spray ionization (ESI) as the ion source. All compounds selected for in vivo hypoglycemic effect investigation were >95% pure according to HPLC (SHIMADZU Labsolutions, Kyoto, Japan, UV detection at *λ* = 230 nm) analysis on the Agilent C18 column (4.6 × 150 mm^2^, 5 μm) eluting at 1 mL/min of 90% methanol/10% water.

### 3.2. Synthesis

#### 3.2.1. Preparation of 5-*O*-Tosyl-d-arabinitol (**12**)

To a solution of **11** (20.0 g, 50.75 mmol) in DCM (100 mL) was added 30% aq TFA (500 mL), and the reaction mixture was stirred at room temperature for 4 h. The reaction mixture was concentrated under reduced pressure, and the resulting residue was purified by flash column chromatography (DCM/MeOH = 20:1) on silica gel to afford the pure product **12** (11.34 g, 73%) as a white solid, m.p. 285–286 °C. ${\left[\alpha \right]}_{D}^{25}$ = −37.2 (c = 1.00, MeOH). ^1^H NMR (300 MHz, DMSO-*d*_6_) *δ* 7.78 (d, *J* = 8.0 Hz, 2H), 7.48 (d, *J* = 8.0 Hz, 2H), 4.19 (dd, *J* = 9.6, 2.2 Hz, 1H), 4.04 (br,4H), 3.93 (dd, *J* = 9.6, 6.6 Hz, 1H), 3.65 (m, *J* = 17.5, 6.6, 1.8 Hz, 2H), 3.34 (dd, *J* = 6.6, 2.3 Hz, 2H), 3.31−3.25 (m, 1H), 2.42 (s, 3H). ^13^C NMR (75 MHz, DMSO-*d*_6_) 144.8, 132.5, 130.2, 127.7, 73.8, 69.8, 69.5, 68.4, 62.6, 21.2. MS (ESI) 307.1 (M + 1).

#### 3.2.2. General Procedure for the Synthesis of Acetals (**13a**–**r**)

To a solution of **12** (612 mg, 2.0 mmol) in MeCN (10 mL) were added the appropriate benzaldehyde (3.0 mmol) and *p*-toluenesulfonic acid (50 mg); the reaction mixture was stirred at room temperature for 24 h. The reaction mixture was neutralized by triethylamine. The reaction mixture was concentrated under reduced pressure, and the resulting residue was purified by flash column chromatography (PE/EtOAc = 2/1) on silica gel to afford the pure products **13a**–**r** as pale-yellow oils with yields from 41 to 60%.

*5-O-tosyl-1,3-O-*(*2,3-Dichlorobenzylidene*)*-d-arabinitol* (**13a**). **13a** can be obtained from **12** with a yield of 49%. Pale-yellow oils. ${\left[\alpha \right]}_{D}^{25}$ = −14.5 (*c* = 0.50, MeOH). ^1^H NMR (300 MHz, chloroform-*d*) *δ* 7.74 (d, *J* = 8.0 Hz, 2H), 7.50 (d, *J* = 7.6 Hz, 1H), 7.43 (t, *J* = 7.5 Hz, 1H), 7.23 (t, *J* = 5.9 Hz, 2H), 7.19–7.11 (m, 1H), 5.64 (s, 1H), 4.23 (t, *J* = 9.7 Hz, 2H), 4.09–4.02 (m, 3H), 3.85 (s, 2H), 3.74 (b r, 1H), 3.62 (br,1H), 2.35 (s, 3H). ^13^C NMR (75 MHz, CDCl_3_) *δ* 145.2, 136.8, 133.1, 132.2, 131.2, 131.1, 129.9, 128.0, 127.5, 125.8, 98.5, 78.2, 72.6, 71.3, 67.2, 62.5, 21.7. MS (ESI) 463.0 (m + 1).

*5-O-Tosyl-1,3-O-*(*2,4-dichlorobenzylidene*)*-d-arabinitol* (**13b**). **13b** can be obtained from **12** with a yield of 54%. Pale-yellow oils. ${\left[\alpha \right]}_{D}^{25}$ = −23.6 (*c* = 0.50, MeOH). ^1^H NMR (300 MHz, chloroform-*d*) *δ* 7.73 (d, *J* = 8.1 Hz, 2H), 7.50 (d, *J* = 8.4 Hz, 1H), 7.36–7.32 (m, 1H), 7.25–7.18 (m, 3H), 5.61 (s, 1H), 4.27–4.18 (m, 2H), 4.06 (t, *J* = 4.4 Hz, 3H), 4.00 (br, 1H), 3.89–3.81 (m, 2H), 3.50 (br, 1H), 2.36 (s, 3H), ^13^C NMR (75 MHz, CDCl_3_) *δ* 145.1, 135.5, 133.5, 133.3, 132.3, 129.9, 129.3, 128.7, 128.0, 127.2, 97.9, 78.1, 72.6, 71.3, 67.2, 62.4, 21.7. MS (ESI) 463.0 (M + 1).

*5-O-Tosyl-1,3-O-*(*2,5-dichlorobenzylidene*)*-d-arabinitol* (**13c**). **13c** can be obtained from **12** with a yield of 44%. Pale-yellow oils. ${\left[\alpha \right]}_{D}^{25}$ = −11.6 (c = 0.50, MeOH). ^1^H NMR (300 MHz, chloroform-*d*) *δ* 7.75 (d, *J* = 8.2 Hz, 2H), 7.55 (s, 1H), 7.29–7.21 (m, 4H), 5.61 (s, 1H), 4.31–4.19 (m, 2H), 4.12–4.04 (m, 3H), 3.88–3.81 (m, 2H), 3.49 (s, 1H), 2.34 (s, 3H). ^13^C NMR (75 MHz, CDCl_3_) *δ* 145.0, 136.2, 132.8, 132.2, 131.1, 130.7, 130.3, 130.0, 129.9, 128.0, 127.8, 97.7, 78.1, 72.6, 71.2, 67.1, 62.4, 21.7. MS (ESI) 463.0 (M + 1).

*5-O-Tosyl-1,3-O-*(*2-chloro-3-fluorobenzylidene*)*-d-arabinitol* (**13d**). **13d** can be obtained from **12** with a yield of 43%. Pale-yellow oils. ${\left[\alpha \right]}_{D}^{25}$ = −22.3(*c* = 0.50, MeOH). ^1^H NMR (300 MHz, chloroform-*d*) *δ* 7.75 (d, *J* = 8.2 Hz, 2H), 7.39 (d, *J* = 7.7 Hz, 1H), 7.33–7.28 (m, 1H), 7.25 (d, *J* = 8.1 Hz, 2H), 7.16 (d, *J* = 8.4 Hz, 1H), 5.65 (s, 1H), 4.27–4.21 (m, 2H), 4.12–4.04 (m, 3H), 3.89–3.84 (m, 2H), 3.35 (br, 1H), 2.37 (s, 3H). ^13^C NMR (75 MHz, CDCl_3_) *δ* 158.0 (d, *J* = 247.9 Hz), 145.1, 136.8, 132.3, 130.0, 129.9, 128.1, 128.0, 127.7 (d, *J* = 7.8 Hz), 122.7 (d, *J* = 3.4 Hz), 120.0 (d, *J* = 18.9 Hz), 119.9, 117.1 (d, *J* = 21.3 Hz), 98.0 (d, *J* = 3.0 Hz), 78.2, 72.6, 71.3, 67.2, 62.5, 21.6. MS (ESI) 447.1 (M + 1).

*5-O-Tosyl-1,3-O-*(*2-chloro-5-fluorobenzylidene*)*-d-arabinitol* (**13e**). **13e** can be obtained from **12** with a yield of 43%. Pale-yellow oils. ${\left[\alpha \right]}_{D}^{25}$ = −24.2(c = 0.50, MeOH). ^1^H NMR (300 MHz, chloroform-*d*) *δ* 7.70 (dd, *J* = 8.4, 2.0 Hz, 2H), 7.26–7.19 (m, 4H), 6.98–6.91 (m, 1H), 5.57 (s, 1H), 4.23–4.13 (m, 2H), 4.08–4.00 (m, 4H), 3.82–3.77 (m, 2H), 3.17 (s, 1H), 2.92 (s, 1H), 2.32 (s, 3H). ^13^C NMR (75 MHz, CDCl_3_) *δ* 145.2, 136.5 (d, *J* = 7.6 Hz), 132.3, 131.0 (d, *J* = 8.1 Hz), 130.1, 130.0, 128.1, 127.7 (d, *J* = 3.0 Hz), 117.5 (d, *J* = 23.0 Hz), 114.9 (d, *J* = 24.9 Hz), 98.0, 78.3, 72.7, 71.1, 67.5, 62.6, 21.7. MS (ESI) 447.1 (M + 1).

*5-O-Tosyl-1,3-O-*(*3,4-Dichlorobenzylidene*)*-d-arabinitol* (**13f**). **13f** can be obtained from **12** with a yield of 42%. Pale-yellow oils. ${\left[\alpha \right]}_{D}^{25}$ = −33.7 (*c* = 0.50,MeOH). ^1^H NMR (300 MHz, chloroform-*d*) *δ* 7.80–7.75 (m, 2H), 7.44–7.42 (m, 1H), 7.39 (d, *J* = 8.3 Hz, 1H), 7.27 (d, *J* = 8.2 Hz, 2H), 7.18 (dd, *J* = 8.3, 2.1 Hz, 1H), 5.40 (s, 1H), 4.22–4.18 (m, 2H), 4.10–4.02 (m, 2H), 3.90–3.87 (m, 1H), 3.87–3.80 (m, 2H), 3.65 (br, 2H), 2.36 (s, 3H). ^13^C NMR (75 MHz, CDCl_3_) *δ* 145.3, 137.5, 133.1, 132.4, 132.3, 130.3, 130.0, 128.1, 128.0, 125.6, 99.3, 77.7, 72.4, 70.9, 67.3, 62.5, 21.7. MS (ESI) 463.0 (M + 1).

*5-O-Tosyl-1,3-O-*(*3,5-Dichlorobenzylidene*)*-d-arabinitol* (**13g**). **13g** can be obtained from **12** with a yield of 55%. Pale-yellow oils. ${\left[\alpha \right]}_{D}^{25}$ = −33.7 (*c* = 0.50, MeOH). ^1^H NMR (300 MHz, chloroform-*d*) *δ* 7.80 (d, *J* = 8.3 Hz, 2H), 7.35–7.33 (m, 2H), 7.31 (s, 1H), 7.26 (d, *J* = 2.0 Hz, 2H), 5.41 (s, 1H), 4.24 (d, J = 3.0 Hz, 2H), 4.10–4.03 (m, 2H), 3.91–3.82 (m, 3H), 3.09 (br, 1H), 2.86 (br, 1H), 2.39 (s, 3H). ^13^C NMR (75 MHz, CDCl_3_) *δ* 145.4, 140.4, 135.0, 132.4, 132.4, 130.1, 129.2, 128.1, 124.7, 99.2, 77.9, 72.5, 70.8, 67.6, 62.6, 21.8. MS (ESI) 463.0 (M + 1).

*5-O-Tosyl-1,3-O-*(*3-Chloro-5-fluorobenzylidene*)*-d-arabinitol* (**13h**). **13h** can be obtained from **12** with a yield of 41%. Pale-yellow oils. ${\left[\alpha \right]}_{D}^{25}$ = −13.7 (*c* = 0.50, MeOH). ^1^H NMR (300 MHz, chloroform-*d*) *δ* 7.81 (d, *J* = 8.1 Hz, 2H), 7.33 (d, *J* = 8.1 Hz, 3H), 7.09 (dq, *J* = 8.3, 2.8, 2.2 Hz, 1H), 6.98 (dt, *J* = 8.9, 1.9 Hz, 1H), 5.43 (s, 1H), 4.30–4.20 (m, 3H), 4.07 (d, *J* = 11.5 Hz, 2H), 3.94–3.86 (m, 3H), 3.19 (d, *J* = 6.4 Hz, 1H), 2.92 (d, *J* = 10.3 Hz, 1H), 2.41 (s, 3H). ^13^C NMR (75 MHz, CDCl_3_) *δ* 145.4, 140.9(d, *J* = 8.5 Hz), 135.1 (d, *J* = 10.6 Hz), 132.5, 130.1, 128.2, 128.1, 122.3 (d, *J* = 3.15 Hz), 116.8 (d, *J* = 24.6 Hz), 111.9 (d, *J* = 23.0 Hz), 99.2, 77.9, 72.5, 70.8, 67.6, 62.6, 21.7. MS (ESI) 447.1 (M + 1).

*5-O-Tosyl-1,3-O-*(*3,5-bis*(*trifluoromethyl*)*benzylidene*)*-d-arabinitol* (**13i**). **13i** can be obtained from **12** with a yield of 56%. Pale-yellow oils. ${\left[\alpha \right]}_{D}^{25}$ = −55.4 (c = 0.50, MeOH). ^1^H NMR (300 MHz, chloroform-*d*) *δ* 7.88 (d, *J* = 5.4 Hz, 3H), 7.81–7.78 (m, 2H), 7.30 (d, *J* = 8.1 Hz, 2H), 5.59 (s, 1H), 4.35–4.29 (m, 1H), 4.24 (d, *J* = 3.7 Hz, 2H), 4.14 (d, *J* = 12.1 Hz, 2H), 3.95 (d, *J* = 13.4 Hz, 2H), 3.46 (br, 2H), 2.38 (s, 3H). ^13^C NMR (75 MHz, CDCl_3_) *δ* 145.5, 139.9, 132.4, 131.7 (q, *J* = 33.6 Hz), 130.3, 130.1, 128.1, 126.7, 124.07 (q, *J* = 161.5 Hz), 99.0, 78.1, 72.6, 71.0, 67.5, 62.6. MS (ESI) 531.0 (M + 1).

*5-O-Tosyl-1,3-O-*(*5-fluoro-2-nitrobenzylidene*)*-d-arabinitol* (**13j**). **13*j*** can be obtained from **12** with a yield of 51%. Pale-yellow oils. ${\left[\alpha \right]}_{D}^{25}$ = −38.7 (c = 0.50, MeOH). ^1^H NMR (300 MHz, chloroform-*d*) *δ* 7.94–7.90 (m, 1H), 7.81 (d, *J* = 6.4 Hz, 2H), 7.45 (d, *J* = 8.6 Hz, 1H), 7.32 (d, *J* = 8.0 Hz, 2H), 7.19–7.14 (m, 1H), 6.04 (s, 1H), 4.22 (d, *J* = 10.8 Hz, 2H), 4.07 (t, *J* = 8.8 Hz, 3H), 3.86 (d, *J* = 8.6 Hz, 2H), 3.31 (br, 2H), 2.39 (s, 3H). ^13^C NMR (75 MHz, CDCl_3_) *δ* 166.2, 162.8, 145.2, 144.2, 134.78 (d, *J* = 9.0Hz), 132.4, 130.0, 128.1, 127.24 (*J* = 9.5Hz), 116.60 (d, *J* = 23.5 Hz), 115.18 (d, *J* = 26.1 Hz), 96.3, 78.6, 72.8, 71.3, 67.4, 62.4, 21.7. MS (ESI) 458.1 (M + 1).

*5-O-Tosyl-1,3-O-*(*2-bromobenzylidene*)*-d-arabinitol* (**13k**). **13k** can be obtained from **12** with a yield of 54%. Pale-yellow oils. ${\left[\alpha \right]}_{D}^{25}$ = −43.2 (*c* = 0.50, MeOH). ^1^H NMR (300 MHz, chloroform-*d*) *δ* 7.73 (d, *J* = 8.3 Hz, 2H), 7.58–7.52 (m, 1H), 7.50 (d, *J* = 1.4 Hz, 1H), 7.29–7.22 (m, 1H), 7.19 (d, *J* = 8.5 Hz, 3H), 5.56 (s, 1H), 4.31–4.22 (m, 1H), 4.18 (d, *J* = 12.9 Hz, 1H), 4.10–3.97 (m, 3H), 3.83 (d, *J* = 5.5 Hz, 2H), 3.74 (br, 2H), 2.31 (s, 3H). ^13^C NMR (75 MHz, CDCl_3_) *δ* 144.9, 136.1, 132.7, 132.3, 130.6, 129.8, 127.9, 127.8, 127.4, 122.3, 100.2, 78.0, 72.5, 71.3, 67.0, 62.3, 21.6. MS (ESI) 473.0 (M + 1).

*5-O-Tosyl-1,3-O-*(*m-bromobenzylidene*)*-d-arabinitol* (**13l**). **13l** can be obtained from **12** with a yield of 53%. Pale-yellow oils. ${\left[\alpha \right]}_{D}^{25}$ = −30.2 (*c* = 0.50, MeOH). ^1^H NMR (300 MHz, chloroform-*d*) *δ* 7.78 (d, *J* = 7.8 Hz, 2H), 7.48 (s, 1H), 7.32–7.22 (m, 4H), 7.22–7.17 (m, 1H), 5.38 (s, 1H), 4.19 (q, *J* = 11.8, 11.0 Hz, 3H), 4.10–3.99 (m, 2H), 3.87–3.80 (m, 2H), 3.71 (br, 1H), 3.28 (br, 1H), 2.34 (s, 3H). ^13^C NMR (75 MHz, CDCl_3_) *δ* 145.2, 139.5, 132.4, 132.1, 130.0, 129.9, 129.1, 128.0, 124.8, 122.3, 99.8, 77.7, 72.4, 71.0, 67.3, 62.5, 21.7. MS (ESI) 473.0 (M + 1).

*5-O-Tosyl-1,3-O-*(*p-Bromobenzylidene*)*-d-arabinitol* (**13m**). **13m** can be obtained from **12** with a yield of 60%. Pale-yellow oils. ${\left[\alpha \right]}_{D}^{25}$ = −42.2 (*c* = 0.50, MeOH). ^1^H NMR (300 MHz, chloroform-*d*) *δ* 7.79 (d, *J* = 8.4 Hz, 2H), 7.48 (d, *J* = 7.9 Hz, 2H), 7.31 (d, 1H), 7.29–7.27 (d, 2H), 7.24 (d, 1H), 5.44 (s, 1H), 4.29–4.20 (m, 3H), 4.14–4.03 (m, 2H), 3.90–3.82 (m, 2H), 3.11 (br, 1H), 2.96 (br, 1H), 2.41 (s, 3H). ^13^C NMR (75 MHz, CDCl_3_) *δ* 145.3, 136.4, 132.5, 131.7, 131.5, 130.1, 128.1, 127.8, 123.3, 100.3, 78.0, 72.5, 70.9, 67.6, 62.7, 21.8. MS (ESI) 473.0 (M + 1).

*5-O-Tosyl-1,3-O-*(*2-fluorophenylmethylene*)*-d-arabinitol* (**13n**). **13n** can be obtained from **12** with a yield of 58%. Colorless oil. ${\left[\alpha \right]}_{D}^{25}$ = −9.9 (c = 0.32, MeOH). ^1^H NMR (300 MHz, chloroform-*d*) *δ* 7.75 (d, *J* = 8.2 Hz, 2H), 7.51 (tdd, *J* = 7.5, 5.3, 1.9 Hz, 1H), 7.34 (tdd, *J* = 7.5, 5.3, 1.9 Hz, 1H), 7.24 (d, *J* = 8.2 Hz, 2H), 7.13 (td, *J* = 7.5, 1.1 Hz, 1H), 7.04 (ddd, *J* = 9.8, 8.3, 1.1 Hz, 1H), 5.68 (s, 1H), 4.29–4.23 (m, 1H), 4.23–4.17 (m, 1H), 4.13–4.02 (m, 3H), 3.87–3.89 (m, 2H), 3.19 (br., 2H), 2.36 (s, 3H). ^13^C NMR (75 MHz, CDCl_3_) *δ* 161.7, 145.1, 131.0 (d, *J*_C-F_ = 8.1 Hz), 130.0, 129.9, 128.1, 128.0, 127.6 (d, *J*_C-F_ = 3.5 Hz), 124.1 (d, *J*_C-F_ = 3.6 Hz), 115.5 (d, *J*_C-F_ = 21.3 Hz), 96.6, 78.2, 72.6, 71.2, 67.5, 62.6, 21.7. MS (ESI) 413.1 (M + 1).

*5-O-Tosyl-1,3-O-*(*2-iodobenzylidene*)*-d-arabinitol* (**13o**). **13o** can be obtained from **12** with a yield of 57%. Colorless oil. ${\left[\alpha \right]}_{D}^{25}$ = −27.4 (c = 0.50, MeOH). ^1^H NMR (300 MHz, chloroform-*d*) *δ* 7.82 (d, *J* = 7.8 Hz, 1H), 7.77 (d, *J* = 8.1 Hz, 2H), 7.54 (dd, *J* = 7.8, 1.7 Hz, 1H), 7.33 (t, *J* = 7.4 Hz, 1H), 7.24 (d, *J* = 8.0 Hz, 2H), 7.06 (td, *J* = 7.6, 1.8 Hz, 1H), 5.43 (s, 1H), 4.33 (d, *J* = 8.8 Hz, 1H), 4.27–4.19 (m, 1H), 4.16 (d, *J* = 5.4 Hz, 1H), 4.08 (t, *J* = 10.0 Hz, 2H), 3.87 (d, *J* = 8.1 Hz, 2H), 3.39 (br, 2H), 2.36 (s, 3H). ^13^C NMR (75 MHz, CDCl_3_) *δ* 145.0, 139.4, 138.9, 132.4, 130.8, 129.9, 128.2, 128.0, 127.6, 103.8, 97.0, 78.1, 72.5, 71.5, 67.2, 62.4, 21.7. MS (ESI) 521.0 (M + 1).

*5-O-Tosyl-1,3-O-*(*2-cyanobenzylidene*)*-d-arabinitol* (**13p**). **13p** can be obtained from **12** with a yield of 55%. Pale-yellow oils. ${\left[\alpha \right]}_{D}^{25}$ = −28.7 (*c* = 0.50, MeOH). ^1^H NMR (300 MHz, chloroform-*d*)) *δ* 7.77–7.74 (m, 2H), 7.67 (d, *J* = 7.5 Hz, 1H), 7.60–7.55 (m, 2H), 7.48–7.44 (m, 1H), 7.24 (d, *J* = 8.3Hz, 2H), 5.63 (s, 1H), 4.30–4.22 (m, 3H), 4.14–4.10 (m, 3H), 3.90 (d, *J* = 8.9 Hz, 2H), 3.62 (br, 1H), 2.35 (s, 3H). ^13^C NMR (75 MHz, CDCl_3_) *δ* 145.0, 140.4, 133.3, 132.9, 132.4, 129.9, 129.7, 128.1, 128.0, 127.7, 118.2, 110.6, 99.6, 78.2, 72.5, 71.4, 67.2, 62.6, 21.7. MS (ESI) 420.1 (M + 1).

*5-O-Tosyl-1,3-O-*(*1-naphthalenebenzenemethylene*)*-d-arabinitol* (**13q**). **13q** can be obtained from **12** with a yield of 52%. Pale-yellow oils. ${\left[\alpha \right]}_{D}^{25}$ = −24.7 (*c* = 0.50, MeOH). ^1^H NMR (300 MHz, chloroform-*d*) *δ* 7.95 (d, *J* = 7.5 Hz, 1H), 7.83 (t, *J* = 8.1 Hz, 2H), 7.69 (d, *J* = 8.0 Hz, 2H), 7.64 (d, *J* = 7.4 Hz, 1H), 7.51 (dt, *J* = 9.5, 5.8 Hz, 2H), 7.39 (t, *J* = 7.7 Hz, 1H), 7.05 (d, *J* = 8.0 Hz, 2H), 5.91 (s, 1H), 4.21 (d, *J* = 12.2 Hz, 1H), 4.17–4.12 (m, 2H), 4.07 (dd, *J* = 10.6, 5.0 Hz, 2H), 3.94 (d, *J* = 8.9 Hz, 1H), 3.84 (s, 1H), 3.29 (br, 2H), 2.15 (s, 3H). ^13^C NMR (75 MHz, CDCl_3_) *δ* 145.0, 133.7, 132.6, 132.3, 130.4, 129.9, 129.8, 128.7, 128.0, 126.6, 125.9, 125.1, 123.8, 123.7, 99.3, 77.9, 72.5, 71.1, 67.3, 62.7, 21.5. MS (ESI) 445.1 (M + 1).

*5-O-Tosyl-1,3-O-*(*2-phenylbenzylidene*)*-d-arabinitol* (**13r**). **13r** can be obtained from **12** with a yield of 57%. Pale-yellow oils. ${\left[\alpha \right]}_{D}^{25}$ = −20.9 (c = 0.50, MeOH). ^1^H NMR (300 MHz, chloroform-*d*) *δ* 7.81 (d, *J* = 8.1 Hz, 2H), 7.76 (dd, *J* = 7.3, 1.9 Hz, 1H), 7.51–7.42 (m, 6H), 7.41–7.38 (m, 2H), 7.35–7.30 (m, 2H), 5.45 (s, 1H), 4.24–4.16 (m, 2H), 4.13–3.99 (m, 2H), 3.87 (d, *J* = 12.1 Hz, 1H), 3.78 (s, 1H), 3.67 (d, *J* = 8.3 Hz, 1H), 3.43 (br, 2H), 2.44 (s, 3H). ^13^C NMR (75 MHz, CDCl_3_) *δ* 145.0, 141.1, 140.2, 134.7, 132.5, 130.1, 129.9, 129.3, 129.2, 128.2, 128.0, 127.6, 127.5, 126.6, 100.0, 78.1, 72.3, 71.4, 67.3, 62.5, 21.7. MS (ESI) 471.1 (M + 1).

#### 3.2.3. General Procedure for the Synthesis of Epoxides (**14a**–**r**)

To a solution of **13a**–**r** (2.0 mmol) in DCM (10 mL) under an argon atmosphere was added DBU (2.5 mL) at 0 °C. The mixture was stirred at room temperature for 2 h. The reaction mixture was concentrated under reduced pressure, and the resulting residue was purified by flash column chromatography (PE/EtOAc, 5:1) on silica gel to afford the pure products **14a**–**r** as colorless oil with yields from 85 to 93%.

*4,5-Anhydro-1,3-O-*(*2,3-dichlorobenzylidene*)*-d-arabinitol* (**14a**). **14a** can be obtained from **13a** with a yield of 85%. Colorless oil. ${\left[\alpha \right]}_{D}^{25}$ = –31.6 (c = 0.50, MeOH). ^1^H NMR (300 MHz, chloroform-*d*) *δ* 7.61 (d, *J* = 7.7 Hz, 1H), 7.46 (d, *J* = 8.0 Hz, 1H), 7.27–7.21 (m, 1H), 5.82 (s, 1H), 4.23 (dt, *J* = 12.0, 1.8 Hz, 1H), 4.08 (d, *J* = 12.1 Hz, 1H), 3.76 (d, *J* = 5.3 Hz, 2H), 3.33–3.29 (m, 1H), 2.98 (br, 1H), 2.91–2.88 (m, 1H), 2.82 (dt, *J* = 4.6, 2.0 Hz, 1H). ^13^C NMR (75 MHz, CDCl_3_) *δ* 136.8, 133.3, 131.3, 127.6, 126.0, 99.1, 80.2, 72.6, 64.3, 50.7, 45.9. MS (ESI) 291.0 (M + 1).

*4,5-Anhydro-1,3-O-*(*2,4-dichlorobenzylidene*)*-d-arabinitol* (**14b**). **14b** can be obtained from **13b** with a yield of 89%. Colorless oil. ${\left[\alpha \right]}_{D}^{25}$ = –26.4 (c = 0.50, MeOH). ^1^H NMR (300 MHz, chloroform-*d*) *δ* 7.64 (d, *J* = 8.4 Hz, 1H), 7.39 (d, *J* = 2.1 Hz, 1H), 7.29 (dd, *J* = 8.3, 2.0 Hz, 1H), 5.78 (s, 1H), 4.24 (dd, *J* = 12.1, 1.8 Hz, 1H), 4.08 (dd, *J* = 12.1, 1.4 Hz, 1H), 3.78–3.74 (m, 2H), 3.32 (ddd, *J* = 5.4, 3.9, 2.6 Hz, 1H), 3.06 (d, *J* = 9.5 Hz, 1H), 2.91 (t, *J* = 4.5 Hz, 1H), 2.84–2.81 (m, 1H). ^13^C NMR (75 MHz, CDCl_3_) *δ* 135.8, 133.5, 133.3, 129.5, 128.9, 127.4, 98.5, 80.1, 72.6, 64.3, 50.7, 45.9. MS (ESI) 291.0 (M + 1).

*4,5-Anhydro-1,3-O-*(*2,5-dichlorobenzylidene*)*-d-arabinitol* (**14c**). **14c** can be obtained from **13c** with a yield of 88%. Colorless oil. ${\left[\alpha \right]}_{D}^{25}$ = –16.3 (c = 0.50, MeOH). ^1^H NMR (300 MHz, chloroform-*d*) *δ* 7.70 (d, *J* = 2.1 Hz, 1H), 7.27 (d, *J* = 4.2 Hz, 2H), 5.79 (s, 1H), 4.25 (dd, *J* = 12.2, 1.9 Hz, 1H), 4.09 (d, *J* = 12.1 Hz, 1H), 3.80–3.75 (m, 2H), 3.34 (p, *J* = 2.9 Hz, 1H), 3.03 (br, 1H), 2.93 (t, *J* = 4.5 Hz, 1H), 2.85–2.83 (m, 1H). ^13^C NMR (75 MHz, CDCl_3_) *δ* 136.1, 133.1, 131.1, 130.8, 130.6, 128.1, 98.4, 80.2, 72.6, 64.3, 50.7, 46.0. MS (ESI) 291.0 (M + 1).

*4,5-Anhydro-1,3-O-*(*2-chloro-3-fluorobenzylidene*)*-d-arabinitol* (**14d**). **14d** can be obtained from **13d** with a yield of 88%. Colorless oil. ${\left[\alpha \right]}_{D}^{25}$ = –23.1 (c = 0.50, MeOH). ^1^H NMR (300 MHz, chloroform-*d*) *δ* 7.43 (d, *J* = 7.9 Hz, 1H), 7.23–7.17 (m, 1H), 7.09 (t, *J* = 8.6 Hz, 1H), 5.75 (s, 1H), 4.17 (d, *J* = 12.1 Hz, 1H), 4.02 (d, *J* = 1 2.1 Hz, 1H), 3.70 (d, *J* = 5.3 Hz, 2H), 3.27–3.23 (m, 1H), 2.99 (d, *J* = 9.1 Hz, 1H), 2.85–2.82 (m, 1H), 2.76 (dd, *J* = 5.1, 2.6 Hz, 1H). ^13^C NMR (75 MHz, CDCl_3_) *δ* 158.1 (d, *J* = 248.1 Hz), 136.8, 127.9 (d, *J* = 7.7 Hz), 123.0 (d, *J* = 3.7 Hz), 120.1 (d, *J* = 18.6 Hz)117.3 (d, *J* = 21.1 Hz), 98.5 (d, *J* = 3.9 Hz). 80.1, 72.6, 64.3, 50.7, 45.9. MS (ESI) 275.0 (M + 1).

*4,5-Anhydro-1,3-O-*(*2-chloro-5-fluorobenzylidene*)*-d-arabinitol* (**14e**). **14e** can be obtained from **13e** with a yield of 91%. Colorless oil. ${\left[\alpha \right]}_{D}^{25}$ = –26.3 (c = 0.50, MeOH). ^1^H NMR (300 MHz, chloroform-*d*) *δ* 7.43 (dd, *J* = 9.1, 3.0 Hz, 1H), 7.32 (dd, *J* = 8.9, 5.0 Hz, 1H), 7.02 (tdd, *J* = 7.5, 3.1, 1.2 Hz, 1H), 5.78 (s, 1H), 4.25 (d, *J* = 12.1 Hz, 1H), 4.09 (d, *J* = 12.2 Hz, 1H), 3.80–3.76 (m, 2H), 3.33 (ddd, *J* = 5.1, 3.9, 2.4 Hz, 1H), 2.99 (d, *J* = 10.1 Hz, 1H), 2.92 (td, *J* = 4.5, 3.9, 1.3 Hz, 1H), 2.85–2.82 (m, 1H). ^13^C NMR (75 MHz, CDCl_3_) *δ* 161.4 (d, *J* = 246.8 Hz), 136.5 (d, *J* = 7.6 Hz), 131.0 (d, *J* = 8.1 Hz), 127.6 (d, *J* = 3.3 Hz), 117.6 (d, *J* = 22.9 Hz), 115.2 (d, *J* = 24.8 Hz). 98.4, 80.2, 72.6, 64.3, 50.7, 45.9. MS (ESI) 275.0 (M + 1).

*4,5-Anhydro-1,3-O-*(*3,4-Dichlorobenzylidene*)*-d-arabinitol* (**14f**). **14f** can be obtained from **13f** with a yield of 94%. Colorless oil. ${\left[\alpha \right]}_{D}^{25}$ = –53.2 (c = 0.50, MeOH). ^1^H NMR (300 MHz, chloroform-*d*) *δ* 7.61 (d, *J* = 2.0 Hz, 1H), 7.44 (d, *J* = 8.3 Hz, 1H), 7.32 (dd, *J* = 8.3, 2.0 Hz, 1H), 5.50 (s, 1H), 4.24 (dd, *J* = 12.1, 1.9 Hz, 1H), 4.05 (dd, *J* = 12.1, 1.4 Hz, 1H), 3.75 (t, *J* = 2.4 Hz, 2H), 3.31 (ddd, *J* = 5.0, 4.0, 2.6 Hz, 1H), 3.02 (br, 1H), 2.92 (dd, *J* = 5.1, 4.0 Hz, 1H), 2.85 (dd, *J* = 5.0, 2.7 Hz, 1H). ^13^C NMR (75 MHz, CDCl_3_) *δ* 137.5, 133.2, 132.5, 130.4, 128.3, 125.6, 99.7, 79.5, 72.3, 64.2, 50.9, 45.9. MS (ESI) 291.0 (M + 1).

*4,5-Anhydro-1,3-O-*(*3,5-dichlorobenzylidene*)*-d-arabinitol* (**14g**). **14g** can be obtained from **13g** with a yield of 93%. Colorless oil. ${\left[\alpha \right]}_{D}^{25}$ = –38.4 (c = 0.50, MeOH). ^1^H NMR (300 MHz, chloroform-*d*) *δ* 7.39 (d, *J* = 2.0 Hz, 2H), 7.35 (t, *J* = 2.0 Hz, 1H), 5.49 (s, 1H), 4.24 (dd, *J* = 12.1, 1.8 Hz, 1H), 4.04 (dd, *J* = 12.1, 1.4 Hz, 1H), 3.78–3.75 (m, 2H), 3.32 (ddd, *J* = 5.0, 4.0, 2.6 Hz, 1H), 3.05 (d, *J* = 10.0 Hz, 1H), 2.93 (dd, *J* = 5.1, 3.9 Hz, 1H), 2.85 (dd, *J* = 5.0, 2.6 Hz, 1H). ^13^C NMR (75 MHz, CDCl_3_) *δ* 140.4, 135.0, 129.2, 124.9, 99.5, 79.6, 72.3, 64.2, 50.8, 45.9. MS (ESI) 291.0 (M + 1).

*4,5-Anhydro-1,3-O-*(*3-chloro-5-fluorobenzylidene*)*-d-arabinitol* (**14h**). **14h** can be obtained from **13h** with a yield of 84%. Colorless oil. ${\left[\alpha \right]}_{D}^{25}$ = –40.6 (c = 0.50, MeOH). ^1^H NMR (300 MHz, chloroform-*d*) *δ* 7.26 (d, *J* = 1.9 Hz, 1H), 7.07 (ddt, *J* = 12.8, 8.4, 2.1 Hz, 2H), 5.46 (s, 1H), 4.21 (dd, *J* = 12.1, 1.8 Hz, 1H), 4.01 (dd, *J* = 12.1, 1.4 Hz, 1H), 3.73 (d, *J* = 6.0 Hz, 2H), 3.28 (td, *J* = 4.4, 2.5 Hz, 1H), 2.98 (d, *J* = 10.0 Hz, 1H), 2.89 (t, *J* = 4.6 Hz, 1H), 2.82 (dd, *J* = 5.0, 2.7 Hz, 1H). ^13^C NMR (75 MHz, CDCl_3_) *δ* 158.5 (d, *J* = 250.0 Hz), 134.6 (d, *J* = 3.8 Hz),128.7, 126.2 (d, *J* = 7.4 Hz), 121.0 (d, *J* = 18.0 Hz), 116.5 (d, *J* = 21.3 Hz). 99.9, 79.6, 72.3, 64.2, 50.9, 45.9. MS (ESI) 275.0 (M + 1).

*4,5-Anhydro-1,3-O-*(*3,5-bis*(*trifluoromethyl*)*benzylidene*)*-d-arabinitol* (**14i**). **14i** can be obtained from **13i** with a yield of 87%. Colorless oil. ${\left[\alpha \right]}_{D}^{25}$ = –18.1 (c = 0.50, MeOH). ^1^H NMR (300 MHz, chloroform-*d*) *δ* 7.97 (s, 2H), 7.87 (s, 1H), 5.65 (s, 1H), 4.29 (d, *J* = 12.3 Hz, 1H), 4.10 (d, *J* = 12.2 Hz, 1H), 3.80 (d, *J* = 8.8 Hz, 2H), 3.37–3.33 (m, 1H), 3.00–2.93 (m, 2H), 2.87 (dd, *J* = 5.1, 2.7 Hz, 1H). ^13^C NMR (75 MHz, CDCl_3_) *δ* 139.8, 131.8 (q, *J* = 33.7 Hz), 126.7 (d, *J* = 2.61 Hz), 125.1, 123.1 (dq, *J* = 7.6, 3.9 Hz), 121.5, 99.2, 79.7, 72.4, 64.2, 50.8, 45.9. MS (ESI) 359.1 (M + 1).

*4,5-Anhydro-1,3-O-*(*5-fluoro-2-nitrobenzylidene*)*-d-arabinitol* (**14j**). **14*j*** can be obtained from **13*j*** with a yield of 86%. Colorless oil. ${\left[\alpha \right]}_{D}^{25}$ = –11.4 (c = 0.50, MeOH). ^1^H NMR (300 MHz, chloroform-*d*) *δ* 7.96 (dd, *J* = 9.0, 4.9 Hz, 1H), 7.59 (dd, *J* = 9.2, 2.9 Hz, 1H), 7.17 (ddd, *J* = 9.0, 7.1, 2.9 Hz, 1H), 6.17 (s, 1H), 4.23 (dd, *J* = 12.1, 1.8 Hz, 1H), 4.09 (dd, *J* = 12.2, 1.4 Hz, 1H), 3.79–3.75 (m, 2H), 3.29 (ddd, *J* = 5.1, 4.0, 2.6 Hz, 1H), 2.93–2.89 (m, 2H), 2.82–2.80 (m, 1H). ^13^C NMR (75 MHz, CDCl_3_) *δ* 164.7 (d, *J* = 256.5 Hz), 135.0 (d, *J* = 8.9 Hz), 127.5 (d, *J* = 9.6 Hz), 116.7 (d, *J* = 23.3 Hz), 115.3 (d, *J* = 26.0 Hz), 99.7, 96.7, 80.3, 72.7, 64.3, 50.6, 45.7. MS (ESI) 286.1 (M + 1).

*4,5-Anhydro-1,3-O-*(*o-bromobenzylidene*)*-d-arabinitol* (**14k**). **14k** can be obtained from **13k** with a yield of 92%. Colorless oil. ${\left[\alpha \right]}_{D}^{25}$ = –34.7 (c = 0.50, MeOH). ^1^H NMR (300 MHz, chloroform-*d*) *δ* 7.68 (d, *J* = 7.8 Hz, 1H), 7.55 (d, *J* = 8.0 Hz, 1H), 7.35 (t, *J* = 7.6 Hz, 1H), 7.22 (t, *J* = 7.7 Hz, 1H), 5.77 (s, 1H), 4.24 (d, *J* = 12.1 Hz, 1H), 4.08 (d, *J* = 12.0 Hz, 1H), 3.75 (t, *J* = 7.1 Hz, 2H), 3.35–3.31 (m, 1H), 3.07 (d, *J* = 10.0 Hz, 1H), 2.87 (dt, *J* = 18.0, 4.6 Hz, 2H). ^13^C NMR (75 MHz, CDCl_3_) *δ* 136.1, 132.9, 130.9, 128.1, 127.6, 122.4, 101.0, 80.2, 72.6, 64.3, 50.7, 46.0. MS (ESI) 301.0 (M + 1).

*4,5-Anhydro-1,3-O-*(*m-bromobenzylidene*)*-d-arabinitol* (**14l**). **14l** can be obtained from **13l** with a yield of 85%. Colorless oil. ${\left[\alpha \right]}_{D}^{25}$ = –28.9 (c = 0.50, MeOH). ^1^H NMR (300 MHz, chloroform-*d*) *δ* 7.66 (s, 1H), 7.49 (d, *J* = 8.0 Hz, 1H), 7.41 (d, *J* = 7.7 Hz, 1H), 7.24 (t, *J* = 7.8 Hz, 1H), 5.51 (s, 1H), 4.23 (dd, *J* = 12.1, 1.9 Hz, 1H), 4.04 (dd, *J* = 12.1, 1.4 Hz, 1H), 3.77–3.73 (m, 2H), 3.31 (ddd, *J* = 5.2, 4.0, 2.6 Hz, 1H), 3.02 (br, 1H), 2.91 (dd, *J* = 5.1, 3.9 Hz, 1H), 2.85 (dd, *J* = 5.0, 2.7 Hz, 1H). ^13^C NMR (75 MHz, CDCl_3_) *δ* 139.5, 132.3, 130.0, 129.3, 124.8, 122.4, 100.3, 79.6, 72.3, 64.3, 50.9, 46.0. MS (ESI) 301.0 (M + 1).

*4,5-Anhydro-1,3-O-*(*p-bromobenzylidene*)*-d-arabinitol* (**14m**). **14m** can be obtained from **13m** with a yield of 85%. Colorless oil. ${\left[\alpha \right]}_{D}^{25}$ = −42.4 (c = 0.50, MeOH). ^1^H NMR (300 MHz, chloroform-*d*) *δ* 7.56 (d, *J* = 8.5 Hz, 2H), 7.43 (d, *J* = 8.5 Hz, 2H), 5.56 (s, 1H), 4.28 (dd, *J* = 12.0, 1.9 Hz, 1H), 4.10 (dd, *J* = 12.0, 1.4 Hz, 1H), 3.81–3.78 (m, 2H), 3.35 (dt, *J* = 4.7, 2.3 Hz, 1H), 3.11 (br, 1H), 2.97–2.88 (m, 2H). ^13^C NMR (75 MHz, CDCl_3_) *δ* 136.5, 131.5, 127.9, 123.4, 100.6, 79.6, 72.3, 64.3, 50.9, 46.0. MS (ESI) 301.0 (M + 1).

*4,5-Anhydro-1,3-O-*(*o-fluorophenylmethylene*)*-d-arabinitol* (**14n**). **14n** can be obtained from **13n** with a yield of 91%. Pale-yellow oils. ${\left[\alpha \right]}_{D}^{25}$ = −33.1 (c = 0.50, MeOH). ^1^H NMR (300 MHz, chloroform-*d*) *δ* 7.62 (td, *J* = 7.5, 1.9 Hz, 1H), 7.38–7.33 (m, 1H), 7.16 (td, *J* = 7.5, 1.3 Hz, 1H), 7.09–7.03 (m, 1H), 5.83 (s, 1H), 4.24 (dd, *J* = 12.1, 2.0 Hz, 1H), 4.08 (dd, *J* = 12.1, 1.3 Hz, 1H), 3.79–3.74 (m, 2H), 3.32 (ddd, *J* = 5.2, 4.0, 2.6 Hz, 1H), 2.96–2.89 (m, 2H), 2.83 (dd, *J* = 5.2, 2.6 Hz, 1H). ^13^C NMR (75 MHz, CDCl_3_) *δ* 131.2 (d, *J*_C-F_ = 8.5 Hz), 127.8 (d, *J*_C-F_ = 3.6 Hz), 124.3 (d, *J*_C-F_ = 3.6 Hz), 115.7 (d, *J*_C-F_ = 21.1 Hz), 97.0 (d, *J*_C-F_ = 4.2 Hz), 80.1, 72.6, 64.5, 50.8, 46.0. MS (ESI) 241.1 (M + 1).

*4,5-Anhydro-1,3-O-*(*o-iodobenzylidene*)*-d-arabinitol* (**14o**). **14o** can be obtained from **13o** with a yield of 90%. Colorless oil. ${\left[\alpha \right]}_{D}^{25}$ = –29.6 (c = 0.50, MeOH). ^1^H NMR (300 MHz, chloroform-*d*) *δ* 7.85 (dd, *J* = 7.9, 1.2 Hz, 1H), 7.65 (dd, *J* = 7.8, 1.7 Hz, 1H), 7.40 (td, *J* = 7.6, 1.2 Hz, 1H), 7.08 (td, *J* = 7.6, 1.8 Hz, 1H), 5.60 (s, 1H), 4.26 (dd, *J* = 12.1, 1.9 Hz, 1H), 4.11 (dd, *J* = 12.1, 1.4 Hz, 1H), 3.80 (s, 1H), 3.76 (dd, *J* = 5.5, 1.2 Hz, 1H), 3.35 (ddd, *J* = 5.4, 3.9, 2.6 Hz, 1H), 3.11 (d, *J* = 10.4 Hz, 1H), 2.92 (dd, *J* = 5.1, 4.0 Hz, 1H), 2.88 (dd, *J* = 5.1, 2.6 Hz, 1H). ^13^C NMR (75 MHz, CDCl_3_) *δ* 139.6, 138.9, 131.1, 128.4, 127.8, 104.6, 97.0, 80.3, 72.5, 64.3, 50.6, 46.0. MS (ESI) 349.0 (M + 1).

*4,5-Anhydro-1,3-O-*(*o-cyanobenzylidene*)*-d-arabinitol* (**14p**). **14p** can be obtained from **13p** with a yield of 91%. Colorless oil. ${\left[\alpha \right]}_{D}^{25}$ = –49.1 (c = 0.50, MeOH). ^1^H NMR (300 MHz, chloroform-*d*) *δ* 7.71 (d, *J* = 7.4 Hz, 1H), 7.64–7.57 (m, 1H), 7.51–7.46 (m, 1H), 5.72 (s, 1H), 4.29 (dd, *J* = 12.1, 2.0 Hz, 1H), 4.10 (d, *J* = 12.1 Hz, 1H), 3.88 (d, *J* = 4.7 Hz, 1H), 3.81 (br, 1H), 3.42–3.28 (m, 2H), 2.91 (s, 2H). ^13^C NMR (75 MHz, CDCl_3_) *δ* 140.3, 133.6, 132.9, 129.9, 127.9, 118.5, 110.9, 100.4, 79.4, 72.6, 64.5, 62.5, 50.9, 45.6. MS (ESI) 248.1 (M + 1).

*4,5-Anhydro-1,3-O-*(*1-naphthalene*)*-d-arabinitol* (**14q**). **14q** can be obtained from **13q** with a yield of 85%. Colorless oil. ${\left[\alpha \right]}_{D}^{25}$ = –35.2 (c = 0.50, MeOH). ^1^H NMR (300 MHz, chloroform-*d*) *δ* 8.12 (dd, *J* = 8.4, 1.6 Hz, 1H), 7.89–7.88 (m, 1H), 7.81 (dd, *J* = 7.2, 1.3 Hz, 1H), 7.51 (dtd, *J* = 11.3, 8.1, 4.4 Hz, 3H), 6.10 (s, 1H), 4.30 (dd, *J* = 12.0, 2.0 Hz, 1H), 4.15 (dd, *J* = 12.1, 1.4 Hz, 1H), 3.87–3.79 (m, 2H), 3.38–3.36 (m, 1H), 3.09 (br, 1H), 2.91–2.87 (m, 2H). ^13^C NMR (75 MHz, CDCl_3_) *δ* 133.8, 132.6, 130.4, 130.0, 128.8, 126.5, 125.8, 125.1, 124.2, 123.8, 100.3, 79.9, 72.5, 64.5, 51.0, 46.0. MS (ESI) 273.1 (M + 1).

*4,5-Anhydro-1,3-O-*(*2-phenylbenzylidene*)*-d-arabinitol* (**14r**). **14r** can be obtained from **13r** with a yield of 87%. Colorless oil. ${\left[\alpha \right]}_{D}^{25}$ = –13.0 (c = 0.50, MeOH). ^1^H NMR (300 MHz, chloroform-*d*) *δ* 7.70–7.63 (m, 1H), 7.36–7.32 (m, 7H), 7.22–7.17 (m, 1H), 5.32 (s, 1H), 4.06 (t, *J* = 10.0 Hz, 1H), 3.78 (d, *J* = 11.8 Hz, 1H), 3.57 (d, *J* = 10.6 Hz, 1H), 3.41 (d, *J* = 5.5 Hz, 1H), 3.17 (t, *J* = 4.3 Hz, 1H), 2.78–2.71 (m, 2H), 2.63–2.58 (m, 1H). ^13^C NMR (75 MHz, CDCl_3_) *δ* 141.1, 140.4, 134.7, 130.3, 129.6, 129.3, 128.1, 127.8, 127.5, 126.5, 100.4, 79.9, 72.3, 64.4, 50.7, 45.9. MS (ESI) 299.1 (M + 1).

#### 3.2.4. General Procedure for Coupling Reactions

Coupling reaction to construct sulfonium salts: To a solution of **14a**–**r** (0.4 mmol) and **15** (0.36 mmol) in MeCN (4.0 mL) under an argon atmosphere was added trifluoroacetic acid (0.36 mmol) at −20 °C; after stirring for 10 min, the reaction temperature was warmed up to room temperature and stirred for another 8 h. The reaction mixture was concentrated under reduced pressure. The resulting residue was resolved in MeOH, and ion exchange reagent IRA (Cl^−^ form, 2.0 g) was added; the reaction mixture was stirred at room temperature for 4 h, filtered through celite, and washed with MeOH. The filtrate was concentrated under reduced pressure, and the resulting residue was purified by flash column chromatography (DCM/MeOH, 15:1) on silica gel to afford the pure products as pale-yellow oils with yields from 38 to 56%.

*1,4-Dideoxy-1,4-[*(*R*)*-*(*5-deoxy-1,3-O-*(*2,3-dichlorobenzylidene*)*-d-arabinitol-5-yl*) *episulfoniumylidene]-d-arabinitol chloride* (**19a**). **19a** was obtained from **14a** and **15** with a yield of 46%. Pale-yellow oils. ${\left[\alpha \right]}_{D}^{25}$ = –15.6 (c = 0.165, MeOH). ^1^H NMR (300 MHz, methanol-*d*_4_) *δ* 7.77 (dd, *J* = 7.8, 1.6 Hz, 1H), 7.55 (dd, *J* = 8.0, 1.7 Hz, 1H), 7.34 (t, *J* = 7.9 Hz, 1H), 5.93 (s, 1H), 4.64 (q, *J* = 2.6 Hz, 1H), 4.42–4.35 (m, 2H), 4.22–4.17 (m, 2H), 4.11–4.05 (m, 1H), 4.03–4.02 (m, 2H), 3.96 (s, 1H), 3.92 (d, *J* = 2.1 Hz, 1H), 3.86 (dd, *J* = 3.2, 1.7 Hz, 3H), 3.74 (dd, *J* = 13.2, 8.8 Hz, 1H). ^13^C NMR (75 MHz, methanol-*d*_4_) *δ* 138.8, 133.7, 132.0, 132.0, 128.8, 127.8, 99.6, 82.8, 79.5, 73.9, 73.8, 67.1, 63.1, 61.0, 52.0, 51.9. HRMS (ESI) calcd for C_17_H_23_Cl_2_O_7_S^+^ [M]^+^ 441.0536 found 441.0528.

*1,4-Dideoxy-1,4-[*(*R*)*-*(*5-deoxy-1,3-O-*(*2,4-dichlorobenzylidene*)*-d-arabinitol-5-yl*) *episulfoniumylidene]-d-arabinitol chloride* (**19b**). **19b** was obtained from **14b** and **15** with a yield of 46%. Pale-yellow oils. ${\left[\alpha \right]}_{D}^{25}$ = –15.2 (*c* = 0.165, MeOH). ^1^H NMR (300 MHz, methanol-*d*_4_) *δ* 7.80 (d, *J* = 8.3 Hz, 1H), 7.45 (d, *J* = 2.1 Hz, 1H), 7.36 (dd, *J* = 8.4, 2.1 Hz, 1H), 5.88 (s, 1H), 4.65 (q, *J* = 2.6 Hz, 1H), 4.41–4.35 (m, 2H), 4.25–4.17 (m, 2H), 4.11–4.00 (m, 3H), 3.98–3.96 (m, 1H), 3.92–3.91 (m, 1H), 3.88–3.83 (m, 3H), 3.74 (dd, *J* = 13.1, 9.3 Hz, 1H). ^13^C NMR (75 MHz, methanol-*d*_4_) *δ* 136.4, 135.4, 134.6, 130.7, 129.9, 128.3, 99.0, 82.6, 79.4, 73.8, 73.7, 67.1, 63.0, 60.9, 51.9, 51.8. HRMS (ESI) calcd for C_17_H_23_Cl_2_O_7_S^+^ [M]^+^ 441.0536 found 441.0537.

*1,4-Dideoxy-1,4-[*(*R*)*-*(*5-deoxy-1,3-O-*(*2,5-dichlorobenzylidene*)*-d-arabinitol-5-yl*) *episulfoniumylidene]-d-arabinitol chloride* (**19c**). **19c** was obtained from **14c** and **15** with a yield of 46%. Pale-yellow oils. ${\left[\alpha \right]}_{D}^{25}$ = –24.3 (*c* = 0.165, MeOH). ^1^H NMR (300 MHz, methanol-*d*_4_) *δ* 7.85–7.82 (m, 1H), 7.41–7.35 (m, 2H), 5.90 (s, 1H), 4.63 (d, *J* = 2.5 Hz, 1H), 4.38 (q, *J* = 5.4 Hz, 2H), 4.23–4.17 (m, 2H), 4.09–3.96 (m, 4H), 3.92 (q, *J* = 3.9, 3.2 Hz, 1H), 3.87–3.84 (m, 3H), 3.73 (dd, *J* = 13.1, 8.7 Hz, 1H). ^13^C NMR (75 MHz, methanol-*d*_4_) *δ* 138.2, 134.0, 132.2, 131.8, 131.4, 129.6, 98.9, 82.7, 79.5, 73.9, 73.8, 67.1, 63.1, 60.9, 52.0, 51.9. HRMS (ESI) calcd for C_17_H_23_Cl_2_O_7_S^+^ [M]^+^ 441.0536 found 441.0526.

*1,4-Dideoxy-1,4-[*(*R*)*-*(*5-deoxy-1,3-O-*(*2-chloro-3-fluorobenzylidene*)*-d-arabinitol-5-yl*) *episulfoniumylidene]-d-arabinitol chloride* (**19d**). **19d** was obtained from **14d** and **15** with a yield of 55%. Pale-yellow oils. ${\left[\alpha \right]}_{D}^{25}$ = –26.3 (c = 0.165, MeOH). ^1^H NMR (300 MHz, methanol-*d*_4_) *δ* 7.64 (dt, *J* = 7.8, 1.4 Hz, 1H), 7.36 (td, *J* = 8.1, 5.1 Hz, 1H), 7.25 (td, *J* = 8.7, 1.7 Hz, 1H), 5.92 (s, 1H), 4.64 (q, *J* = 2.6 Hz, 1H), 4.42–4.35 (m, 2H), 4.22 (dd, *J* = 3.8, 1.7 Hz, 2H), 4.11–4.00 (m, 4H), 3.96–3.92 (m, 1H), 3.88–3.86 (m, 3H), 3.74 (dd, *J* = 13.2, 8.8 Hz, 1H). ^13^C NMR (75 MHz, methanol-*d*_4_) *δ* 159.1 (d, *J* = 246.4 Hz), 138.7, 129.1 (d, *J* = 7.8 Hz), 124.7 (d, *J* = 3.3 Hz), 120.8 (d, *J* = 18.6 Hz), 117.9 (d, *J* = 21.3 Hz), 99.0 (d, *J* = 3.6 Hz), 82.7, 79.5, 73.9, 73.8, 67.1, 63.1, 61.0, 52.0, 51.9. HRMS (ESI) calcd for C_17_H_23_ClFO_7_S^+^ [M]^+^ 425.0832 found 425.0816.

*1,4-Dideoxy-1,4-[*(*R*)*-*(*5-deoxy-1,3-O-*(*2-chloro-5-fluorobenzylidene*)*-d-arabinitol-5-yl*) *episulfoniumylidene]-d-arabinitol chloride* (**19e**). **19e** was obtained from **14e** and **15** with a yield of 51%. Pale-yellow oils. ${\left[\alpha \right]}_{D}^{25}$ = –24.6 (c = 0.165, MeOH). ^1^H NMR (300 MHz, methanol-*d*_4_) *δ* 7.61 (dd, *J* = 9.4, 3.1 Hz, 1H), 7.48–7.42 (m, 1H), 7.16 (td, *J* = 8.3, 3.1 Hz, 1H), 5.92 (s, 1H), 4.69–4.61 (m, 1H), 4.45–4.39 (m, 2H), 4.24 (dd, *J* = 5.0, 1.8 Hz, 2H), 4.14–4.09 (m, 2H), 3.95 (d, *J* = 2.2 Hz, 1H), 3.90 (t, *J* = 3.3 Hz, 4H), 3.81–3.74 (m, 1H). ^13^C NMR (75 MHz, methanol-*d*_4_) *δ* 162.7 (d, *J* = 245.2 Hz), 138.6 (d, *J* = 7.7 Hz), 132.0 (d, *J* = 8.3 Hz), 128.7, 118.4 (d, *J* = 23.4 Hz), 116.4 (d, *J* = 25.2 Hz), 99.0, 82.7, 79.5, 73.9, 73.8, 67.1, 63.1, 60.9, 52.0, 51.8. HRMS (ESI) calcd for C_17_H_23_ClFO_7_S^+^ [M]^+^ 425.0832 found 425.0811.

*1,4-Dideoxy-1,4-[*(*R*)*-*(*5-deoxy-1,3-O-*(*3,4-dichlorobenzylidene*)*-d-arabinitol-5-yl*) *episulfoniumylidene]-d-arabinitol chloride* (**19f**). **19f** was obtained from **14f** and **15** with a yield of 43%. Pale-yellow oils. ${\left[\alpha \right]}_{D}^{25}$ = –26.4 (*c* = 0.165, MeOH). ^1^H NMR (300 MHz, methanol-*d*_4_) *δ* 7.74–7.72 (m, 1H), 7.54–7.44 (m, 2H), 5.65 (d, *J* = 4.6 Hz, 1H), 4.64 (q, 1H), 4.42–4.33 (m, 2H), 4.25–4.12 (m, 3H), 4.11–4.02 (m, 3H), 3.98–3.92 (m, 2H), 3.87–3.83 (m, 2H), 3.79–3.71 (m, 1H). ^13^C NMR (75 MHz, methanol-*d*_4_) *δ* 140.0, 131.3, 129.8, 127.5, 100.9, 82.6, 79.5, 73.8, 73.6, 67.3, 63.2, 61.0, 52.0, 51.9. HRMS (ESI) calcd for C_17_H_23_Cl_2_O_7_S^+^ [M]^+^ 441.0536 found 441.0519.

*1,4-Dideoxy-1,4-[*(*R*)*-*(*5-deoxy-1,3-O-*(*3,5-dichlorobenzylidene*)*-d-arabinitol-5-yl*) *episulfoniumylidene]-d-arabinitol chloride* (**19g**). **19g** was obtained from **14g** and **15** with a yield of 47%. Pale-yellow oils. ${\left[\alpha \right]}_{D}^{25}$ = –19.9 (*c* = 0.165, MeOH). ^1^H NMR (300 MHz, methanol-*d*_4_) *δ* 7.54–7.52 (m, 2H), 7.43 (t, *J* = 2.0 Hz, 1H), 5.64 (s, 1H), 4.64 (q, *J* = 2.6 Hz, 1H), 4.41–4.33 (m, 2H), 4.24–4.13 (m, 3H), 4.04 (dd, *J* = 8.2, 4.4 Hz, 2H), 3.98–3.88 (m, 3H), 3.87 (d, *J* = 2.6 Hz, 2H), 3.76 (dd, *J* = 13.2, 9.1 Hz, 1H). ^13^C NMR (75 MHz, methanol-*d*_4_) *δ* 143.0, 135.9, 129.8, 126.5, 126.3, 100.7, 82.6, 79.5, 73.7, 73.6, 67.3, 63.2, 61.0, 52.1, 51.9. HRMS (ESI) calcd for C_17_H_23_Cl_2_O_7_S^+^ [M]^+^ 441.0536 found 441.0522.

*1,4-Dideoxy-1,4-[*(*R*)*-*(*5-deoxy-1,3-O-*(*3-chloro-5-fluorobenzylidene*)*-d-arabinitol5-yl*) *episulfoniumylidene]-d-arabinitol chloride* (**19h**). **19h** was obtained from **14h** and **15** with a yield of 41%. Pale-yellow oils. ${\left[\alpha \right]}_{D}^{25}$ = –23.9 (*c* = 0.165, MeOH). ^1^H NMR (300 MHz, methanol-*d*_4_) *δ* 5.86 (d, *J* = 1.8 Hz, 1H), 5.75–5.66 (m, 1H), 5.62 (dh, *J* = 9.2, 2.1 Hz, 1H), 4.10 (d, *J* = 3.9 Hz, 1H), 3.11–3.04 (m, 1H), 2.87–2.70 (m, 2H), 2.63 (qd, *J* = 11.5, 10.9, 5.6 Hz, 3H), 2.55–2.47 (m, 2H), 2.44–2.36 (m, 2H), 2.33–2.24 (m, 3H), 2.24–2.18 (m, 1H). ^13^C NMR (75 MHz, methanol-*d*_4_) *δ* 162.6 (d, *J* = 248.1 Hz), 142.0 (d, *J* = 8.3 Hz), 134.5 (d, *J* = 10.5 Hz), 122.6 (d, *J* = 3.0 Hz), 116.0 (d, *J* = 25.3 Hz), 112.1 (d, *J* = 23.0 Hz), 99.4, 81.3, 78.2, 72.4, 72.3, 66.0, 61.9, 59.7. HRMS (ESI) calcd for C_17_H_23_ClFO_7_S^+^ [M]^+^ 425.0832 found 425.0866.

*1,4-Dideoxy-1,4-[*(*R*)*-*(*5-deoxy-1,3-O-*(*3,5-bis*(*trifluoromethyl*)*benzylidene*)*-d-arabinitol-5-yl*) *episulfoniumylidene]-d-arabinitol chloride* (**19i**). **19i** was obtained from **14i** and **15** with a yield of 38%. Pale-yellow oils. ${\left[\alpha \right]}_{D}^{25}$ = –26.1 (c = 0.165, MeOH). ^1^H NMR (300 MHz, methanol-*d*_4_) *δ* 8.19–8.16 (m, 2H), 7.96 (s, 1H), 5.85 (d, *J* = 1.9 Hz, 1H), 4.62 (dq, *J* = 14.0, 2.9 Hz, 1H), 4.45–4.36 (m, 2H), 4.29–4.23 (m, 2H), 4.12–4.04 (m, 3H), 3.96–3.95 (m, 2H), 3.92–3.87 (m, 3H), 3.77 (dd, *J* = 13.2, 8.9 Hz, 1H). ^13^C NMR (75 MHz, methanol-*d*_4_) *δ* 142.4, 130.2, 132.6 (q, *J* = 33.4 Hz), 126.6, 128.6 (d, *J* = 3.2 Hz), 123.6 (dq, *J* = 8.3, 4.8 Hz), 122.9, 100.7, 82.7, 79.5, 73.7, 67.3, 63.2, 61.0, 52.0, 51.9. HRMS (ESI) calcd for C_19_H_23_F_6_O_7_S^+^ [M]^+^ 509.1063 found 509.1085.

*1,4-Dideoxy-1,4-[*(*R*)*-*(*5-deoxy-1,3-O-*(*5-fluoro-2-nitrobenzylidene*)*-d-arabinitol-5-yl*) *episulfoniumylidene]-d-arabinitol chloride* (**19j**). **19*j*** was obtained from **14*j*** and **15** with a yield of 39%. Pale-yellow oils. ${\left[\alpha \right]}_{D}^{25}$ = –29.7 (c = 0.165, MeOH). ^1^H NMR (300 MHz, methanol-*d*_4_) *δ* 8.02–7.97 (m, 2H), 7.67 (dd, *J* = 9.5, 2.8 Hz, 1H), 7.37–7.31 (m, 1H), 6.18 (s, 1H), 4.68–4.58 (m, 1H), 4.43 (d, *J* = 2.5 Hz, 1H), 4.39–4.33 (m, 1H),4.25–4.15 (m, 3H),4.10 (d, *J* = 7.2 Hz, 3H),3.99–3.94 (m, 2H), 3.88–3.83 (m, 2H),3.65–3.58 (m, 1H). ^13^C NMR (75 MHz, methanol-*d*_4_) *δ* 165.7 (d, *J* = 253.6 Hz), 146.0 (d, *J* = 3.2 Hz), 136.3 (d, *J* = 9.2 Hz), 128.3 (d, *J* = 10.0 Hz), 117.7 (d, *J* = 23.6 Hz), 116.1 (d, *J* = 26.1 Hz), 97.5, 82.4, 79.6, 79.4, 73.9, 73.8, 66.8, 62.9, 60.9, 51.8, 51.5. HRMS (ESI) calcd for C_17_H_23_FNO_9_S^+^ [M]^+^ 436.1072 found 436.1035.

*1,4-Dideoxy-1,4-[*(*R*)*-*(*5-deoxy-1,3-O-*(*o-bromobenzylidene*)*-d-arabinitol-5-yl*) *episulfoniumylidene]-d-arabinitol chloride* (**19k**). **19k** was obtained from **14k** and **15** with a yield of 51%. Pale-yellow oils. ${\left[\alpha \right]}_{D}^{25}$ = –13.7 (*c* = 0.165, MeOH). ^1^H NMR (300 MHz, methanol-*d*_4_) *δ* 7.83 (dd, *J* = 7.8, 1.8 Hz, 1H), 7.60 (dd, *J* = 7.9, 1.3 Hz, 1H), 7.44–7.39 (m, 1H), 7.31 (qd, *J* = 7.2, 2.6 Hz, 1H), 5.87 (s, 1H), 4.67 (q, *J* = 2.4 Hz, 1H), 4.45–4.38 (m, 2H), 4.29–4.20 (m, 2H), 4.16–4.11 (m, 1H), 4.08–4.00 (m, 2H), 4.00–3.92 (m, 2H), 3.92–3.88 (m, 3H), 3.79 (dt, *J* = 13.1, 7.1 Hz, 1H). ^13^C NMR (75 MHz, methanol-*d*_4_) *δ* 138.0, 133.6, 131.8, 129.6, 128.5, 123.4, 101.7, 82.7, 79.4, 73.9, 73.7, 67.1, 63.1, 60.9, 51.9. HRMS (ESI) calcd for C_17_H_24_BrO_7_S^+^ [M]^+^ 451.0421 found 451.0411.

*1,4-Dideoxy-1,4-[*(*R*)*-*(*5-deoxy-1,3-O-*(*m-bromobenzylidene*)*-d-arabinitol-5-yl*) *episulfoniumylidene]-d-arabinitol chloride* (**19l**). **19l** was obtained from **14l** and **15** with a yield of 54%. Pale-yellow oils. ${\left[\alpha \right]}_{D}^{25}$ = –13.9 (*c* = 0.165, MeOH). ^1^H NMR (300 MHz, methanol-*d*_4_) *δ* 7.75 (t, *J* = 1.9 Hz, 1H), 7.50 (dd, *J* = 7.8, 1.8 Hz, 2H), 7.32–7.25 (m, 1H), 5.63 (s, 1H), 4.64–4.58 (m, 1H), 4.42–4.34 (m, 2H), 4.23–4.16 (m, 2H), 4.13–4.06 (m, 2H), 3.98–3.88 (m, 3H), 3.86 (d, *J* = 2.6 Hz, 2H), 3.83–3.82 (m, 1H), 3.77 (dd, *J* = 9.0, 4.1 Hz, 1H). ^13^C NMR (75 MHz, methanol-*d*_4_) *δ* 141.8, 133.0, 131.0, 130.6, 126.5, 123.0, 101.6, 82.6, 79.5, 73.8, 73.6, 67.3, 63.2, 61.5, 61.0, 52.0, 51.9. HRMS (ESI) calcd for C_17_H_24_BrO_7_S^+^ [M]^+^ 451.0421 found 451.0429.

*1,4-Dideoxy-1,4-[*(*R*)*-*(*5-deoxy-1,3-O-*(*p-bromobenzylidene*)*-d-arabinitol-5-yl*) *episulfoniumylidene]-d-arabinitol chloride* (**19m**). **19m** was obtained from **14m** and **15** with a yield of 56%. Pale-yellow oils. ${\left[\alpha \right]}_{D}^{25}$ = –18.7 (*c* = 0.165, MeOH). ^1^H NMR (300 MHz, methanol-*d*_4_) *δ* 7.54–7.45 (m, 4H), 5.63 (s, 1H), 4.63 (q, *J* = 2.6 Hz, 1H), 4.42–4.34 (m, 2H), 4.22–4.16 (m, 2H), 4.08–4.00 (m, 3H), 3.98–3.95 (m, 1H), 3.92–3.90 (m, 1H), 3.87–3.82 (m, 3H), 3.79–3.72 (m, 1H). ^13^C NMR (75 MHz, methanol-*d*_4_) *δ* 138.7, 132.2, 129.5, 123.8, 101.7, 82.5, 79.5, 73.8, 73.5, 67.3, 63.2, 61.5, 60.9, 52.0, 51.9. HRMS (ESI) calcd for C_17_H_24_BrO_7_S^+^ [M]^+^ 451.0421 found 451.0433.

*1,4-Dideoxy-1,4-{*(*R*)*-[5-deoxy-1,3-O-*(*o-fluorophenylmethylene*)*-d-arabinitol-5-yl] episulfoniumylidene}-d-arabinitol chloride* (**19n**). **19n** was obtained from **14n** and **15** with a yield of 45%. Pale-yellow oils. ${\left[\alpha \right]}_{D}^{25}$ = −31.6 (c = 0.542, MeOH). ^1^H NMR (300 MHz, methanol-*d*_4_) *δ* 7.65 (td, *J* = 7.5, 1.8 Hz, 1H), 7.28 (tdd, *J* = 7.5, 5.3, 1.8 Hz, 1H), 7.09 (td, *J* = 7.5, 1.1 Hz, 1H), 6.98 (ddd, *J* = 10.5, 8.3, 1.1 Hz, 1H), 5.82 (s, 1H), 4.53 (q, *J* = 2.6 Hz, 1H), 4.32–4.26 (m, 2H), 4.09 (qd, *J* = 12.3, 1.8 Hz, 2H), 3.93 (dd, *J* = 7.3, 5.5 Hz, 2H), 3.88 (dd, *J* = 8.0, 1.6 Hz, 1H), 3.85–3.79 (m, 1H), 3.75 (td, *J* = 8.0, 7.3, 2.5 Hz, 4H), 3.63 (dd, *J* = 13.2, 8.7 Hz, 1H). ^13^C NMR (75 MHz, methanol-*d*_4_) *δ* 163.1, 159.8, 132.0 (d, *J*_C-F_ = 8.4 Hz), 129.3 (d, *J* = 3.3 Hz), 129.3 (d, *J*_C-F_ = 3.3 Hz), 125.2 (d, *J*_C-F_ = 3.6 Hz), 116.1 (d, *J*_C-F_ = 21.5 Hz), 97.3 (d, *J*_C-F_ = 4.8 Hz), 82.7, 79.5, 74.0, 73.7, 67.2, 63.3, 61.0, 51.9. HRMS (ESI) calcd for C_17_H_24_FO_7_S^+^ [M]^+^ 391.1221, found 391.1214.

*1,4-Dideoxy-1,4-[*(*R*)*-*(*5-deoxy-1,3-O-*(*o-iodobenzylidene*)*-d-arabinitol-5-yl*) *episulfoniumylidene]-d-arabinitol chloride* (**19o**). **19o** was obtained from **14o** and **15** with a yield of 50%. Pale-yellow oils. ${\left[\alpha \right]}_{D}^{25}$ = –29.2 (c = 0.165, MeOH). ^1^H NMR (300 MHz, methanol-*d*_4_) *δ* 7.84 (dd, *J* = 8.1, 3.9 Hz, 1H), 7.73 (dd, *J* = 8.4, 4.1 Hz, 1H), 7.40 (td, *J* = 7.6, 3.7 Hz, 1H), 7.12–7.06 (m, 1H), 5.64 (d, *J* = 4.2 Hz, 1H), 4.64–4.56 (m, 1H), 4.42–4.34 (m, 2H), 4.27–4.19 (m, 2H), 4.08–3.97 (m, 3H), 3.93–3.85 (m, 3H), 3.79–3.64 (m, 1H). ^13^C NMR (75 MHz, methanol-*d*_4_) *δ* 139.6, 139.1, 130.6, 127.9, 104.2, 96.8, 81.4, 78.2, 72.6, 72.5, 65.8, 61.7, 59.7, 50.8, 50.6, 47.5, 47.2, 46.9. HRMS (ESI) calcd for C_17_H_24_IO_7_S^+^ [M]^+^ 499.0282 found 499.0295.

*1,4-Dideoxy-1,4-[*(*R*)*-*(*5-deoxy-1,3-O-*(*o-cyanobenzyliden*)*-d-arabinitol-5-yl*) *episulfoniumylidene]-d-arabinitol chloride* (**19p**). **19p** was obtained from **14p** and **15** with a yield of 48%. Pale-yellow oils. ${\left[\alpha \right]}_{D}^{25}$ = –32.5 (*c* = 0.165, MeOH). ^1^H NMR (300 MHz, methanol-*d*_4_) *δ* 7.86 (d, *J* = 7.6 Hz, 1H), 7.73 (d, *J* = 11.3 Hz, 2H), 7.56 (d, *J* = 7.6 Hz, 1H), 5.90 (s, 1H), 4.65 (s, 1H), 4.41 (s, 2H), 4.26 (d, *J* = 7.2 Hz, 2H), 4.08 (t, *J* = 12.8 Hz, 4H), 3.89 (s, 4H), 3.78 (d, *J* = 9.4 Hz, 1H). ^13^C NMR (75 MHz, methanol-*d*_4_) *δ* 142.0, 134.2, 130.8, 128.2, 118.9, 111.7, 100.0, 82.3, 79.6, 79.2, 73.7, 73.6, 67.0, 63.1, 60.9, 51.7, 51.6. HRMS (ESI) calcd for C_18_H_24_NO_7_S^+^ [M]^+^ 398.1268 found 398.1273.

*1,4-Dideoxy-1,4-[*(*R*)*-*(*5-deoxy-1,3-O-*(*1-naphthalene*)*-d-arabinitol-5-yl*) *episulfoniumylidene]-d-arabinitol chloride* (**19q**). **19q** was obtained from **14q** and **15** with a yield of 56%. Pale-yellow oils. ${\left[\alpha \right]}_{D}^{25}$ = –63.2 (*c* = 0.165, MeOH). ^1^H NMR (300 MHz, methanol-*d*_4_) *δ* 8.33 (d, *J* = 8.3 Hz, 1H), 7.91–7.85 (m, 2H), 7.80 (dt, *J* = 7.4, 1.9 Hz, 1H), 7.50 (ddt, *J* = 14.4, 6.6, 1.6 Hz, 3H), 6.20 (s, 1H), 4.58 (q, *J* = 2.5 Hz, 1H), 4.48–4.40 (m, 1H), 4.34–4.33 (m, 1H), 4.28 (t, *J* = 2.4 Hz, 2H), 4.16–4.06 (m, 4H), 3.98–3.96 (m, 1H), 3.93–3.87 (m, 2H), 3.85–3.83 (m, 1H), 3.79 (d, *J* = 2.4 Hz, 2H), 3.76–3.70 (m, 1H). ^13^C NMR (75 MHz, methanol-*d*_4_) *δ* 135.2, 134.6, 131.9, 130.6, 129.4, 127.2, 126.8, 126.0, 125.7, 125.6, 101.7, 82.8, 79.4, 73.8, 73.7, 67.4, 63.4, 60.9, 54.8, 52.0. HRMS (ESI) calcd for C_21_H_27_O_7_S^+^ [M]^+^ 423.1472 found 423.1477.

*1,4-Dideoxy-1,4-[*(*R*)*-*(*5-deoxy-1,3-O-*(*2-phenylbenzylidene*)*-d-arabinitol-5-yl*) *episulfoniumylidene]-d-arabinitol chloride* (**19r**). **19r** was obtained from **14r** and **15** with a yield of 41%. Pale-yellow oils. ${\left[\alpha \right]}_{D}^{25}$ = –37.6 (c = 0.165, MeOH). ^1^H NMR (300 MHz, methanol-*d*_4_) *δ* 7.90–7.87 (m, 1H), 7.42–7.39 (m, 7H), 7.27–7.24 (m, 1H), 5.51 (s, 1H), 4.62 (d, *J* = 2.7 Hz, 1H), 4.39–4.36 (m, 2H), 4.30–4.15 (m, 3H),4.11–4.05 (m, 3H), 3.94–3.90 (m, 3H), 3.71 (s, 2H), 3.60 (d, *J* = 5.1 Hz, 1H). ^13^C NMR (75 MHz, methanol-*d*_4_) *δ* 142.4, 141.8, 136.4, 130.7, 130.3, 130.0, 129.4, 129.1, 128.5, 128.2, 100.9, 82.9, 79.4, 73.8, 73.5, 67.2, 63.1, 61.0, 52.2, 51.9. HRMS (ESI) calcd for C_23_H_29_O_7_S^+^ [M]^+^ 449.1629 found 449.1663.

Coupling reaction to construct selenonium salts: To a solution of **14a**–**r** (0.4 mmol) and **16** (0.36 mmol) in MeCN (4.0 mL) under an argon atmosphere was added trifluoroacetic acid (0.36 mmol) at −20 °C; after stirring for 10 min, the reaction temperature was warmed up to room temperature and stirred for another 8 h. The reaction mixture was concentrated under reduced pressure. The resulting residue was resolved in MeOH, and ion exchange reagent IRA (Cl^−^ form, 2.0 g) was added; the reaction mixture was stirred at room temperature for 4 h, filtered through celite, and washed with MeOH. The filtrate was concentrated under reduced pressure, and the resulting residue was purified by flash column chromatography (DCM/MeOH, 15:1) on silica gel to afford the pure products as colorless solid with yields from 39 to 53%.

(*1S,2R,3S,4S*)*-1-*((*2S*)*-2-*((*4S,5R*)*-2-*(*2,3-Dichlorophenyl*)*-5-hydroxy-1,3-dioxan-4-yl*)*-2-hydroxyethyl*)*-3,4-dihydroxy-2-*(*hydroxymethyl*)*tetrahydro-1H-selenophen-1-ium chloride* (**20a**). **20a** was obtained from **14a** and **16** with a yield of 43%. Colorless solid. ${\left[\alpha \right]}_{D}^{25}$ = –24.7 (c = 0.165, MeOH). ^1^H NMR (300 MHz, methanol-*d*_4)_ *δ* 7.79–7.75 (m, 1H), 7.56 (dd, *J* = 8.1, 1.6 Hz, 1H), 7.34 (t, *J* = 7.9 Hz, 1H), 5.94 (s, 1H), 4.74 (q, *J* = 2.5 Hz, 1H), 4.47–4.43 (m, 1H), 4.36 (m, *J* = 8.1, 3.8 Hz, 1H), 4.21 (dd, *J* = 3.0, 1.6 Hz, 2H), 4.19–4.15 (m, 1H), 4.01–3.95 (m, 2H), 3.95–3.85 (m, 2H), 3.85–3.77 (m, 2H), 3.76 (d, *J* = 2.5 Hz, 2H). ^13^C NMR (75 MHz, methanol-*d*_4_) *δ* 138.9, 133.8, 132.1, 128.8, 127.8, 99.7, 83.5, 80.4, 80.3, 74.2, 73.8, 67.1, 63.4, 61.0, 49.8. HRMS (ESI) calcd for C_17_H_23_Cl_2_O_7_Se^+^ [M]^+^ 488.9981 found 488.9987.

(*1S,2R,3S,4S*)*-1-*((*2S*)*-2-*((*4S,5R*)*-2-*(*2,4-Dichlorophenyl*)*-5-hydroxy-1,3-dioxan-4-yl*)*-2-hydroxyethyl*)*-3,4-dihydroxy-2-*(*hydroxymethyl*)*tetrahydro-1H-selenophen-1-ium chloride* (**20b**). **20b** was obtained from **14b** and **16** with a yield of 43%. Colorless solid. ${\left[\alpha \right]}_{D}^{25}$ = –23.8 (c = 0.165, MeOH). ^1^H NMR (300 MHz, methanol-*d*_4_) *δ* 7.80 (dd, *J* = 8.4, 3.9 Hz, 1H), 7.47 (d, *J* = 2.1 Hz, 1H), 7.37 (dd, *J* = 8.5, 2.2 Hz, 1H), 5.89 (s, 1H), 4.79–4.60 (m, 1H), 4.46 (dd, *J* = 2.9, 1.3 Hz, 1H), 4.35 (m, *J* = 8.0, 3.8 Hz, 1H), 4.21–4.13 (m, 3H), 4.11–4.06 (m, 1H), 4.03–3.93 (m, 3H), 3.91–3.87 (m, 1H), 3.82 (d, *J* = 8.4 Hz, 2H), 3.75 (d, *J* = 2.5 Hz, 1H). ^13^C NMR (75 MHz, methanol-*d*_4_) *δ* 136.6, 135.5, 134.7, 130.7, 130.0, 128.3, 99.1, 83.4, 80.4, 80.3, 74.2, 73.8, 67.1, 63.3, 61.0, 49.7,49.5. HRMS (ESI) calcd for C_17_H_23_Cl_2_O_7_Se^+^ [M]^+^ 488.9981 found 488.9979.

(*1S,2R,3S,4S*)*-1-*((*2S*)*-2-*((*4S,5R*)*-2-*(*2,5-Dichlorophenyl*)*-5-hydroxy-1,3-dioxan-4-yl*)*-2-hydroxyethyl*)*-3,4-dihydroxy-2-*(*hydroxymethyl*)*tetrahydro-1H-selenophen-1-ium chloride* (**20c**). **20c** was obtained from **14c** and **16** with a yield of 43%. Colorless solid. ${\left[\alpha \right]}_{D}^{25}$ = –33.6 (c = 0.165, MeOH). ^1^H NMR (300 MHz, methanol-*d*_4_) *δ* 7.85 (s, 1H), 7.41–7.35 (m, 2H), 5.91 (s, 1H), 4.74 (s, 1H), 4.47 (d, *J* = 2.8 Hz, 1H), 4.36 (td, *J* = 8.2, 3.8 Hz, 1H), 4.20 (dd, *J* = 5.0, 2.1 Hz, 2H), 4.10 (q, *J* = 7.2 Hz, 2H), 4.06–3.88 (m, *J* = 6.9, 6.2 Hz, 4H), 3.84–3.83 (m, 1H), 3.78 (dd, *J* = 10.3, 3.0 Hz, 2H). ^13^C NMR (75 MHz, methanol-*d*_4_) *δ* 138.2, 134.0, 132.2, 131.8, 131.5, 129.6, 99.0, 83.4, 80.4, 80.3, 74.1, 73.8, 67.0, 63.3, 61.0, 49.8, 49.3. HRMS (ESI) calcd for C_17_H_23_Cl_2_O_7_Se^+^ [M]^+^ 488.9981 found 488.9984.

(*1S,2R,3S,4S*)*-1-*((*2S*)*-2-*((*4S,5R*)*-2-*(*2-Chloro-3-fluorophenyl*)*-5-hydroxy-1,3-dioxan-4-yl*)*-2-hydroxyethyl*)*-3,4-dihydroxy-2-*(*hydroxymethyl*)*tetrahydro-1H-selenophen-1-ium chloride* (**20d**). **20d** was obtained from **14d** and **16** with a yield of 52%. Colorless solid. ${\left[\alpha \right]}_{D}^{25}$ = –36.2 (c = 0.165, MeOH). ^1^H NMR (300 MHz, methanol-*d*_4_) *δ* 7.64 (d, *J* = 7.7 Hz, 1H), 7.37 (m, *J* = 7.9, 5.3 Hz, 1H), 7.29–7.23 (m, 1H), 4.74 (s, 1H), 4.47 (d, *J* = 2.9 Hz, 1H), 4.36 (dt, *J* = 8.2, 4.1 Hz, 1H), 4.22–4.17 (m, 3H), 4.06–3.90 (m, 4H), 3.83 (d, *J* = 8.5 Hz, 2H), 3.76 (d, *J* = 2.6 Hz, 2H). ^13^C NMR (75 MHz, methanol-*d*_4_) *δ* 160.8, 157.5, 138.8, 129.2, 129.1, 124.7, 124.6, 120.7, 118.1, 117.8, 99.1, 99.1, 83.5, 80.4, 80.2, 74.2, 73.8, 67.1, 63.4, 61.0, 49.7,49.6. HRMS (ESI) calcd for C_17_H_23_ClFO_7_Se^+^ [M]^+^ 473.0276 found 473.0298.

(*1S,2R,3S,4S*)*-1-*((*2S*)*-2-*((*4S,5R*)*-2-*(*2-Chloro-5-fluorophenyl*)*-5-hydroxy-1,3-dioxan-4-yl*)*-2-hydroxyethyl*)*-3,4-dihydroxy-2-*(*hydroxymethyl*)*tetrahydro-1H-selenophen-1-ium chloride* (**20e**). **20e** was obtained from **14e** and **16** with a yield of 45%. Colorless solid. ${\left[\alpha \right]}_{D}^{25}$ = –33.9 (c = 0.165, MeOH). ^1^H NMR (300 MHz, methanol-*d*_4_) *δ* 7.58 (dd, *J* = 9.4, 3.2 Hz, 1H), 7.41 (dd, *J* = 8.8, 4.9 Hz, 1H), 7.13 (m, *J* = 8.3, 3.1 Hz, 1H), 5.90 (s, 1H), 4.74 (s, 1H), 4.46 (d, *J* = 2.7 Hz, 1H), 4.39–4.33 (m, 1H), 4.33–4.13 (m, 3H), 4.10 (q, *J* = 7.2 Hz, 1H), 4.03–3.91 (m, 3H), 3.91–3.81 (m, 2H), 3.78 (dd, *J* = 12.6, 3.1 Hz, 2H). ^13^C NMR (75 MHz, methanol-*d*_4_) *δ* 132.0, 131.9, 118.6, 118.3, 116.6, 116.3, 99.0, 83.4, 80.4, 80.3, 74.2, 73.8, 67.0, 63.3, 61.5, 61.0, 49.8. HRMS (ESI) calcd for C_17_H_23_ClFO_7_Se^+^ [M]^+^ 473.0276 found 473.0277.

(*1S,2R,3S,4S*)*-1-*((*2S*)*-2-*((*4S,5R*)*-2-*(*3,4-Dichlorophenyl*)*-5-hydroxy-1,3-dioxan-4-yl*)*-2-hydroxyethyl*)*-3,4-dihydroxy-2-*(*hydroxymethyl*)*tetrahydro-1H-selenophen-1-ium chloride* (**20f**). **20f** was obtained from **14f** and **16** with a yield of 52%. Colorless solid. ${\left[\alpha \right]}_{D}^{25}$ = –34.7 (c = 0.165, MeOH). ^1^H NMR (300 MHz, methanol-*d*_4_) *δ* 7.75 (d, *J* = 2.1 Hz, 1H), 7.54–7.45 (m, 2H), 5.65 (s, 1H), 4.74 (d, *J* = 2.8 Hz, 1H), 4.47 (d, *J* = 3.1 Hz, 1H), 4.35 (m, *J* = 7.7, 3.9 Hz, 1H), 4.19 (m, *J* = 6.9, 5.8, 3.1 Hz, 3H), 4.13–4.04 (m, 1H), 3.97 (m, *J* = 11.5, 6.1, 3.7 Hz, 3H), 3.89–3.78 (m, 2H), 3.78–3.72 (m, 1H). ^13^C NMR (75 MHz, methanol-*d*_4_) *δ* 140.0, 133.1, 131.4, 129.8, 127.5, 100.9, 83.1, 80.4, 80.2, 74.0, 73.6, 67.3, 63.4, 61.0, 49.0,48.7. HRMS (ESI) calcd for C_17_H_23_Cl_2_O_7_Se^+^ [M]^+^ 488.9981 found 488.9986.

(*1S,2R,3S,4S*)*-1-*((*2S*)*-2-*((*4S,5R*)*-2-*(*3,5-Dichlorophenyl*)*-5-hydroxy-1,3–dioxan-4-yl*)*-2-hydroxyethyl*)*-3,4-dihydroxy-2-*(*hydroxymethyl*)*tetrahydro-1H-selenophen-1-ium Chloride* (**20g**). **20g** was obtained from **14g** and **16** with a yield of 46%. Colorless solid. ${\left[\alpha \right]}_{D}^{25}$ = –29.8 (*c* = 0.165, MeOH). ^1^H NMR (300 MHz, methanol-*d*_4_) *δ* 7.54 (t, *J* = 2.3 Hz, 2H), 7.43 (t, *J* = 1.9 Hz, 1H), 5.65 (d, *J* = 3.7 Hz, 1H), 4.75 (p, *J* = 2.9 Hz, 1H), 4.47 (m, *J* = 4.6, 2.8, 1.5 Hz, 1H), 4.35 (m, *J* = 7.9, 3.8 Hz, 1H), 4.20 (m, *J* = 13.1, 6.2, 4.4 Hz, 3H), 4.13–4.06 (m, 1H), 4.01–3.92 (m, 3H), 3.89–3.81 (m, 2H), 3.78–3.73 (m, 2H). ^13^C NMR (75 MHz, methanol-*d*_4_) *δ* 143.0, 135.9, 129.8, 126.4, 126.4, 100.7, 83.2, 80.4, 80.2, 73.9, 73.6, 67.2, 63.4, 61.1, 49.7,49.6. HRMS (ESI) calcd for C_17_H_23_Cl_2_O_7_Se^+^ [M]^+^ 488.9981 found 488.9984.

(*1S,2R,3S,4S*)*-1-*((*2S*)*-2-*((*4S,5R*)*-2-*(*3-Chloro-5-fluorophenyl*)*-5-hydroxy-1,3-dioxan-4-yl*)*-2-hydroxyethyl*)*-3,4-dihydroxy-2-*(*hydroxymethyl*)*tetrahydro-1H-selenophen-1-ium chloride* (**20h**)**. 20h** was obtained from **14h** and **16** with a yield of 43%. Colorless solid. ${\left[\alpha \right]}_{D}^{25}$ = –32.5 (*c* = 0.165, MeOH). ^1^H NMR (300 MHz, methanol-*d*_4_) *δ* 7.43 (s, 1H), 7.28 (d, *J* = 9.6 Hz, 1H), 7.20 (s, 1H), 5.65 (s, 1H), 4.74 (s, 1H), 4.47 (dd, *J* = 4.6, 2.2 Hz, 1H), 4.36 (td, *J* = 7.8, 7.1, 3.0 Hz, 1H), 4.18 (dt, *J* = 6.9, 2.6 Hz, 3H), 4.02–3.92 (m, 4H), 3.86–3.81 (m, 2H), 3.78–3.72 (m, 2H). ^13^C NMR (75 MHz, methanol-*d*_4_) *δ* 165.5, 143.4, 143.3, 136.0, 135.8, 123.9, 123.8, 117.5, 117.2, 113.5, 113.2, 100.7, 83.2, 82.6, 81.4, 80.4, 80.2, 74.0, 73.6, 67.3, 66.6, 63.4, 61.1, 49.7. HRMS (ESI) calcd for C_17_H_23_ClFO_7_Se^+^ [M]^+^ 473.0276 found 473.0297.

*(1S,2R,3S,4S)-1-((2S)-2-((4S,5R)-2-(3,5-Bis(trifluoromethyl)phenyl)-5-hydroxy-1,3-dioxan-4-yl)-2-hydroxyethyl)-3,4-dihydroxy-2-(hydroxymethyl)tetrahydro-1H-selenophen-1-ium chloride* (**20i**). **20i** was obtained from **14i** and **16** with a yield of 40%. Colorless solid. ${\left[\alpha \right]}_{D}^{25}$ = –42.3 (c = 0.165, MeOH). ^1^H NMR (300 MHz, methanol-*d*_4_) *δ* 7.72 (d, *J* = 6.6 Hz, 2H), 7.52 (s, 1H), 4.29 (s, 1H), 4.02 (d, *J* = 3.0 Hz, 1H), 3.92 (q, *J* = 5.8, 4.1 Hz, 1H), 3.89–3.78 (m, 2H), 3.74–3.71 (m, 1H), 3.65–3.48 (m, 3H), 3.42 (d, *J* = 5.2 Hz, 2H), 3.36–3.28 (m, 2H).^13^C NMR (75 MHz, methanol-*d*_4_) *δ* 142.4, 142.4, 132.8, 132.4, 128.5, 126.6, 123.0, 100.7, 83.3, 82.7, 80.4, 80.2, 73.9, 73.7, 67.2, 63.4, 61.1, 54.8, 49.7,49.6. HRMS (ESI) calcd for C_19_H_23_F_6_O_7_Se^+^ [M]^+^ 557.0508 found 557.0525.

(*1S,2R,3S,4S*)*-1-*((*2S*)*-2-*((*4S,5R*)*-2-*(*5-Fluoro-2-nitrophenyl*)*-5-hydroxy-1,3-dioxan-4-yl*)*-2-hydroxyethyl*)*-3,4-dihydroxy-2-*(*hydroxymethyl*)*tetrahydro-1H-selenophen-1-ium chloride* (**20j**)**. 20j** was obtained from **14j** and **16** with a yield of 39%. Colorless solid. ${\left[\alpha \right]}_{D}^{25}$ = –43.2 (c = 0.165, MeOH). ^1^H NMR (300 MHz, methanol-*d*_4_) *δ* 7.99 (dd, *J* = 8.9, 4.9 Hz, 1H), 7.67 (dd, *J* = 9.6, 2.9 Hz, 1H), 7.34 (td, *J* = 9.3, 8.5, 2.7 Hz, 1H), 6.20 (d, *J* = 7.4 Hz, 1H), 4.78 (s, 1H), 4.51 (s, 1H), 4.33 (m, *J* = 7.8, 5.1 Hz, 1H), 4.28–4.18 (m, 3H), 4.10 (q, *J* = 7.2 Hz, 1H), 4.01 (m, *J* = 14.1, 13.0, 7.9 Hz, 2H), 3.94–3.85 (m, 3H), 3.77 (dd, *J* = 11.9, 3.2 Hz, 1H), 3.68 (dd, *J* = 11.9, 7.7 Hz, 1H). ^13^C NMR (75 MHz, methanol-*d*_4_) *δ* 167.4, 164.1, 136.4, 136.3, 128.4, 128.3, 117.9, 117.5, 116.2, 115.9, 97.6, 83.2, 80.5, 80.2, 74.4, 73.9, 66.6, 63.1, 61.5, 61.1,49.5,49.3. HRMS (ESI) calcd for C_17_H_23_FNO_9_Se^+^ [M]^+^ 484.0517 found 484.0536.

(*1S,2R,3S,4S*)*-1-*((*2S*)*-2-*((*4S,5R*)*-2-*(*2-Bromophenyl*)*-5-hydroxy-1,3-dioxan-4-yl*)*-2-hydroxyethyl*)*-3,4-dihydroxy-2-*(*hydroxymethyl*)*tetrahydro-1H-selenophen-1-ium chloride* (**20k**). **20k** was obtained from **14k** and **16** with a yield of 53%. Colorless solid. ${\left[\alpha \right]}_{D}^{25}$ = –24.3 (*c* = 0.165, MeOH). ^1^H NMR (300 MHz, methanol-*d*_4_) *δ* 7.82–7.79 (m, 1H), 7.57 (dd, *J* = 7.9, 1.3 Hz, 1H), 7.41–7.36 (m, 1H), 7.27 (td, *J* = 7.7, 1.8 Hz, 1H), 5.85 (s, 1H), 4.73 (q, *J* = 2.8 Hz, 1H), 4.46 (pd, *J* = 3.7, 2.9, 1.5 Hz, 1H), 4.36 (td, *J* = 8.0, 3.8 Hz, 1H), 4.25–4.15 (m, 3H), 4.10 (q, *J* = 7.1 Hz, 1H), 3.98–3.94 (m, 2H), 3.91 (dd, *J* = 4.3, 3.1 Hz, 1H), 3.87–3.82 (m, 2H), 3.74 (d, *J* = 2.5 Hz, 2H). ^13^C NMR (75 MHz, methanol-*d*_4_) *δ* 138.0, 133.6, 131.8, 129.6, 128.6, 123.4, 101.7, 83.4, 80.4, 80.2, 74.2, 73.8, 67.1, 63.4, 61.5, 61.0, 48.7. HRMS (ESI) calcd for C_17_H_24_BrO_7_Se^+^ [M]^+^ 498.9865 found 498.9867.

**(***1S,2R,3S,4S*)*-1-*((*2S*)*-2-*((*4S,5R*)*-2-*(*3-Bromophenyl*)*-5-hydroxy-1,3-dioxan-4-yl*)*-2-hydroxyethyl*)*-3,4-dihydroxy-2-*(*hydroxymethyl*)*tetrahydro-1H-selenophen-1-ium chloride* (**20l**). **20l** was obtained from **14l** and **16** with a yield of 51%. Colorless solid. ${\left[\alpha \right]}_{D}^{25}$ = –24.7 (c = 0.165, MeOH). ^1^H NMR (300 MHz, methanol-*d*_4_) *δ* 7.76 (s, 1H), 7.51 (d, *J* = 7.8 Hz, 2H), 7.32–7.27 (m, 1H), 5.65 (d, *J* = 3.4 Hz, 1H), 4.73 (s, 1H), 4.47 (m, 1H), 4.36 (td, *J* = 7.6, 3.9 Hz, 1H), 4.29–4.16 (m, 3H), 4.14–4.00 (m, 2H), 3.99–3.95 (m, 2H), 3.87–3.80 (m, 2H), 3.77–3.69 (m, 2H). ^13^C NMR (75 MHz, methanol-*d*4) *δ* 141.8, 133.0, 131.0, 130.6, 126.5, 123.1, 101.6, 83.2, 80.4, 80.2, 74.0, 73.6, 67.3, 63.5, 61.1, 49.7,49.6. HRMS (ESI) calcd for C_17_H_24_BrO_7_Se^+^ [M]^+^ 498.9865 found 498.9869.

(*1S,2R,3S,4S)-1-((2S)-2-((4S,5R)-2-(4-Bromophenyl)-5-hydroxy-1,3-dioxan-4-yl)-2-hydroxyethyl)-3,4-dihydroxy-2-(hydroxymethyl)tetrahydro-1H-selenophen-1-ium chloride* (**20m**). **20m** was obtained from **14m** and **16** with a yield of 53%. Colorless solid. ${\left[\alpha \right]}_{D}^{25}$ = –29.2 (c = 0.165, MeOH). ^1^H NMR (300 MHz, methanol-*d*_4_) *δ* 7.50 (q, *J* = 8.6 Hz, 4H), 5.64 (s, 1H), 4.73 (d, *J* = 2.6 Hz, 1H), 4.60 (br, 1H), 4.46 (d, *J* = 2.5 Hz, 1H), 4.36 (td, *J* = 7.4, 4.2 Hz, 1H), 4.18–4.12 (m, 3H), 3.98 (d, *J* = 5.2 Hz, 1H), 3.96–3.90 (m, 2H), 3.87–3.79 (m, 2H), 3.77–3.68 (m, 2H). ^13^C NMR (75 MHz, methanol-*d*_4_) *δ* 138.7, 132.3, 129.5, 123.9, 101.8, 83.2, 80.4, 80.2, 74.1, 73.6, 67.4, 63.5, 61.1, 49.7. HRMS (ESI) calcd for C_17_H_24_BrO_7_Se^+^ [M]^+^ 498.9865 found 498.9868.

*(1S,2R,3S,4S)-1-((2S)-2-((4S,5R)-2-(2-Fluorophenyl)-5-hydroxy-1,3-dioxan-4-yl)-2-hydroxyethyl)-3,4-dihydroxy-2-(hydroxymethyl)tetrahydro-1H-selenophen-1-ium chloride* (**20n**). **20n** was obtained from **14n** and **16** with a yield of 47%. Colorless solid. ${\left[\alpha \right]}_{D}^{25}$ = –43.7 (c = 0.165, MeOH). ^1^H NMR (300 MHz, methanol-*d*_4_) *δ* 7.76 (td, *J* = 7.4, 2.0 Hz, 1H), 7.43–7.36 (m, 1H), 7.20 (t, *J* = 7.5 Hz, 1H), 7.09 (dd, *J* = 10.5, 8.3 Hz, 1H), 5.93 (s, 1H), 4.72 (d, *J* = 2.7 Hz, 1H), 4.45 (d, *J* = 2.9 Hz, 1H), 4.37 (m, *J* = 7.8, 4.0 Hz, 1H), 4.23–4.15 (m, 3H), 4.12–4.00 (m, 1H), 3.98–3.92 (m, 2H), 3.89 (t, *J* = 3.8 Hz, 1H), 3.86–3.78 (m, 2H), 3.75 (d, *J* = 2.6 Hz, 1H). ^13^C NMR (75 MHz, methanol-*d*_4_) *δ* 132.1, 132.0, 129.3, 129.3, 126.6, 126.4, 125.2, 125.2, 116.3, 116.0, 97.3, 97.2, 83.3, 80.3, 80.2, 74.1, 73.7, 67.1, 63.4, 61.0, 49.7, 49.5. HRMS (ESI) calcd for C_17_H_24_FO_7_Se^+^ [M]^+^ 439.0666 found 439.0672.

(*1S,2R,3S,4S*)*-3,4-Dihydroxy-1-*((*2S*)*-2-hydroxy-2-*((*4S,5R*)*-5-hydroxy-2-*(*2-iodophenyl*)*-1,3-dioxan-4-yl*)*ethyl*)*-2-*(*hydroxymethyl*)*tetrahydro-1H-selenophen-1-ium chloride* (**20o**). **20o** was obtained from **14o** and **16** with a yield of 52%. Colorless solid. ${\left[\alpha \right]}_{D}^{25}$ = –19.7 (c = 0.165, MeOH). ${\left[\alpha \right]}_{D}^{25}$ = (c = 0.165, MeOH). ^1^H NMR (300 MHz, methanol-*d*_4_) *δ* 7.87–7.84 (m, 1H), 7.73 (m, *J* = 7.7, 2.1 Hz, 1H), 7.43–7.38 (m, 1H), 7.11 (m, *J* = 7.7, 5.1, 2.5 Hz, 1H), 5.66 (s, 1H), 4.73 (s, 1H), 4.47–4.46 (m, 1H), 4.36 (q, *J* = 4.1 Hz, 1H), 4.23–4.19 (m, 2H), 4.11–4.06 (m, 1H), 3.99–3.94 (m, 3H), 3.90 (d, *J* = 8.1 Hz, 1H), 3.86–3.82 (m, 2H), 3.75 (d, *J* = 2.5 Hz, 2H). ^13^C NMR (75 MHz, methanol-*d*_4_) *δ* 140.9, 140.4, 131.9, 129.2, 105.6, 98.0, 83.4, 80.4, 80.2, 74.3, 73.8, 67.1, 63.3, 61.5, 61.0, 50.0. HRMS (ESI) calcd for C_17_H_24_IO_7_Se^+^ [M]^+^ 546.9726 found 546.9736.

(*1S,2R,3S,4S)-1-((2S)-2-((4S,5R)-2-(2-Cyanophenyl)-5-hydroxy-1,3-dioxan-4-yl)-2-hydroxyethyl)-3,4-dihydroxy-2-(hydroxymethyl)tetrahydro-1H-selenophen-1-ium chloride* (**20p**). **20p** was obtained from **14p** and **16** with a yield of 50%. Colorless solid. ${\left[\alpha \right]}_{D}^{25}$ = –35.2 (c = 0.165, MeOH). ^1^H NMR (300 MHz, methanol-*d*_4_) *δ* 7.86 (d, *J* = 7.8 Hz, 1H), 7.79–7.68 (m, 2H), 7.56 (m, *J* = 8.4, 4.2 Hz, 1H), 5.91 (s, 1H), 4.74 (s, 1H), 4.48 (s, 1H), 4.35 (d, *J* = 20.7 Hz, 2H), 4.27–4.24 (m, 2H), 4.03 (m, *J* = 8.3, 5.8, 3.8 Hz, 2H), 3.97 (d, *J* = 2.8 Hz, 1H), 3.94–3.85 (m, 3H), 3.79–3.70 (m, 2H). ^13^C NMR (75 MHz, methanol-*d*_4_) *δ* 142.2, 134.2, 130.8, 128.2, 118.9, 111.8, 100.1, 83.0, 80.5, 80.0, 74.1, 73.8, 66.9, 63.3, 61.0. HRMS (ESI) calcd for C_18_H_24_NO_7_Se^+^ [M]^+^ 446.0713 found 446.0726.

*(1S,2R,3S,4S)-3,4-dihydroxy-1-((2S)-2-hydroxy-2-((4S,5R)-5-hydroxy-2-(naphthalen-1-yl)-1,3-dioxan-4-yl)ethyl)-2-(hydroxymethyl)tetrahydro-1H-selenophen-1-ium chloride* (**20q**). **20q** was obtained from **14q** and **16** with a yield of 45%. Colorless solid. ${\left[\alpha \right]}_{D}^{25}$ = –80.3 (c = 0.165, MeOH). ^1^H NMR (300 MHz, methanol-*d*_4_) *δ* 8.34 (d, *J* = 8.2 Hz, 1H), 7.88 (d, *J* = 8.1 Hz, 2H), 7.80 (d, *J* = 7.1 Hz, 1H), 7.55–7.47 (m, 3H), 6.22 (d, *J* = 2.0 Hz, 1H), 4.65 (s, 1H), 4.41 (s, 2H), 4.29 (s, 2H), 4.13 (d, *J* = 8.1 Hz, 2H), 3.96–3.93 (m, 2H), 3.88 (dd, *J* = 10.3, 3.9 Hz, 3H), 3.72–3.62 (m, 1H), 3.55 (d, *J* = 11.9 Hz, 1H). ^13^C NMR (75 MHz, methanol-*d*_4_) *δ* 135.2, 131.9, 130.7, 129.5, 127.3, 126.8, 126.0, 125.7, 125.6, 101.8, 83.4, 80.5, 74.0, 73.7, 67.5, 63.8, 61.0. HRMS (ESI) calcd for C_21_H_27_O_7_Se^+^ [M]^+^ 471.0917 found 471.0930.

*(1S,2R,3S,4S)-1-((2S)-2-((4S,5R)-2-([1,1′-biphenyl]-2-yl)-5-hydroxy-1,3-dioxan-4-yl)-2-hydroxyethyl)-3,4-dihydroxy-2-(hydroxymethyl)tetrahydro-1H-selenophen-1-ium chloride* (**20r**). **20r** was obtained from **14r** and **16** with a yield of 49%. Colorless solid. ${\left[\alpha \right]}_{D}^{25}$ = –51.2 (c = 0.165, MeOH). ^1^H NMR (300 MHz, methanol-*d*_4_) *δ* 7.86 (dd, *J* = 6.7, 3.1 Hz, 1H), 7.36 (q, *J* = 7.8, 7.3 Hz, 7H), 7.23–7.20 (m, 1H), 5.46 (s, 1H), 4.67 (d, *J* = 15.3 Hz, 1H), 4.42 (s, 1H), 4.25 (s, 1H), 4.12–4.05 (m, 3H), 3.93–3.84 (m, 3H), 3.65 (d, *J* = 16.4 Hz, 4H), 3.59–3.54 (m, 1H). ^13^C NMR (75 MHz, methanol-*d*_4_) *δ* 142.4, 141.7, 136.4, 130.8, 130.4, 130.0, 129.4, 128.5, 128.4, 128.2, 101.0, 83.6, 80.4, 80.2, 74.0, 73.6, 67.2, 63.4, 61.0, 49.7. HRMS (ESI) calcd for C_23_H_29_O_7_Se^+^ [M]^+^ 497.1073 found 497.1091.

### 3.3. In Vitro α-Glucosidase Inhibition Assay

α-Glucosidase inhibitory activities of the target compounds **19a**–**r** and **20a**–**r** were tested for rat intestinal *α*-glucosidases such as maltase and sucrase in vitro and compared with those of clinically used anti-diabetic agent voglibose and acarbose. The testing method previously described was modified and used. Thus, rat small intestinal brush border membrane vesicles were first prepared, and its suspension in 0.1 M maleate buffer (pH = 6.0) was used as small intestinal *α*-glucosidases of maltase and sucrase. A test compound was dissolved in dimethylsulfoxide (DMSO), and the resulting solution was diluted with 0.1 M maleate buffer to prepare the test compound solution (concentration of DMSO: 10%). The substrate solution in maleate buffer (maltose, 74 μM, sucrose, 74 μM, 50 μL), a test compound solution (25 μL), and the enzyme solution (25 μL) were mixed and incubated at 37 °C for 30 min. After incubation, the solution was immediately heated by boiling water for 2 min to stop the reaction, and the solution was mixed with water (150 μL). Glucose concentration was determined by the glucose-oxidase method. Final concentration of DMSO in the test solution was 2.5% and no influence of DMSO was detected on the inhibitory activity. The IC_50_ value was determined graphically by a plot of percent inhibition versus log of the test compound.

### 3.4. Cytotoxicity Evaluation of Compounds (***20b***, ***20e*** and ***21b***) on Normal HELF Cell Lines

The cytotoxicity of the most potent inhibitors (**20a**, **20c** and **20m**) obtained from the present study was evaluated via the 3-[4,5-dimethylthiazol-2-yl]-2,5-diphenyl-tetrazolium bromide (MTT) assay on HELF, a human embryonic lung fibroblast cell line which was a kind gift from KeyGEN BioTECH. Briefly, the HELF-adherent cells were cultured in a 96-well plate overnight in a CO_2_ environment at 37 °C. Supernatant was removed, and 100 μL serially diluted compounds with complete medium DMEM supplemented with 10% (*v*/*v*) fetal bovine serum, penicillin (100 units/mL), and streptomycin (100 μg/mL) were added to each well. After the incubation, the culture medium was aspirated carefully, and 50 μL of MTT solution (2mg/mL PBS) was added to each well and further incubated for 4h. After this, MTT solution was aspirated, and cells were PBS-washed once, and 100 μL of DMSO was added to dissolve the blue insoluble MTT formazan produced by the action of mitochondrial dehydrogenase. The plate was agitated at room temperature for 15 min and then read at 570 nm by using a microplate reader. The percentage of viable cells was calculated as the relative ratio of optical densities.

### 3.5. Molecular Docking

The crystalline complex of the natural product neokotalanol with ntMGAM (pdbcode: 3L4U) was selected from the PDB database. The .pdb file for 3L4U was downloaded. Proteins (PDB ID:3L4U) were visualized and checked using PyMOL 3.0.3 software to ensure file integrity and docking suitability. Ligands were geometrically optimized by the MMFF94 force field of OpenBabel 2.4.1 software. The optimized molecular structures had the lowest energy and optimal conformation to ensure their reliability for subsequent molecular docking. Receptor proteins and ligand molecules were pre-processed using AutoDock Tools 1.5.6, and molecular docking parameters were set in the Grid module. The molecular docking was performed in semi-flexible docking mode with the docking accuracy parameter exhaustiveness = 25. The chosen docking algorithm was the Lamarckian Genetic Algorithm, and the docking calculations were completed by running AutoDock Vina 1.2.0 software. The docking results were visualized by Pymol.

### 3.6. Inhibition-Kinetic Studies

For kinetic analyses of maltase and sucrase by compounds (**20a**, **20c**, and **10**), the enzyme and test samples (1.0 mg/mL for maltase and 0.2 mg/mL for sucrase) were incubated with increasing concentrations of maltose (2.5, 5, 10, 20, 50, 100 μM) and sucrose (5, 10, 20, 50, 100, 1000 μM) at 37 °C for 30 min. The tube was immediately immersed in boiling water for 2 min to stop the reaction, then cooled with water. The glucose concentration was determined by the glucose-oxidase method. The results plotted according to Lineweaver–Burk revealed a fully competitive type of inhibition on each α-glucosidase. The Lineweaver–Burk equation follows as:

$$\frac{1}{V}=\frac{\mathrm{Km}}{\mathrm{Vmax}\times [S]}+\frac{1}{\mathrm{Vmax}}$$

$$\frac{1}{V}=\frac{\mathrm{Km}}{\mathrm{Vmax}\times [S]}+\frac{1}{\mathrm{Vmax}}$$

K_i_ values for each inhibitor were determined by the equation:

$$\mathrm{Ki}=\frac{[I]\times \mathrm{Km}}{\left[\mathrm{Kcat}-\mathrm{Km}\right]}$$

### 3.7. Hypoglycemic Effect in Normal ICR Mice

The animal experimental protocols in this study were approved by the Experimental Animal Center of Shanghai Institute of Materia Medica (Shanghai, China). Male ICR (Institute of Cancer Research) mice (29−33 g) after an overnight fast were used for acute maltose and sucrose loading tests. In the maltose loading test, maltose (2.0 mpk of body weight) and the inhibitors and voglibose were dissolved in distillation−distillation water and administered to the mice via a stomach tube. A control group was loaded with distillation−distillation water. In the sucrose loading test, sucrose (2.5 g/kg of body weight) and the inhibitor miglitol were dissolved in distillation−distillation water and administered to the mice via a stomach tube. A control group was loaded with distillation−distillation water. Blood samples for glucose measurements were obtained from the tail vein at 0, 15, 30, 60, and 120 min after maltose and sucrose loading. The blood glucose levels were measured by a portable kit, StatStrip Xpress (Nova Biomedical Co., Ltd., Waltham, MA, USA). The AUC over was calculated by the trapezoidal method.

## 4. Conclusions

Based on our previously reported *Salacia*-derived sulfonium salts featuring a 3′,5′-benzylidene core structure on side chain moiety, a more comprehensive structural modification was conducted in the present investigation. Multiple hydrophobic halogens were attached on the phenyl ring of the benzylidene structure, and an additional phenyl ring was incorporated as either naphthyl or biphenyl groups. Furthermore, applying the principle of “bioisosterism”, the sulfonium cation center was replaced by a selenonium cation. Most of the resulting sulfonium or selenonium salts presented strong inhibitory activity against both maltase and sucrase. Strong electron-withdrawing groups attached at the ortho-position on the phenyl ring, such as nitro or chloro, play a critical and fundamental role in potent α-glucosidase inhibitory activities. Compared to sulfonium salts, their selenonium analogs usually generally exhibited improved in vitro inhibitory potency against both maltase and sucrase. The most potent inhibitor, selenonium 20c (IC_50_ = 0.05 mM against sucrase and 0.14 mM against maltase), was selected for in vivo hypoglycemic evaluation. Although slightly less potent than compound **10**, **20c** (10.0 mg/kg) showed significant antihyperglycemic effects superior to those of acarbose (20.0 mg/kg) in the sucrose leading assay. At an equimolar dose, **20c** (1.0 mg/kg) demonstrated antihyperglycemic effects comparable to voglibose (1.0 mg/kg) in the maltose loading assay. Enzyme kinetic studies on **20c** showed fully competitive inhibition against α-glucosidases. Cytotoxicity evaluation of **20c** against multiple cell lines indicated low toxicity. Docking studies of **20c** with ntMGAM revealed binding to α-glucosidase via hydrogen bonds, π-alkyl interactions, alkyl interactions, and critical electrostatic interactions contributing to its potent inhibitory activity. This study provides strong evidence that replacing the sulfonium cation core with selenonium may enhance the α-glucosidase inhibitory activity of certain *Salacia*-derived inhibitors.

## Figures and Tables

**Table 1:** IC_50_ Values for synthetic sulfonium salts **19a**–**r**, acarbose, and voglibose against maltase and sucrase.

Entry	Compounds	Maltase	Sucrase
1	voglibose	1.45/1.5 ^[a]^	0.35/0.3 ^[a]^
2	acarbose	3.5/3.2 ^[a]^	2.2/2.5 ^[a]^
3	neoponkoranol (**7**)	7.3 ± 1.5/6.9 ^[a]^	2.8 ± 0.5/2.3 ^[a]^
4	**19a**	0.5 ± 0.08	1.6 ± 0.43
5	**19b**	2.0 ± 0.08	1.2 ± 0.23
6	**19c**	0.55 ± 0.05	0.8 ± 0.12
7	**19d**	1.0 ± 0.05	0.7 ± 0.13
8	**19e**	5.6 ± 0.29	6.6 ± 0.45
9	**19f**	5.0 ± 0.39	4.6 ± 0.09
10	**19g**	4.9 ± 0.25	5.5 ± 0.62
11	**19h**	0.85 ± 0.28	1.4 ± 0.33
12	**19i**	8.9 ± 0.53	10.0 ± 1.8
13	**19j**	0.75 ± 0.08	1.5 ± 0.21
14	**19k**	1.9 ± 0.08	1.1 ± 0.02
15	**19l**	9.7 ± 0.74	7.9 ± 1.6
16	**19m**	4.1 ± 0.08	3.5 ± 0.06
17	**19n**	0.42/0.4 [33]	0.55/0.84 [33]
18	**19o**	3.1 ± 0.66	3.3 ± 0.28
19	**19p**	0.88 ± 0.04	0.9 ± 0.05
20	**19q**	4.6 ± 0.37	5.8 ± 0.41
21	**19r**	10.5 ± 1.1	3.3 ± 0.22

^[a]^: Ref. [36], all data were obtained by triple testing and are expressed as mean ± SD (*n* = 3).

**Table 2:** IC_50_ values for synthetic selenonium salts **20a**–**r**, acarbose, and voglibose against maltase and sucrase.

Entry	Compound	Maltase	Sucrase
1	voglibose	1.45/1.5 ^[a]^	0.35/0.3 ^[a]^
2	acarbose	3.5/3.2 ^[a]^	2.2/2.5 ^[a]^
3	neoponkoranol (**7**)	7.3 ± 1.5/6.9 ^[a]^	2.8 ± 0.5/2.3 ^[a]^
4	**20a**	0.33 ± 0.15	0.19 ± 0.02
5	**20b**	0.26 ± 0.08	0.22 ± 0.06
6	**20c**	0.14 ± 0.05	0.05 ± 0.02
7	**20d**	0.62 ± 0.07	0.87 ± 0.23
8	**20e**	0.45 ± 0.08	0.41 ± 0.11
9	**20f**	0.47 ± 0.11	0.33 ± 0.04
10	**20g**	0.71 ± 0.21	0.22 ± 0.05
11	**20h**	3.67 ± 0.89	1.96 ± 0.25
12	**20i**	1.01 ± 0.32	0.41 ± 0.11
13	**20j**	0.33 ± 0.08	0.09 ± 0.03
14	**20k**	0.53 ± 0.22	0.12 ± 0.05
15	**20l**	0.69 ± 0.05	0.21 ± 0.02
16	**20m**	0.07 ± 0.03	0.20 ± 0.05
17	**20n**	3.75 ± 0.58	0.20 ± 0.06
18	**20o**	0.36 ± 0.07	0.30 ± 0.11
19	**20p**	0.82 ± 0.21	0.37 ± 0.05
20	**20q**	0.92 ± 0.23	0.68 ± 0.14
21	**20r**	2.00 ± 0.52	0.72 ± 0.17

^[a]^: Ref. [36], all data were obtained by triple testing and expressed as mean ± SD (*n* = 3).

**Table 3:** Ki values of compounds **20a**, **20c**, and **10** for small intestinal α-glucosidase.

	Ki (μg/mL)	
Compounds	Maltase (Km = 3.0 mM)	Sucrase (Km = 23.56 mM)
**20a**	0.94	0.49
**20c**	0.67	0.20
**10**	0.42	0.29
acarbose	1.98	0.67

**Table 4:** Declined rates of blood glucose levels and AUC caused by **20c** (10 mpk), **10**, neoponkoranol (10 mpi), and acarbose (20 mpk) in sucrose loading test.

Compounds	Declined Rate of Blood Glucose Levels (%)			
	15 min	30 min	60 min	AUC
acarbose-20.0 mpk	45.7%	48.4%	44.3%	40.5%
Neoponkoranol-10.0 mpk	42.8%	49.5%	43.5%	39.4%
**20c**-10.0 mpk	48.6%	52.8%	48.1%	46.4%
**10**-10.0 mpk	48.0%	54.6%	52.5%	46.6%

**Table 5:** Declined rates of blood glucose levels and AUC caused by **20c** (1 mpk), **10**, neoponkoranol (1 mpk), and acarbose (20 mpk) in sucrose loading test.

	Declined Rate of Blood Glucose Levels (%)			
Compounds	15 min	30 min	60 min	AUC
acarbose-20.0 mpk	45.7%	48.4%	44.3%	40.5%
neoponkoranol-1.0 mpk	33.5%	36.4%	27.1%	27.1%
**20c**-1.0 mpk	40.0%	40.3%	38.2%	34.00%
**10**-1.0 mpk	41.8%	43.00%	39.1%	35.2%

**Table 6:** Declined rates of blood glucose levels and AUC resulted by **20c** (1 mpk), **10**, neoponkoranol (1 mpk), and voglibose (1.0 mpk) in the maltose loading test.

Compounds	Declined Rate of Blood Glucose Levels (%)			
	15 min	30 min	60 min	AUC
voglibose-1.0 mpk	49.2%	53.7%	40.1%	39.8%
neoponkoranol-1.0 mpk	42.3%	33.9%	19.4%	21.5%
**20c**-1.0 mpk	45.8%	47.4%	43.5%	40.2%
**10**-1.0 mpk	52.8%	55.0%	47.2%	43.5%

## Data Availability

Data is contained within the article and Supplementary Materials.

## Supplementary Materials

The following supporting information can be downloaded at: https://www.mdpi.com/article/10.3390/molecules30132856/s1. The supporting information contains ^1^H and ^13^C NMR, curves of selected sulfonium and selenonium salts in enzyme assay and IC_50_ Values of **20a** and **20c** against different normal cell lines.
