# Supplementary material for: Design and Synthesis of Sulfonium and Selenonium Derivatives Bearing 3′,5′-O-Benzylidene Acetal Side Chain Structure as Potent α-Glucosidase Inhibitors
Source: Molecules. 2025 Jul 4;30(13):2856. doi: 10.3390/molecules30132856 (PMC12250965; doi:10.3390/molecules30132856)

Supporting Information

Design, synthesis of sulfonium and selenonium derivatives bearing 3',5'-*O* benzylidene acetal side chain structure as potent α-glucosidase inhibitors

Xiaosong He [1], Jiahao Yi [2], Jianchen Yang, Genzoh Tanabe[3], Osamu Muraoka[4], and Weijia Xie [1,*]

[1] Department of Medicinal Chemistry, China Pharmaceutical University, 24 Tong Jia Xiang, Nanjing 210009, PR China 1; e-mail@e-mail.com
[2] Department of Biomedical Engineering, The University of Texas at Austin, Austin, TX, 78712, USA 2; e-mail@e-mail.com
[3] School of Pharmacy, Kindai University, 3-4-1 Kowakae, Higashi-osaka, Osaka 577-8502, Japan 3; e-mail@e-mail.com
* Correspondence: weijiaxie@cpu.edu.cn;

....

Contents

1. General Information S2

2. Copies of ^{1}H NMR and ^{13}C NMR Spectra S3-S75

3. Curves of selected sulfonium and selenonium salts in enzyme assay…….S76-77

4. IC_{50} Values (μM) of 20a and 20c against different normal cell lines....…………………………………………………………………………….S78

1. General Information

Reagents and Solvents: All solvents were purified and dried according to standard methods. PE refers to petroleum ether (b.p. 60 - 90 ºC), EA refers to ethyl acetate, DCM refers to methylene dichloride and DMF refers to dimethyl formamide.

Chromatography: Flash column chromatography was carried out using commercially available 200-300 mesh under pressure and conducted by eluting with PE/EA, which are listed as volume/volume ratios.

Data Collection: Melting point (m.p.) was measured on a microscopic melting point apparatus. ^{1}H NMR spectra were collected on a BRUKER AV-300 (300 MHz) spectrometer using $CDCl_3$ or DMSO-d_6 or Methanol-d_4 as solvent. Chemical shifts of ^{1}H NMR were recorded in parts per million (ppm, δ) relative to tetramethylsilane (δ = 0.00 ppm) with the solvent resonance as an internal standard ($CDCl_3$，δ = 7.26 ppm, DMSO-d_6, δ = 2.50 ppm, Methanol-d_4, δ = 3.31 ppm). Data are reported as follows: chemical shift in ppm (δ), multiplicity (s = singlet, d = doublet, t = triplet, q = quartet, brs = broad singlet, m = multiplet), coupling constant (Hz), and integration. ^{13}C NMR spectra were collected on a BRUKER AV-300 (75 MHz) and BRUKER AVANCE500 (125 MHz) spectrometer using $CDCl_3$ or DMSO-d_6 or Methanol-d_4 as solvent. Chemical shifts of ^{13}C NMR were reported in ppm with the solvent as the internal standard ($CDCl_3$, δ = 77.0 ppm, DMSO-d_6, δ = 39.5 ppm, Methanol-d_4, δ = 47.1 ppm). Low-resolution mass spectra (MS) were measured on Finnigan MAT 95 spectrometer (Finnigan, Germany). High Resolution Mass measurement was performed on Agilent QTOF 6520 mass spectrometer with electron spray ionization (ESI) as the ion source. All compounds selected for *in vivo* hypoglycemic effect investigation are >95% pure by HPLC (SHIMADZU Labsolutions, UV detection at λ = 230 nm) analysis on the Agilent C18 column (4.6 × 150 mm^2, 5 μm) eluting at 1 mL/min of 90% methanol/10% water.

2. Copies of ^{1}H NMR and ^{13}C NMR Spectra

^{1}H-NMR spectrum of **12**

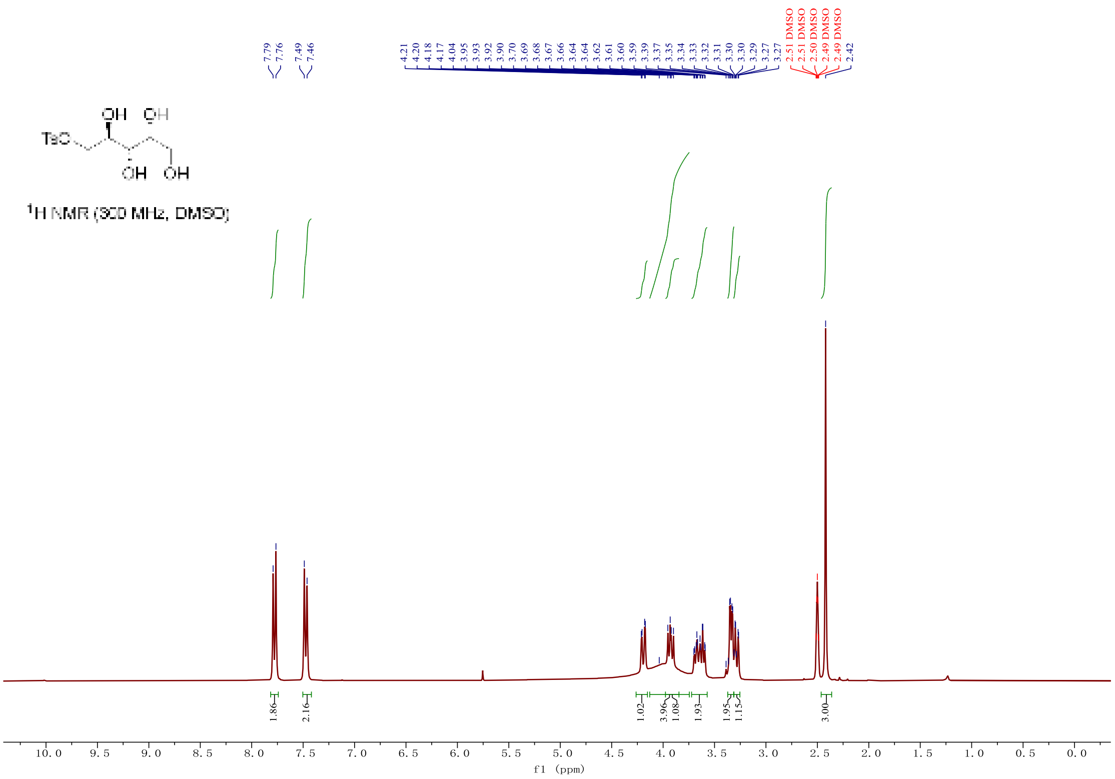

^{13}C-NMR spectrum of **12**

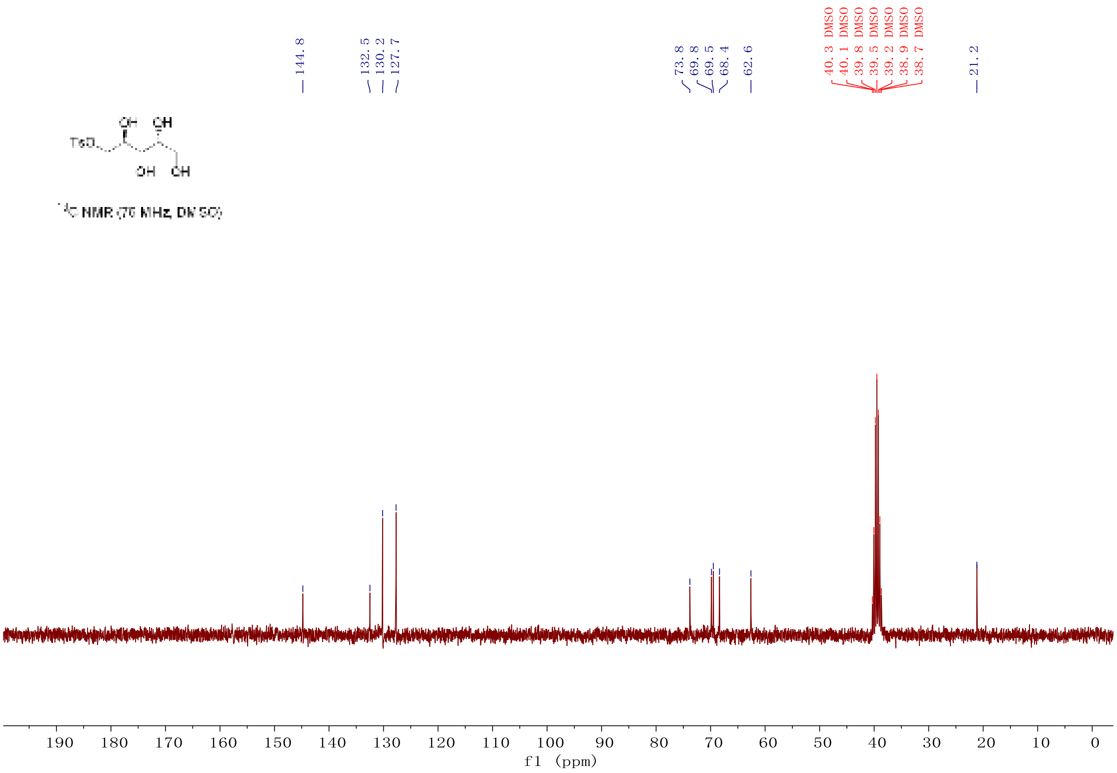

^{1}H NMR Spectrum of **13a** (300 MHz, $CDCl_3$)

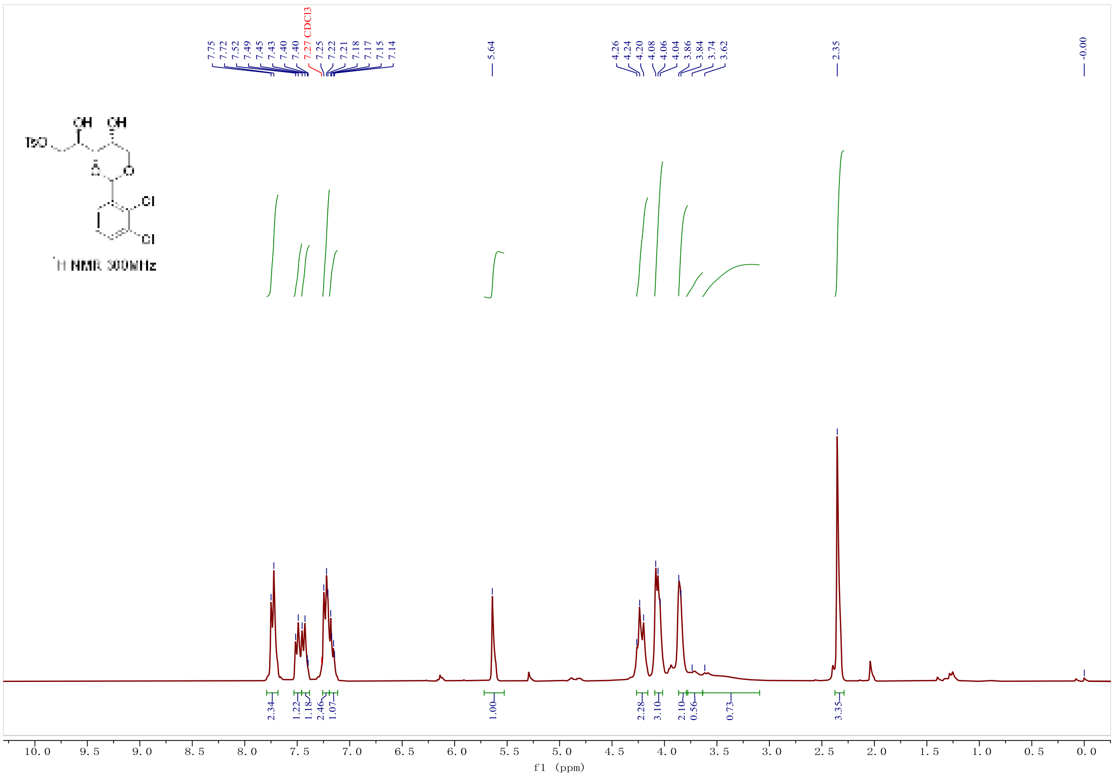

^{13}C NMR Spectrum of **13a** (75 MHz, $CDCl_3$)

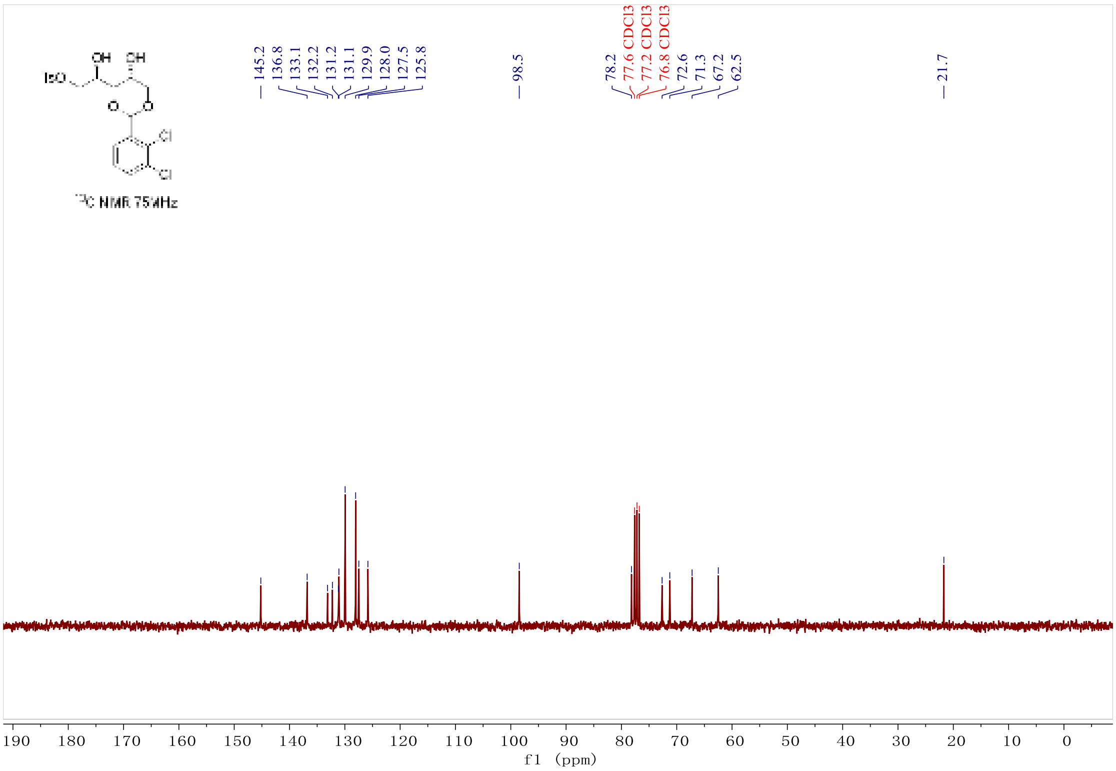

^{1}H NMR Spectrum of **13b** (300 MHz, $CDCl_3$)

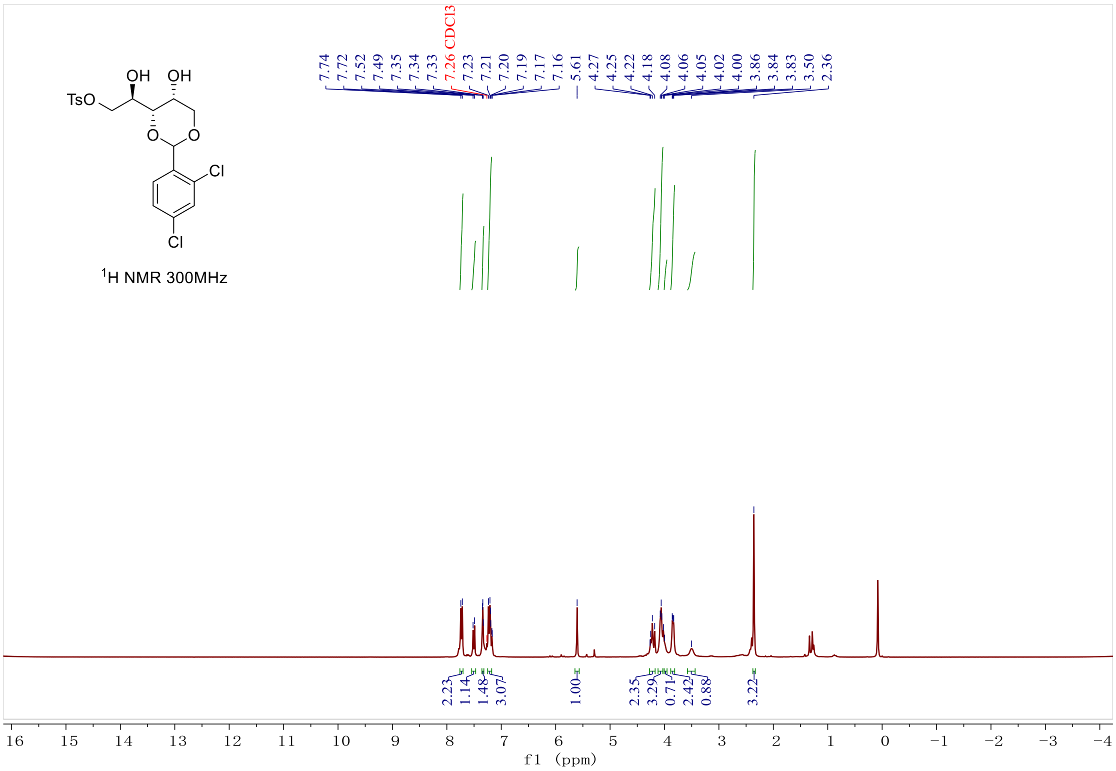

^{13}C NMR Spectrum of **13b** (75 MHz, $CDCl_3$)

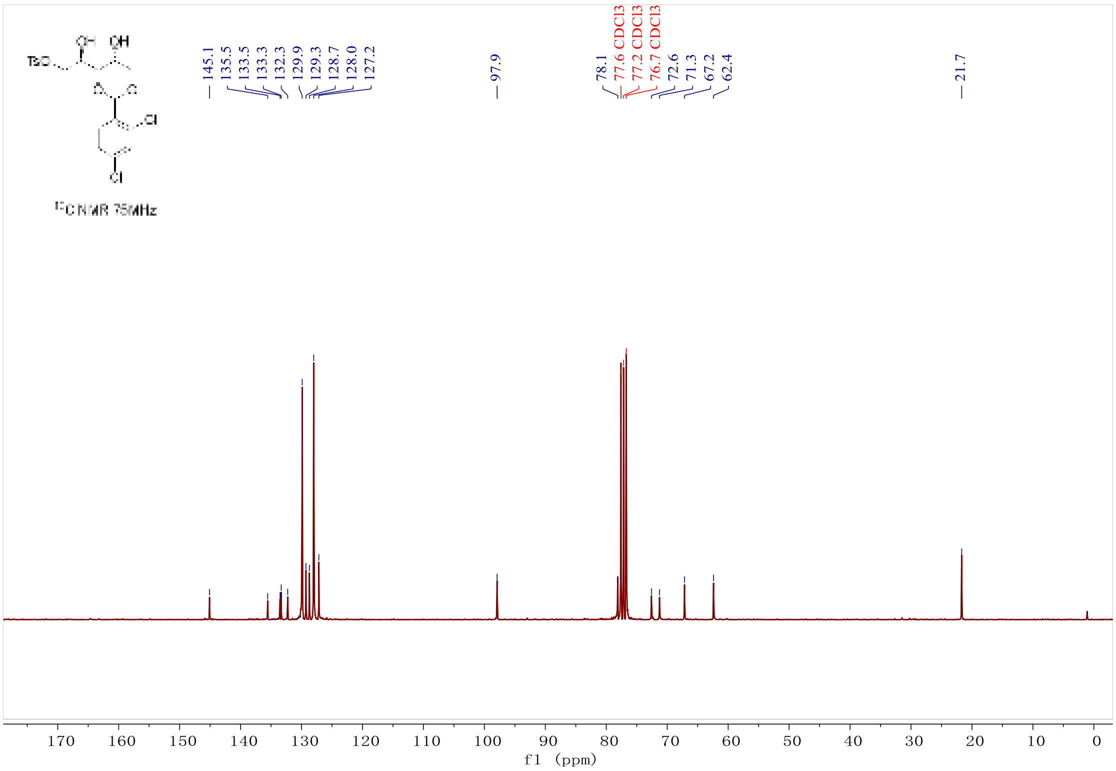

^{1}H NMR Spectrum of **13c** (300 MHz, $CDCl_3$)

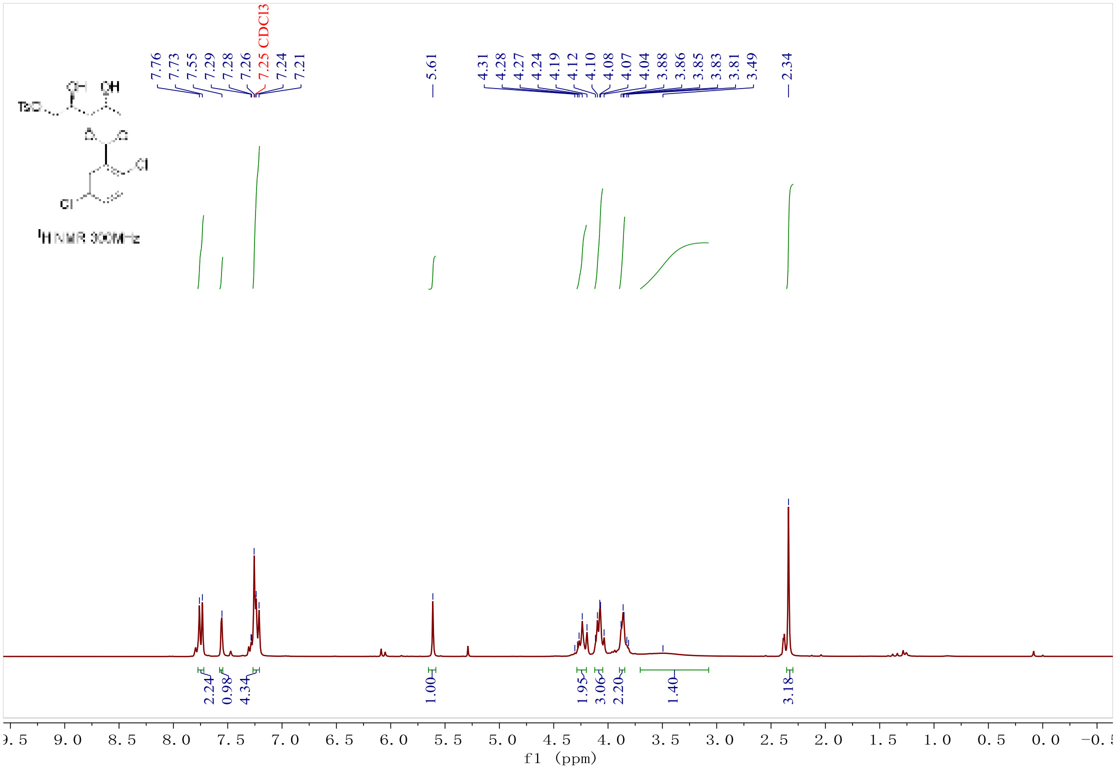

^{13}C NMR Spectrum of **13c** (75 MHz, $CDCl_3$)

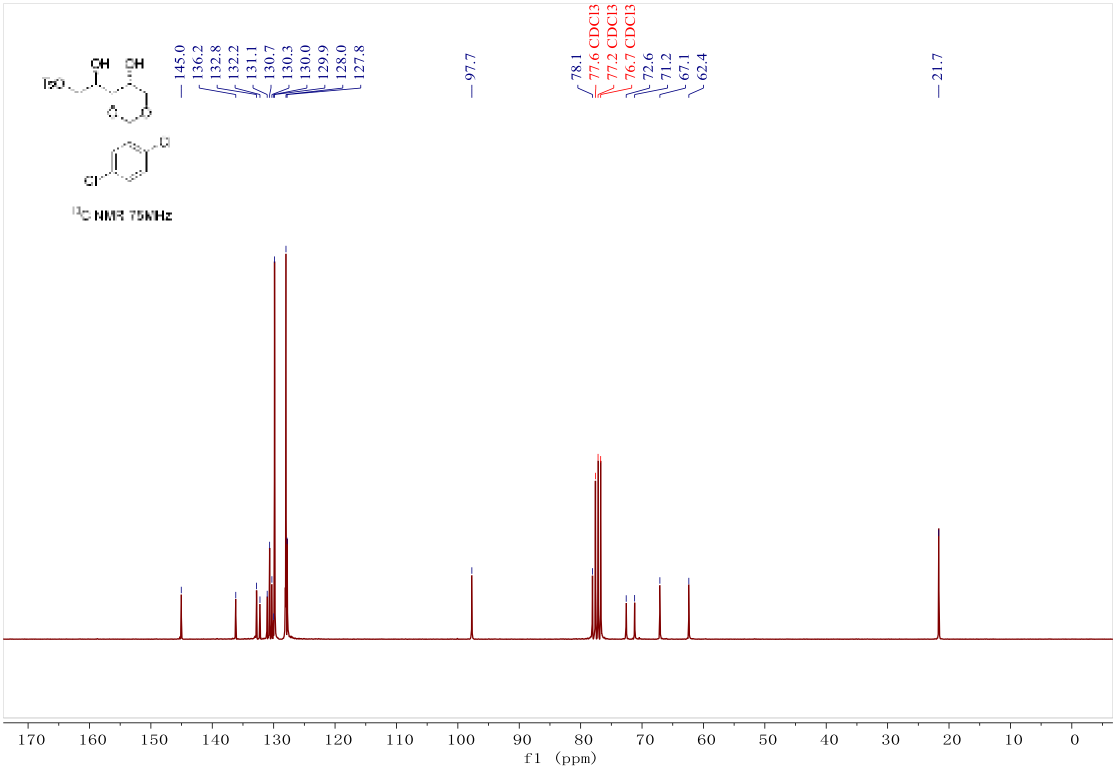

^{1}H NMR Spectrum of **13d** (300 MHz, $CDCl_3$)

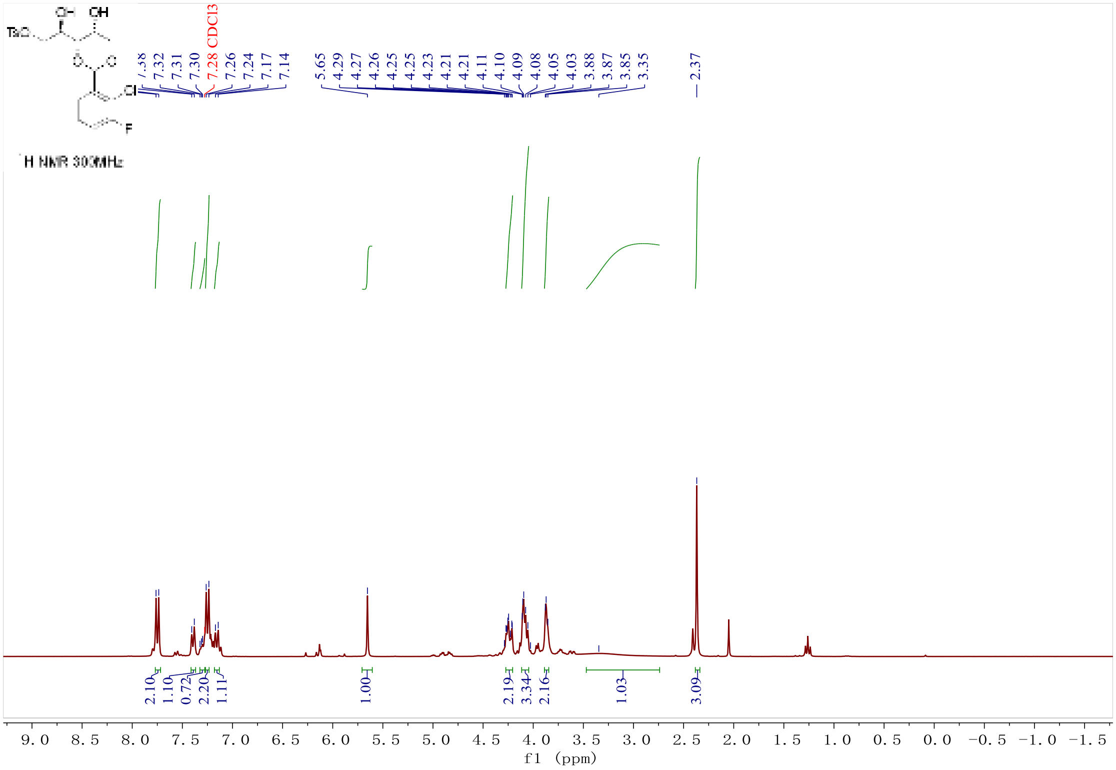

^{13}C NMR Spectrum of **13d** (75 MHz, $CDCl_3$)

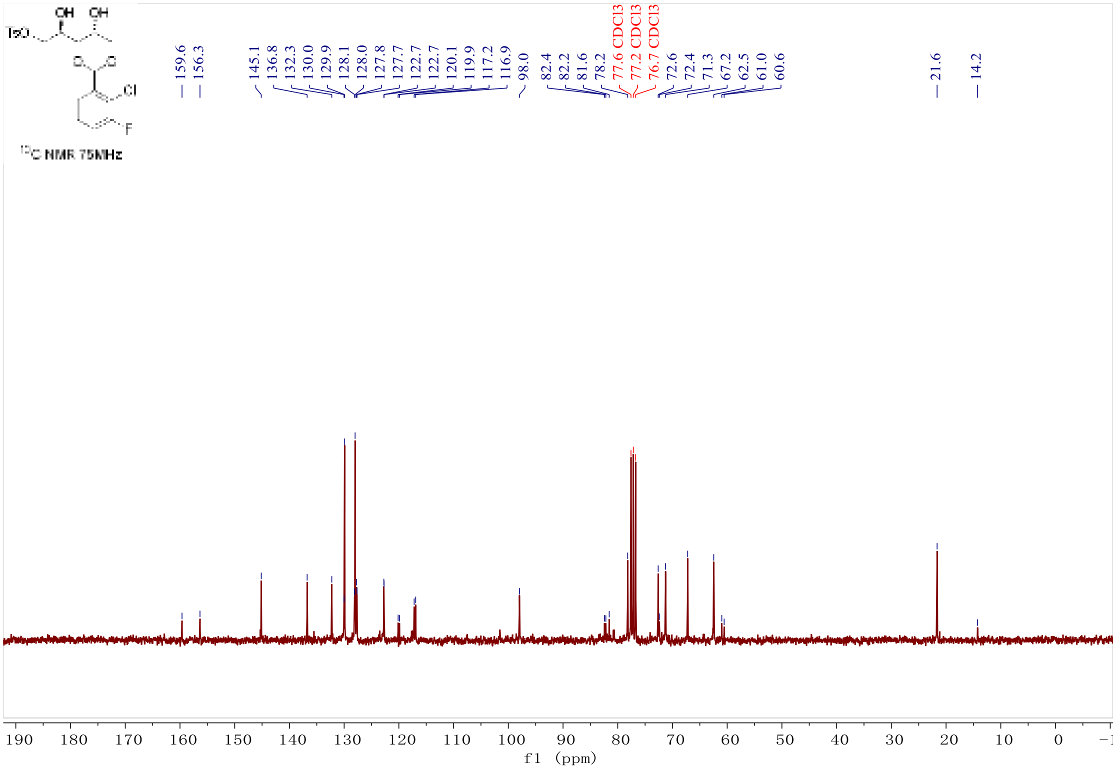

^{1}H NMR Spectrum of **13e** (300 MHz, $CDCl_3$)

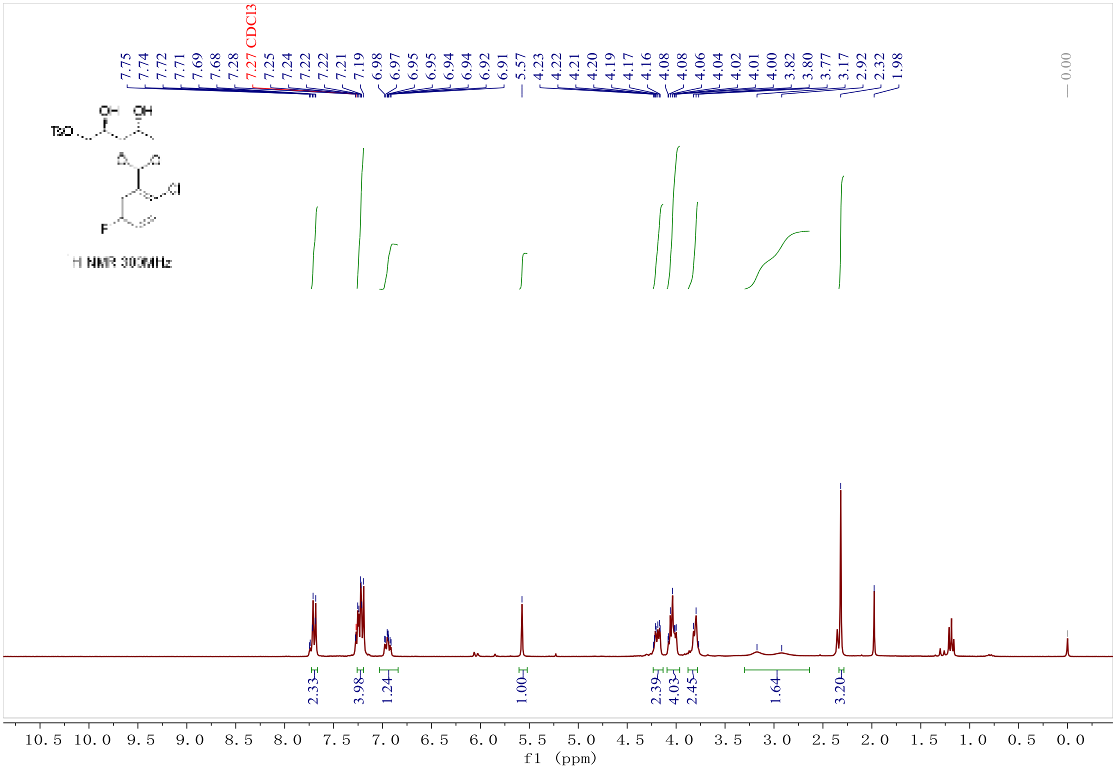

^{13}C NMR Spectrum of **13e** (75 MHz, $CDCl_3$)

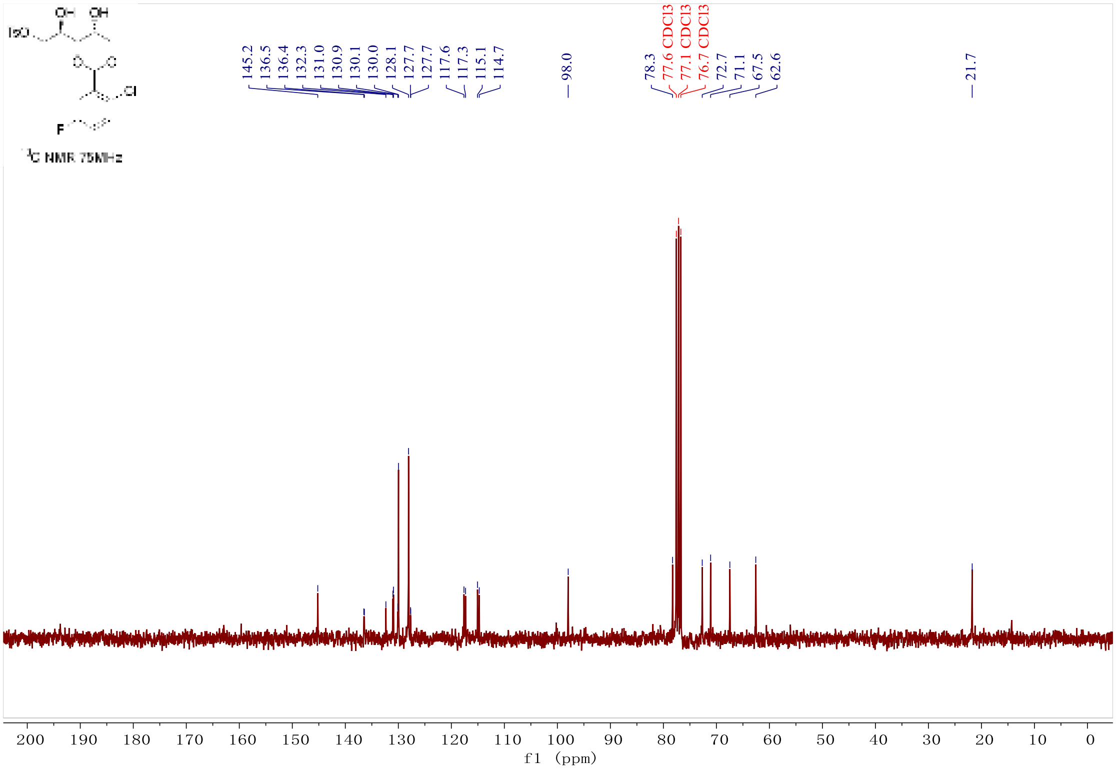

^{1}H NMR Spectrum of **13f** (300 MHz, $CDCl_3$)

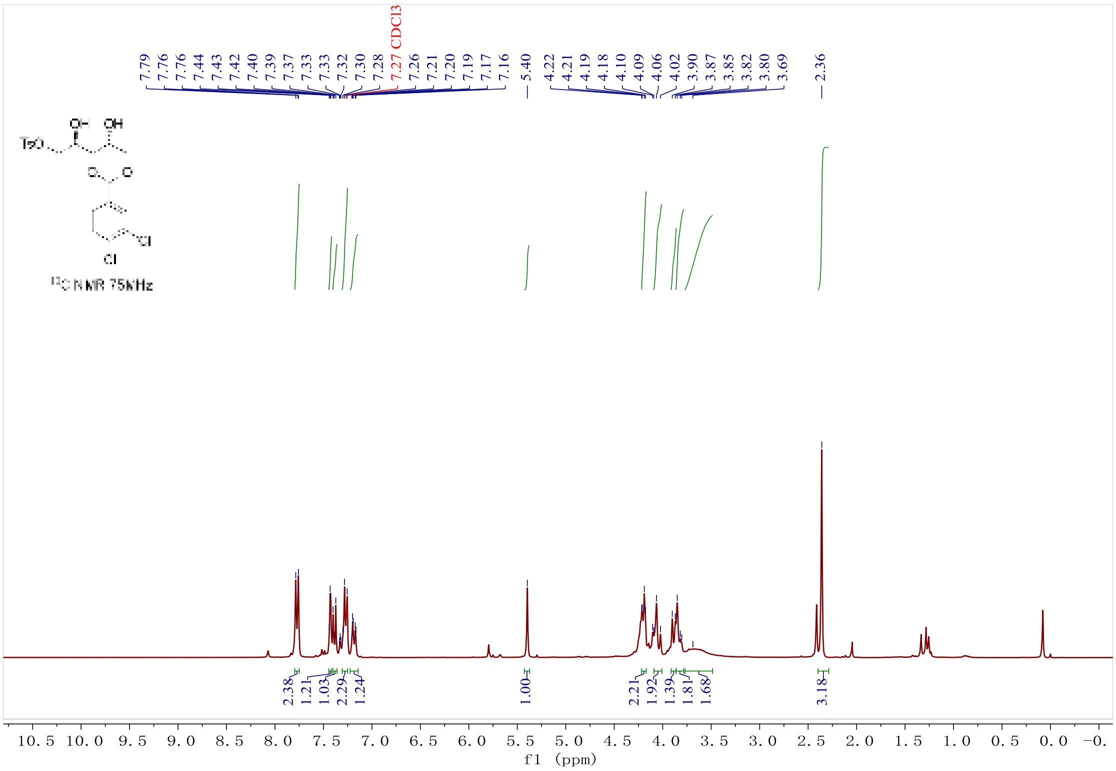

^{13}C NMR Spectrum of **13f** (75 MHz, $CDCl_3$)

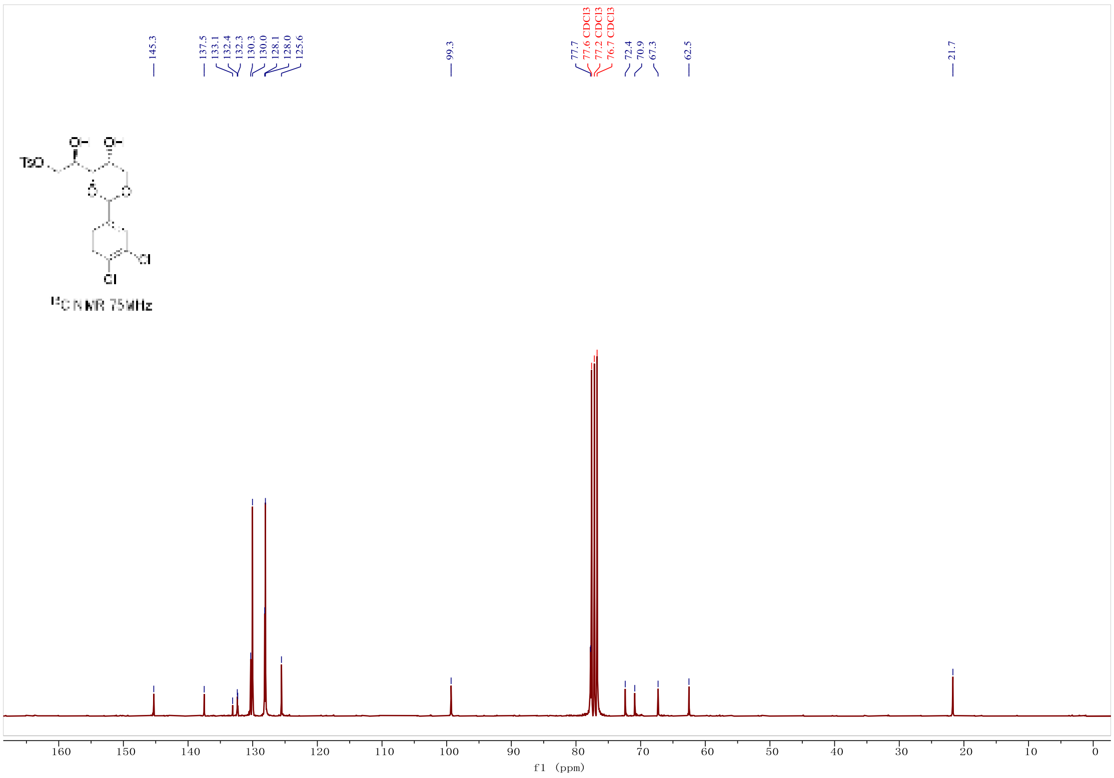

^{1}H NMR Spectrum of **13g** (300 MHz, $CDCl_3$)

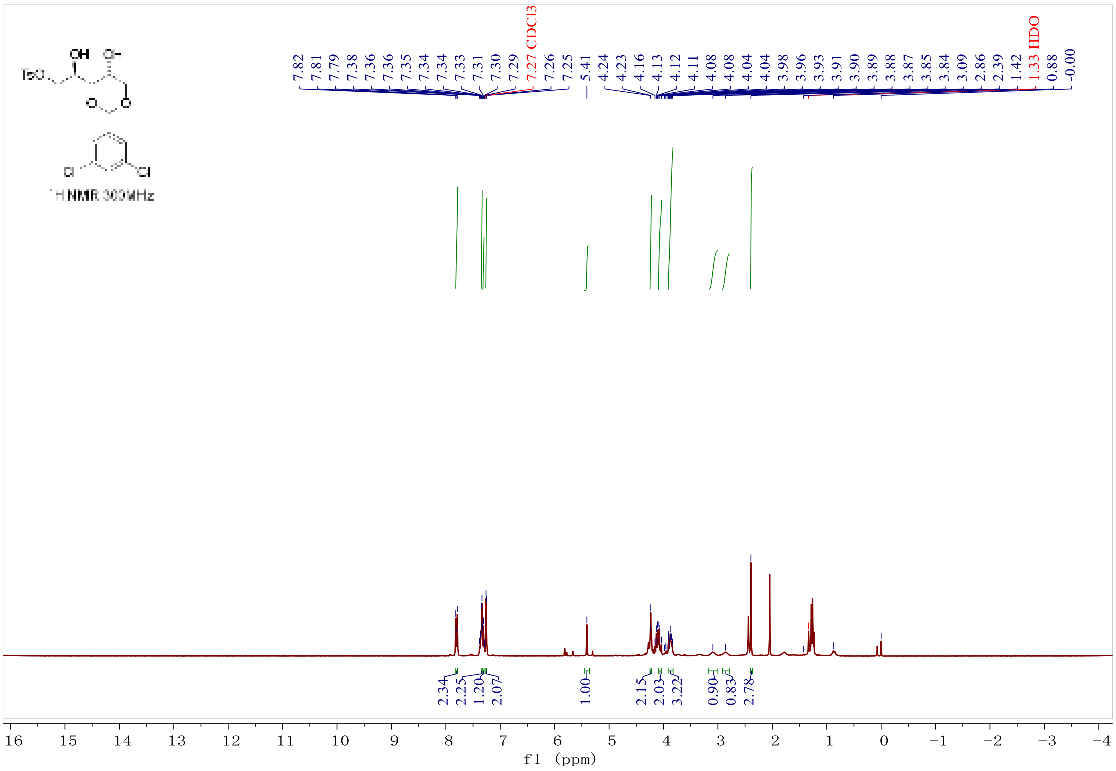

^{13}C NMR Spectrum of **13g** (75 MHz, $CDCl_3$)

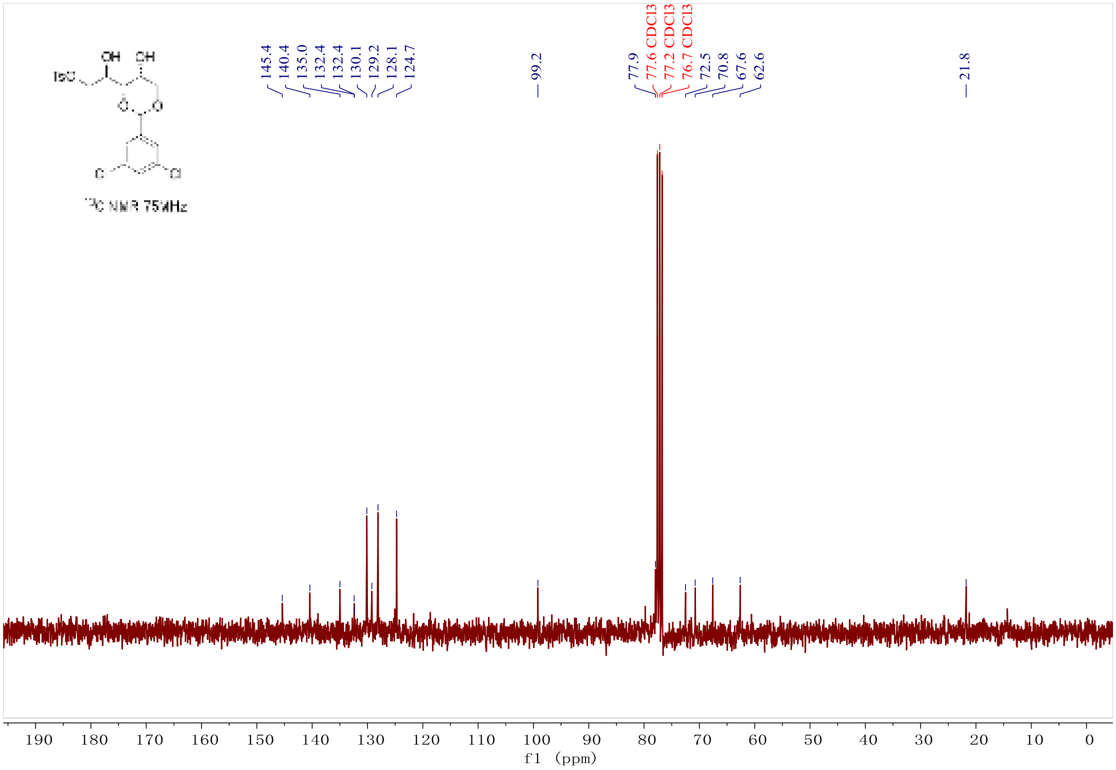

^{1}H NMR Spectrum of **13h** (300 MHz, $CDCl_3$)

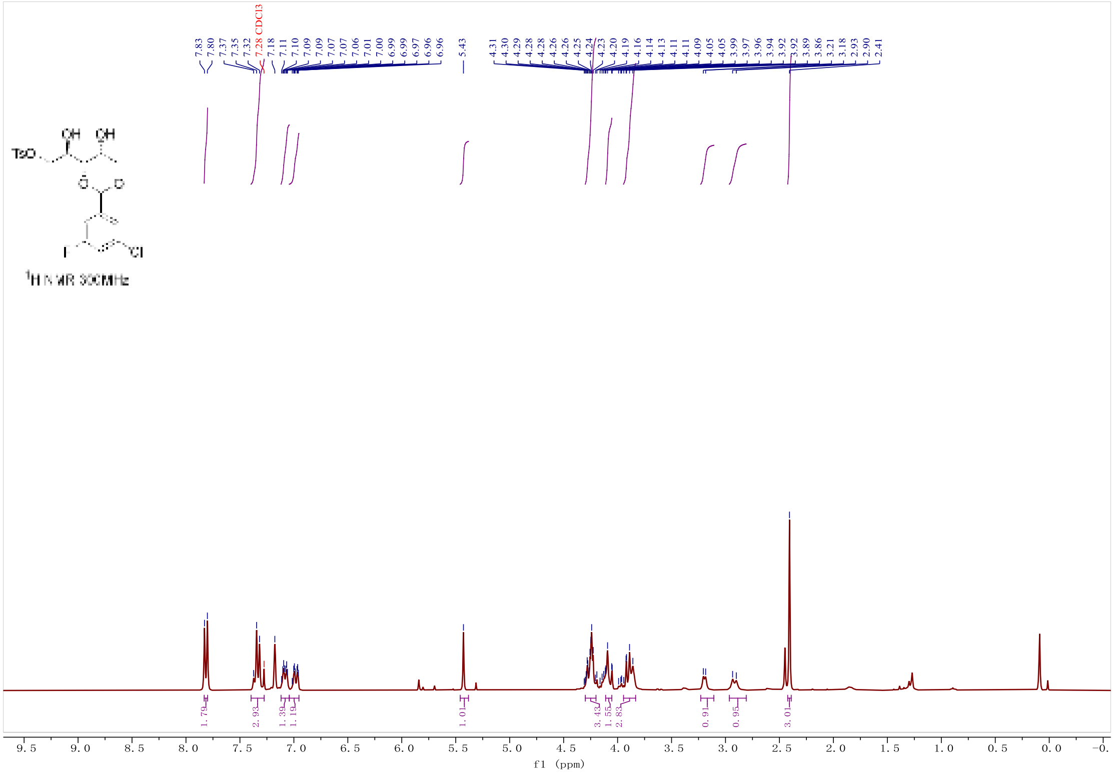

^{13}C NMR Spectrum of **13h** (75 MHz, $CDCl_3$)

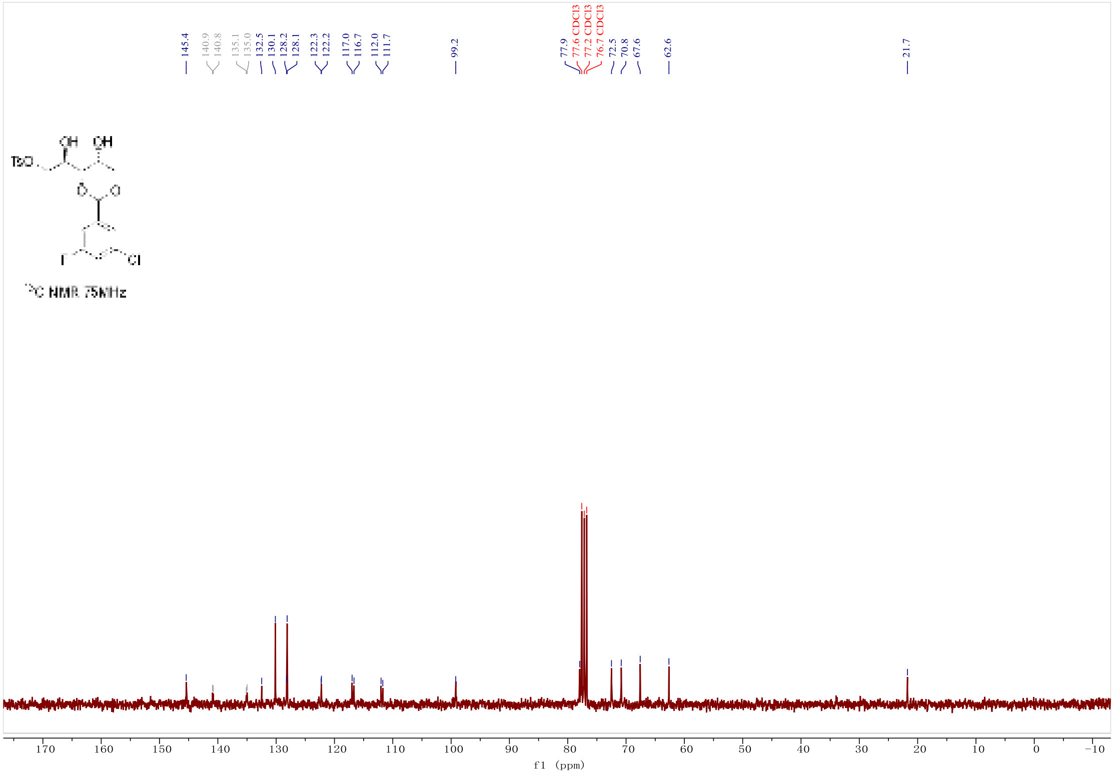

^{1}H NMR Spectrum of **13i** (300 MHz, $CDCl_3$)

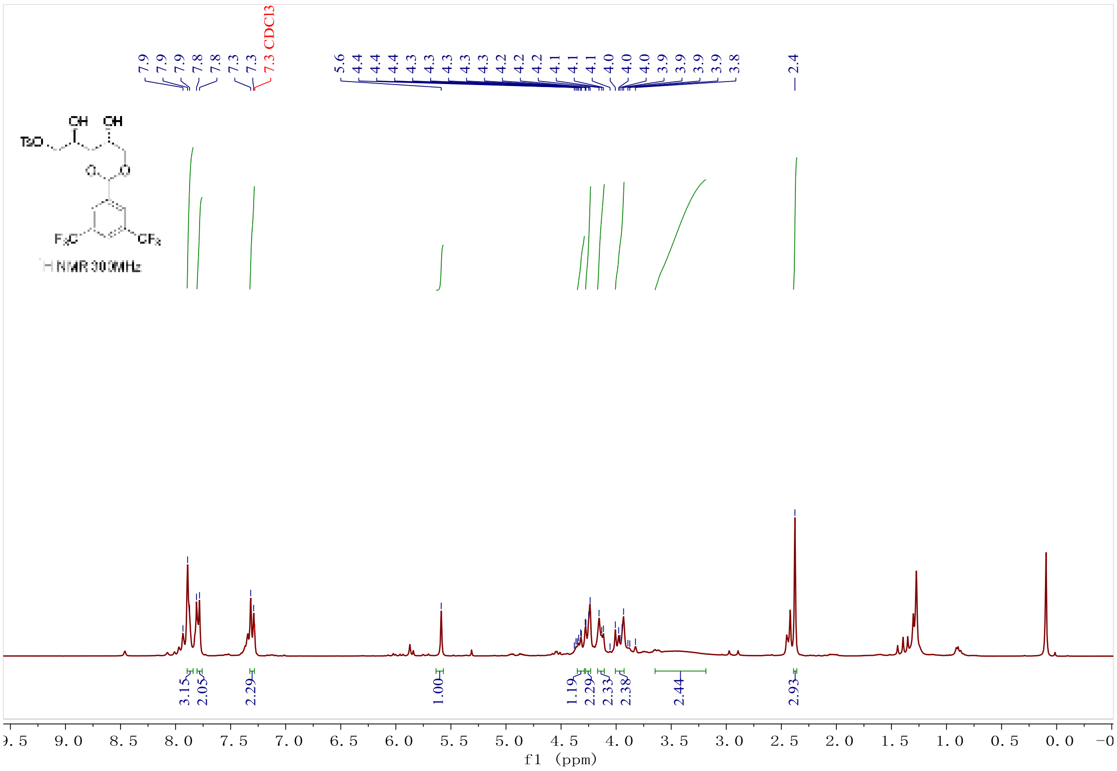

^{13}C NMR Spectrum of **13i** (75 MHz, $CDCl_3$)

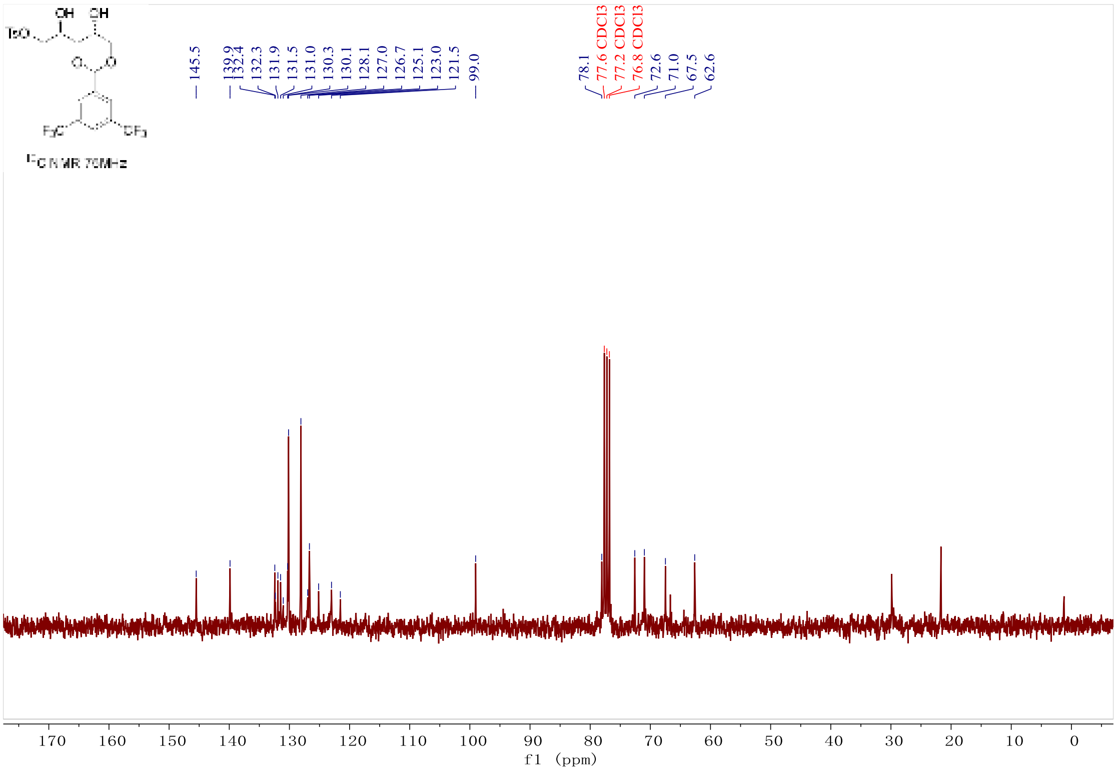

^{1}H NMR Spectrum of **13j** (300 MHz, $CDCl_3$)

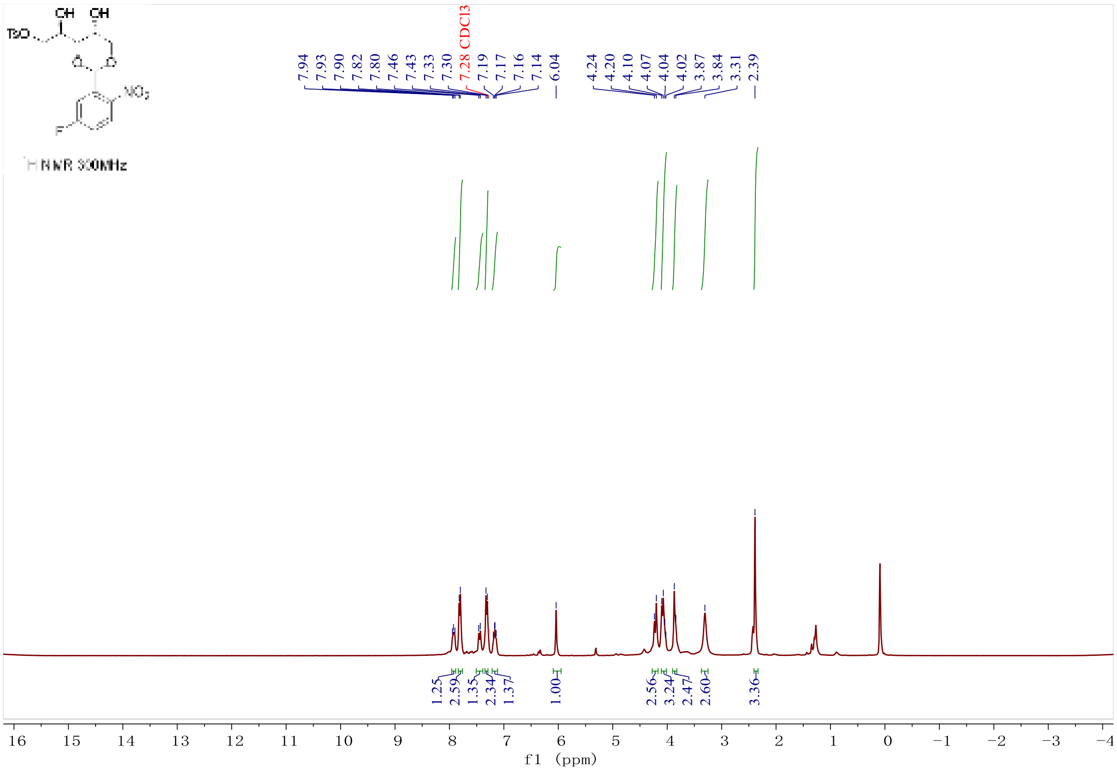

^{13}C NMR Spectrum of **13j** (75 MHz, $CDCl_3$)

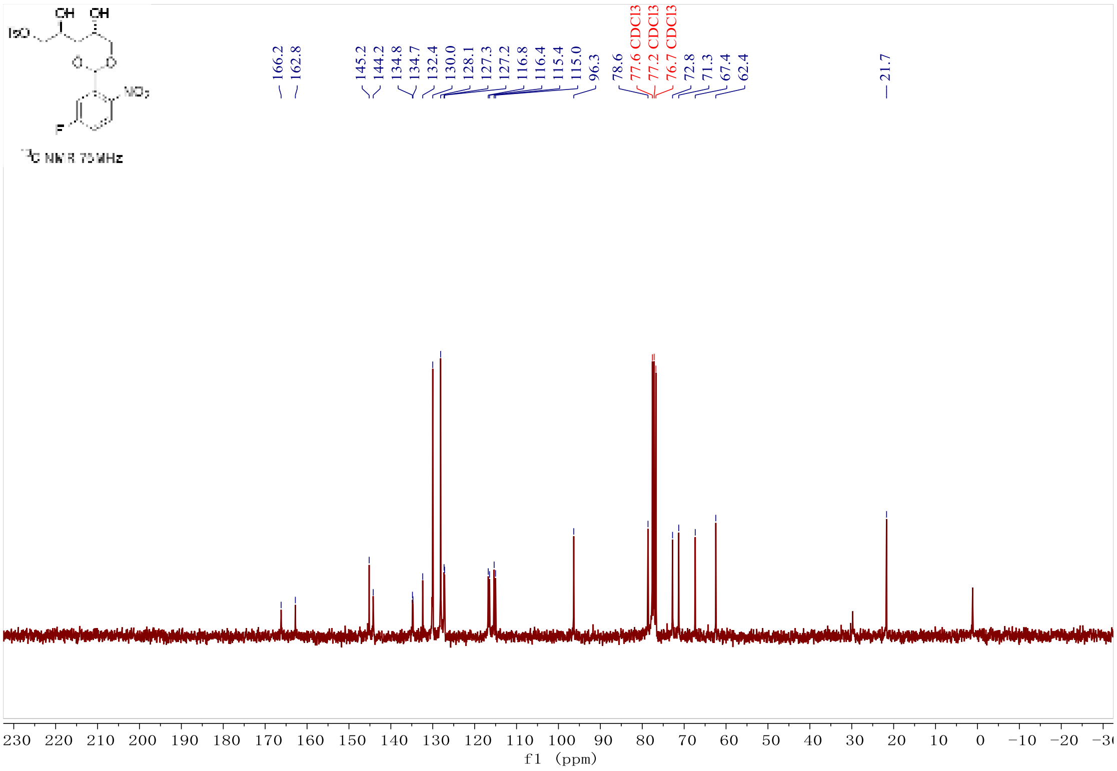

^{1}H NMR Spectrum of **13k** (300 MHz, $CDCl_3$)

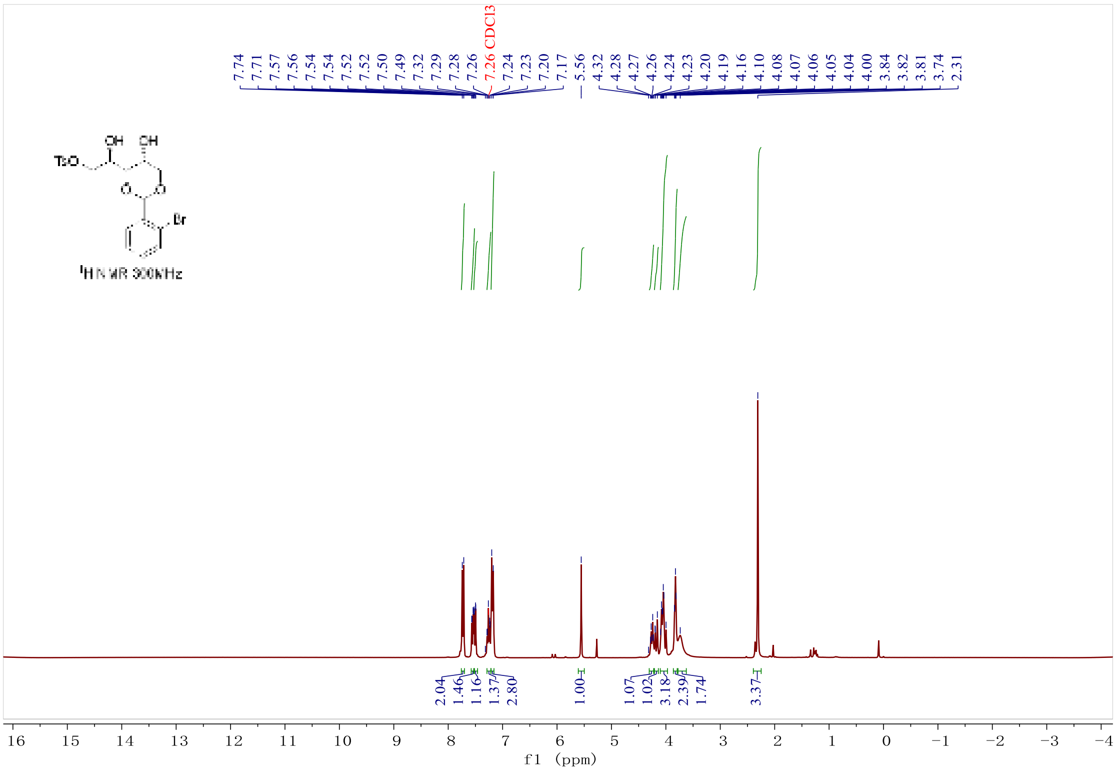

^{13}C NMR Spectrum of **13k** (75 MHz, $CDCl_3$)

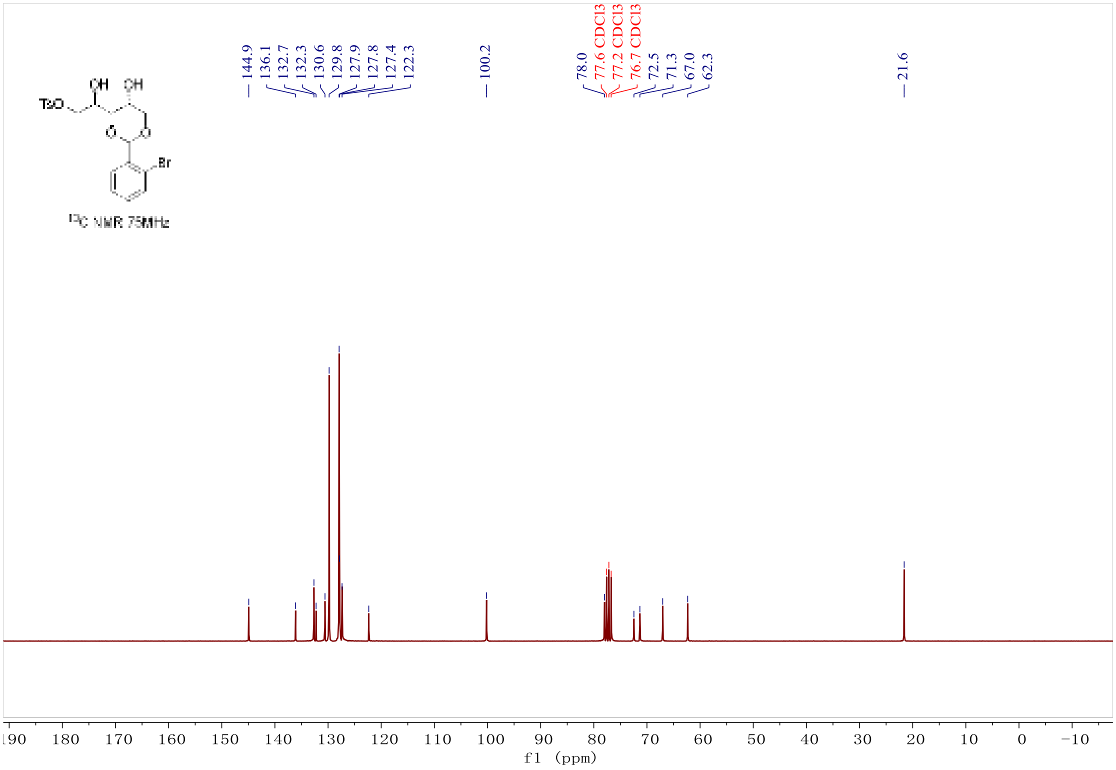

^{1}H NMR Spectrum of **13l** (300 MHz, $CDCl_3$)

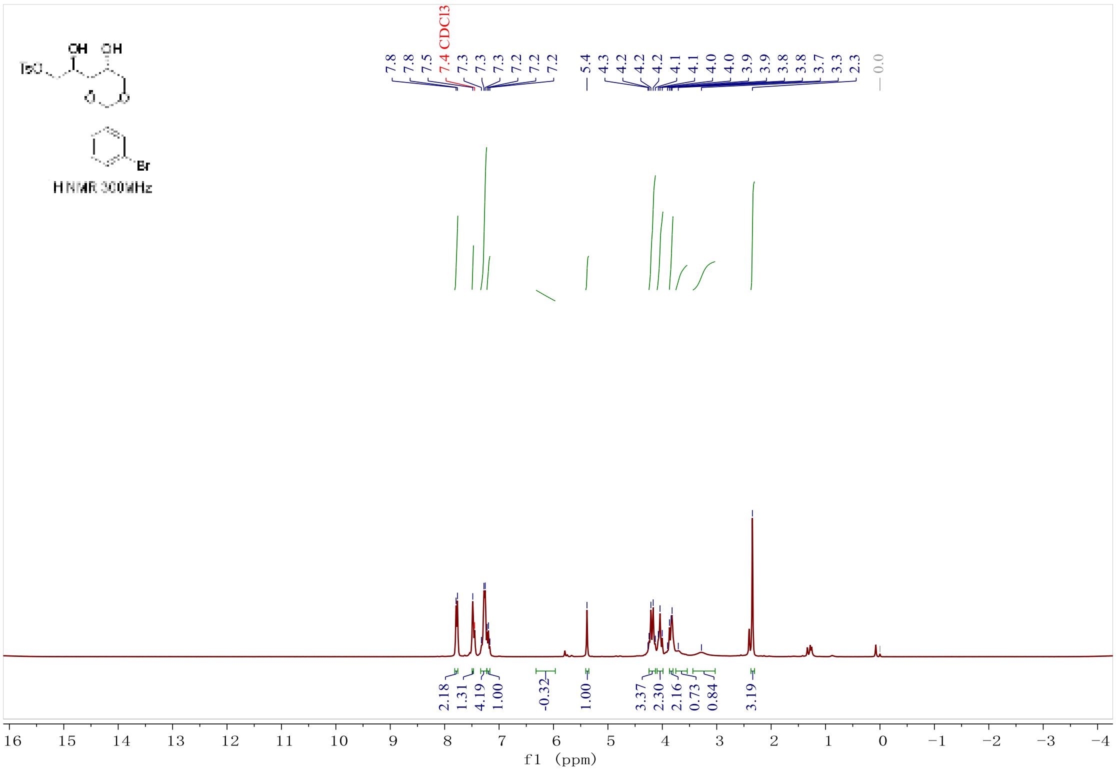

^{13}C NMR Spectrum of **13l** (75 MHz, $CDCl_3$)

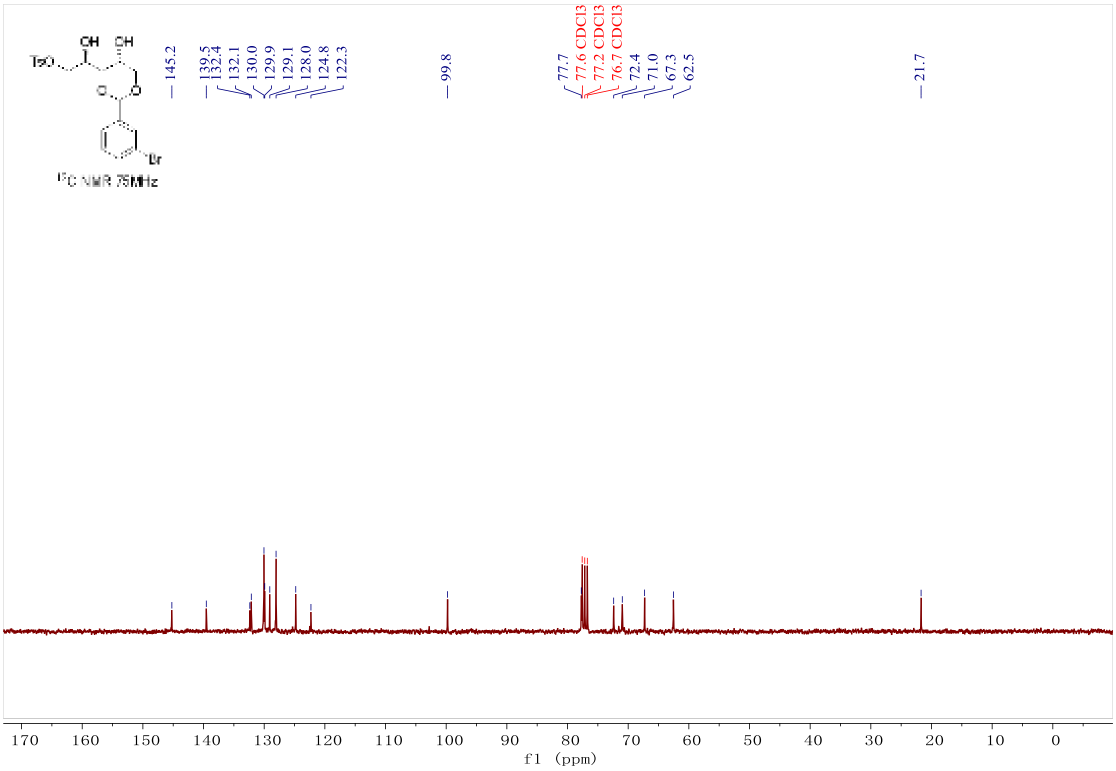

^{1}H NMR Spectrum of **13m** (300 MHz, $CDCl_3$)

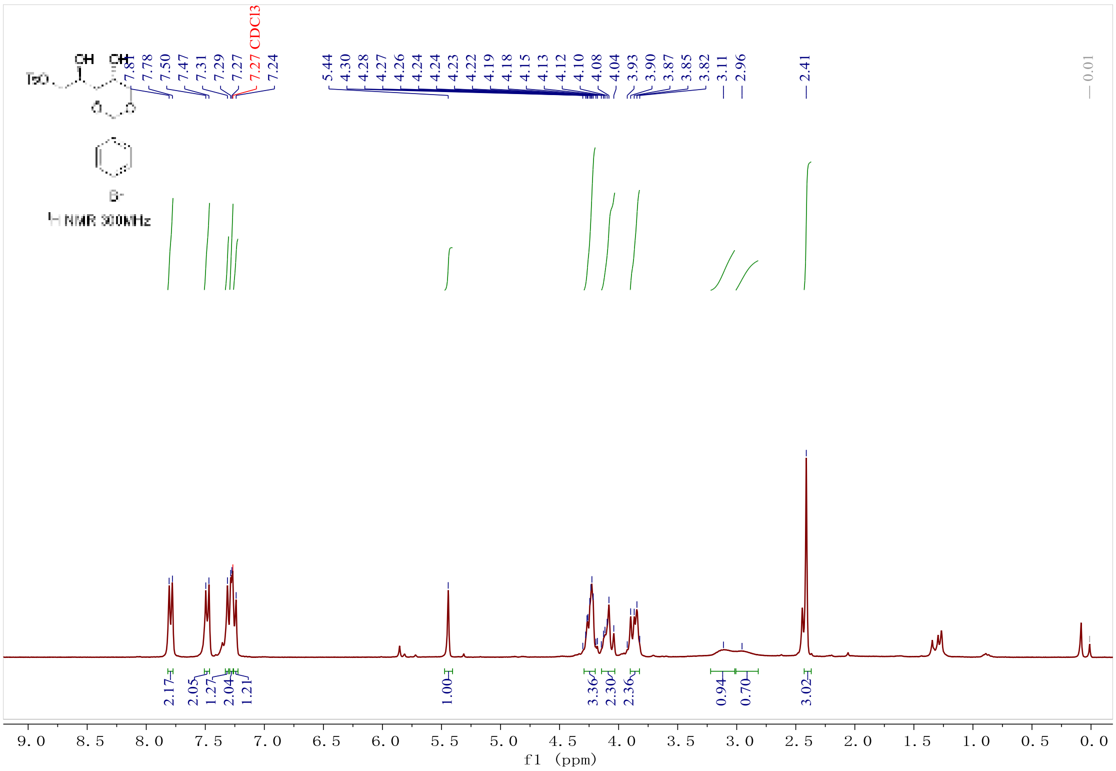

^{13}C NMR Spectrum of **13m** (75 MHz, $CDCl_3$)

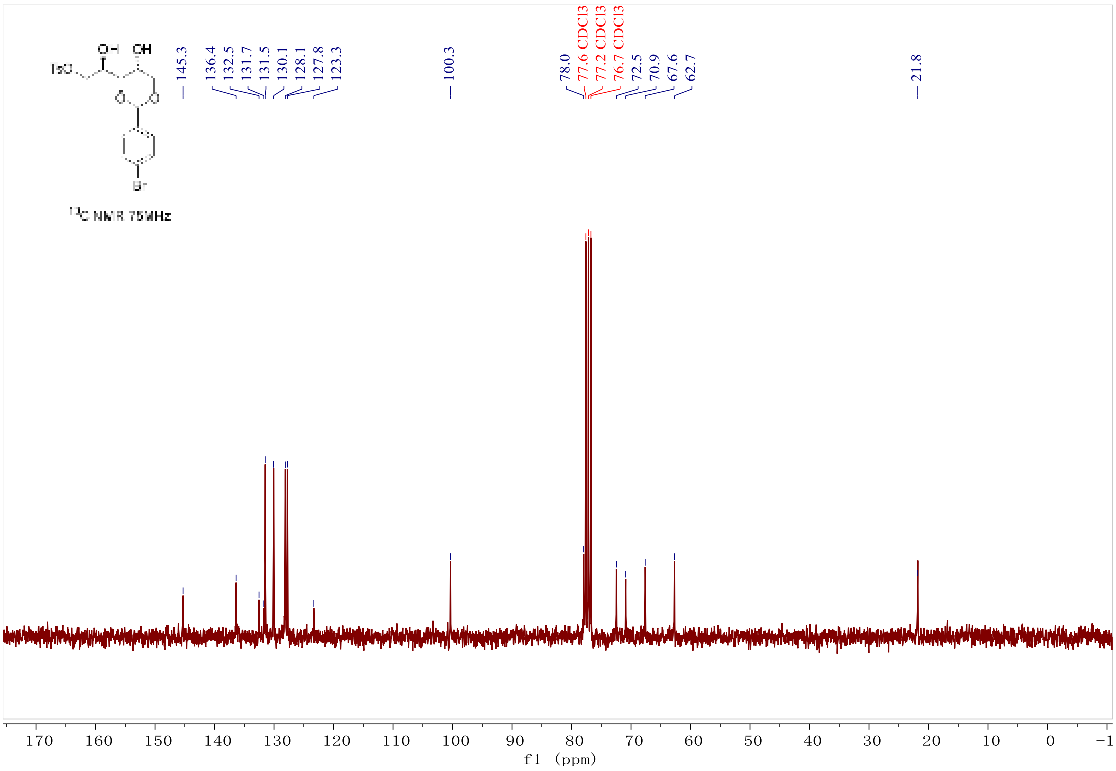

^{1}H-NMR spectrum of **13n**

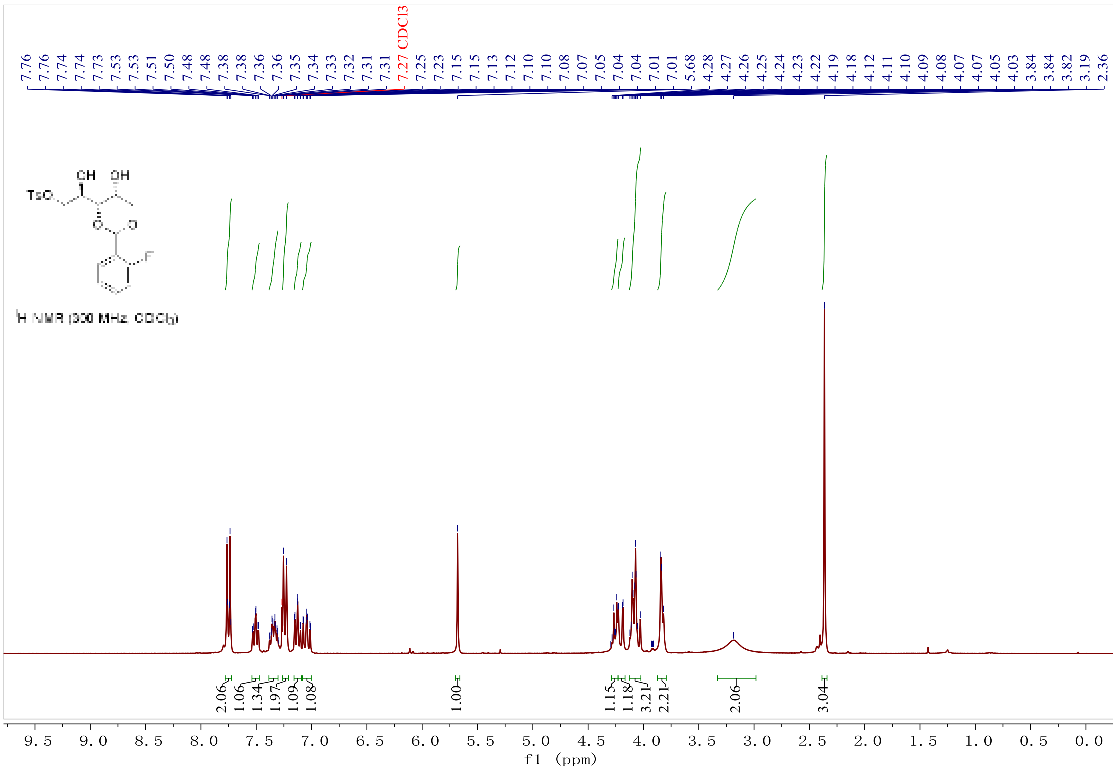

^{13}C-NMR spectrum of **13n**

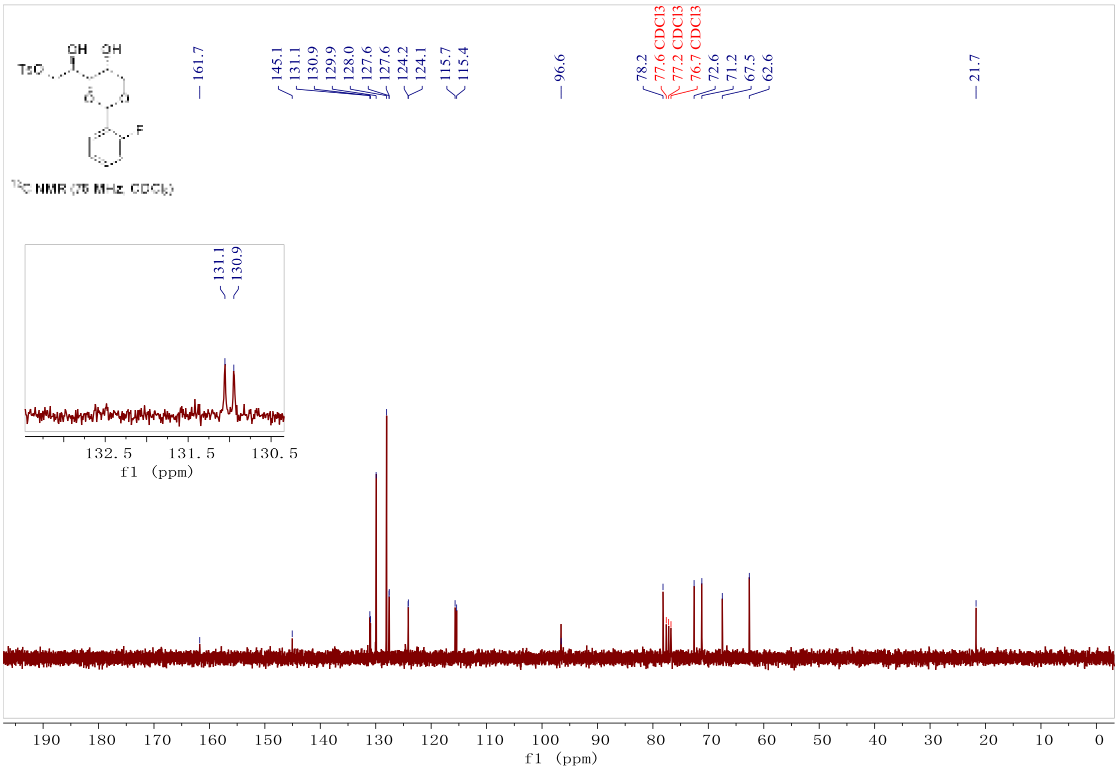

^{1}H NMR Spectrum of **13o** (300 MHz, $CDCl_3$)

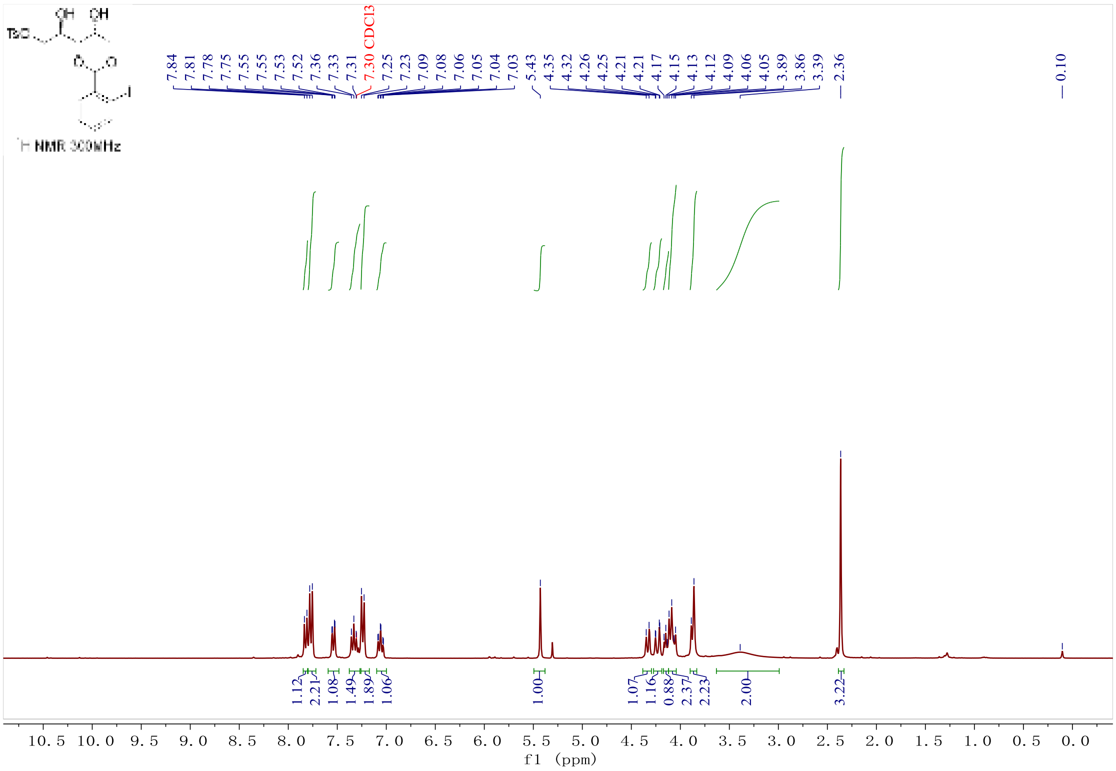

^{13}C NMR Spectrum of **13o** (75 MHz, $CDCl_3$)

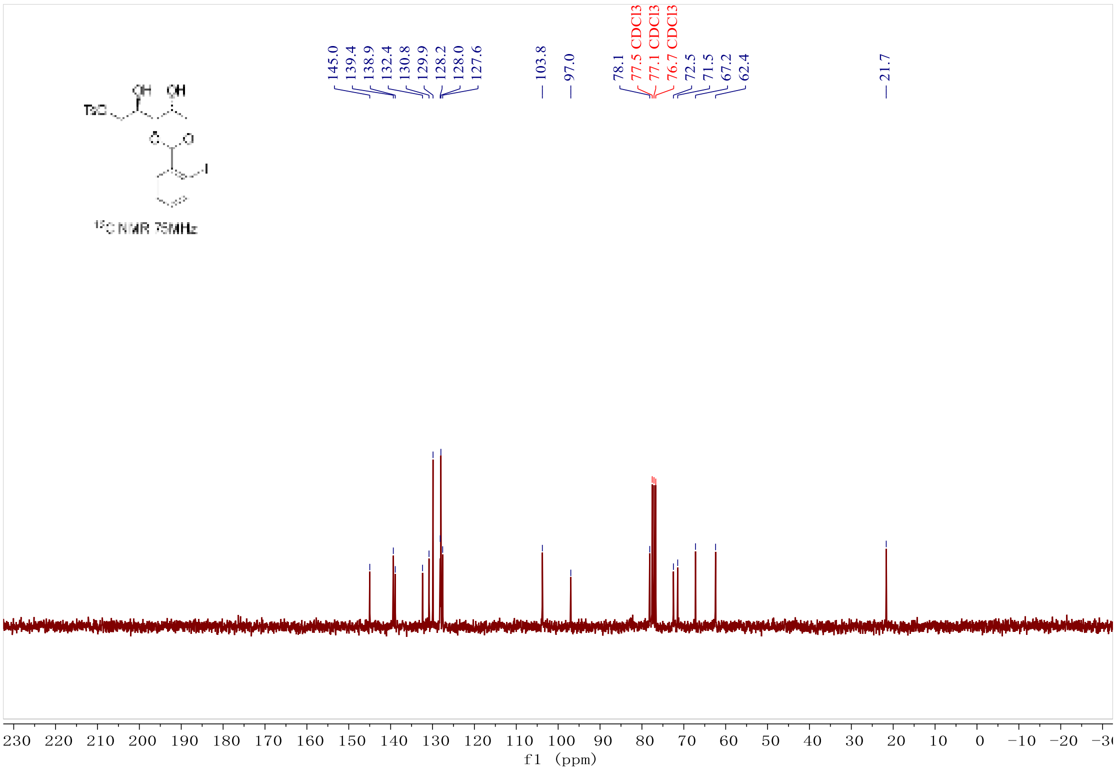

^{1}H NMR Spectrum of **13p** (300 MHz, $CDCl_3$)

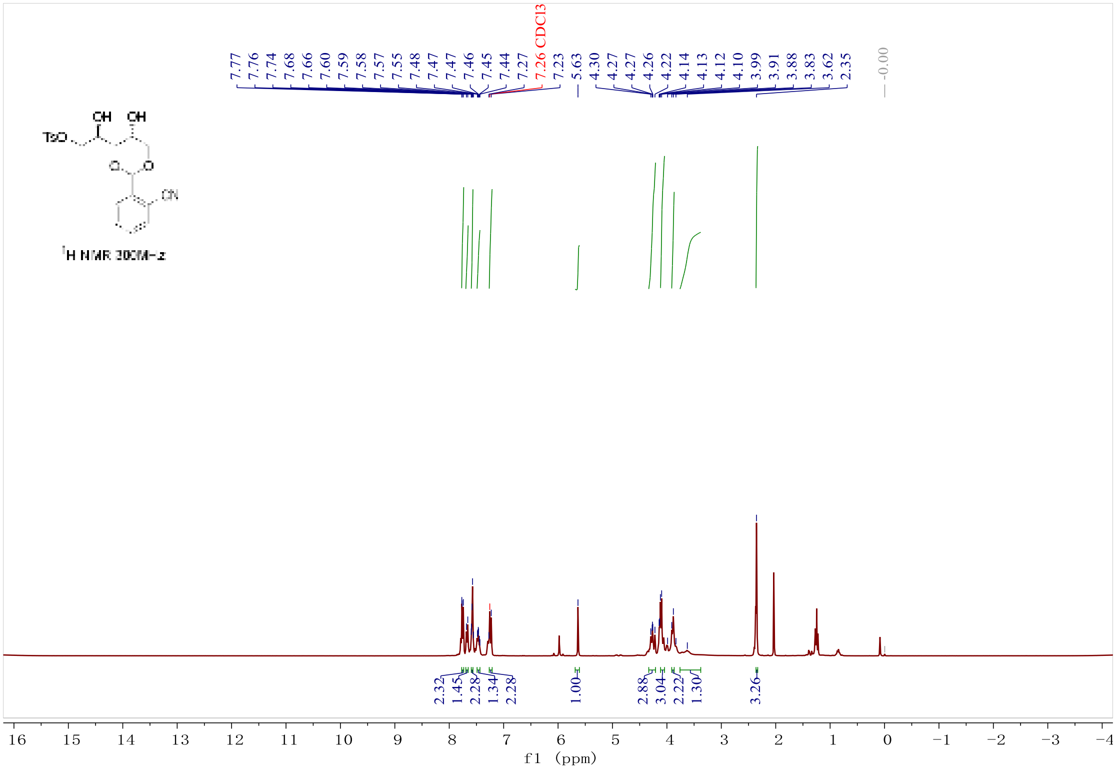

^{13}C NMR Spectrum of **13p** (75 MHz, $CDCl_3$)

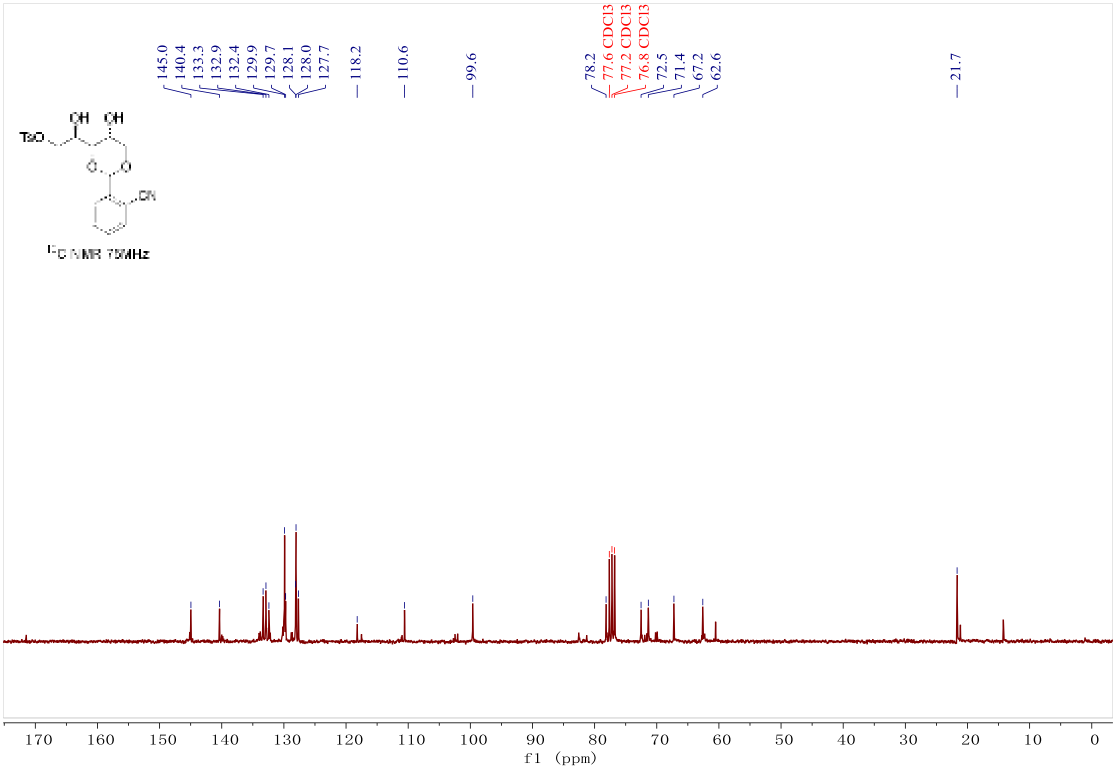

^{1}H NMR Spectrum of **13q** (300 MHz, $CDCl_3$)

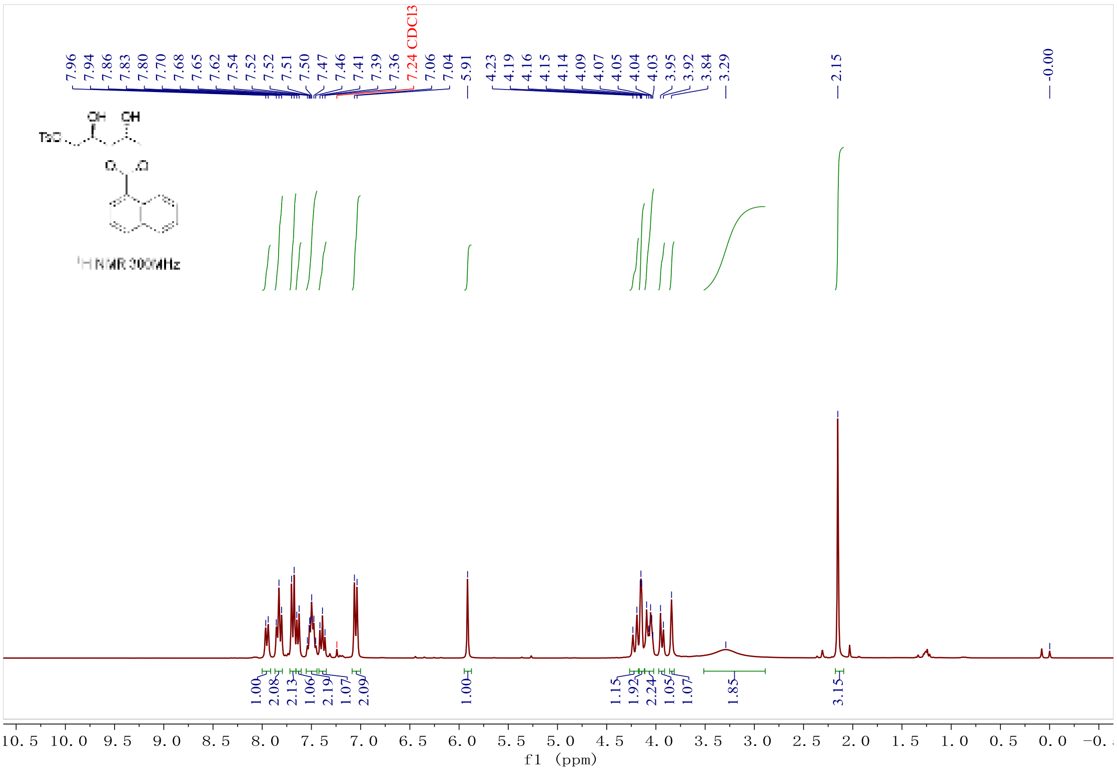

^{13}C NMR Spectrum of **13q** (75 MHz, $CDCl_3$)

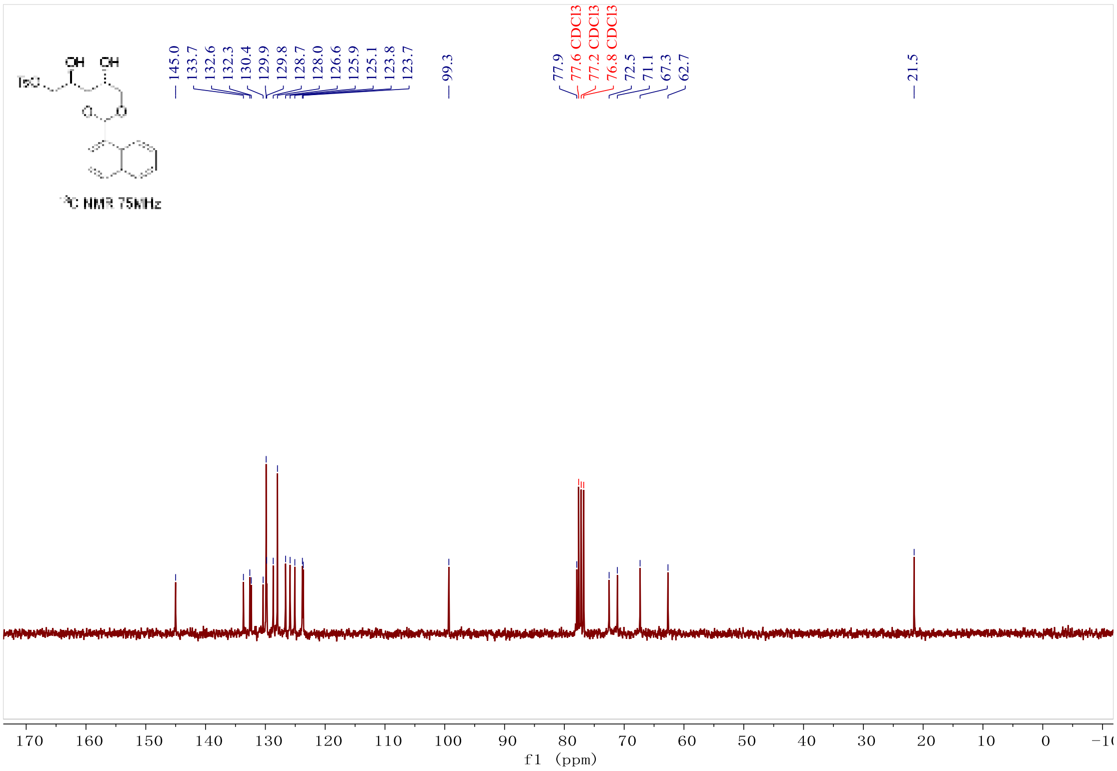

^{1}H NMR Spectrum of **13r** (300 MHz, CDCl3)

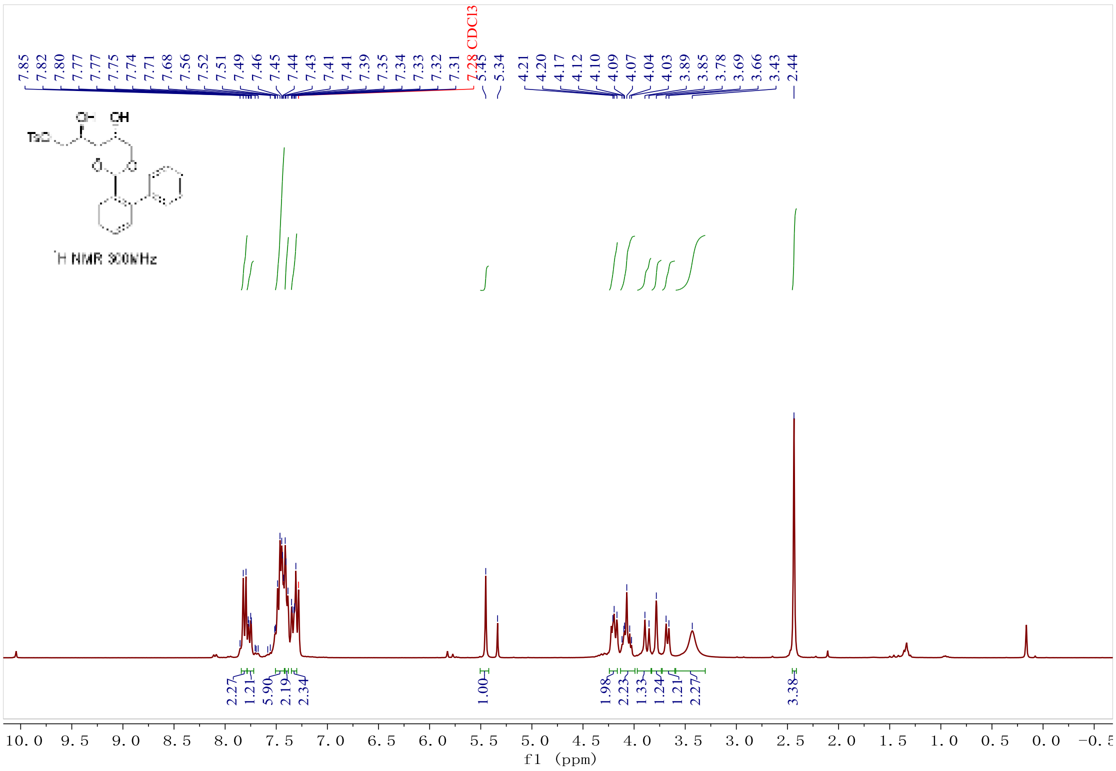

^{13}C NMR Spectrum of **13r** (75 MHz, CDCl3)

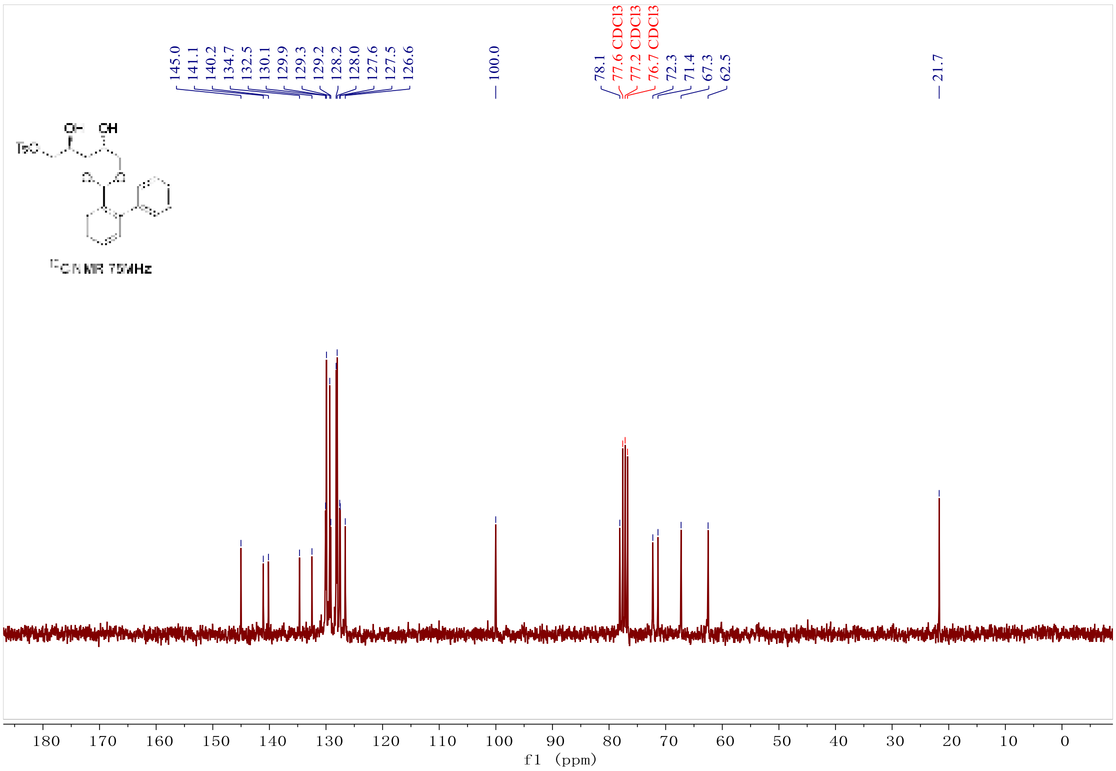

^{1}H-NMR spectrum of **14a**

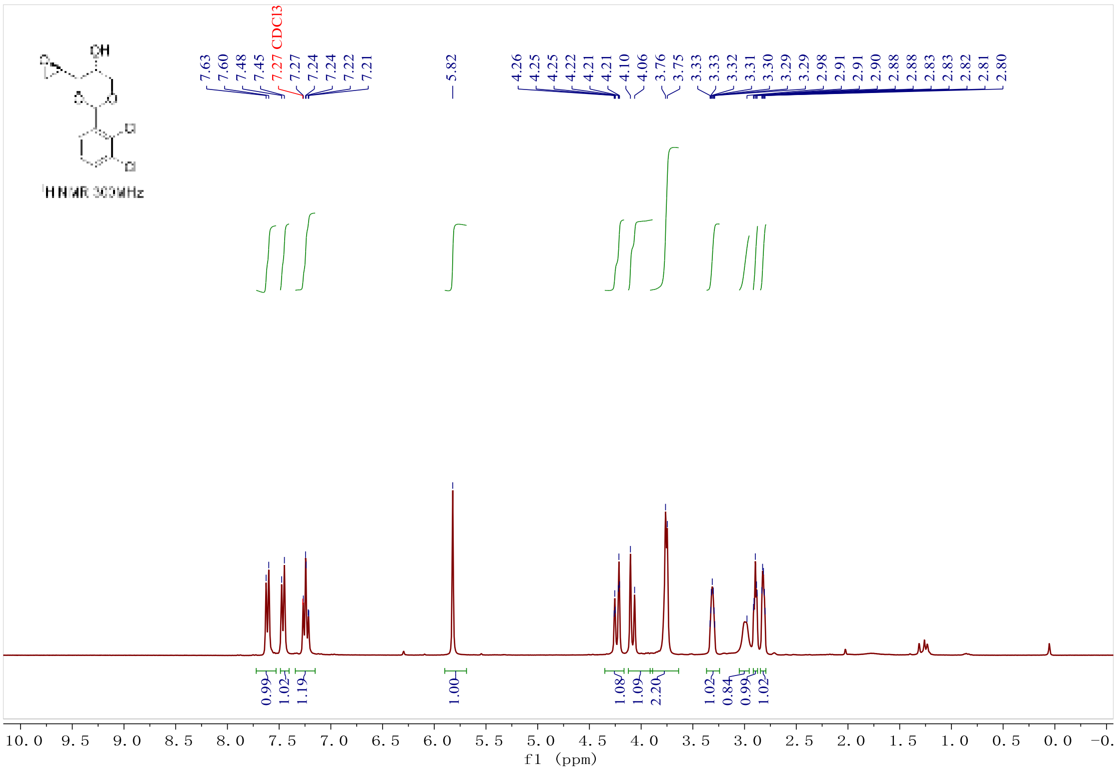

^{13}C-NMR spectrum of **14a**

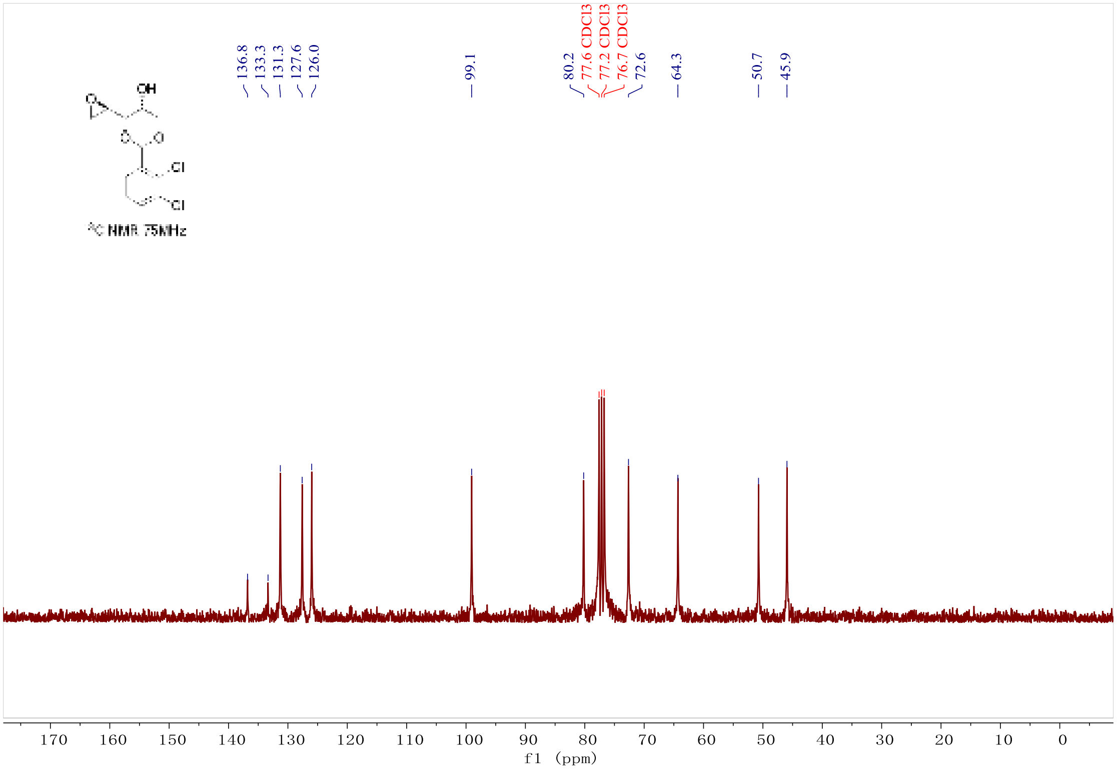

^{1}H-NMR spectrum of **14b**

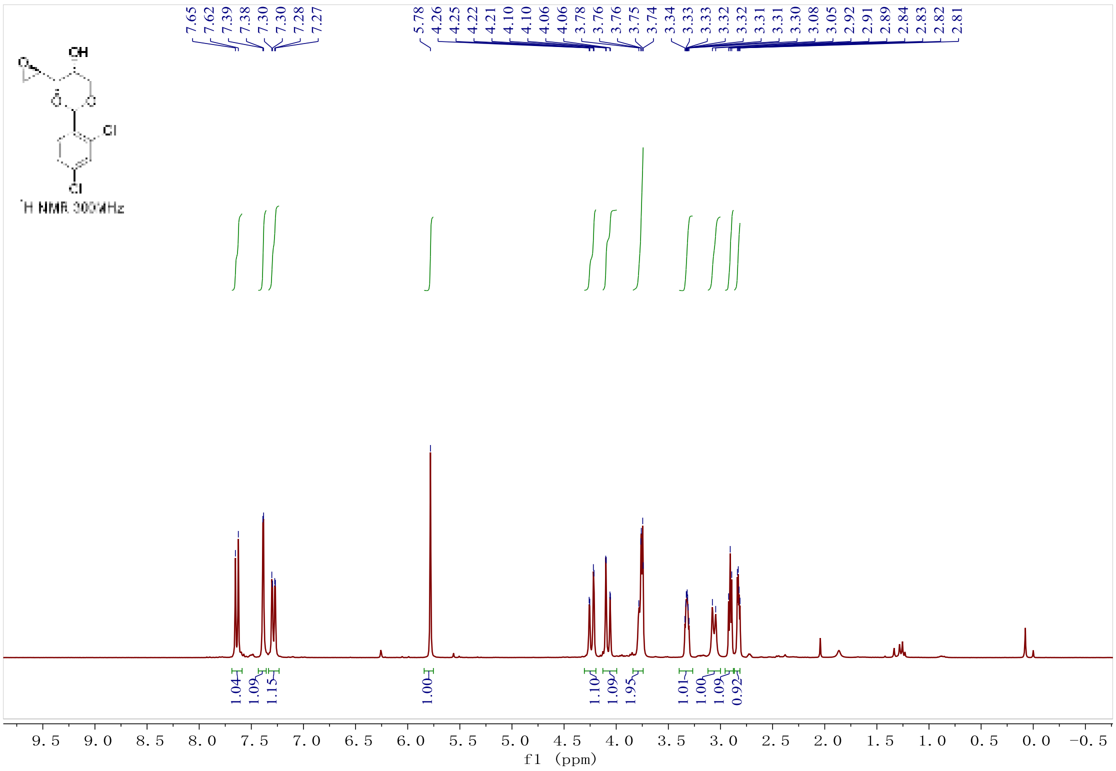

^{13}C-NMR spectrum of **14b**

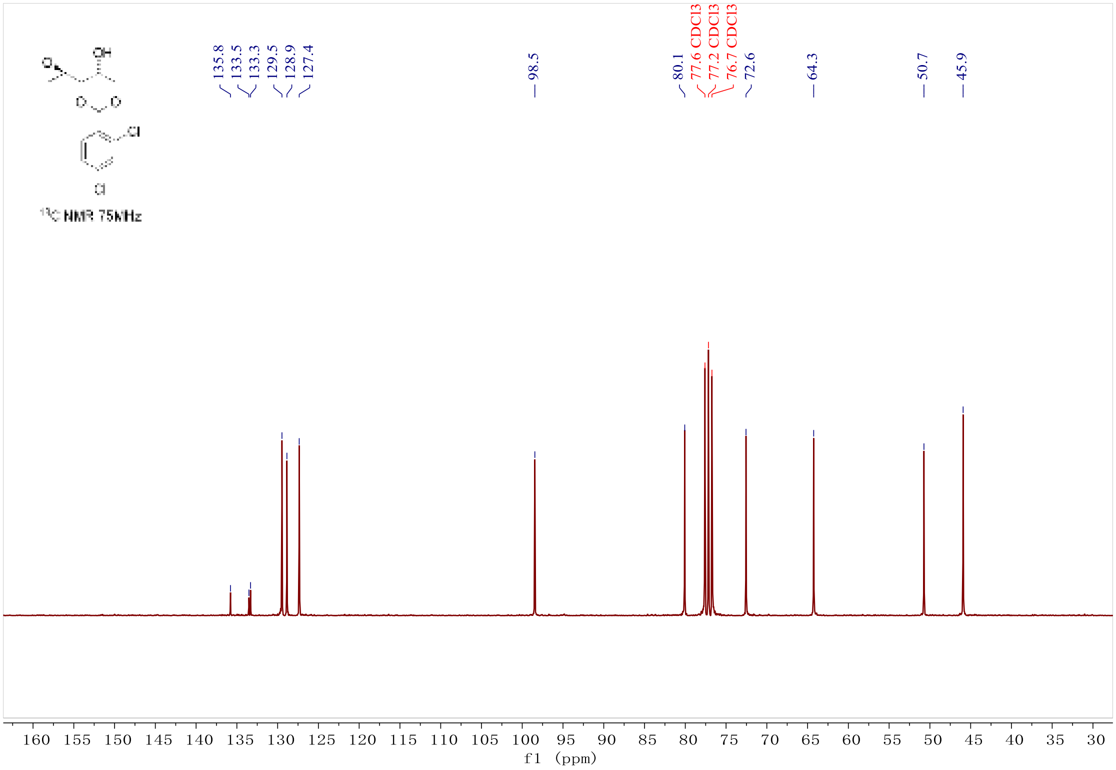

^{1}H NMR Spectrum of **14c**

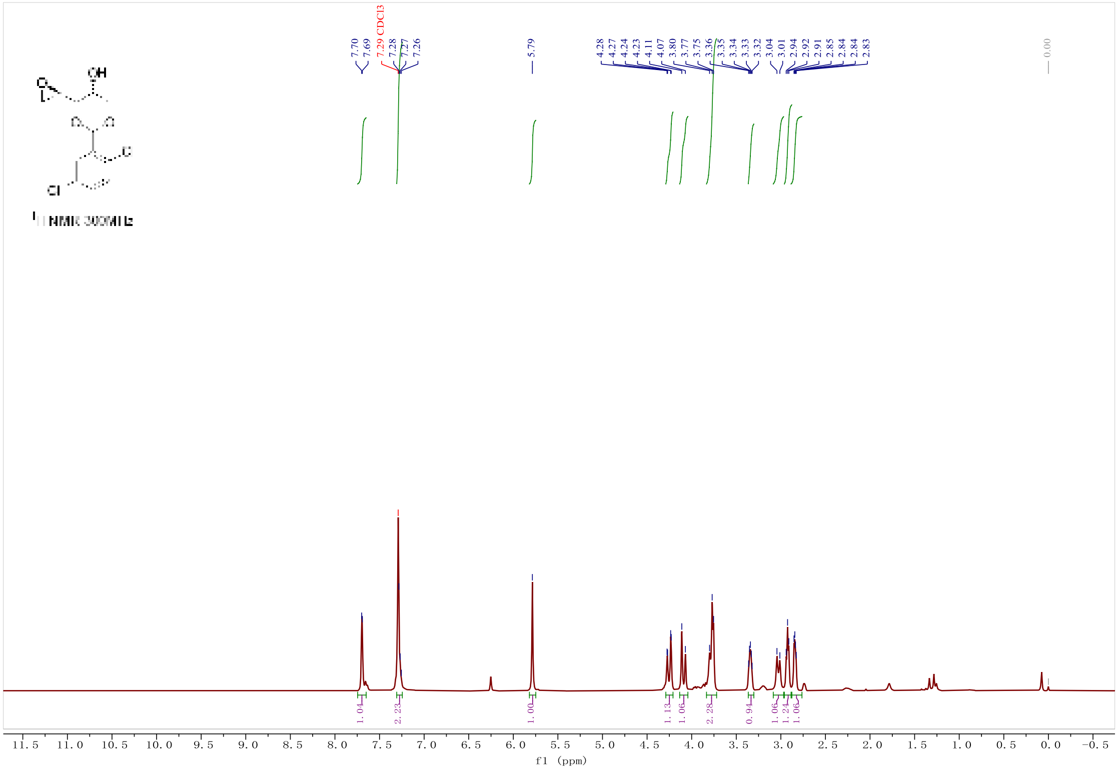

^{13}C NMR Spectrum of **14c**

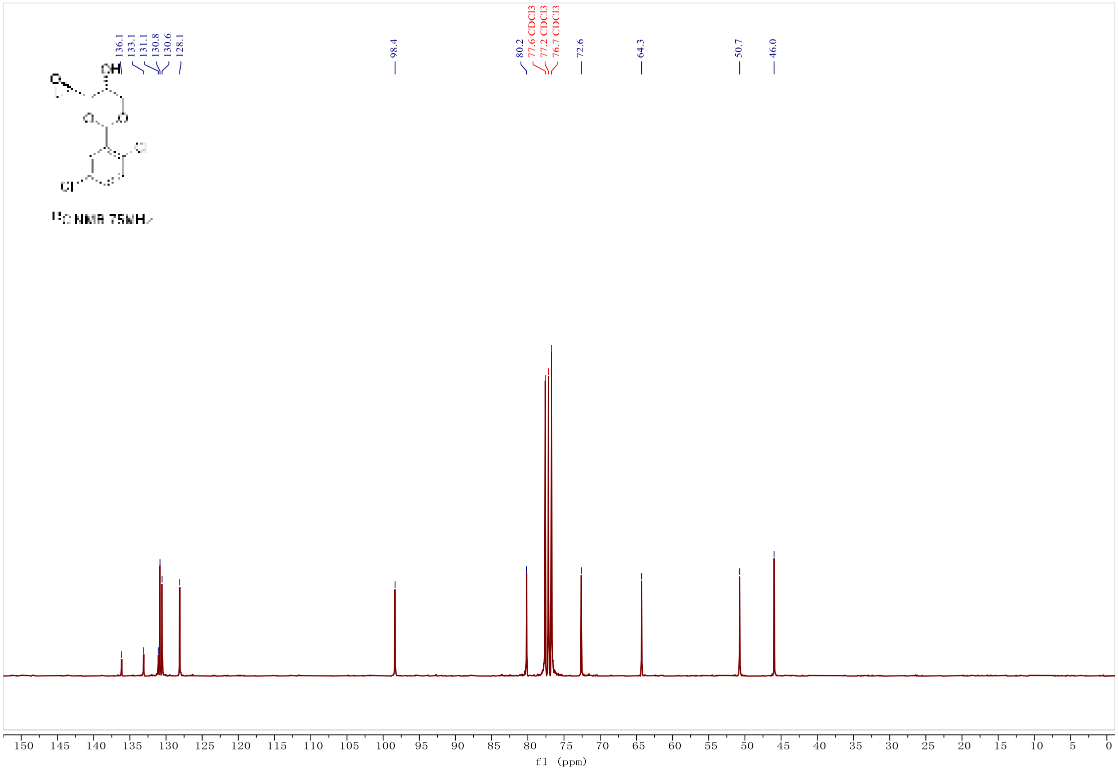

^{1}H NMR Spectrum of **14d**

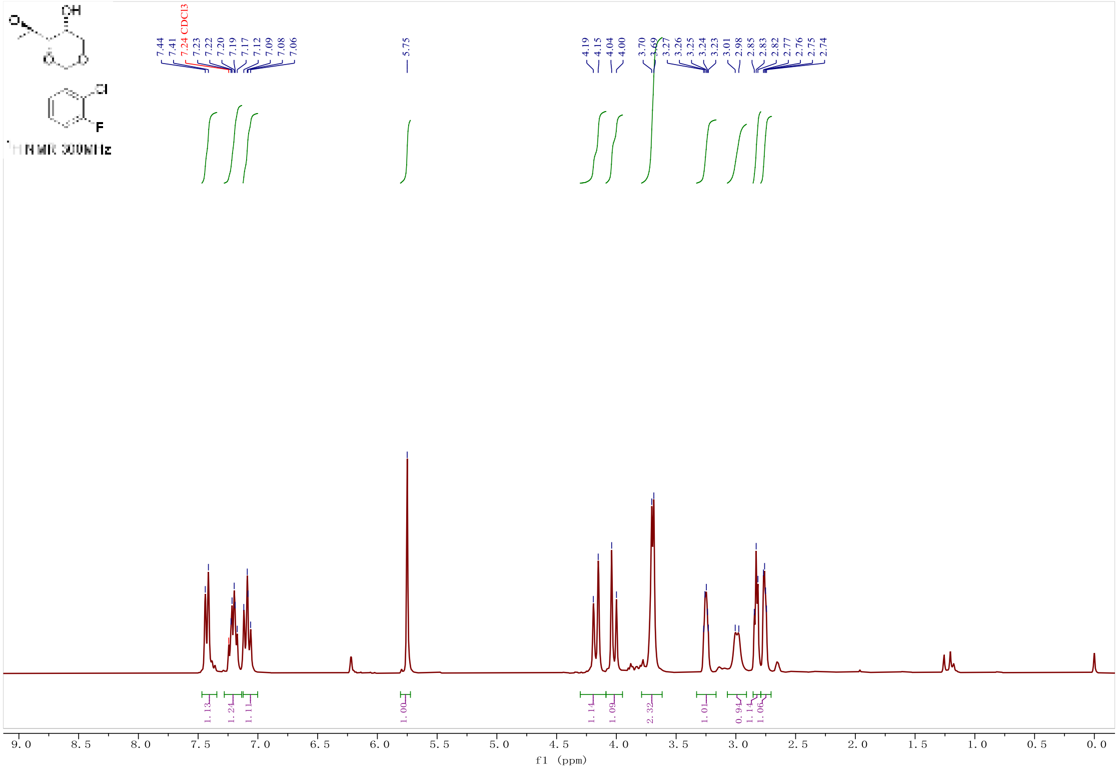

^{13}C NMR Spectrum of **14d**

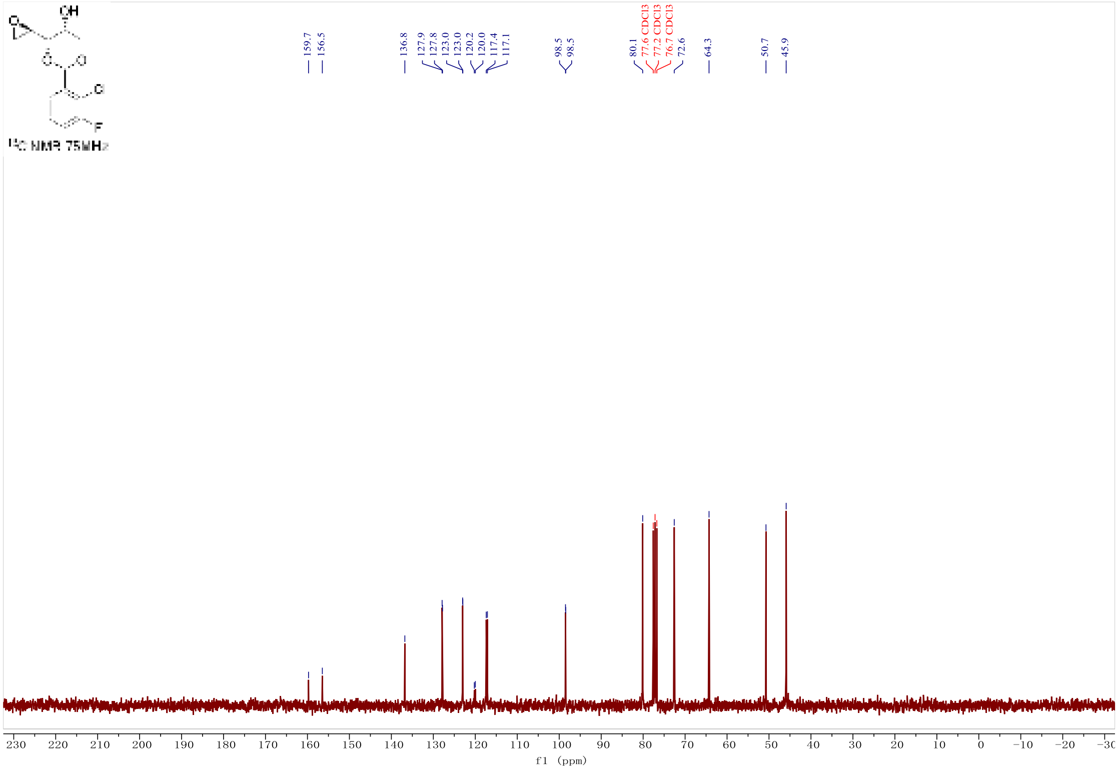

^{1}H NMR Spectrum of **14e**

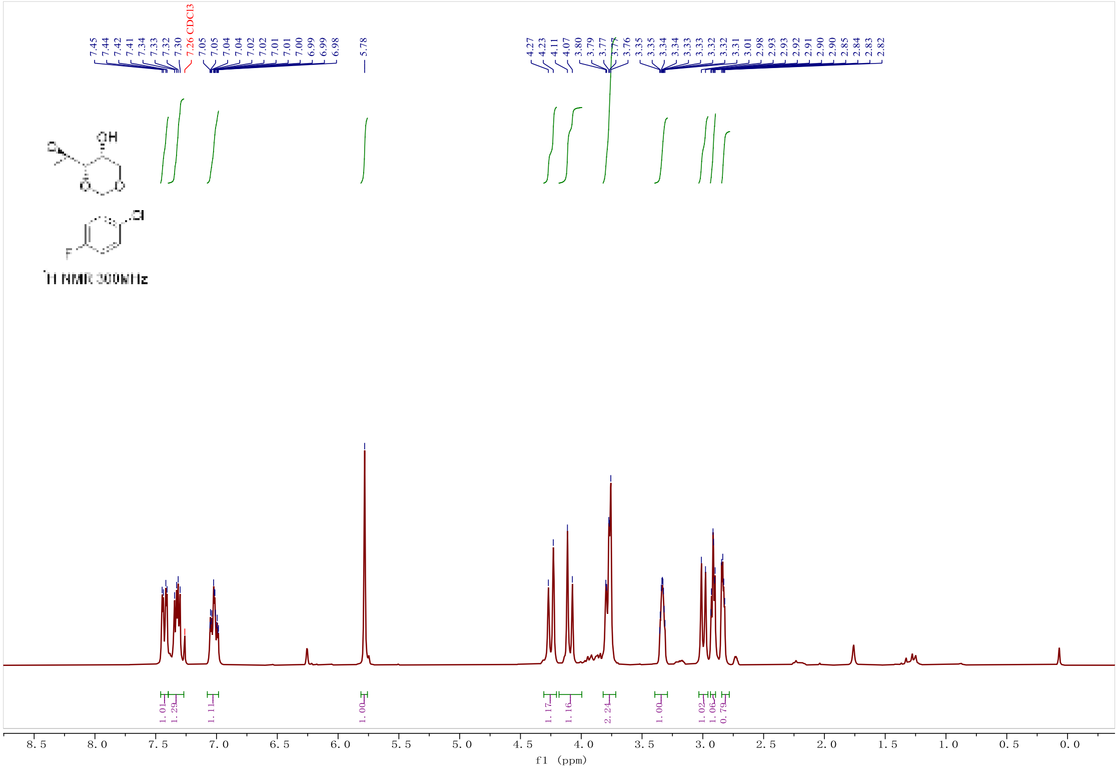

^{13}C NMR Spectrum of **14e**

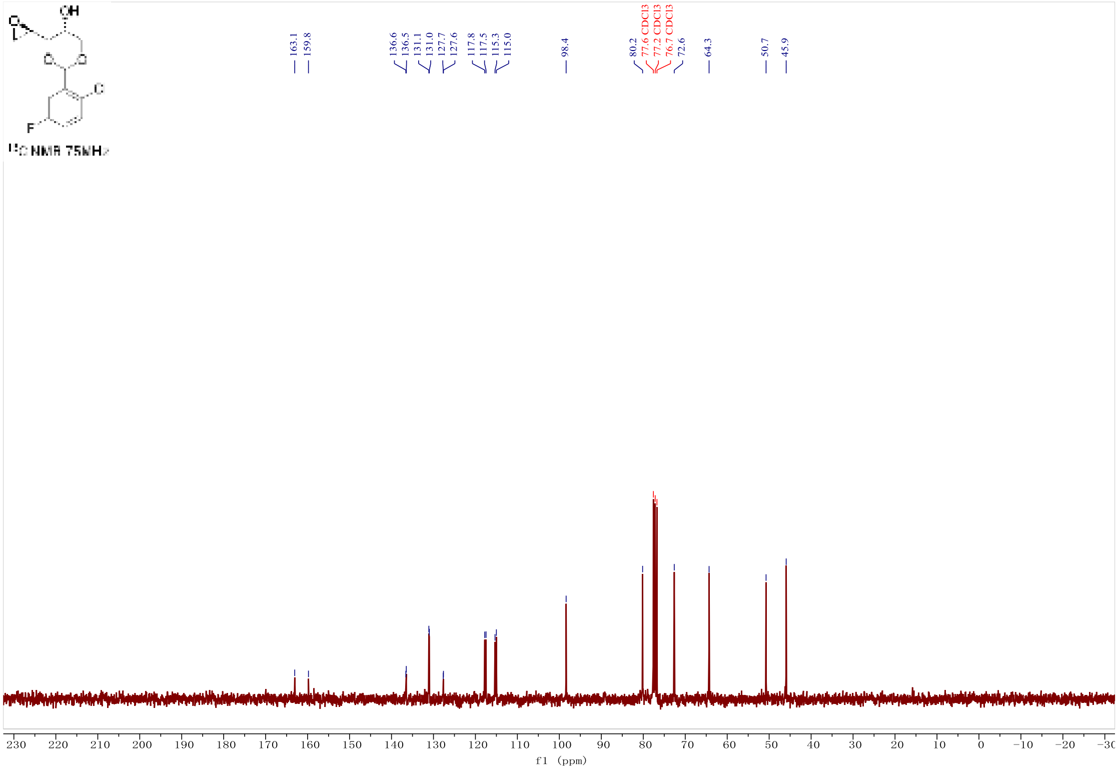

^{1}H NMR Spectrum of **14f**

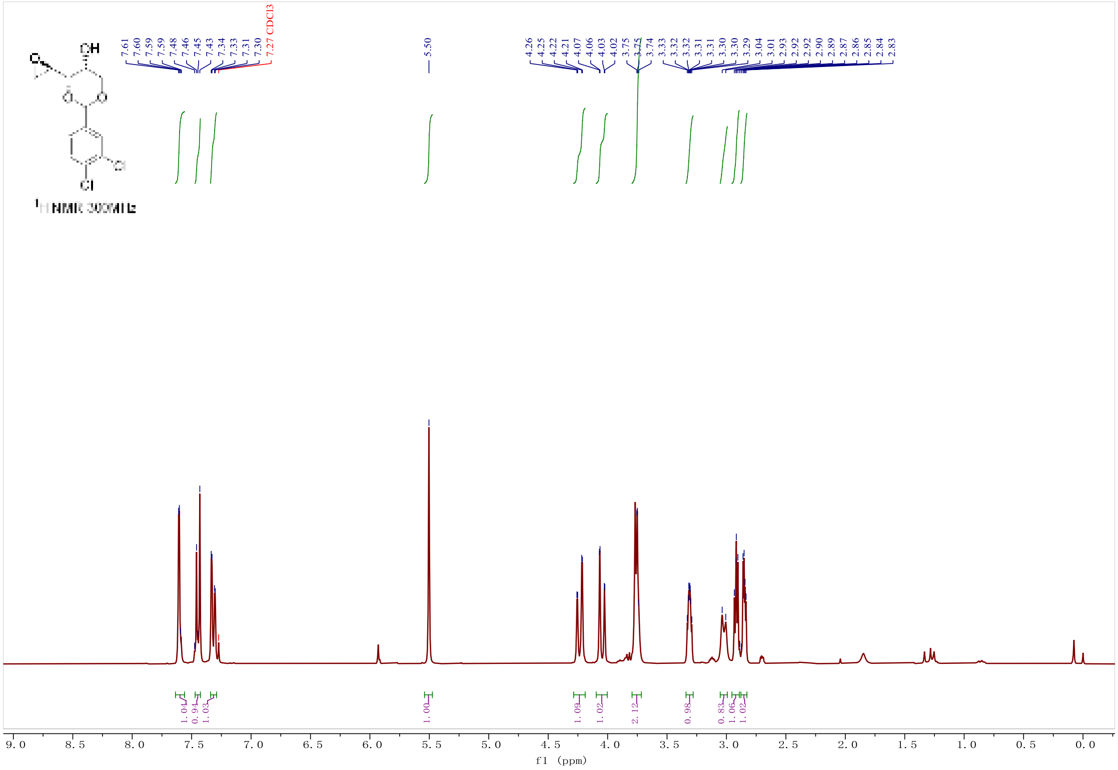

^{13}C NMR Spectrum of **14f**

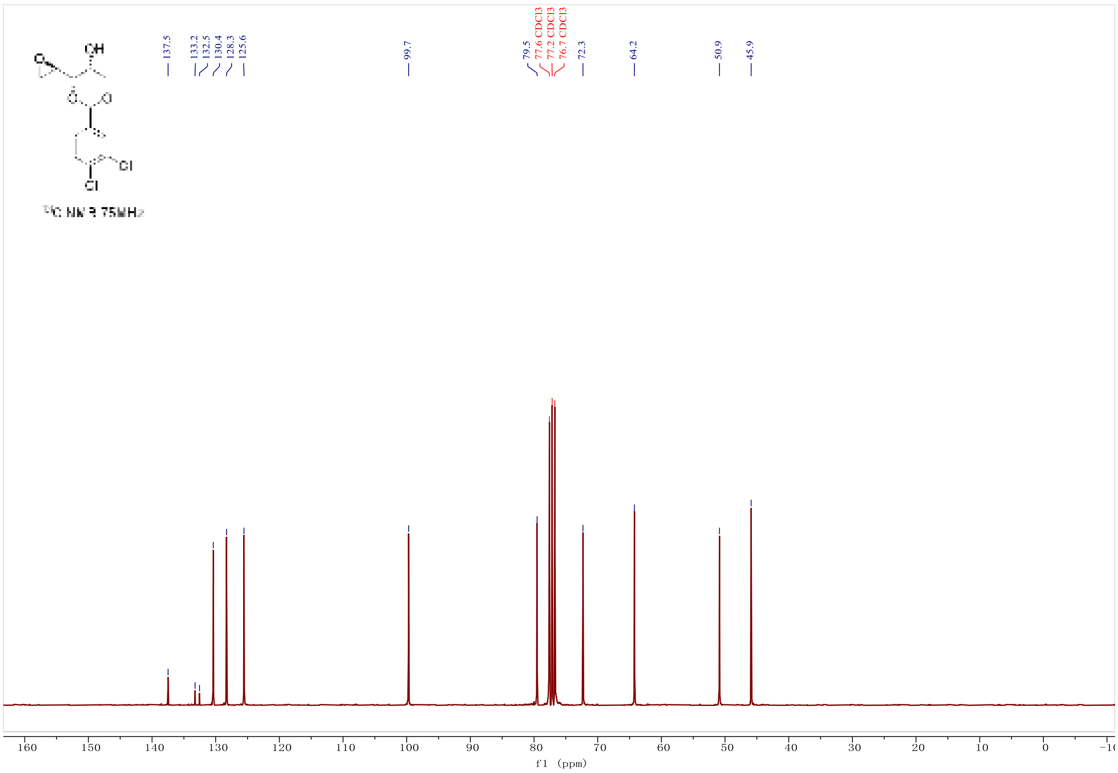

^{1}H NMR Spectrum of **14g**

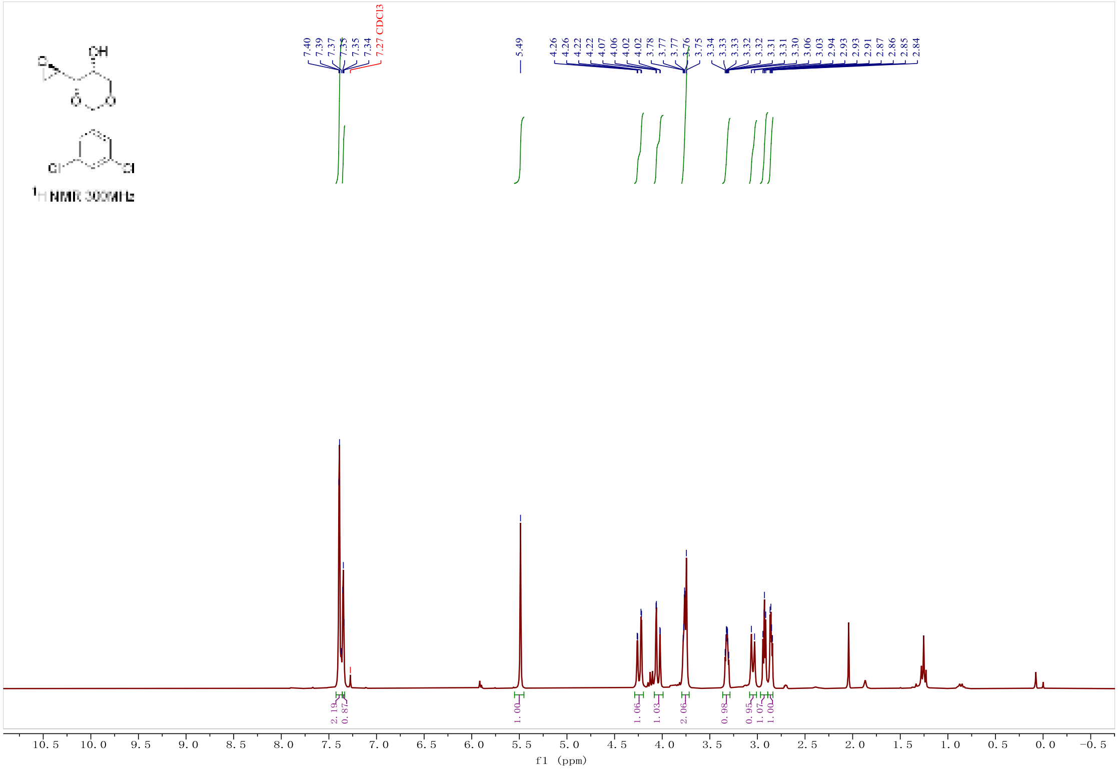

^{13}C NMR Spectrum of **14g**

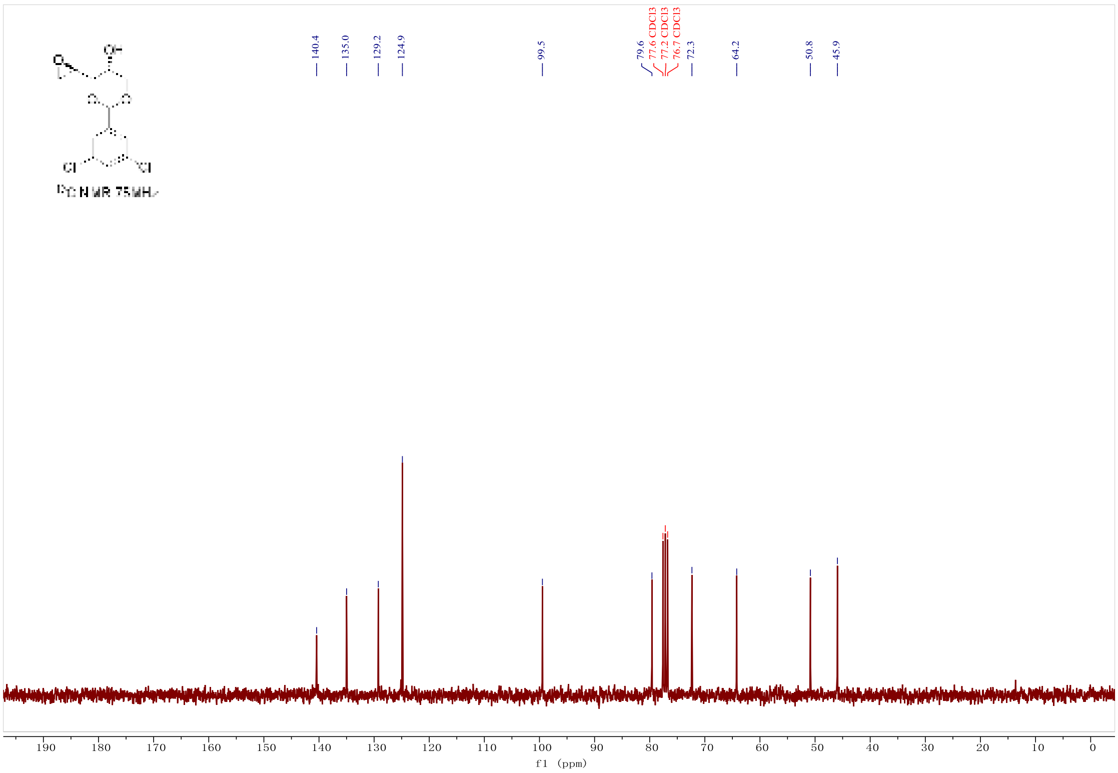

^{1}H NMR Spectrum of **14h**

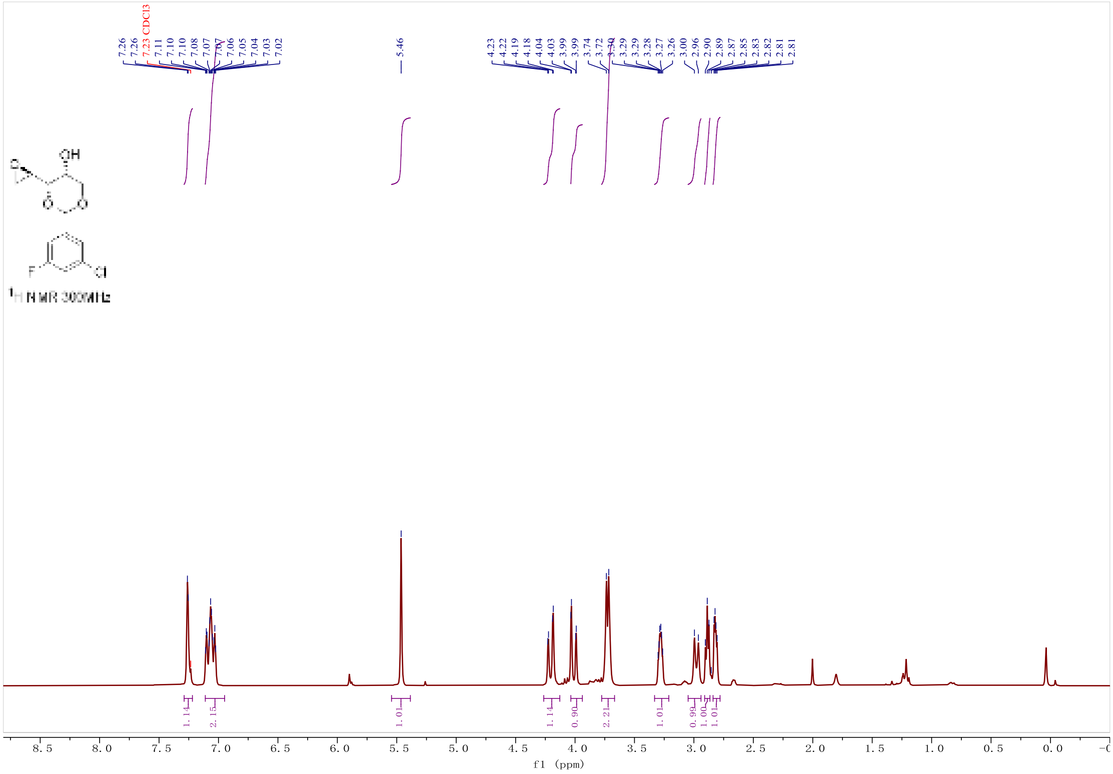

^{13}C NMR Spectrum of **14h**

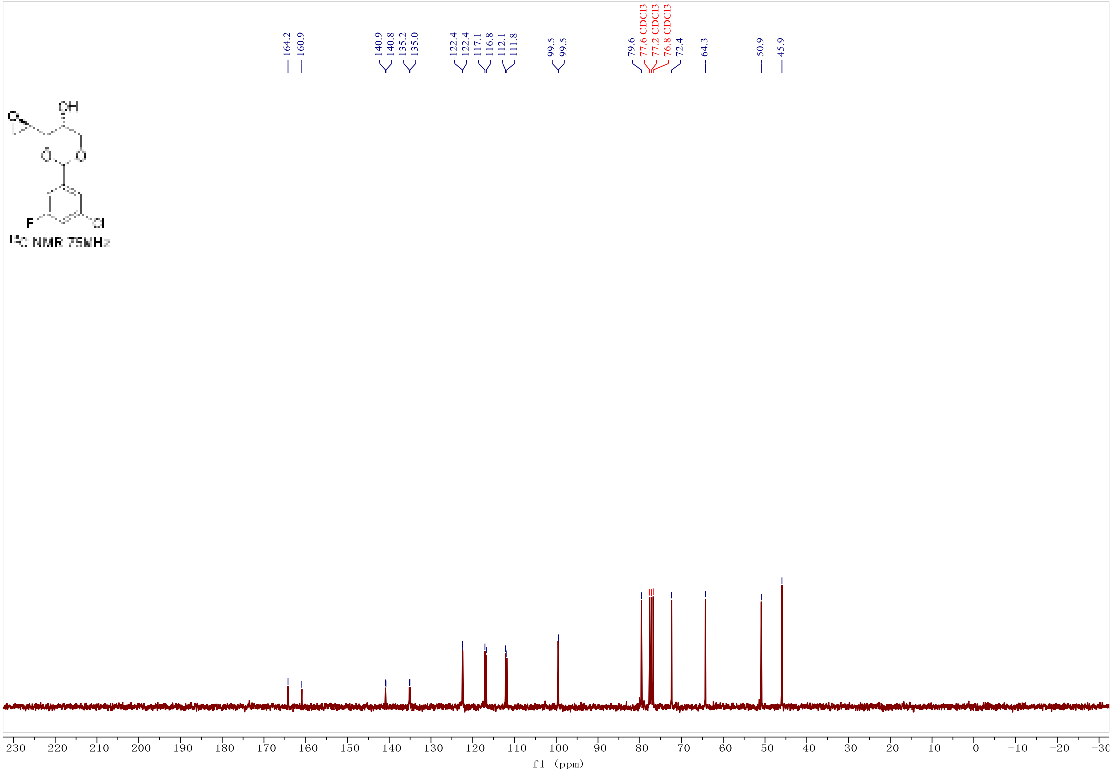

^{1}H NMR Spectrum of **14i**

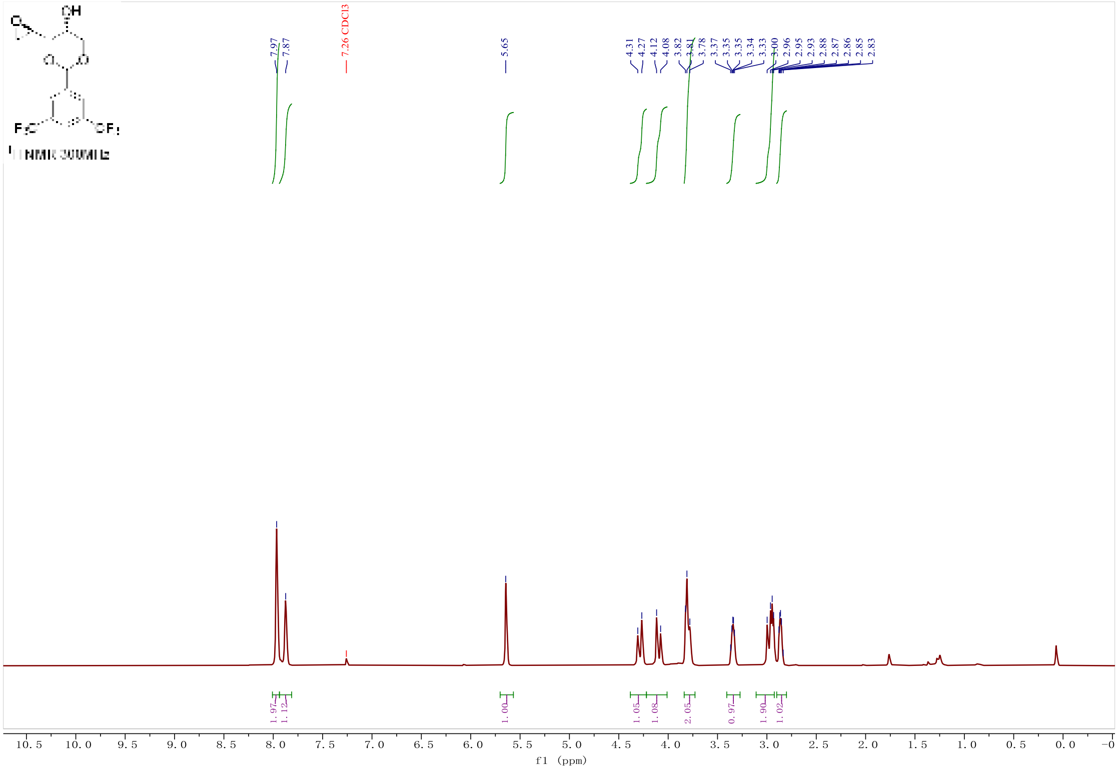

^{13}C NMR Spectrum of **14i**

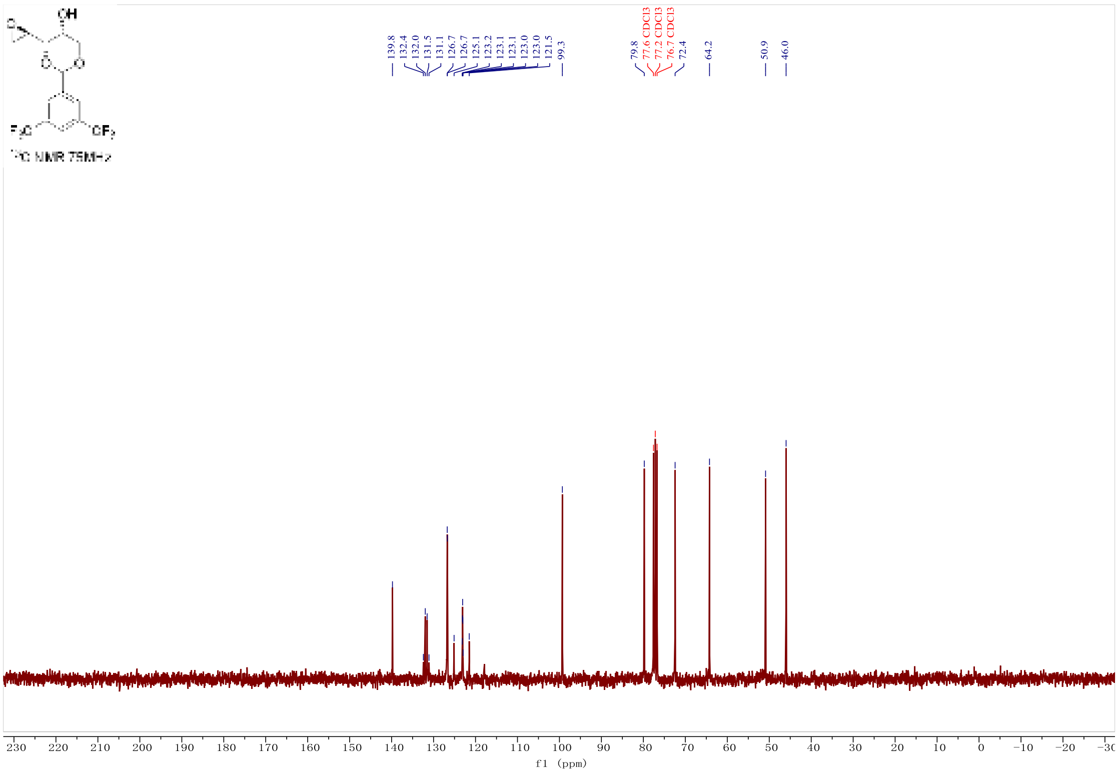

^{1}H NMR Spectrum of **14j**

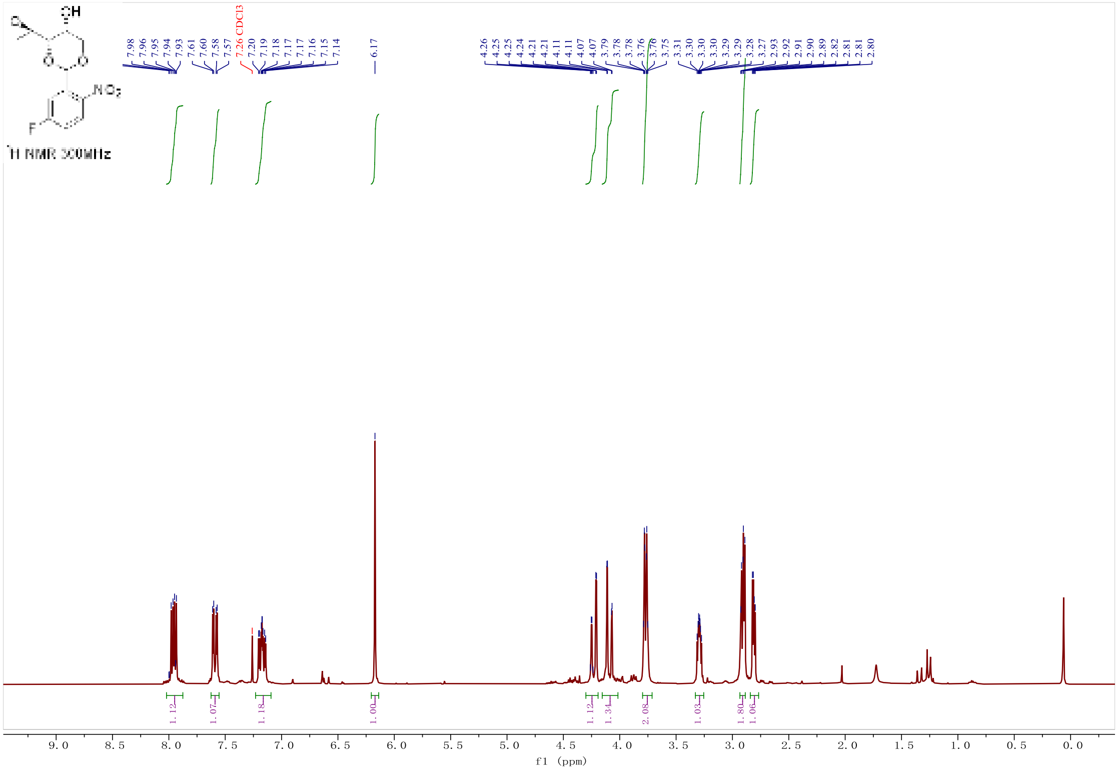

^{13}C NMR Spectrum of **14j**

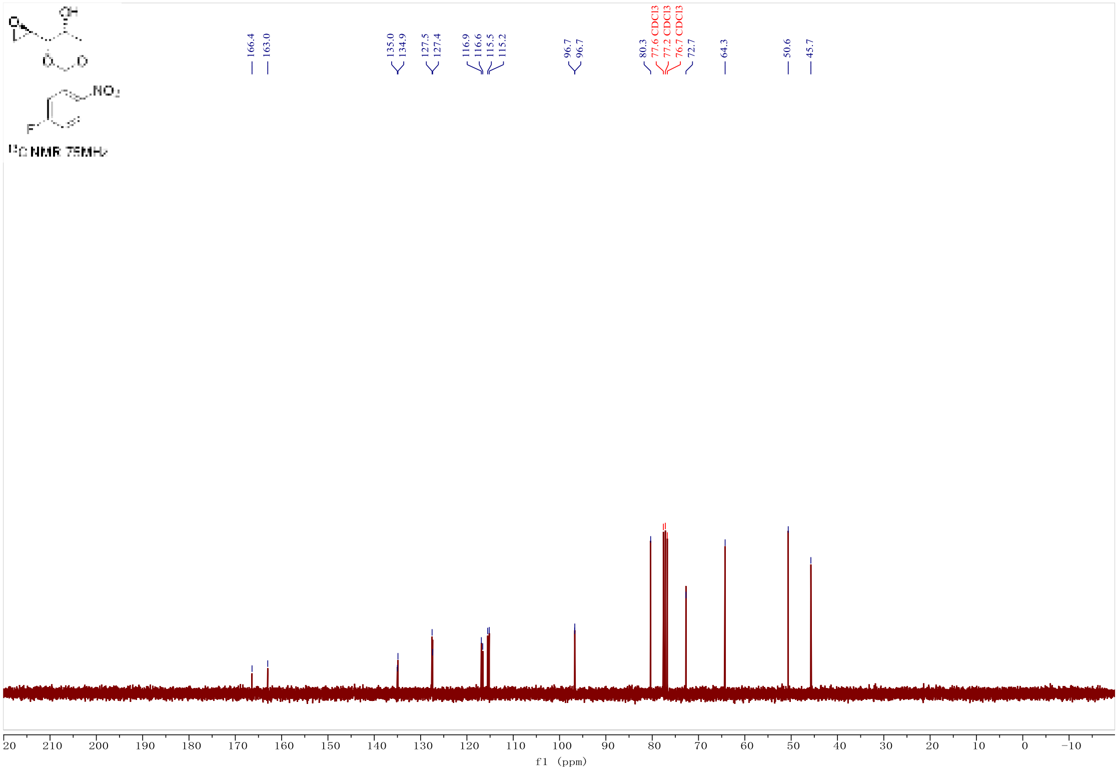

^{1}H NMR Spectrum of **14k**

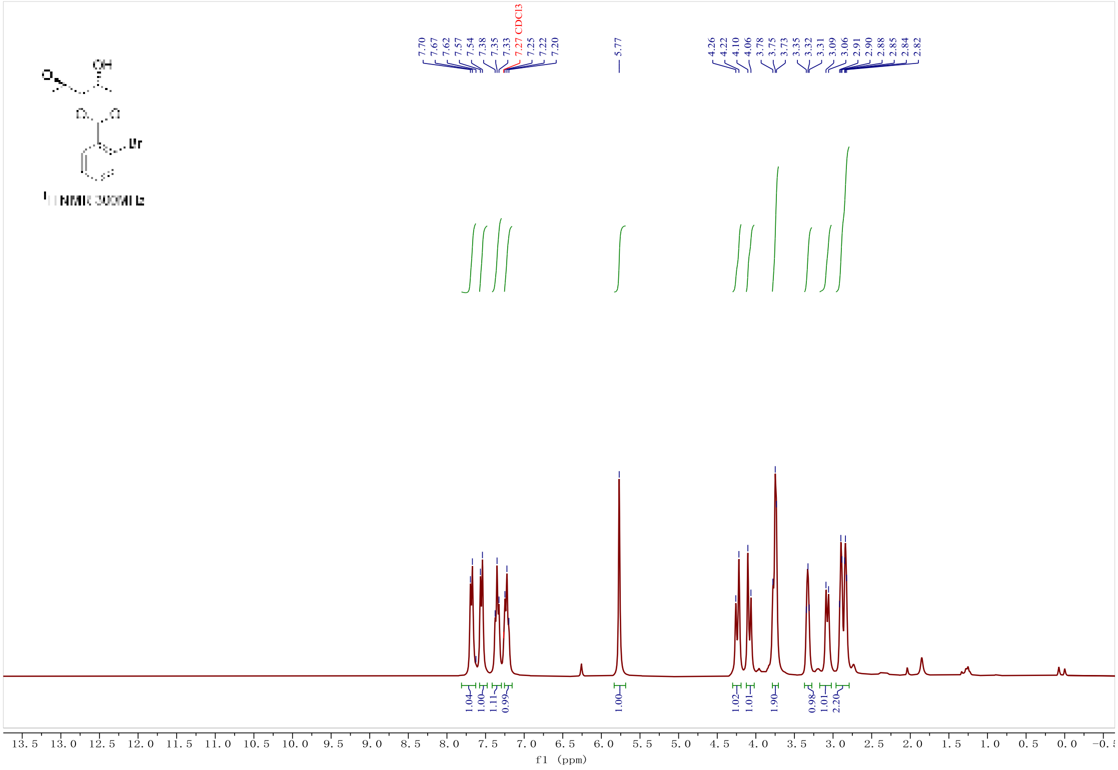

^{13}C NMR Spectrum of **14k**

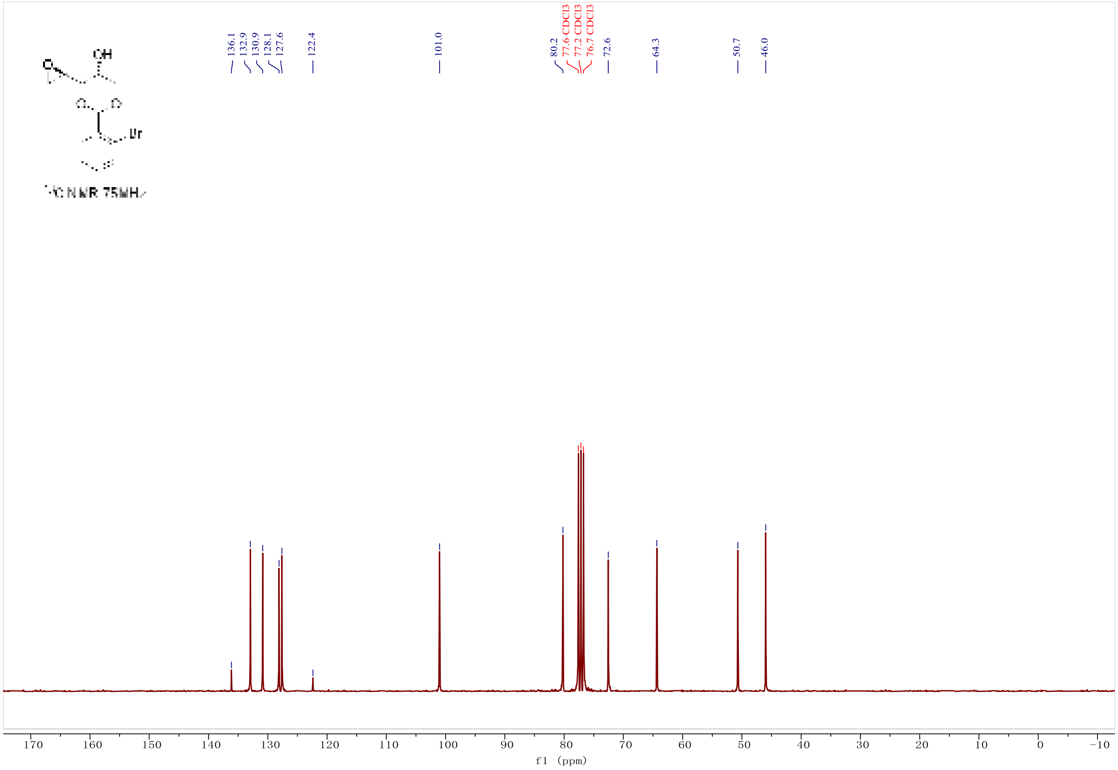

^{1}H NMR Spectrum of **14l**

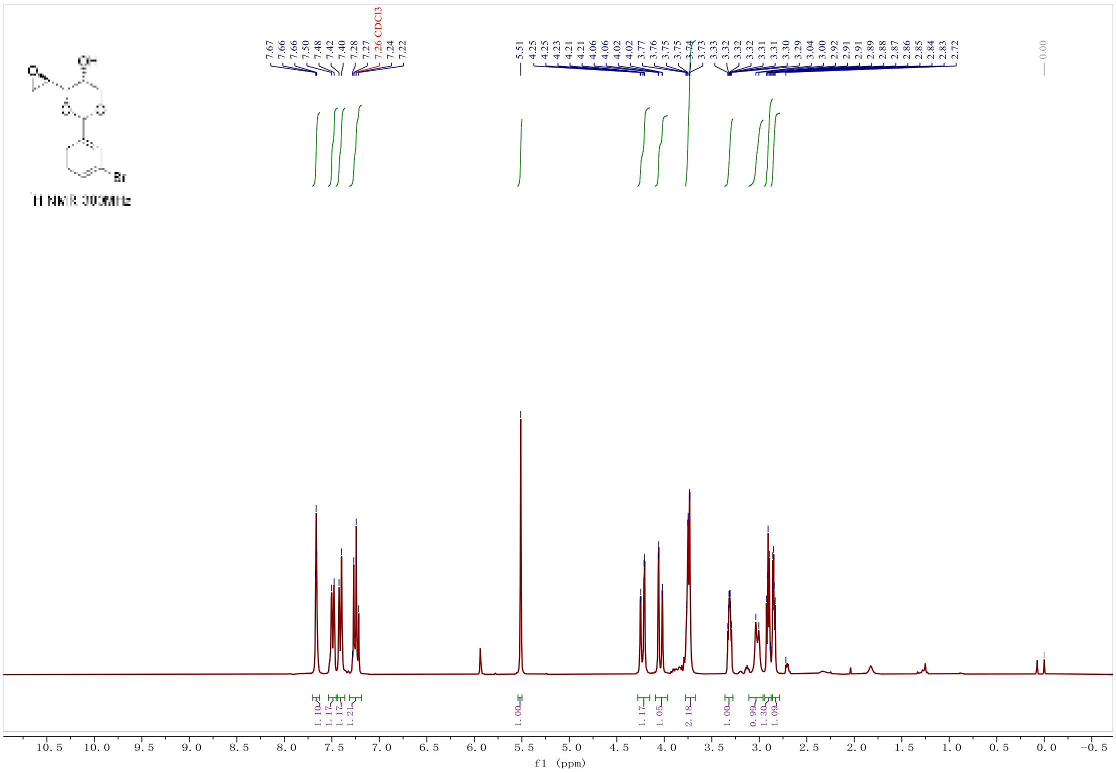

^{13}C NMR Spectrum of **14l**

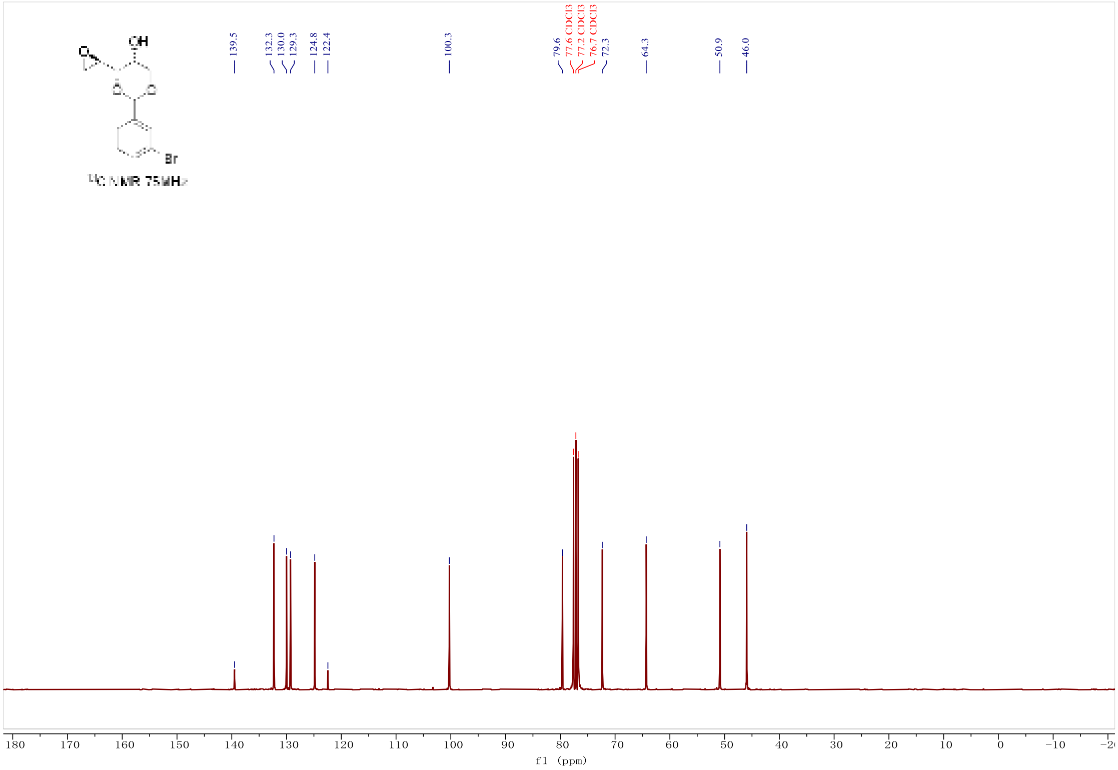

^{1}H NMR Spectrum of **14m**

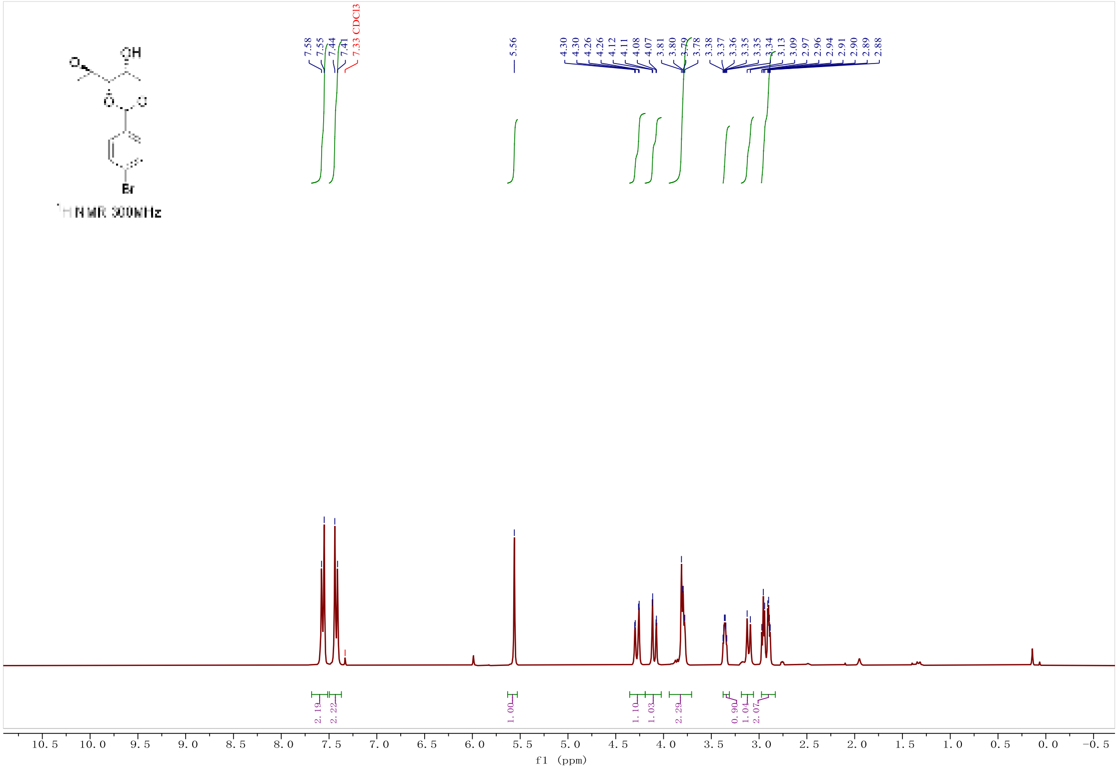

^{13}C NMR Spectrum of **14m**

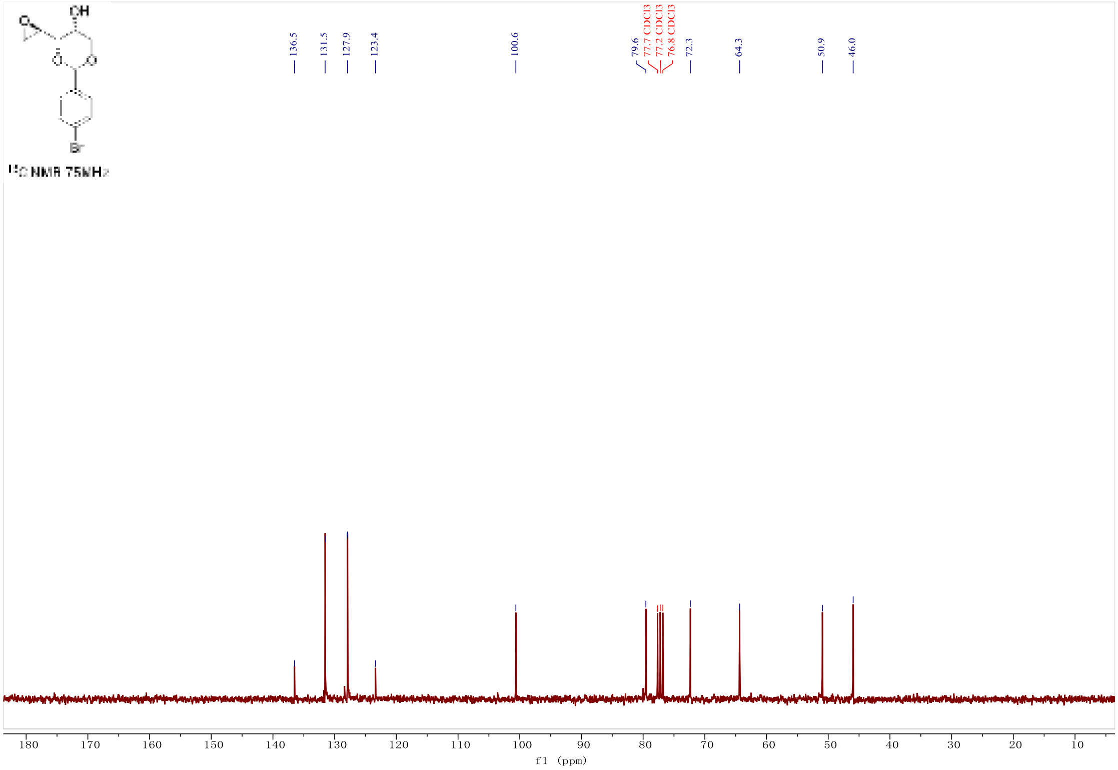

^{1}H-NMR spectrum of **14n**

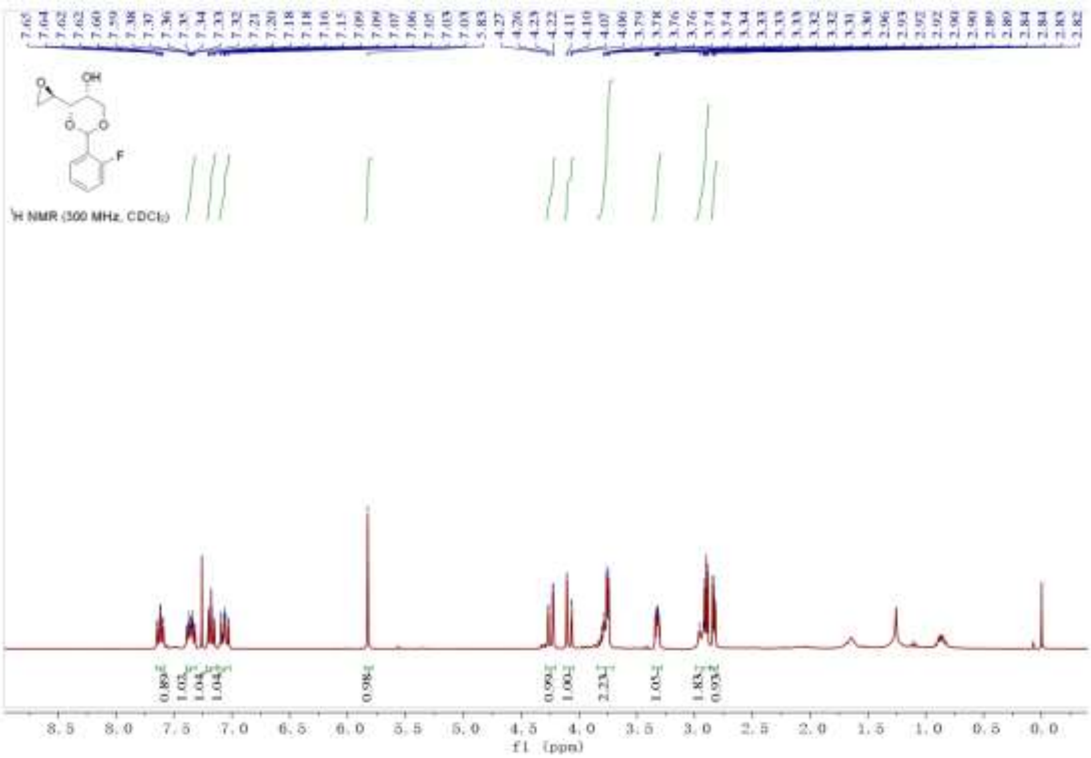

^{13}C-NMR spectrum of **14n**

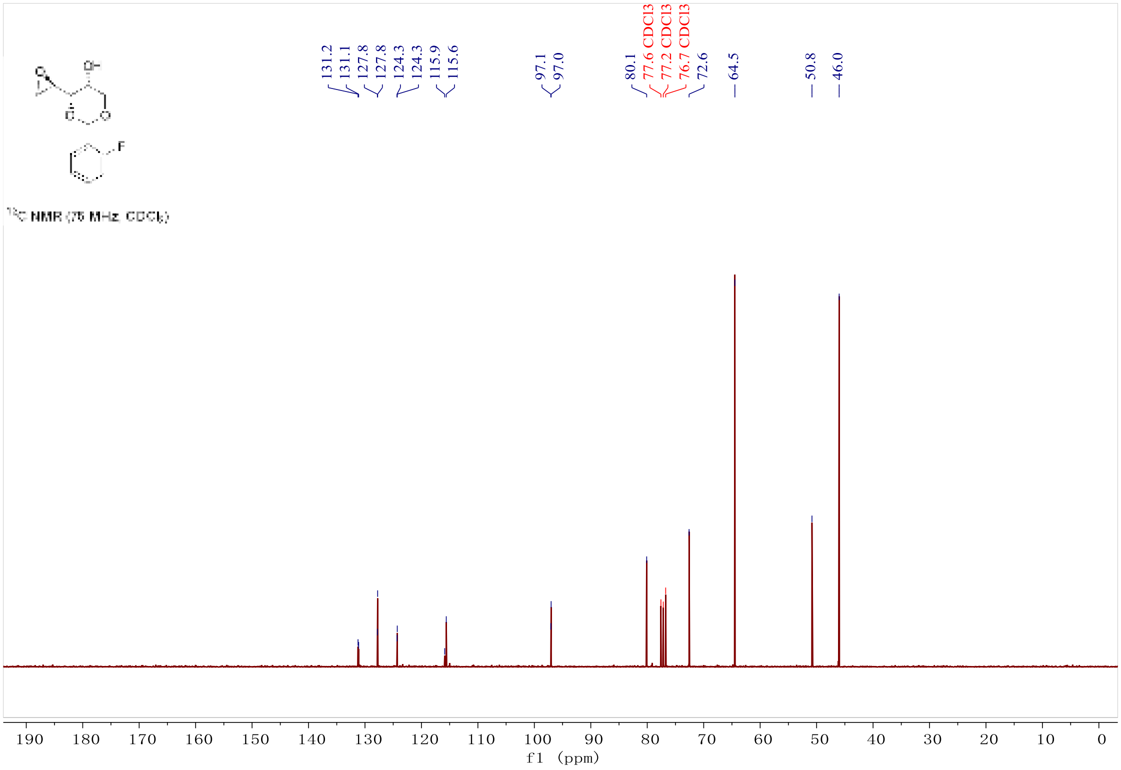

^{1}H-NMR spectrum of **14o**

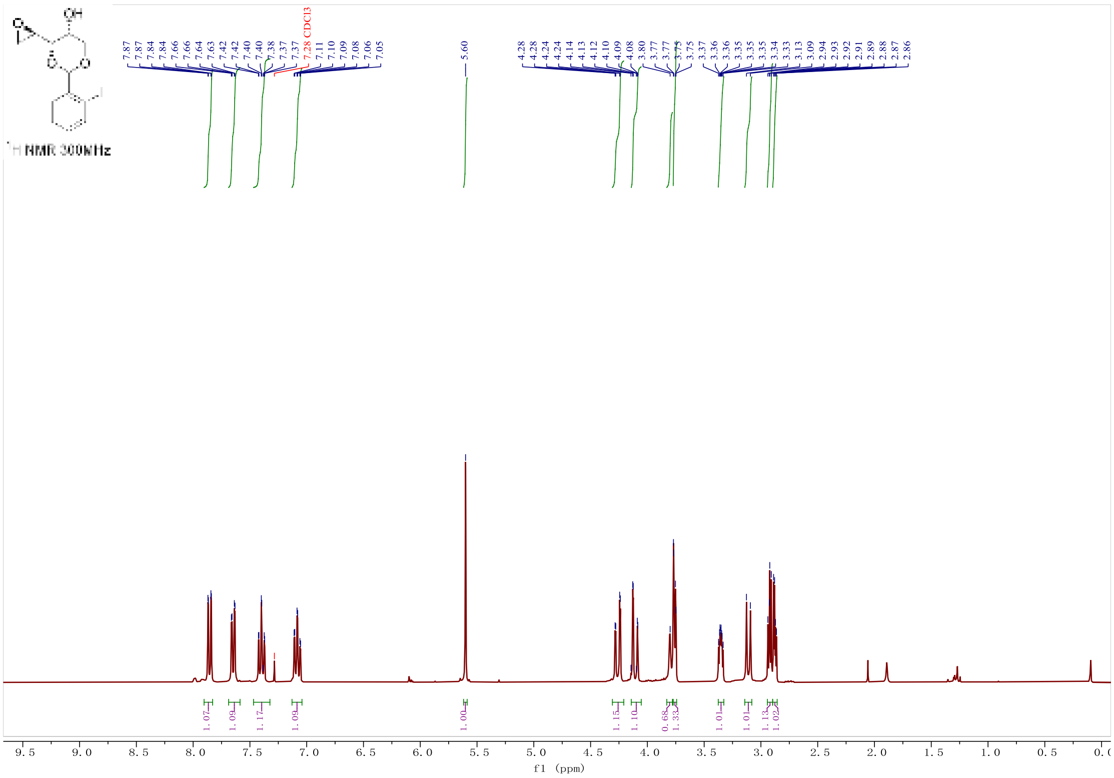

^{13}C-NMR spectrum of **14o**

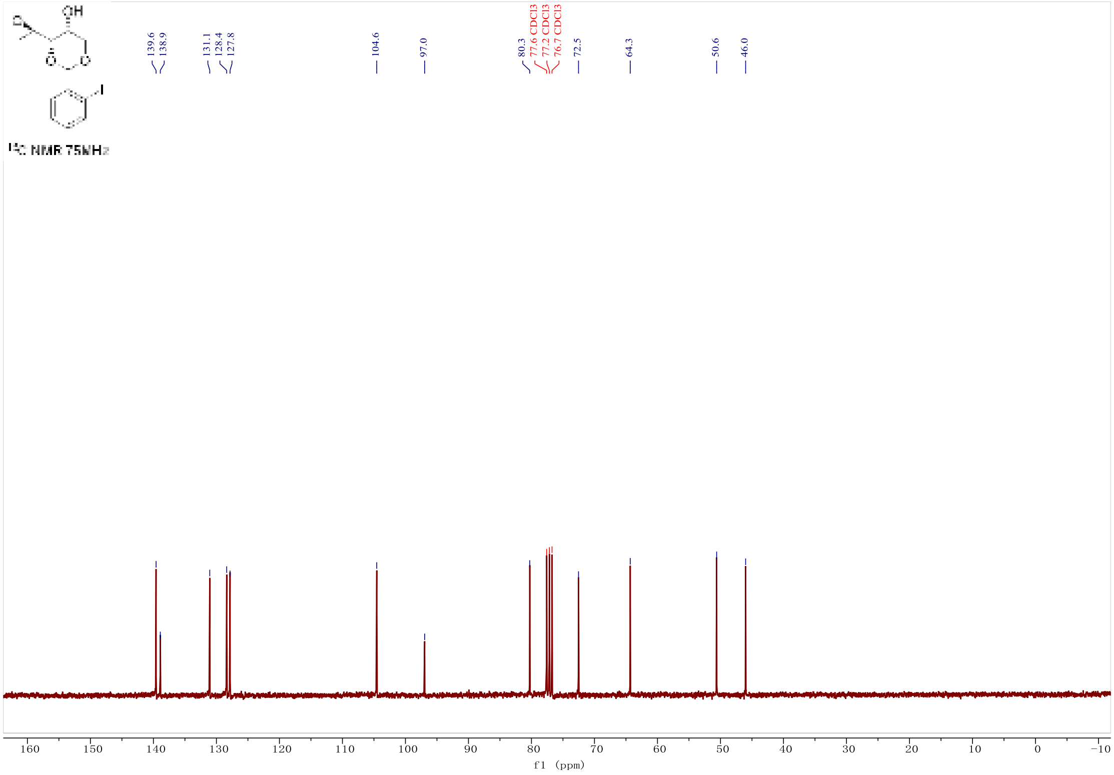

^{1}H NMR Spectrum of **14p**

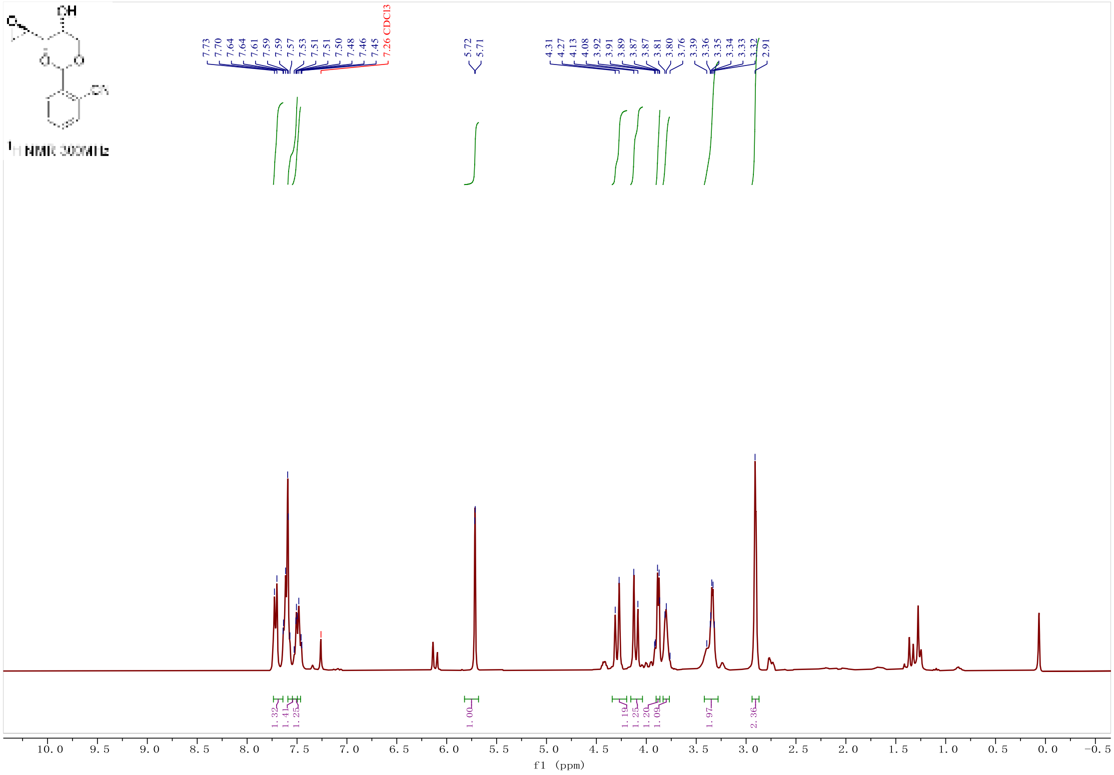

^{13}C NMR Spectrum of **14p**

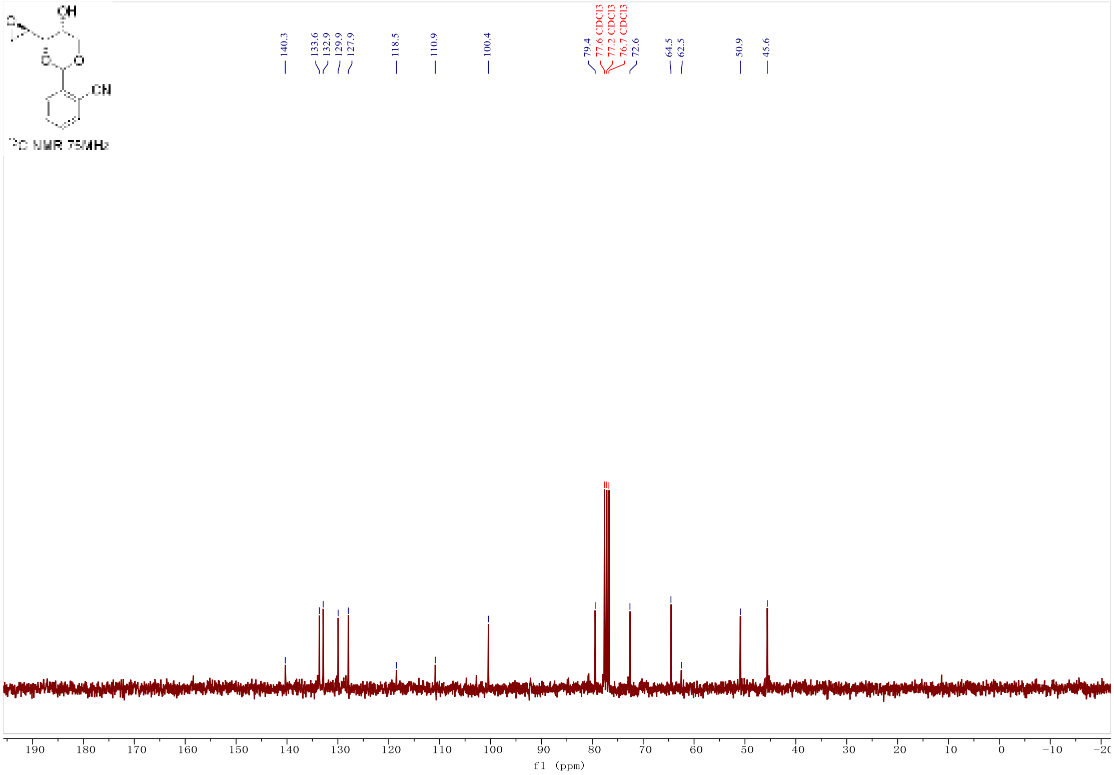

^{1}H-NMR spectrum of **14q**

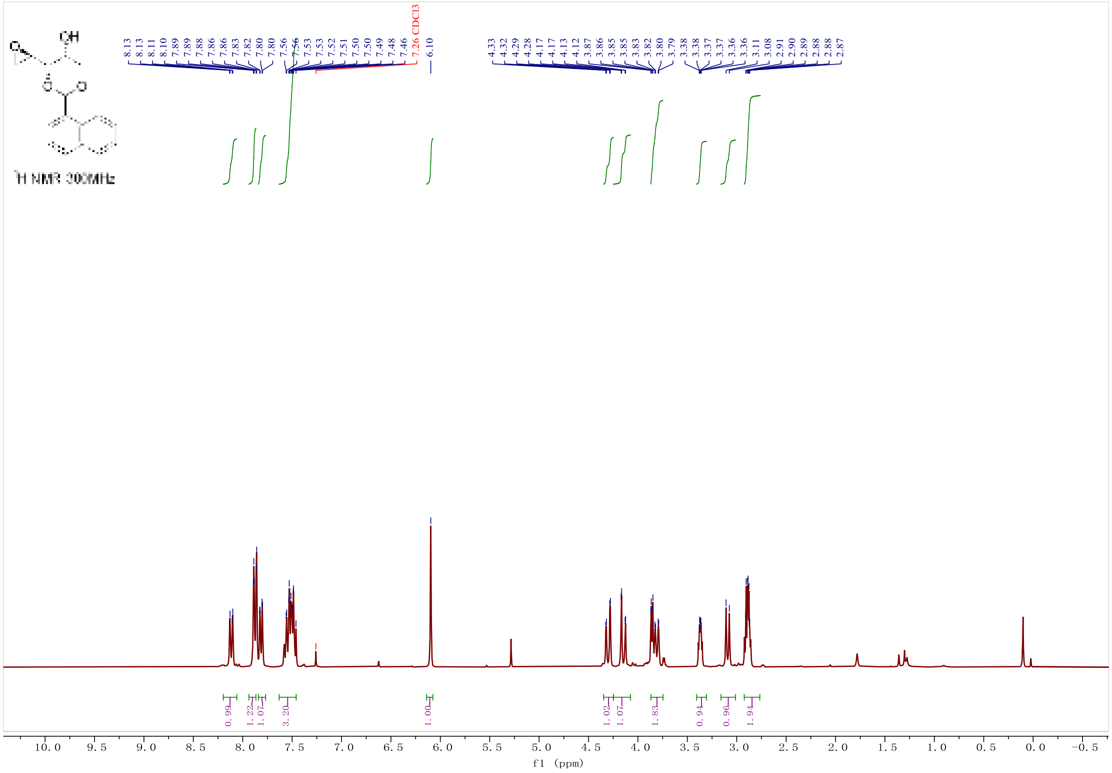

^{13}C-NMR spectrum of **14q**

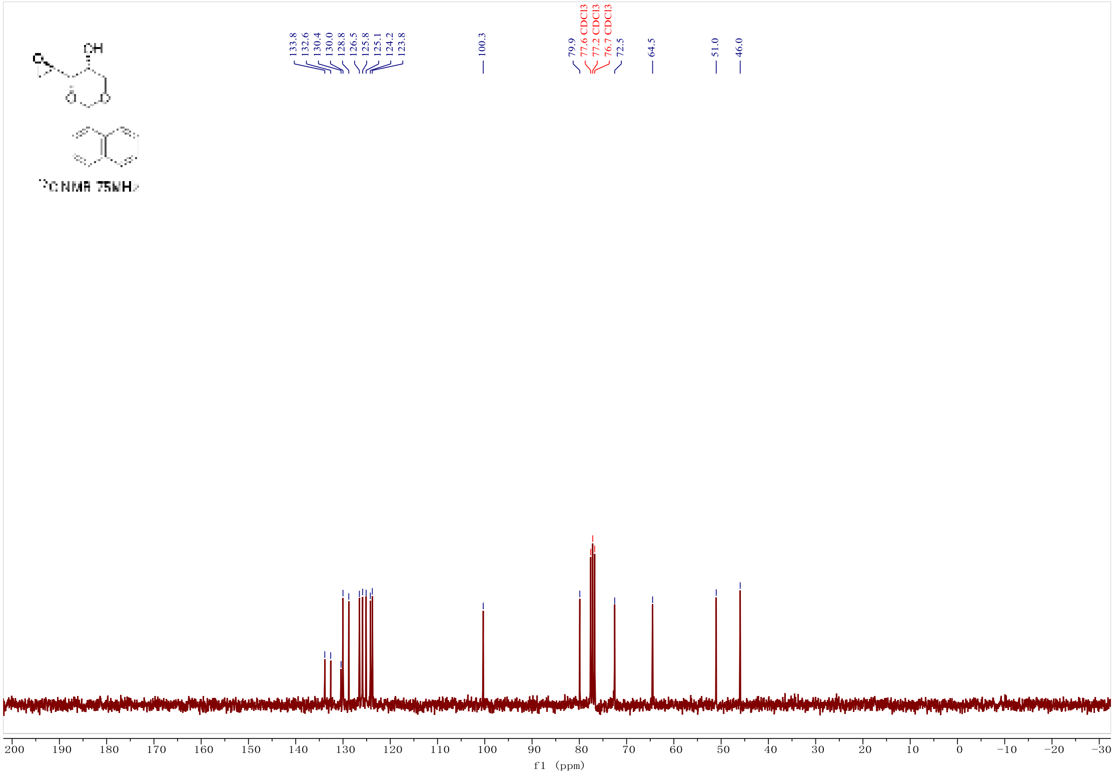

^{1}H-NMR spectrum of **14r**

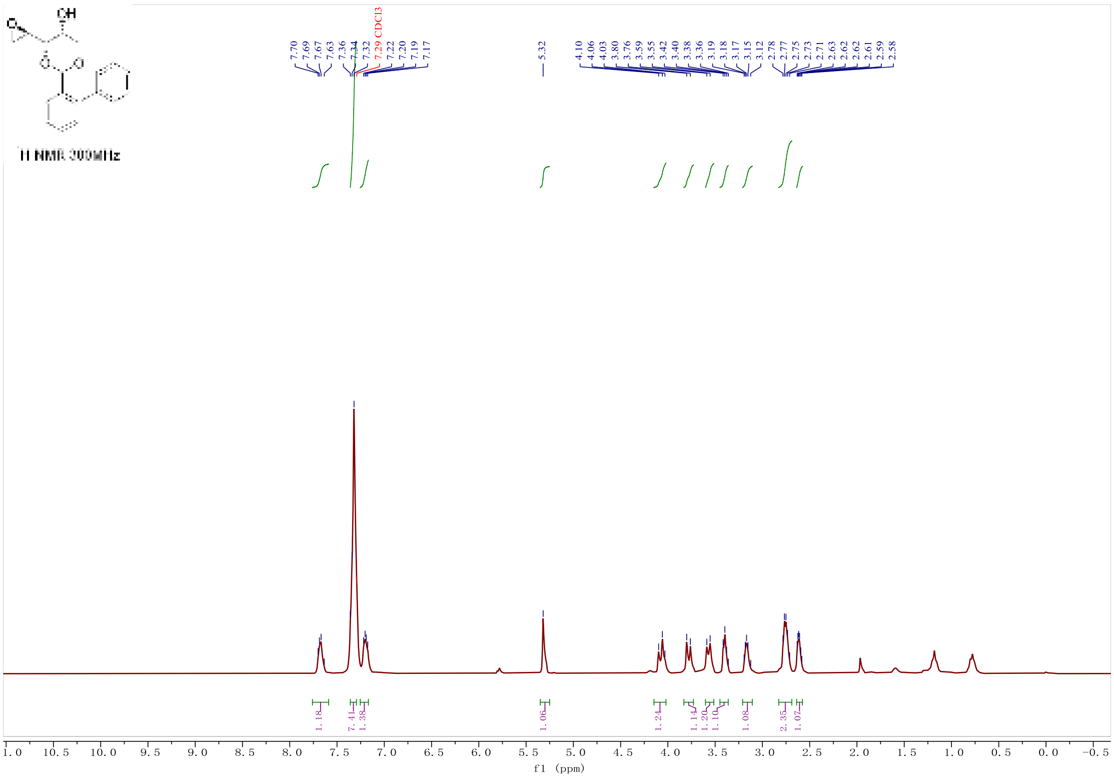

^{13}C-NMR spectrum of **14r**

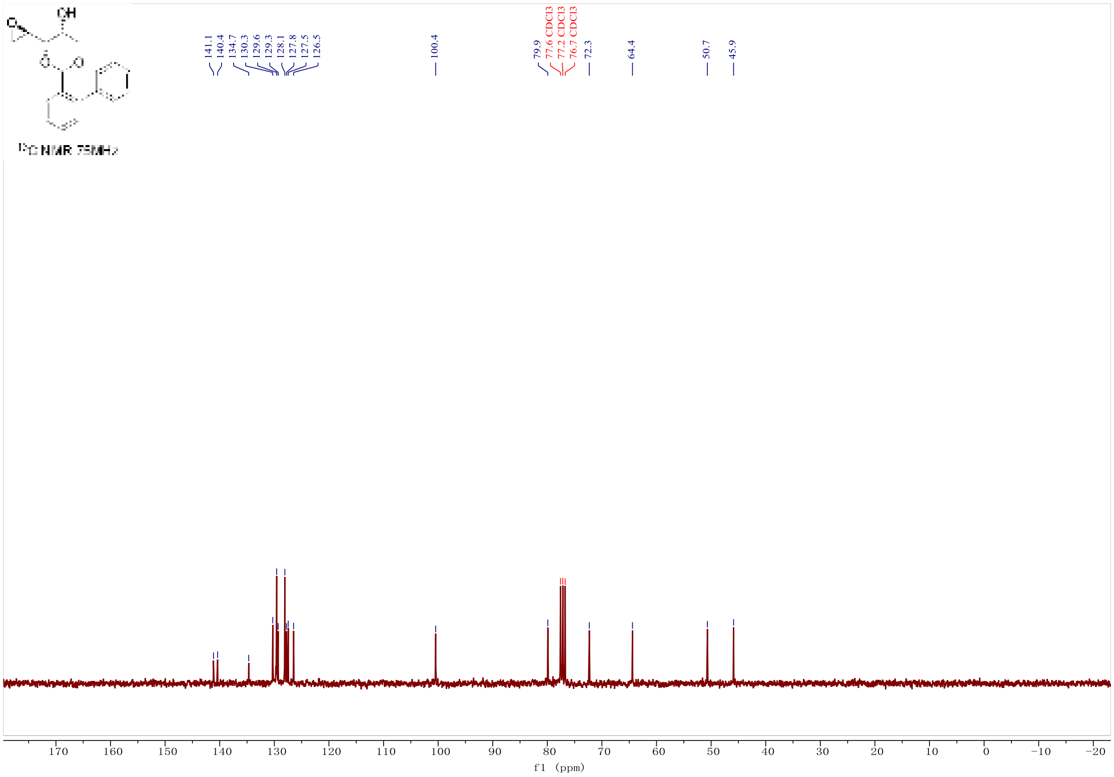

^{1}H-NMR spectrum of **19a**

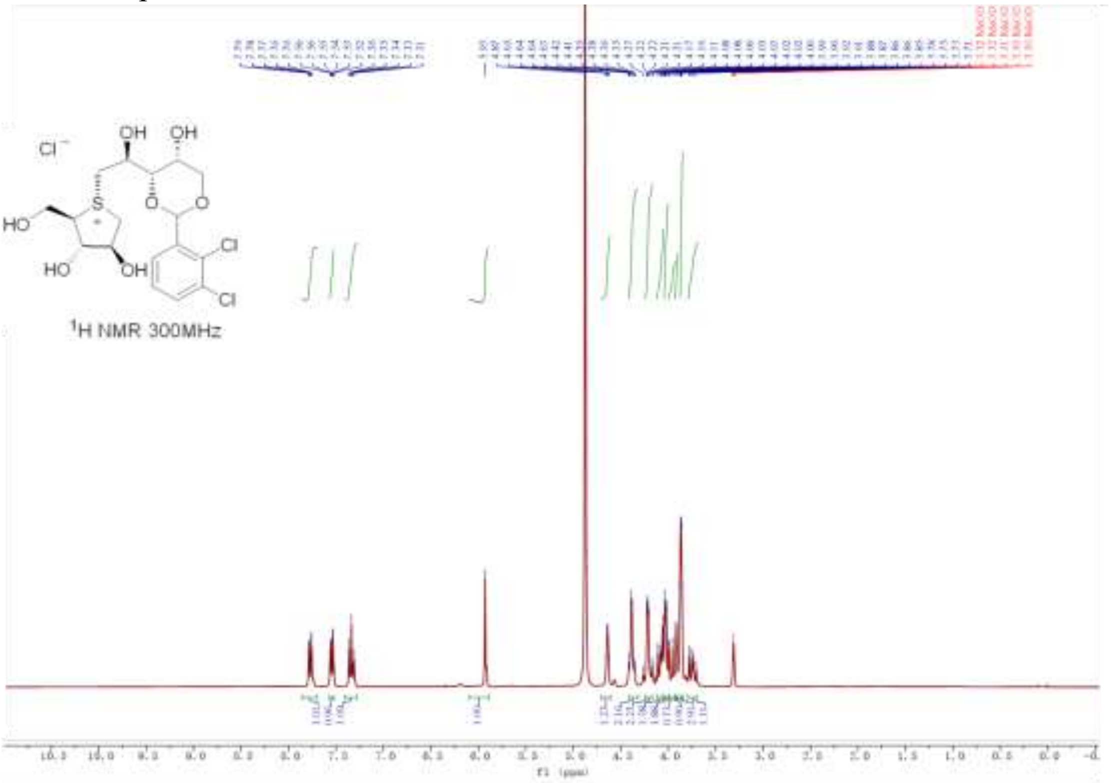

^{13}C-NMR spectrum of **19a**

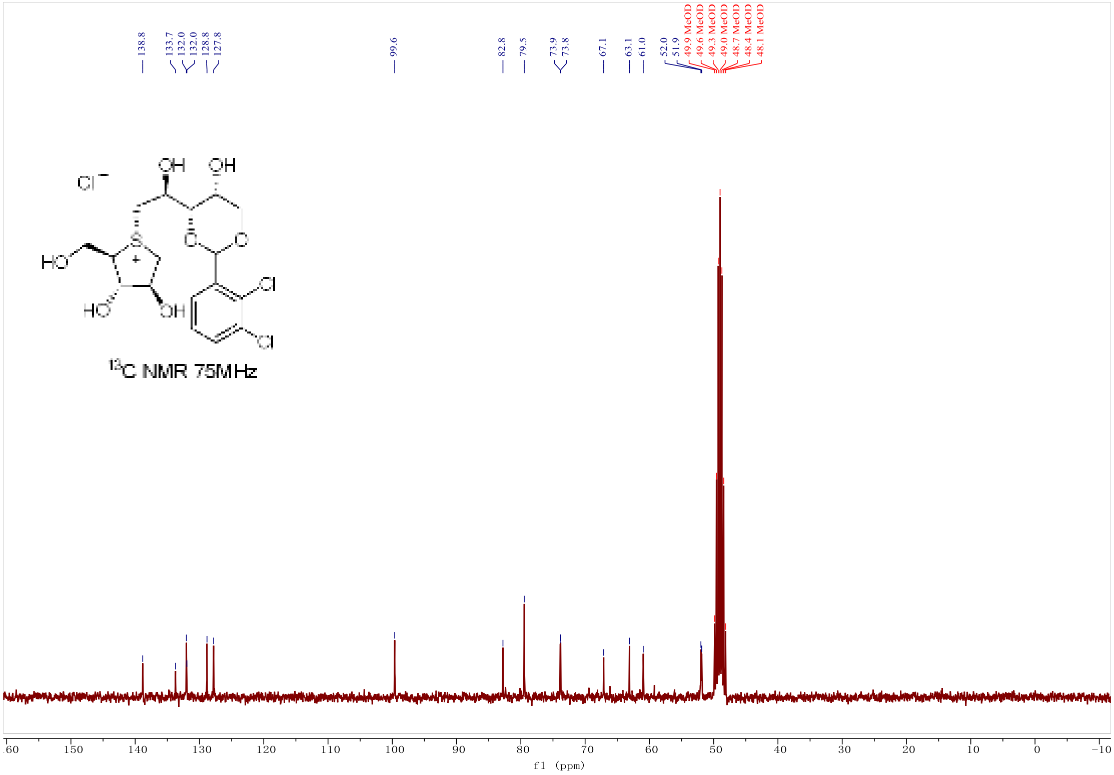

^{1}H-NMR spectrum of **19b**

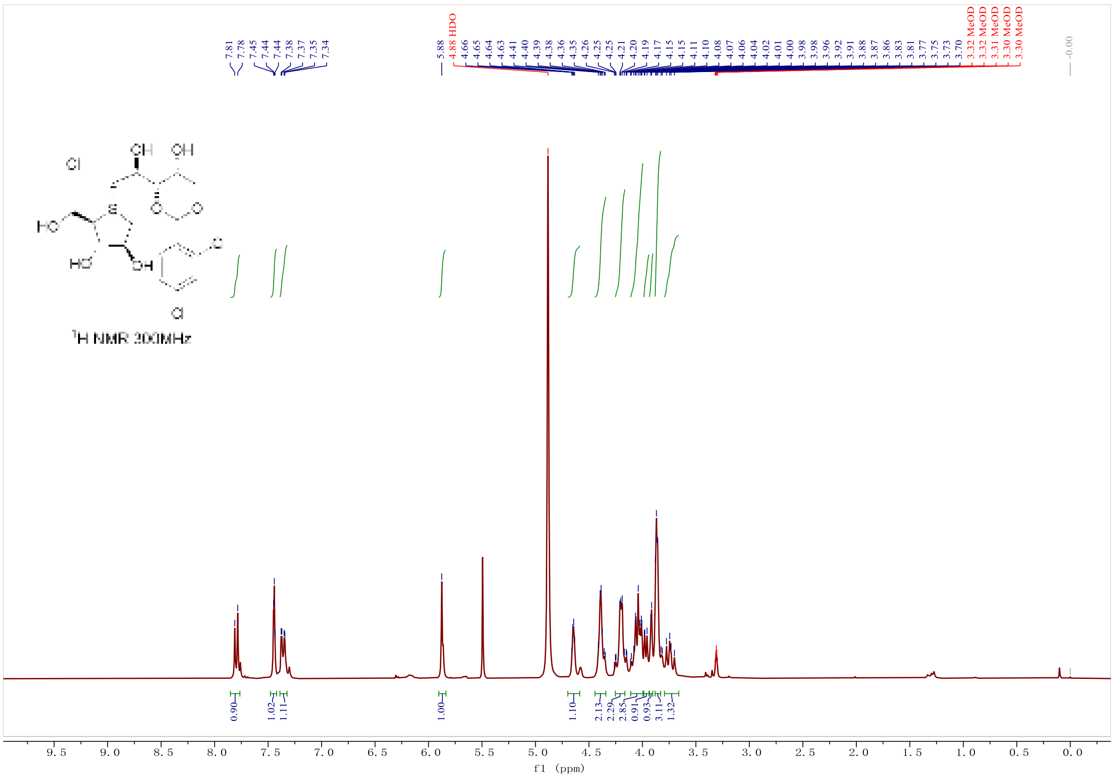

^{13}C-NMR spectrum of **19b**

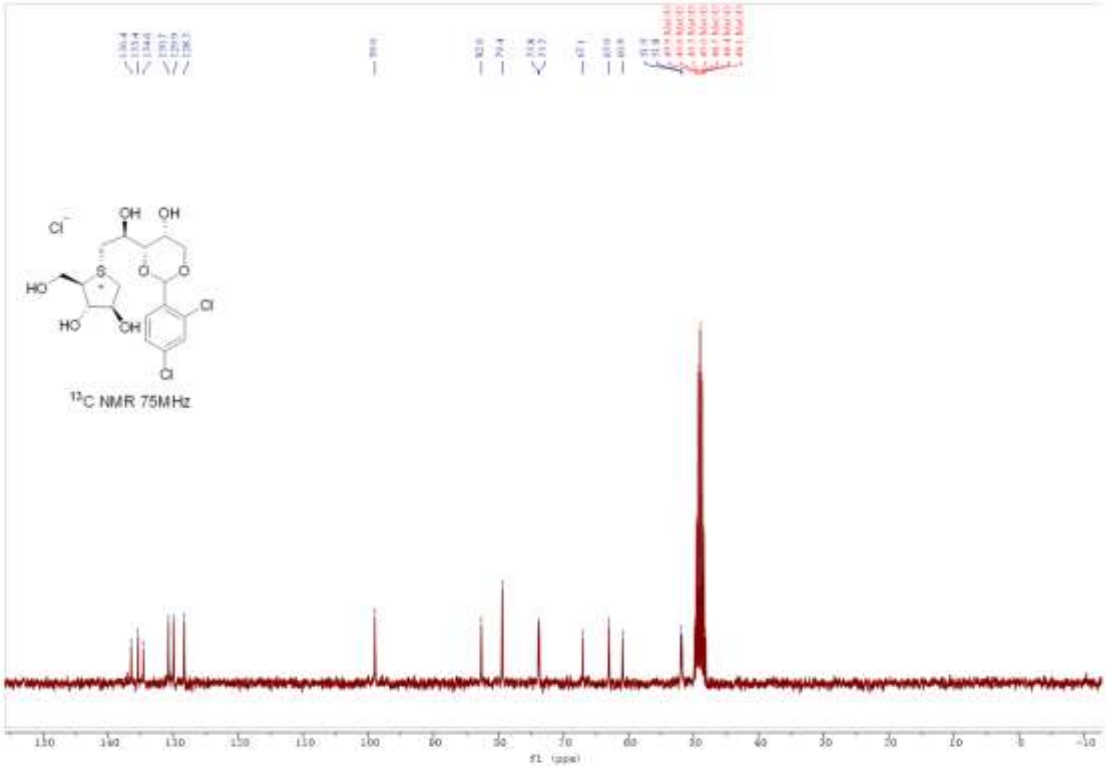

^{1}H-NMR spectrum of **19c**

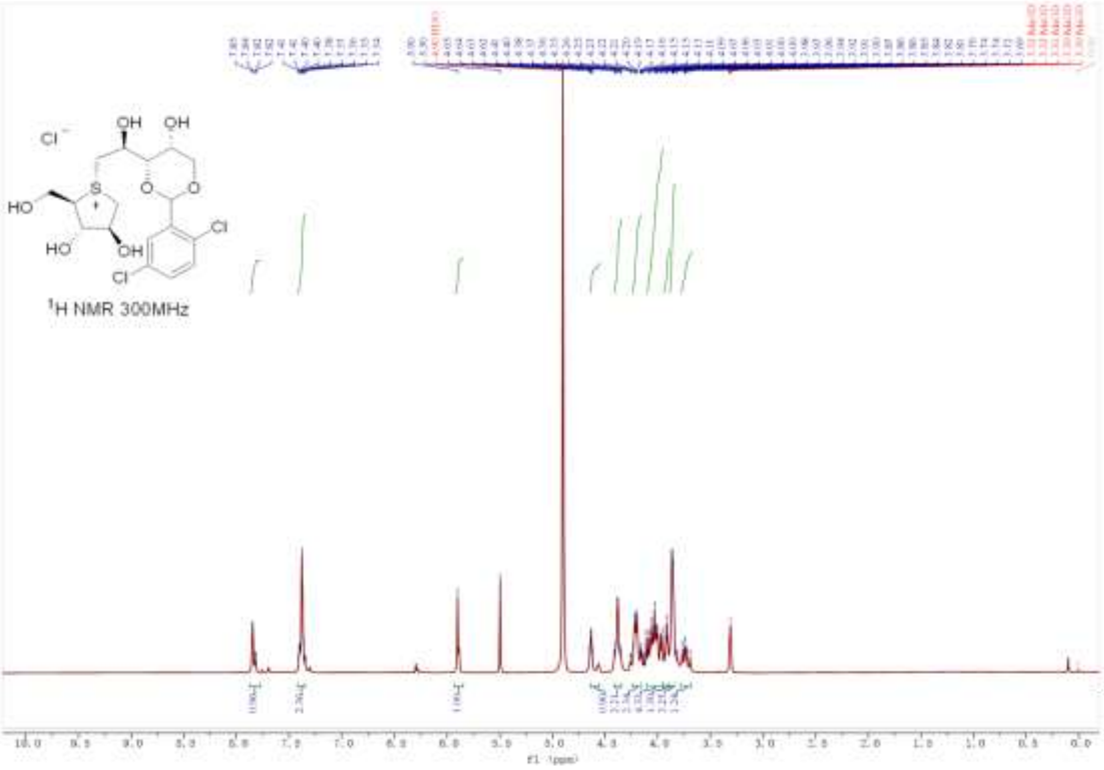

^{13}C-NMR spectrum of **19c**

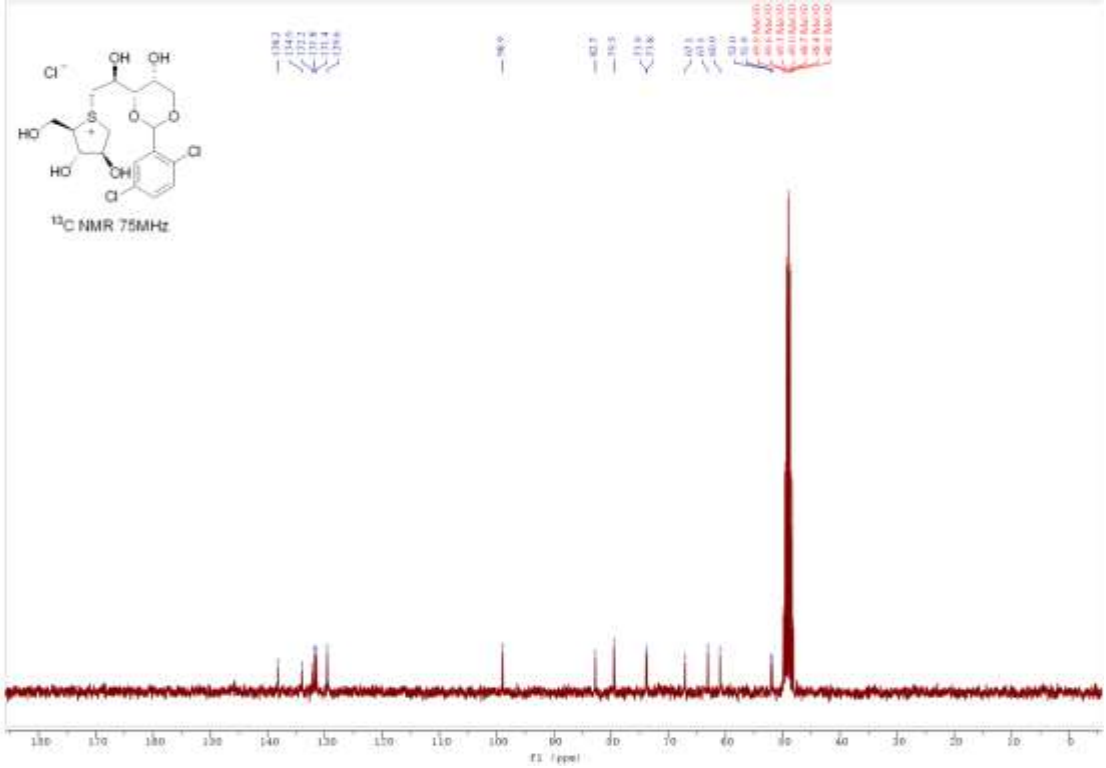

^{1}H-NMR spectrum of **19d**

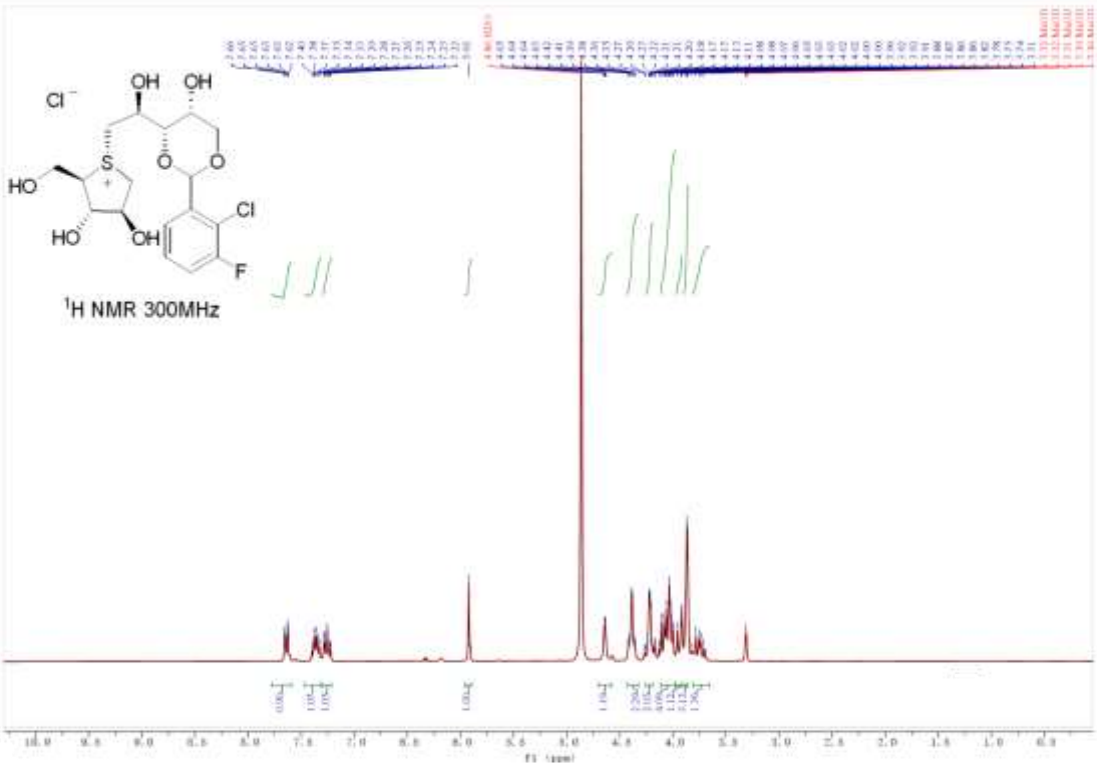

^{13}C-NMR spectrum of **19d**

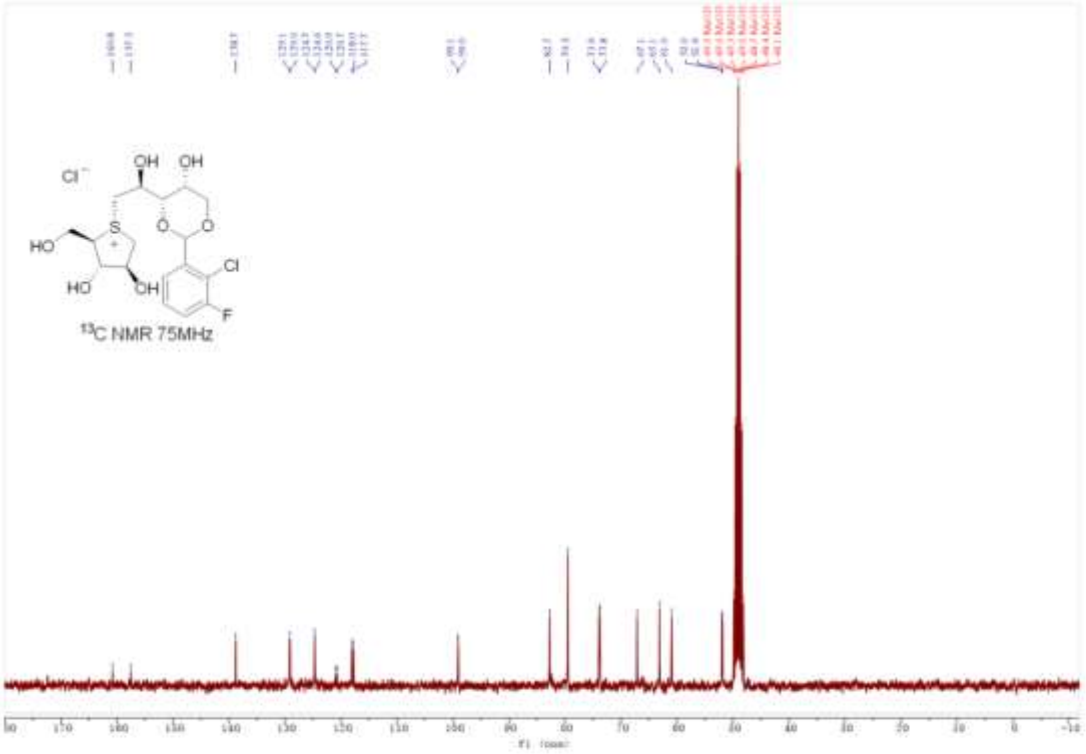

^{1}H-NMR spectrum of **19e**

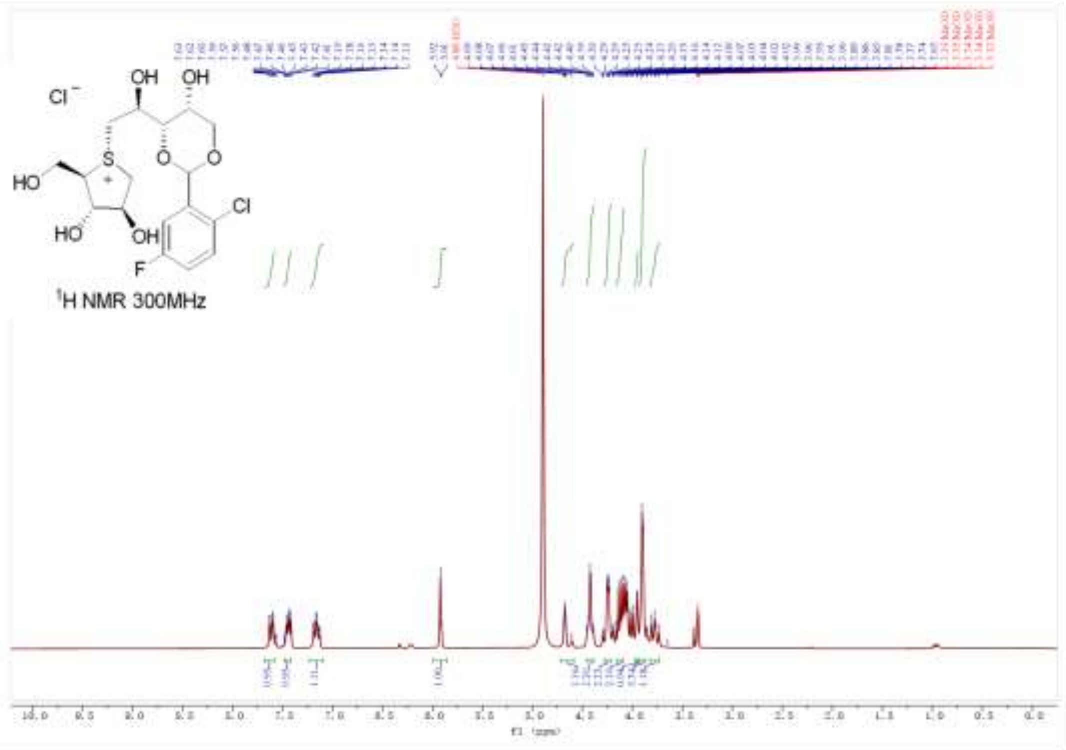

^{13}C-NMR spectrum of **19e**

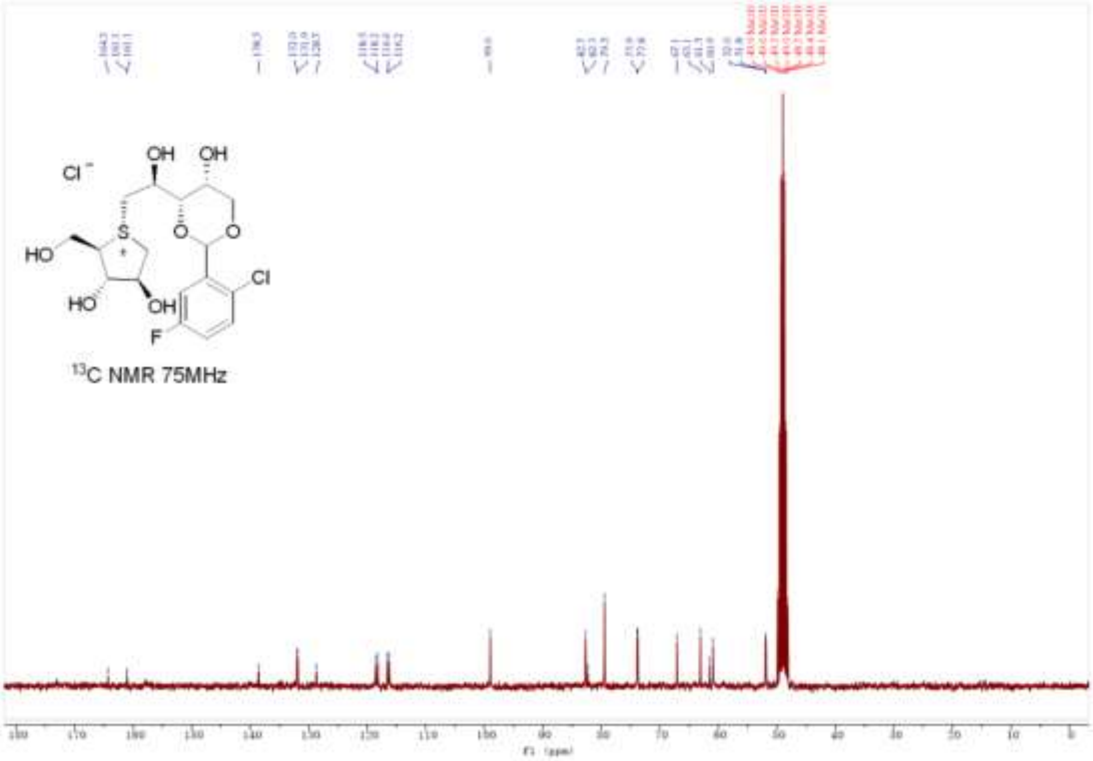

^{1}H-NMR spectrum of **19f**

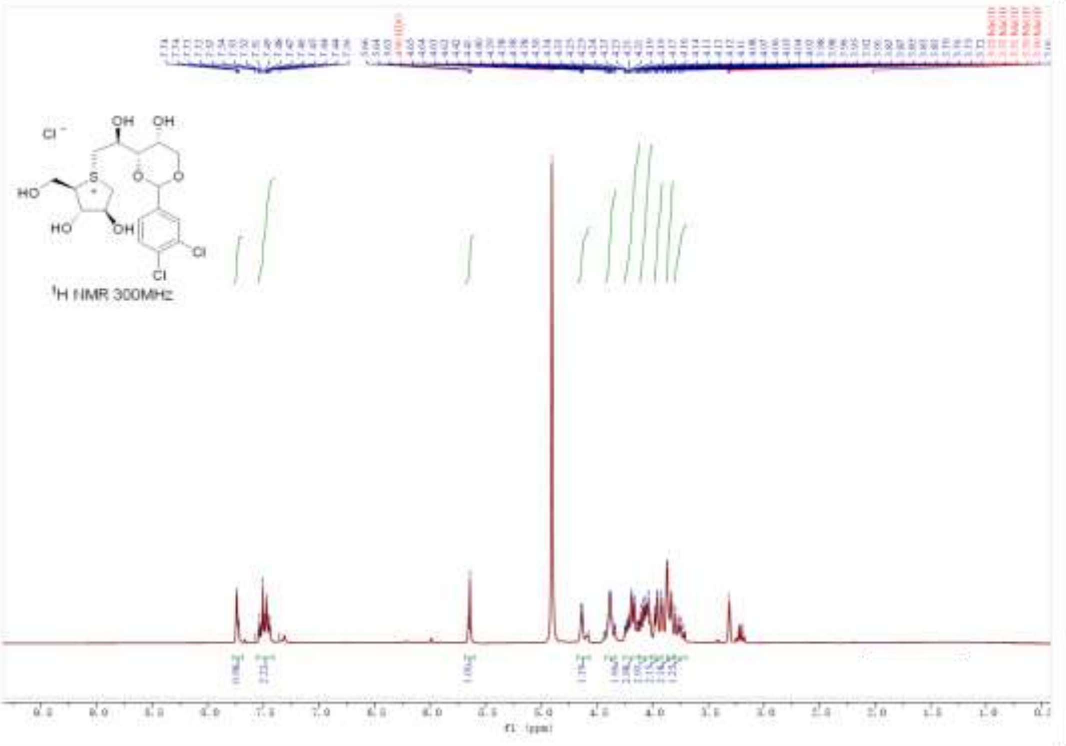

^{13}C-NMR spectrum of **19f**

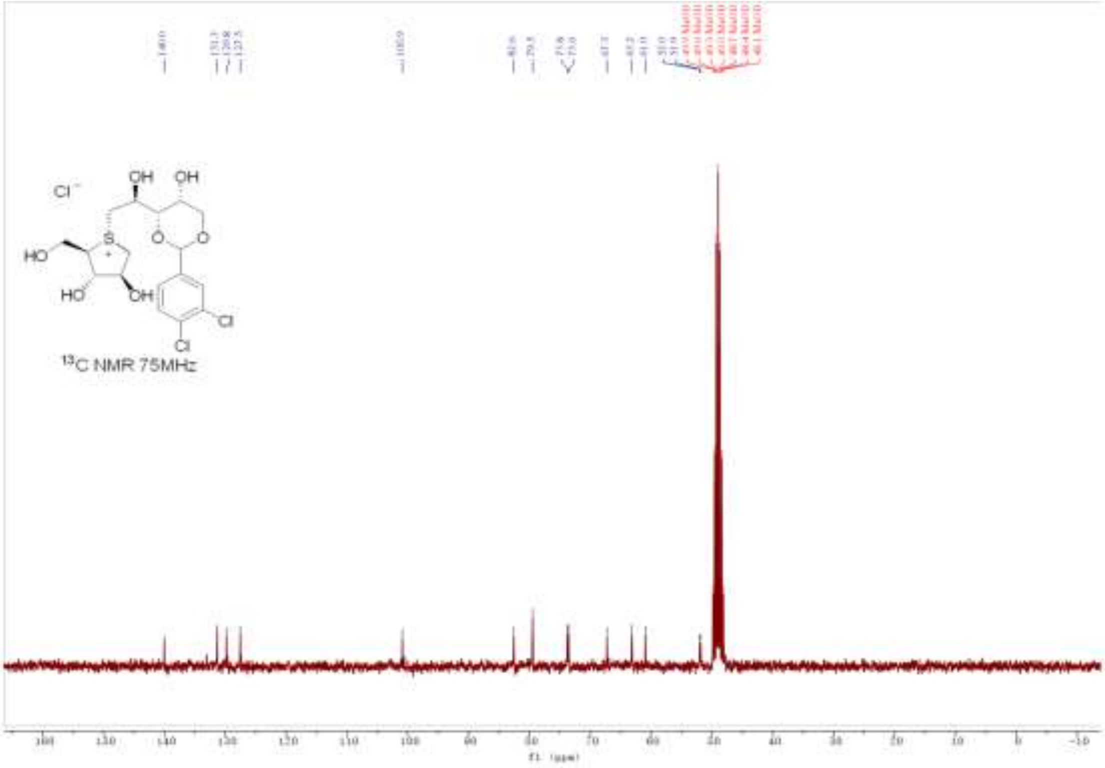

^{1}H-NMR spectrum of **19g**

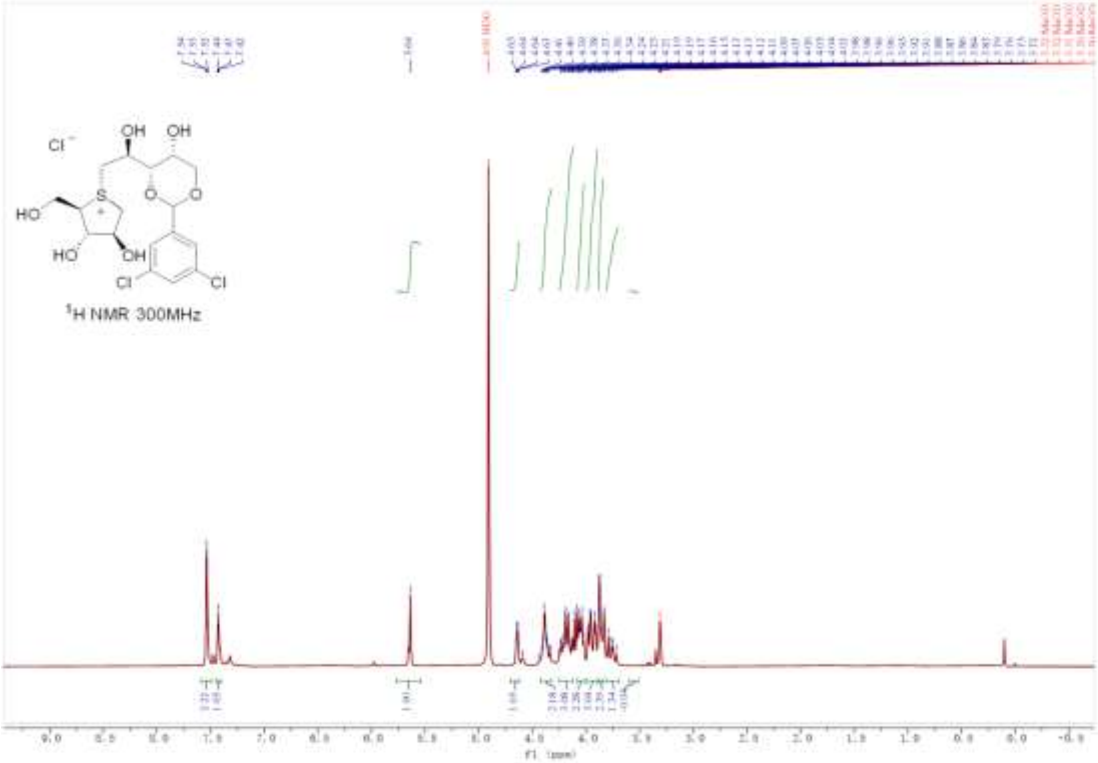

^{13}C-NMR spectrum of **19g**

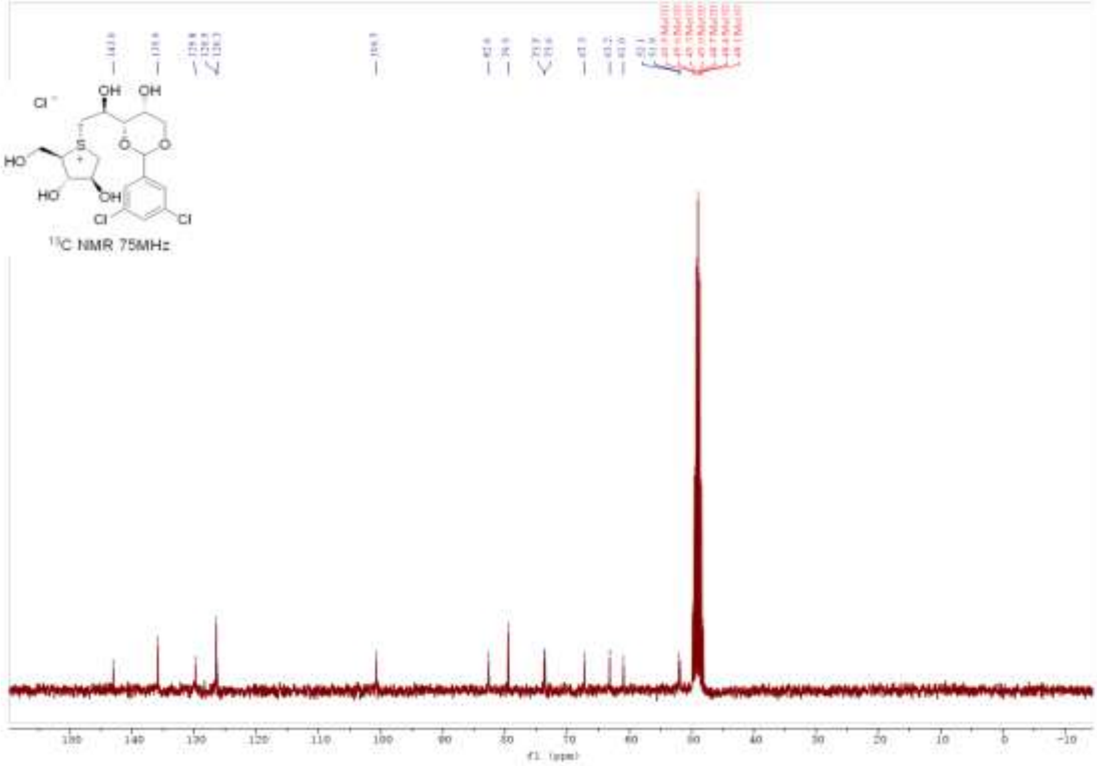

^{1}H-NMR spectrum of **19h**

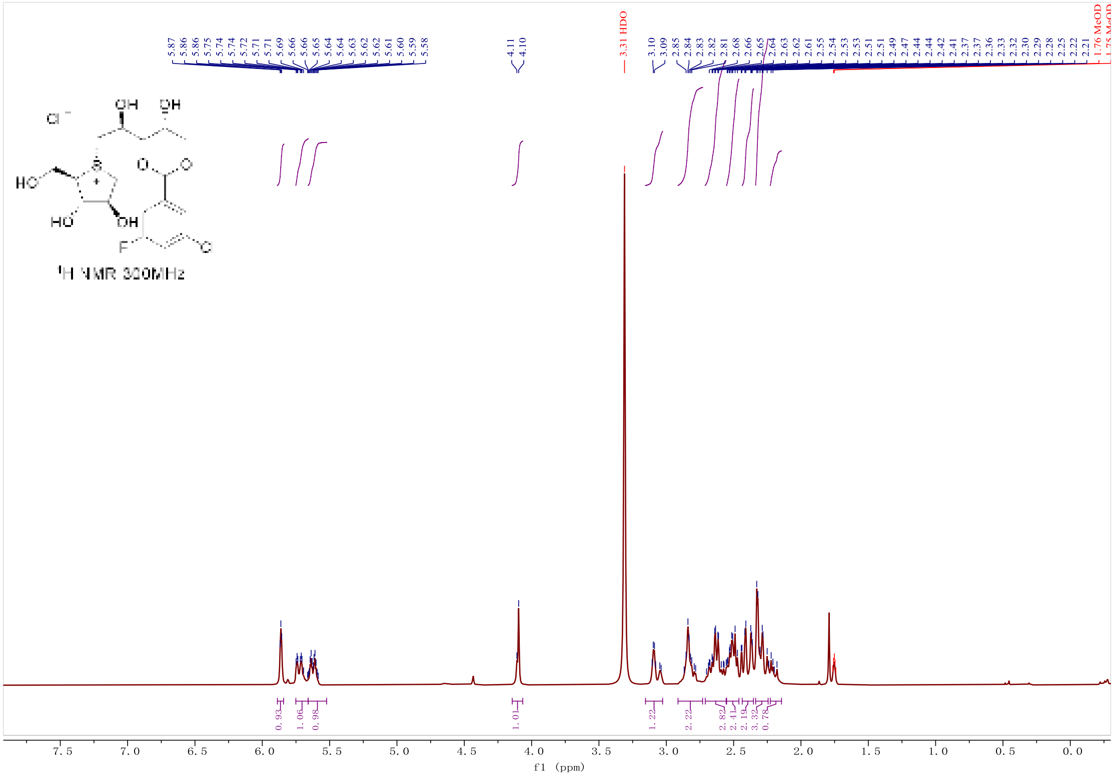

^{13}C-NMR spectrum of **19h**

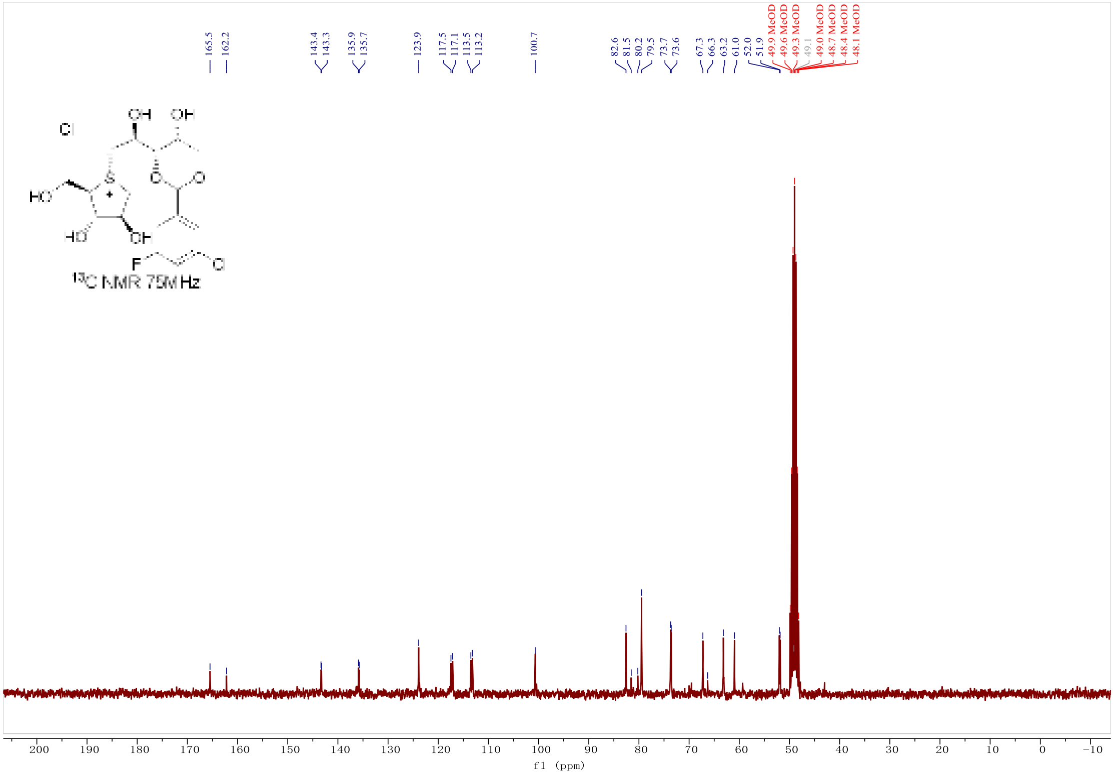

^{1}H-NMR spectrum of **19i**

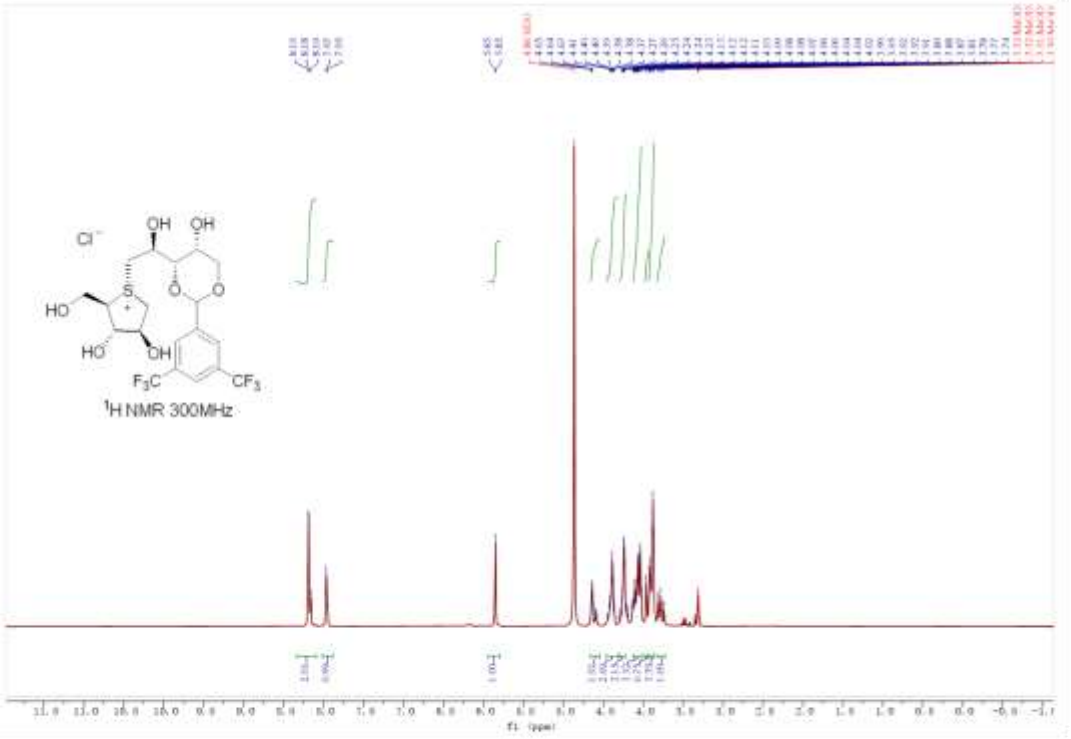

^{13}C-NMR spectrum of **19i**

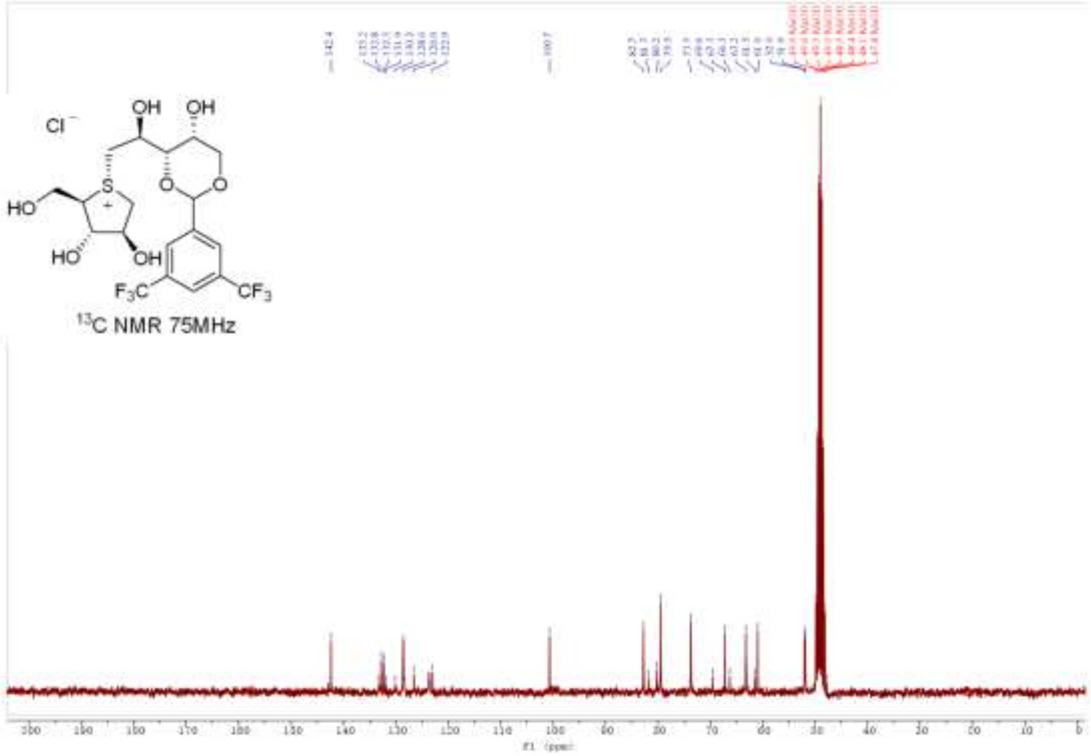

^{1}H-NMR spectrum of **19j**

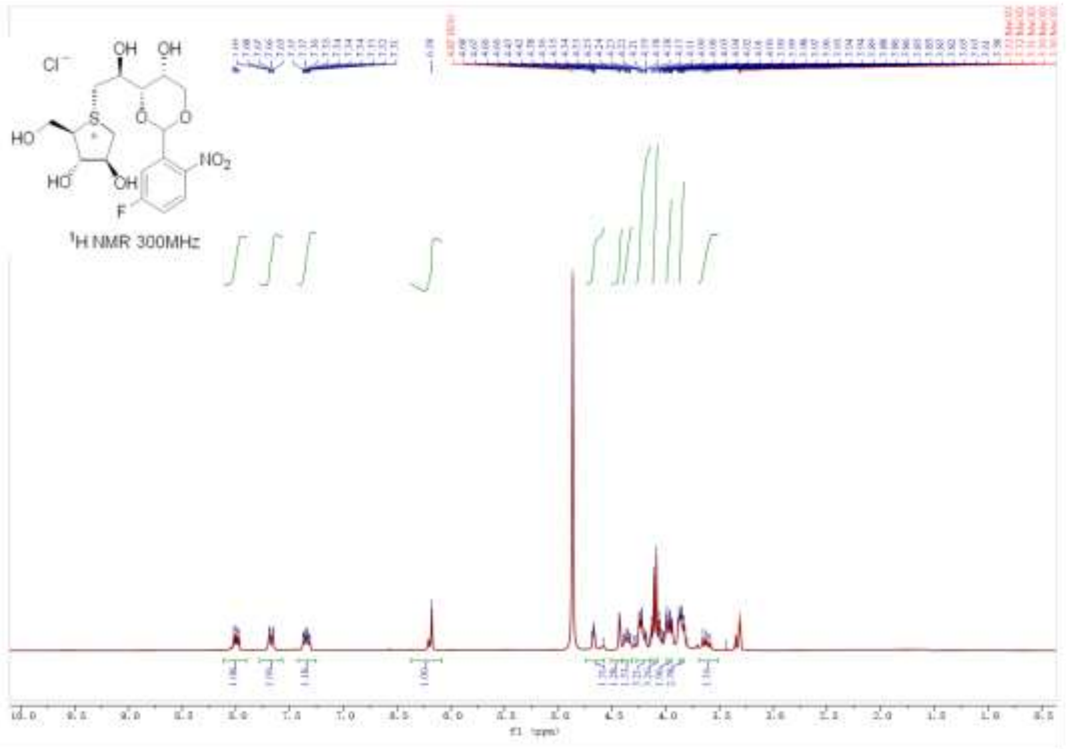

^{13}C-NMR spectrum of **19j**

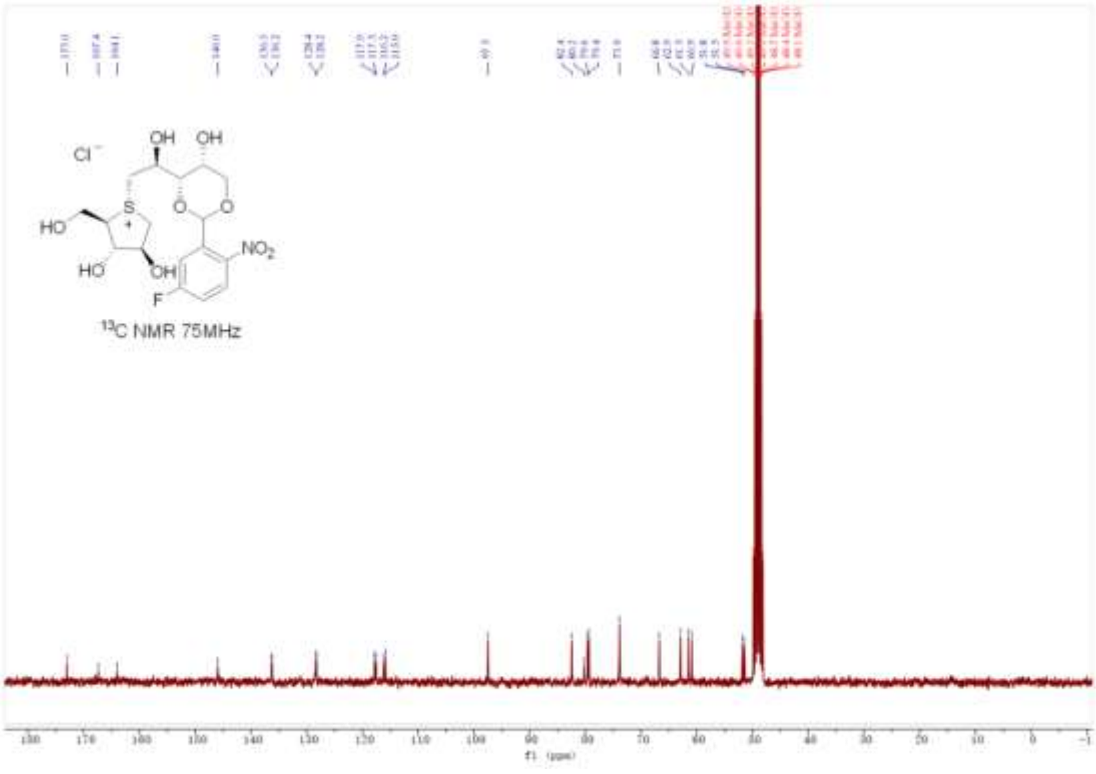

^{1}H-NMR spectrum of **19k**

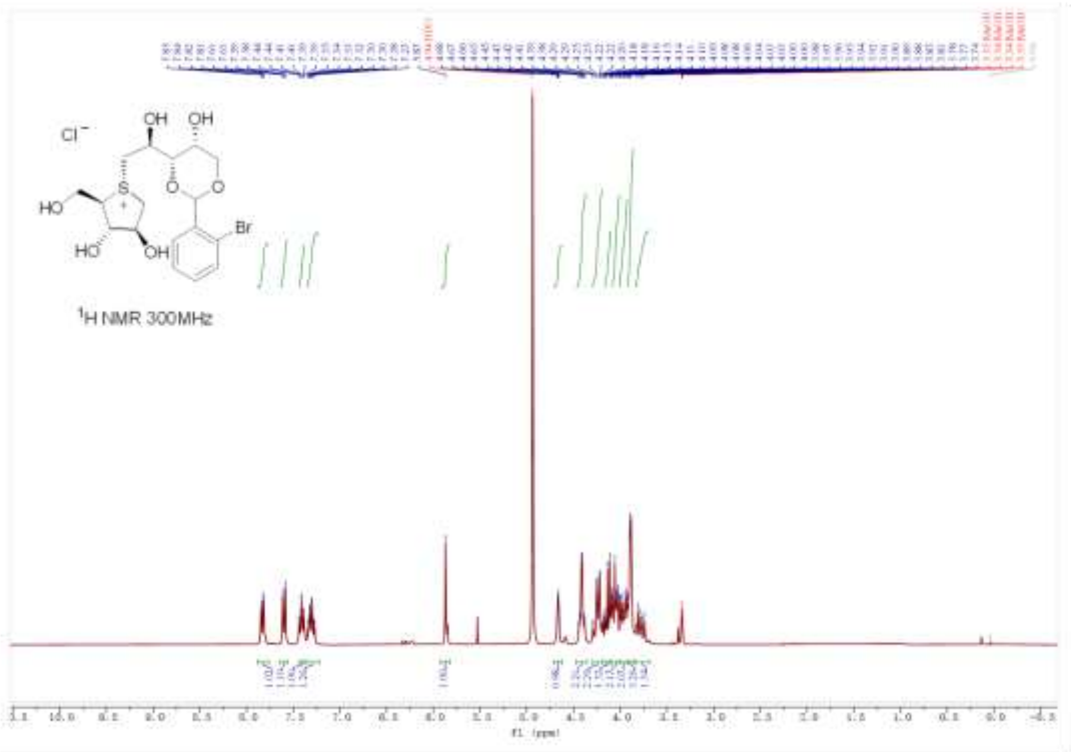

^{13}C-NMR spectrum of **19k**

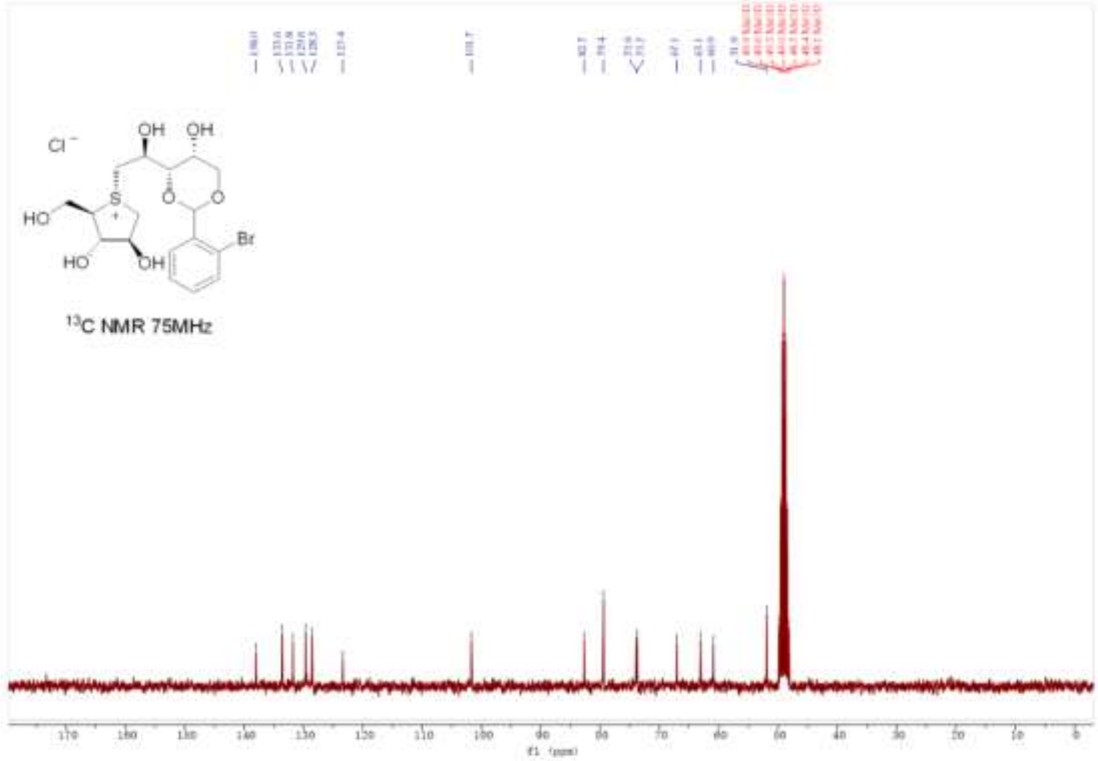

^{1}H-NMR spectrum of **19l**

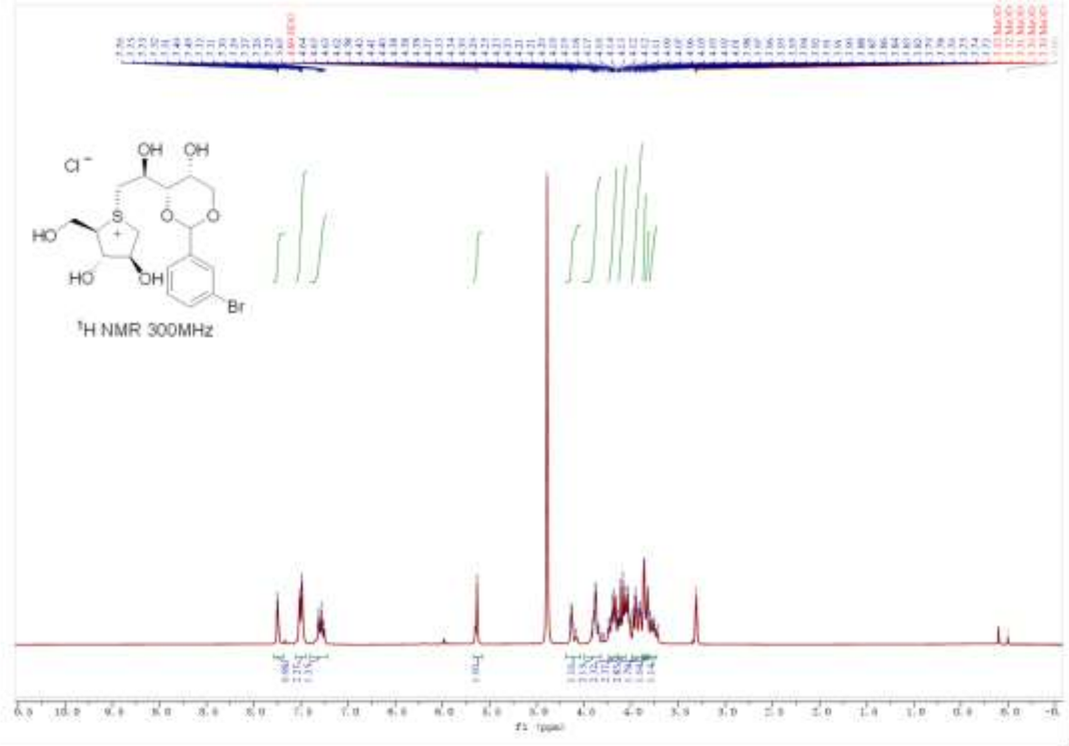

^{13}C-NMR spectrum of **19l**

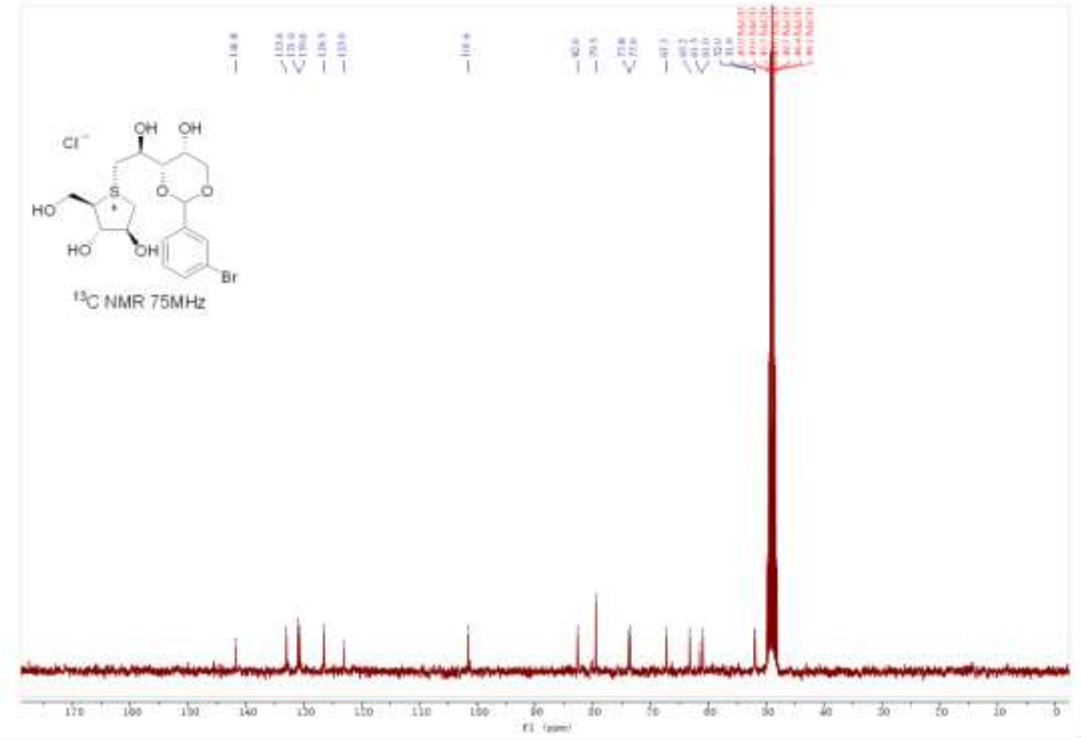

^{1}H-NMR spectrum of **19m**

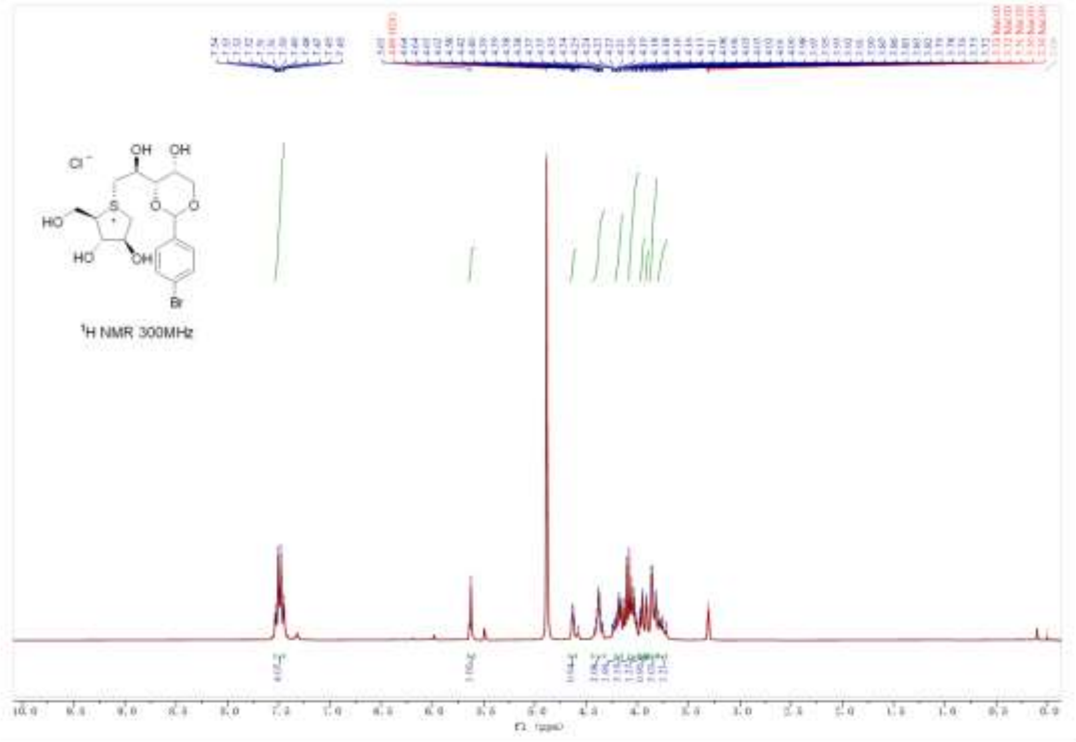

^{13}C-NMR spectrum of **19m**

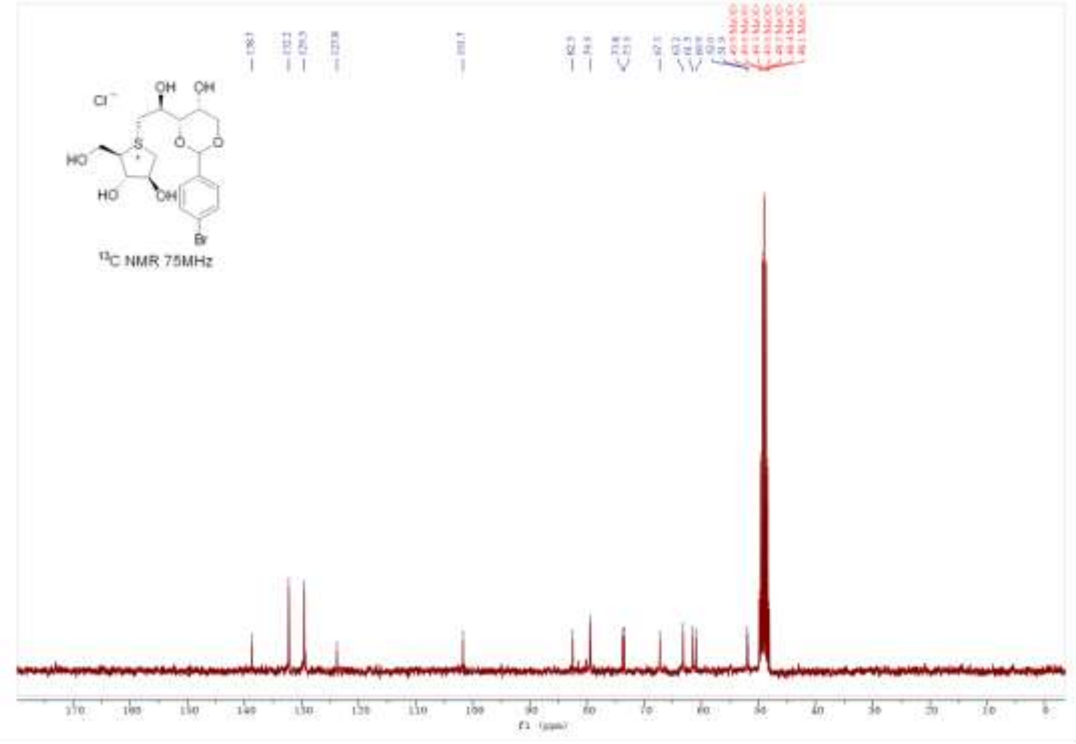

^{1}H-NMR spectrum of **19n**

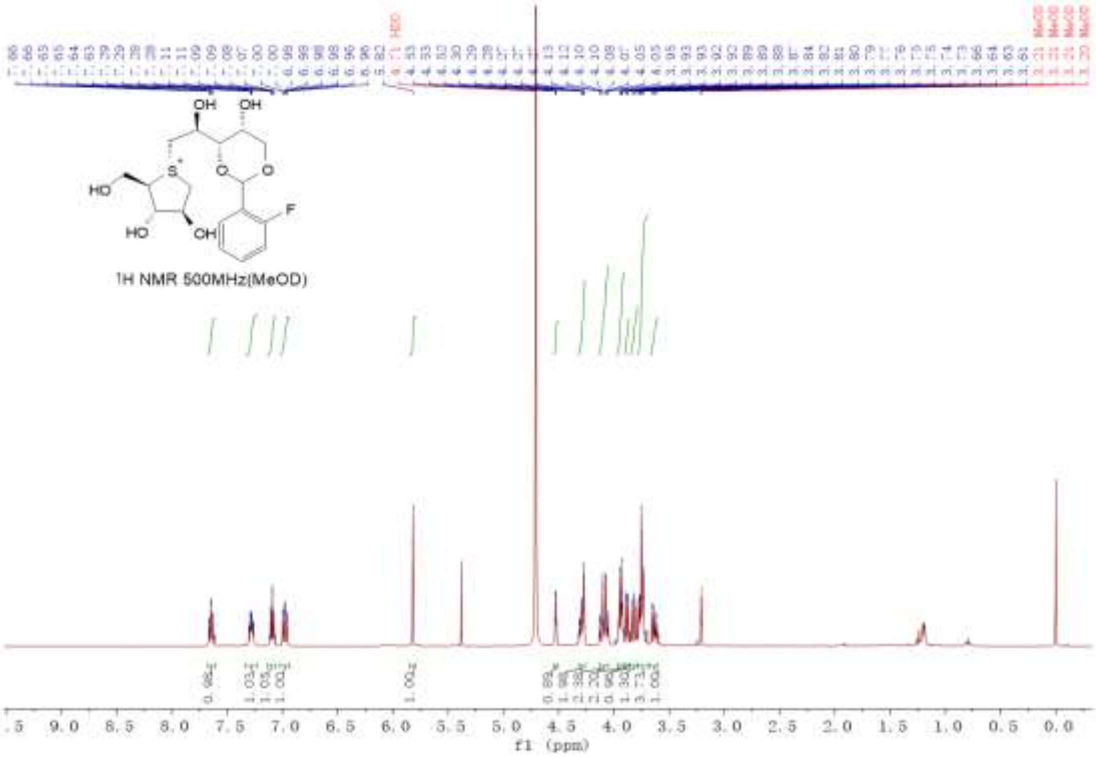

^{13}C-NMR spectrum of **19n**

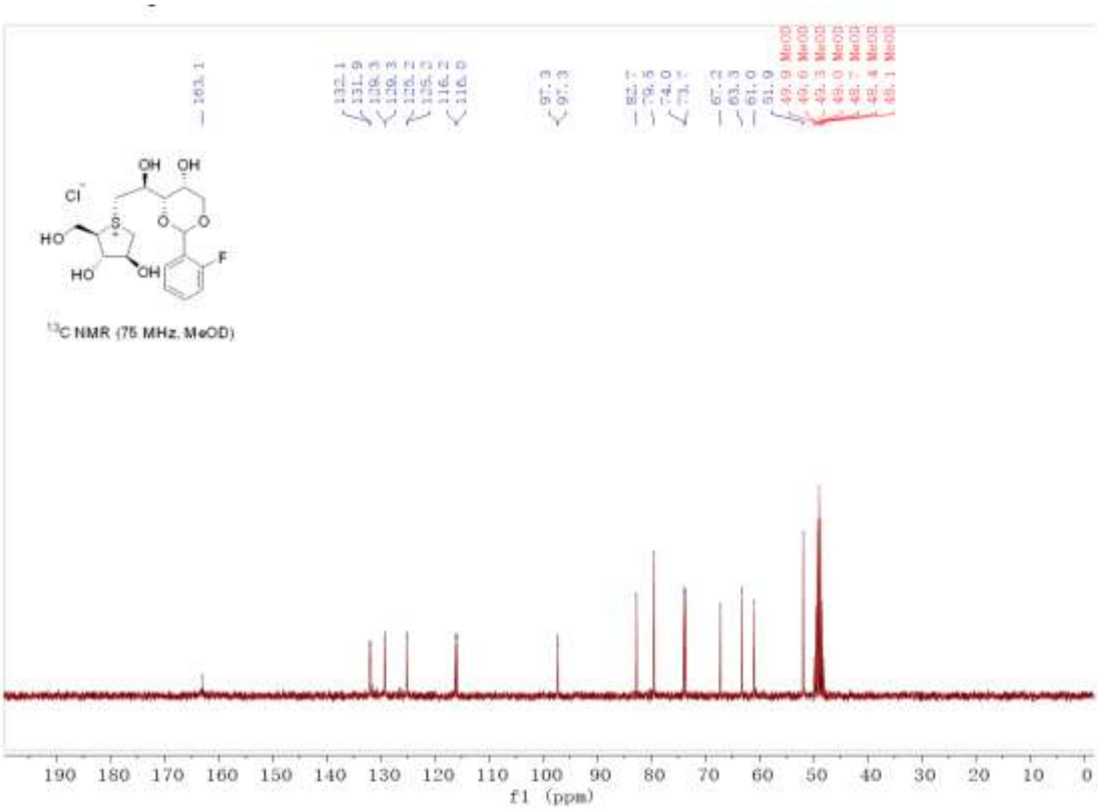

^{1}H-NMR spectrum of **19o**

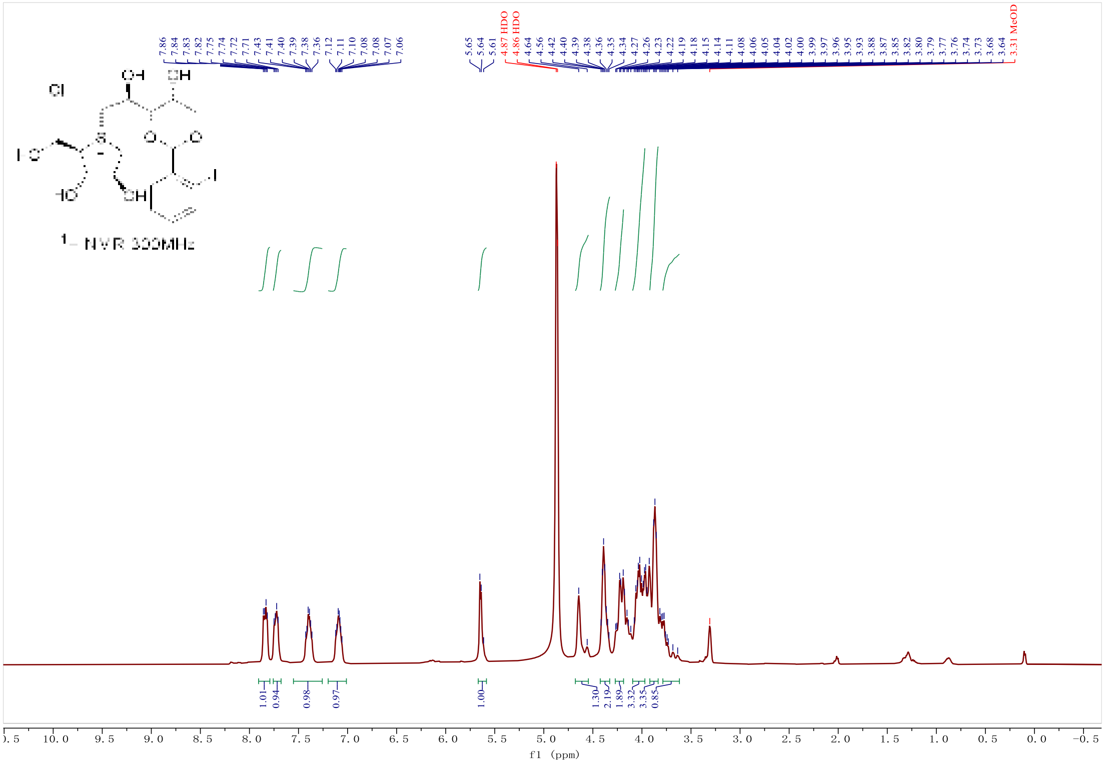

^{13}C-NMR spectrum of **19o**

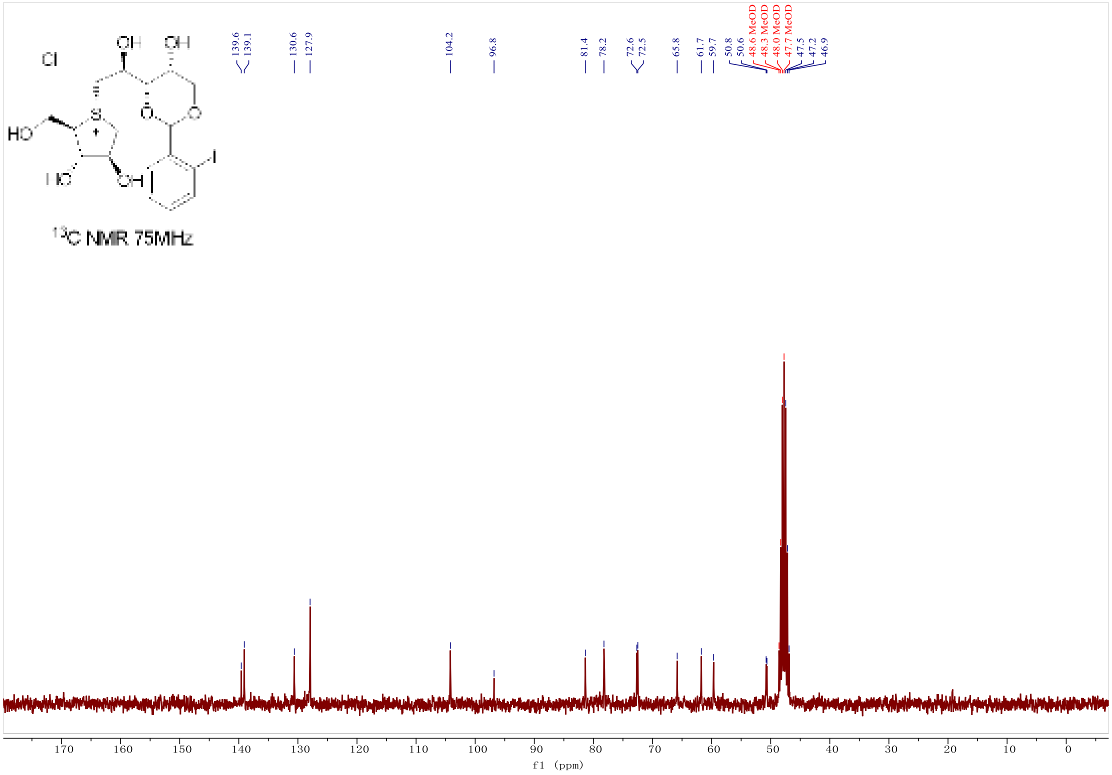

^{1}H-NMR spectrum of **19p**

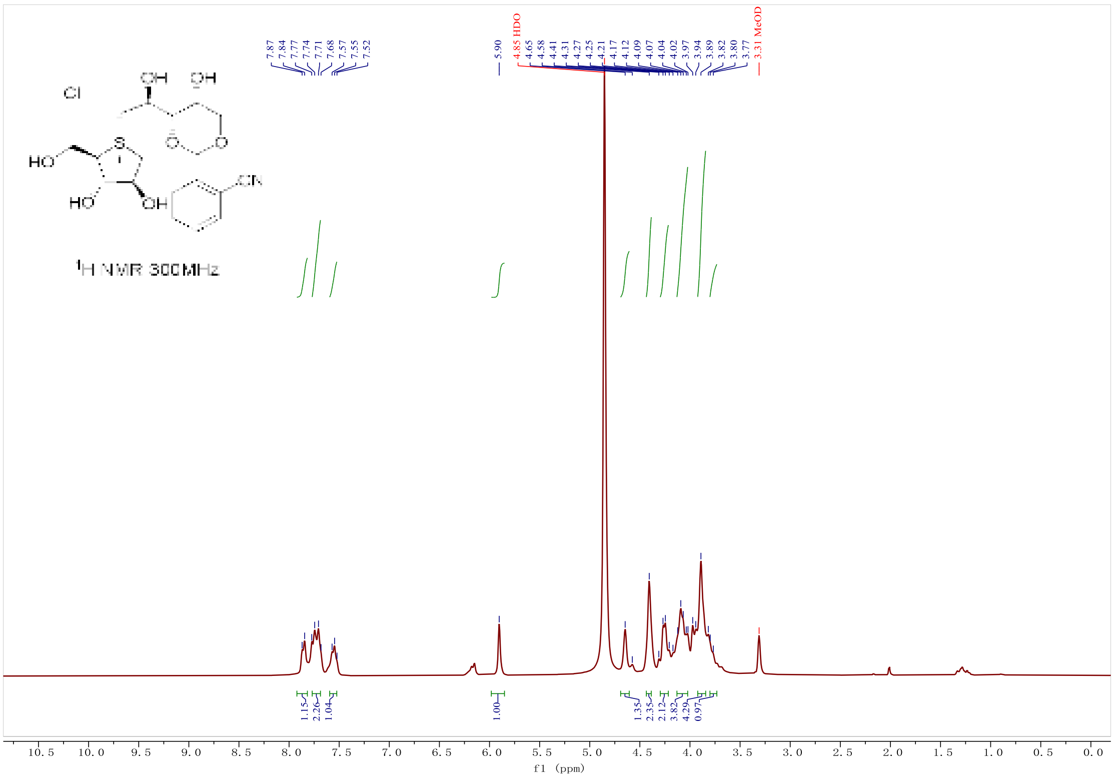

^{13}C-NMR spectrum of **19p**

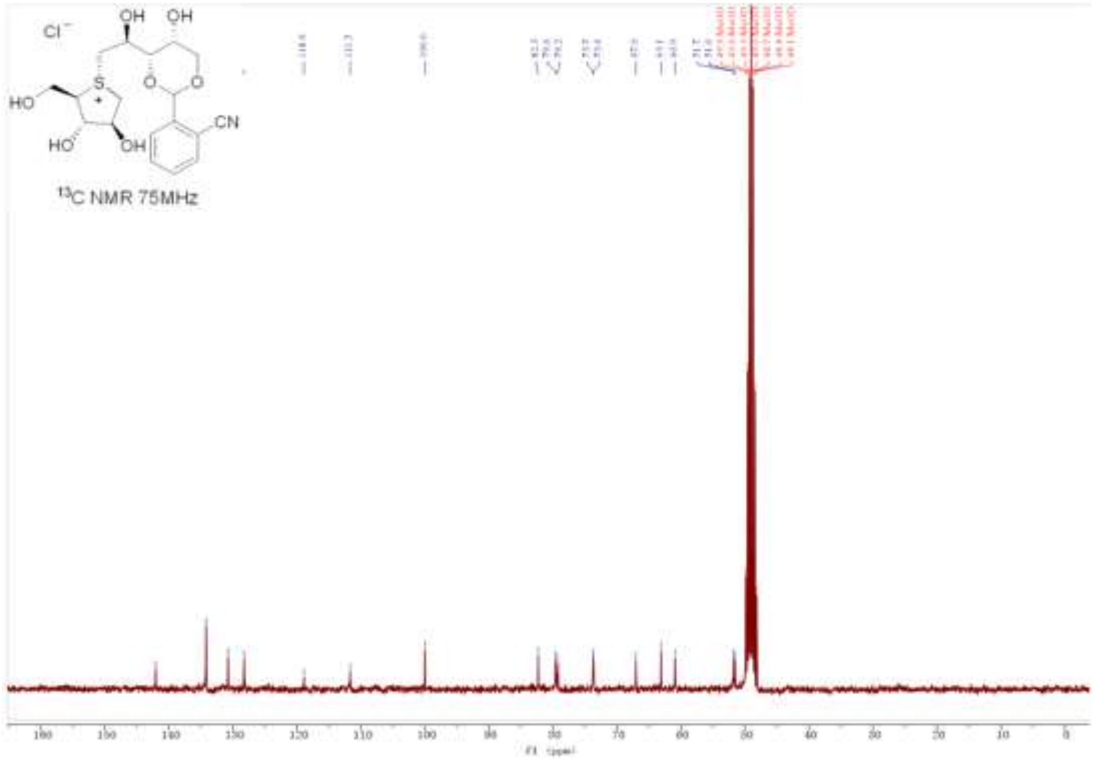

^{1}H-NMR spectrum of **19q**

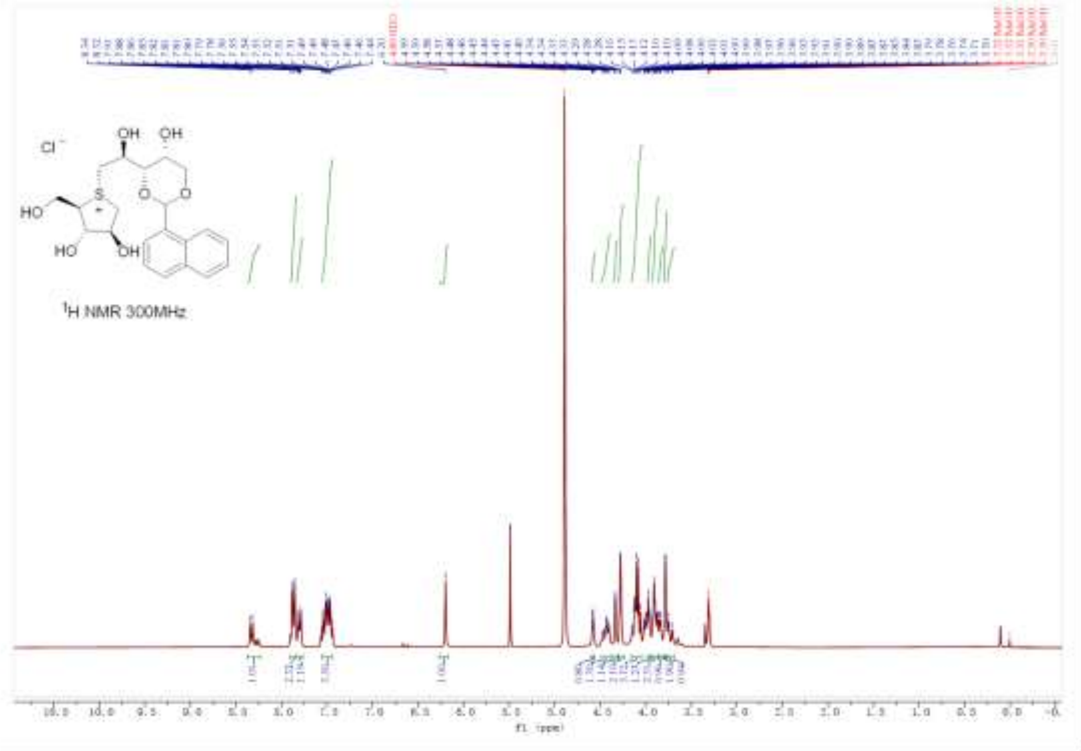

^{13}C-NMR spectrum of **19q**

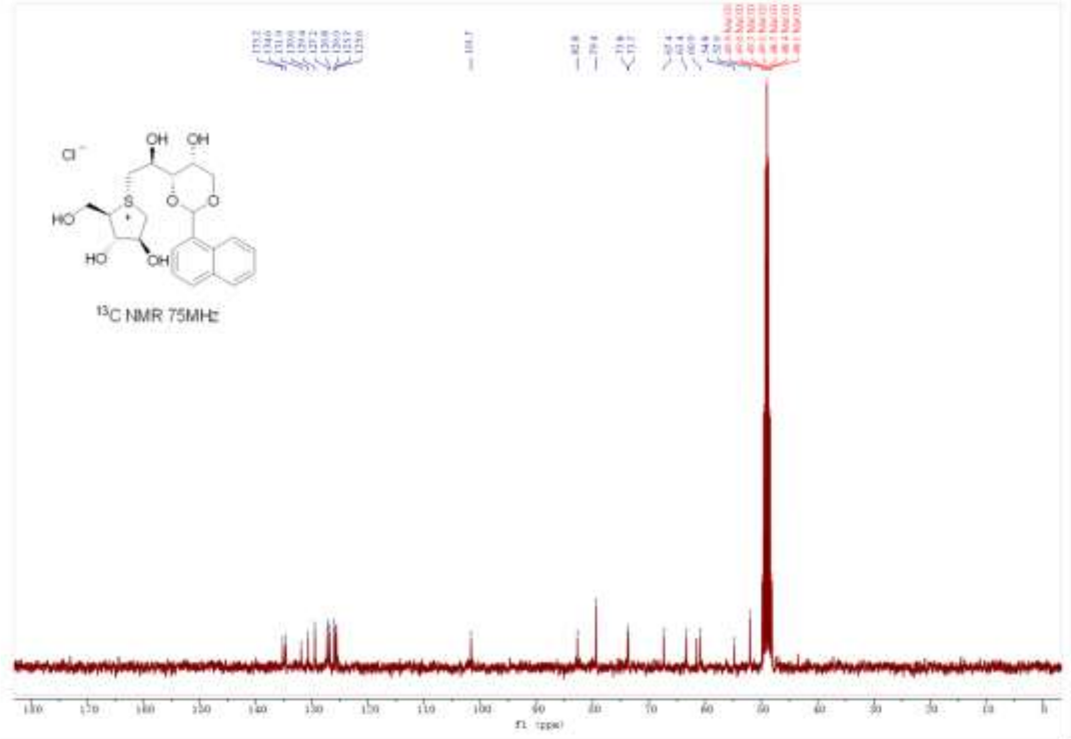

^{1}H-NMR spectrum of **19r**

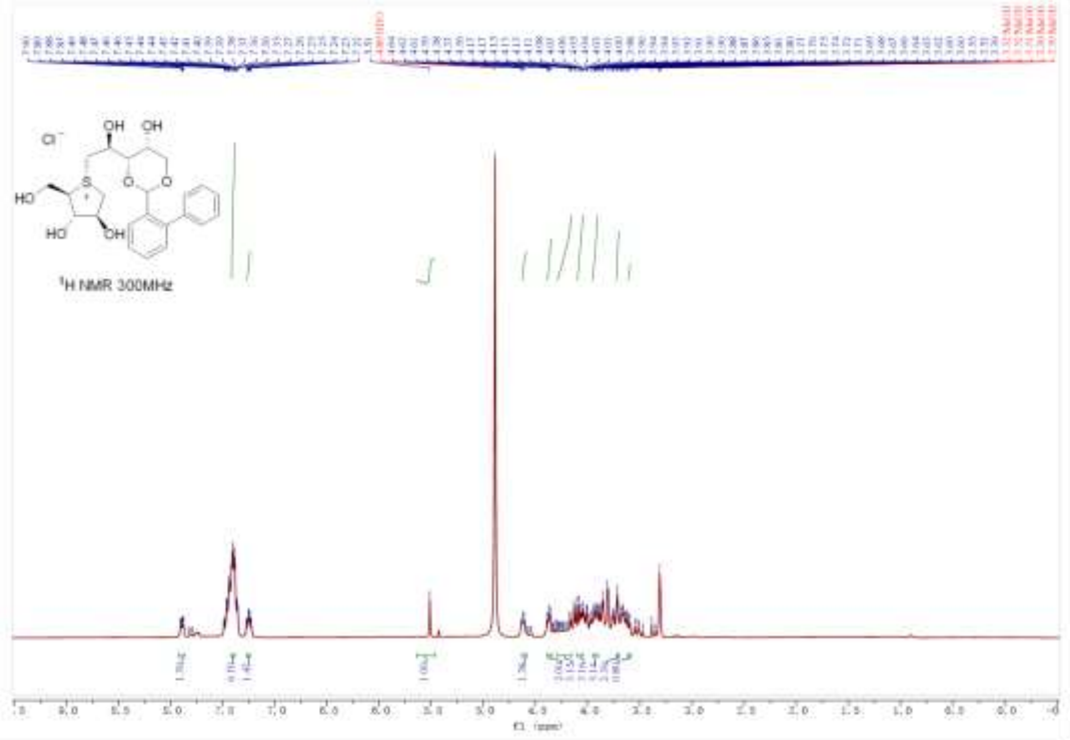

^{13}C-NMR spectrum of **19r**

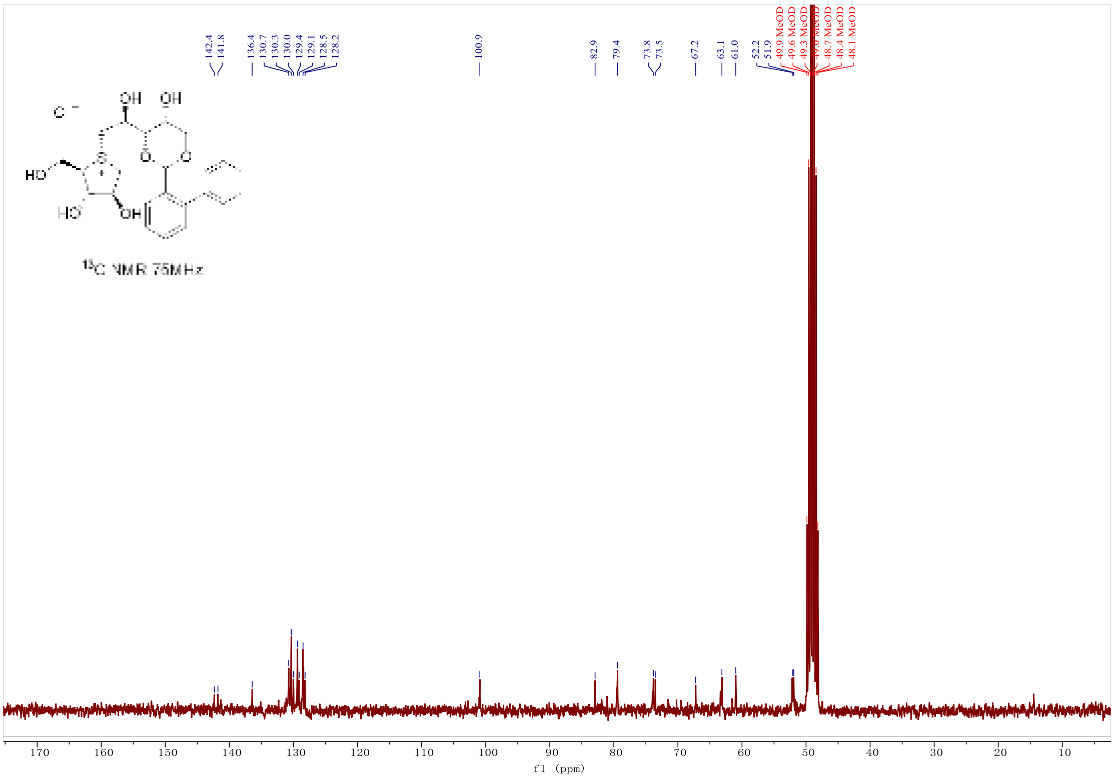

^{1}H-NMR spectrum of **20a**

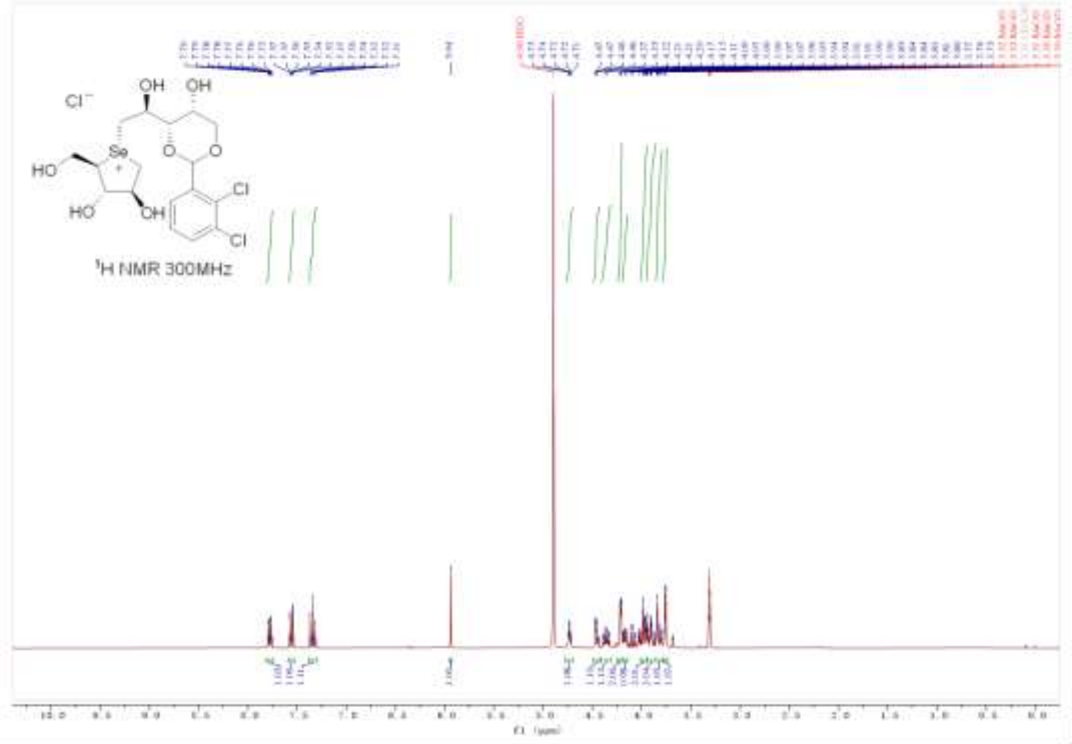

^{13}C-NMR spectrum of **20a**

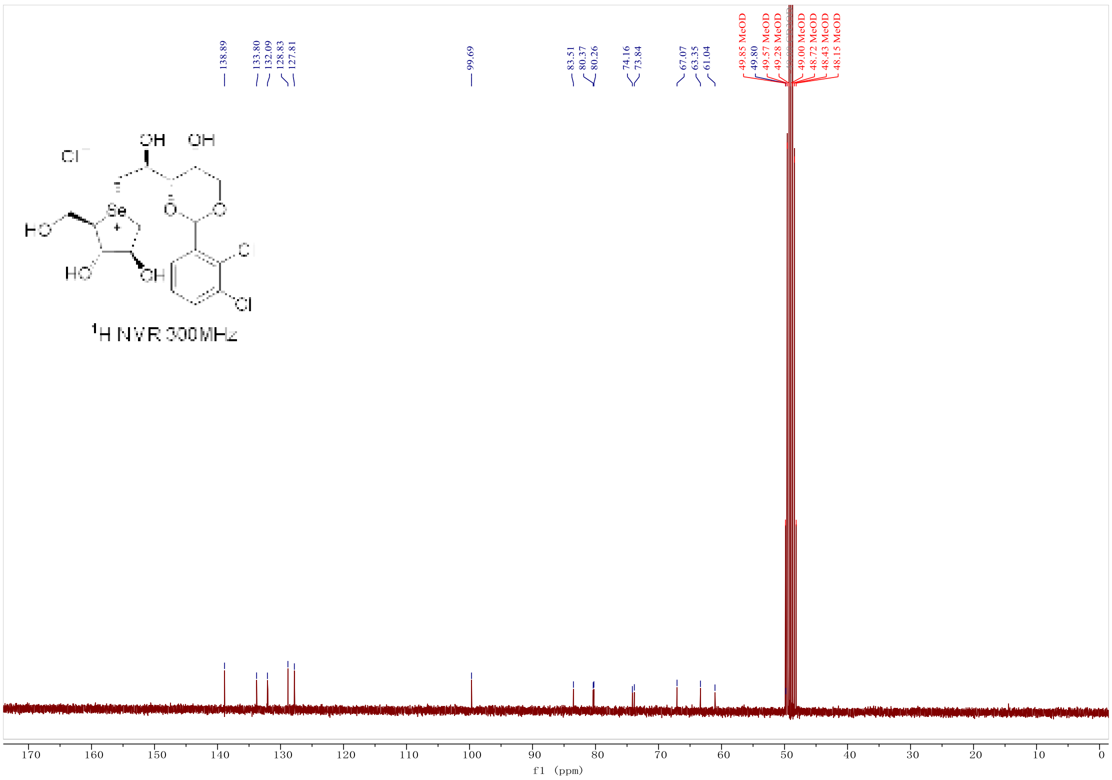

^{1}H-NMR spectrum of **20b**

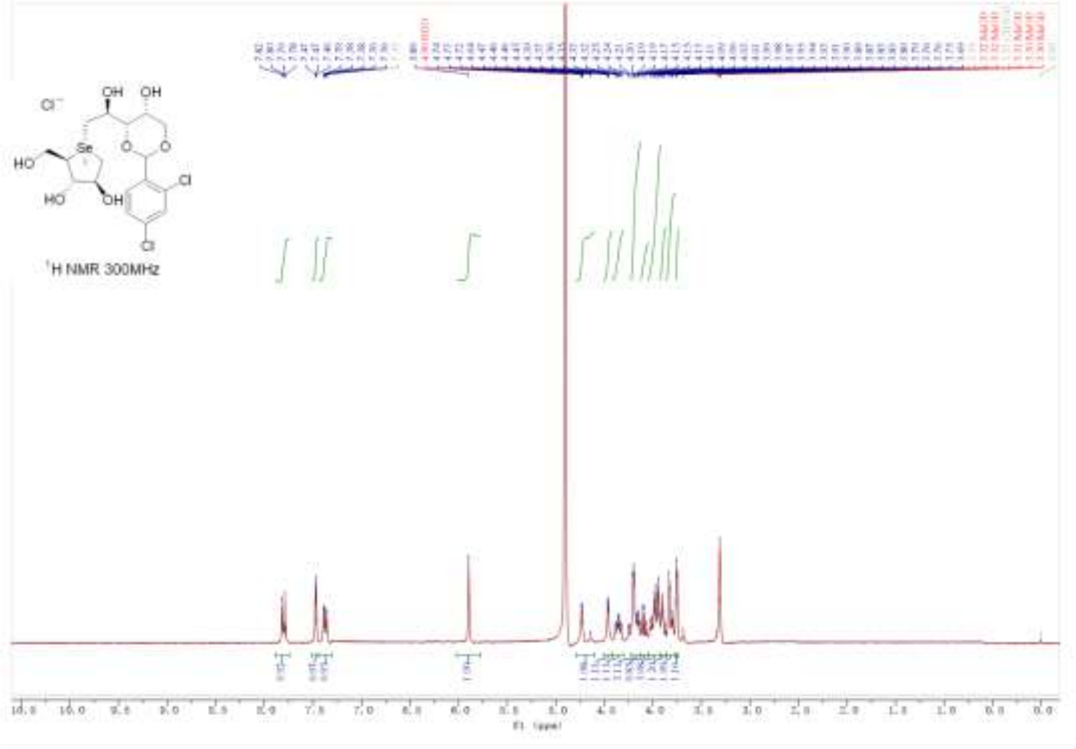

^{13}C-NMR spectrum of **20b**

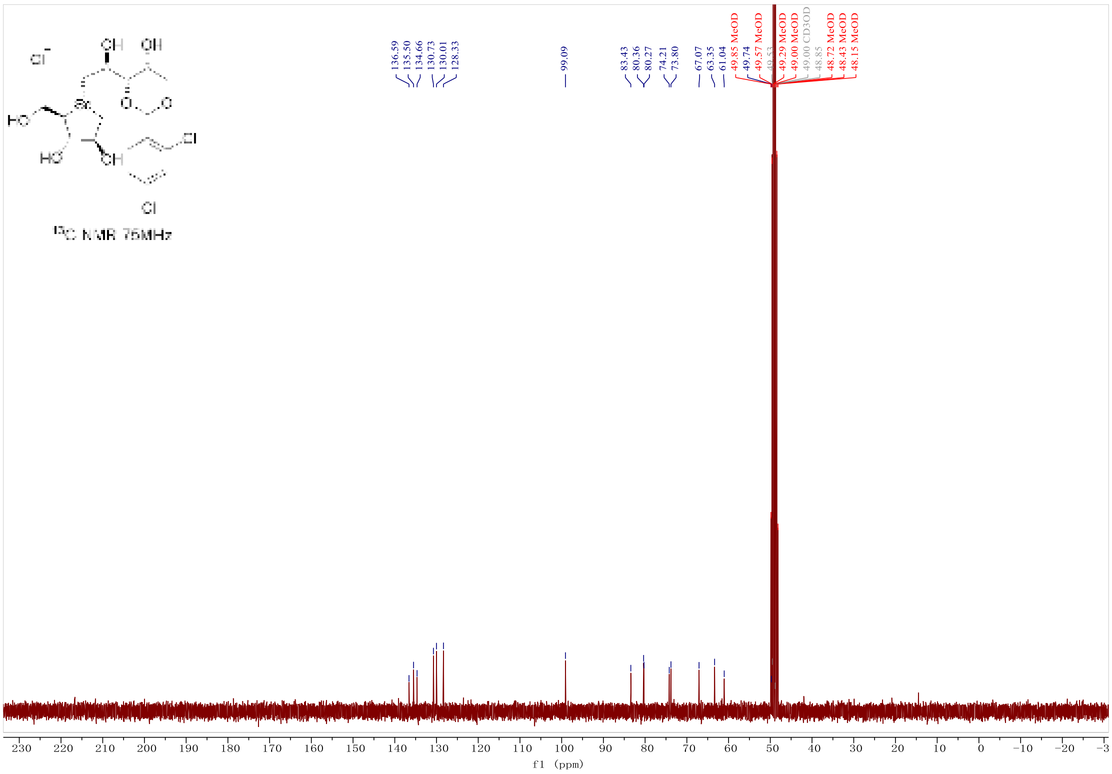

^{1}H-NMR spectrum of **20c**

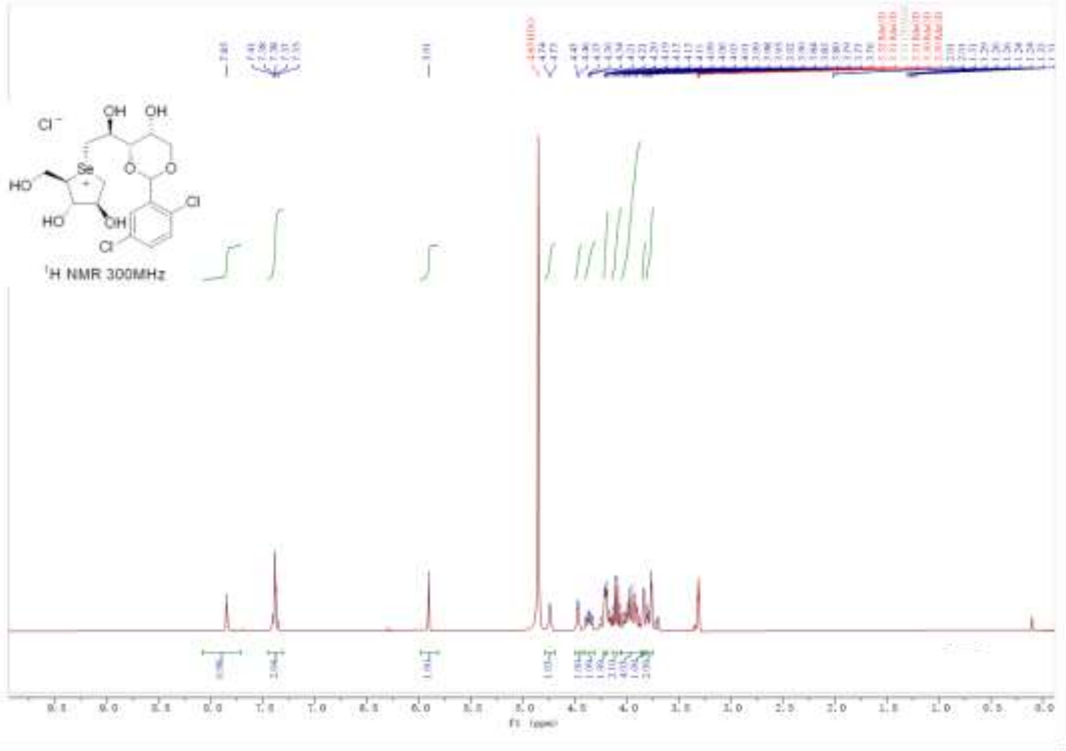

^{13}C-NMR spectrum of **20c**

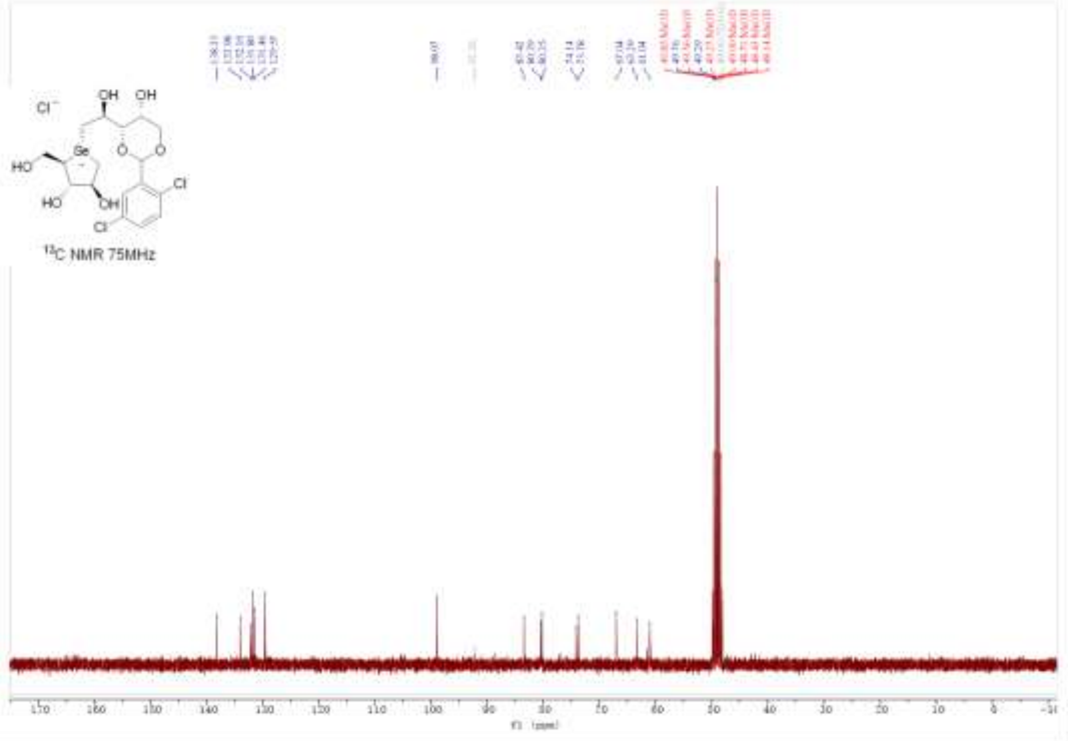

^{1}H-NMR spectrum of **20d**

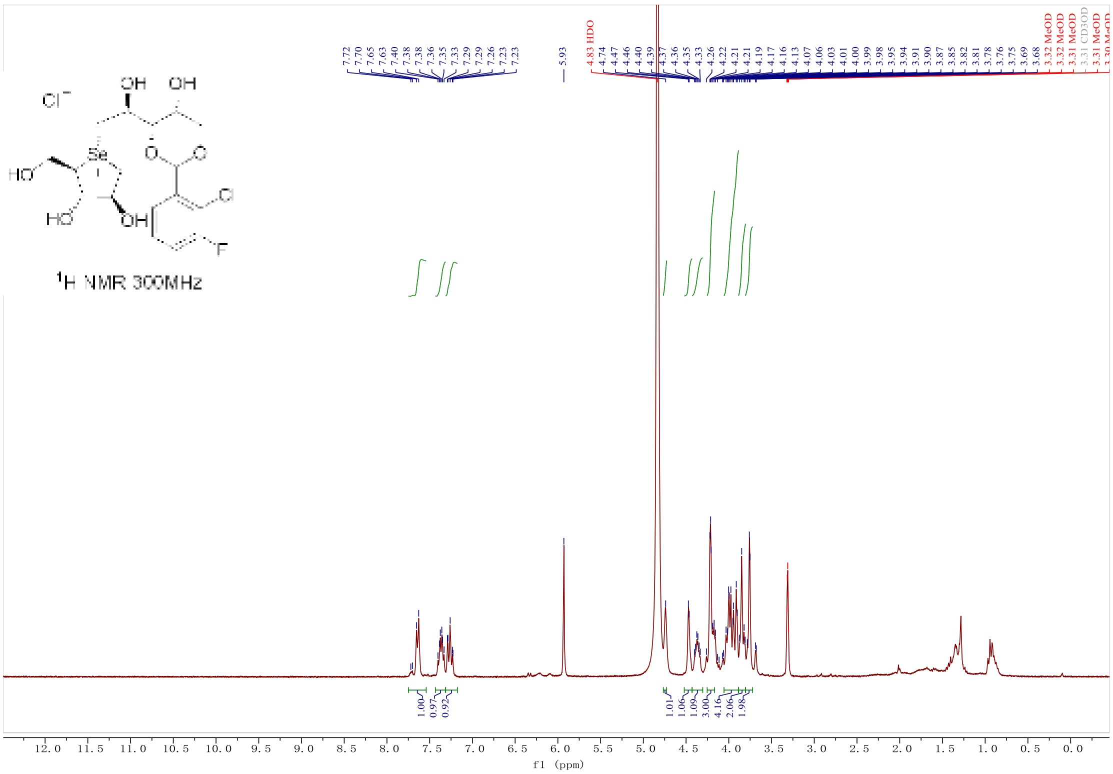

^{13}C-NMR spectrum of **20d**

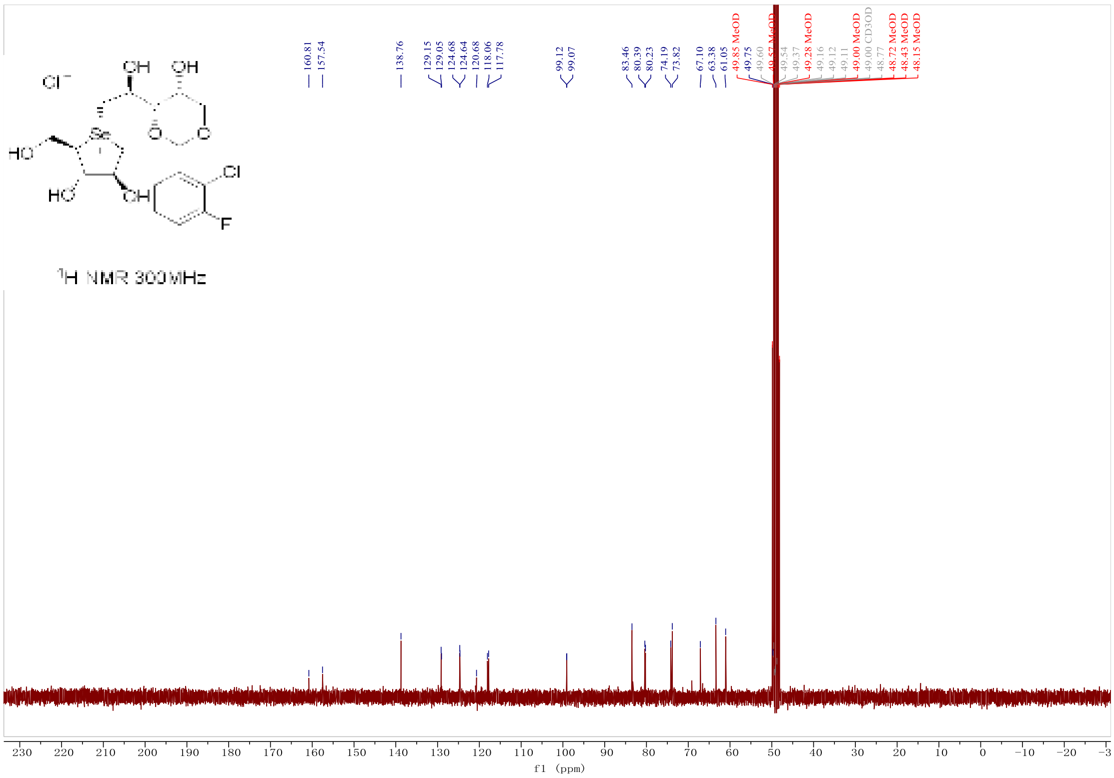

^{1}H-NMR spectrum of **20e**

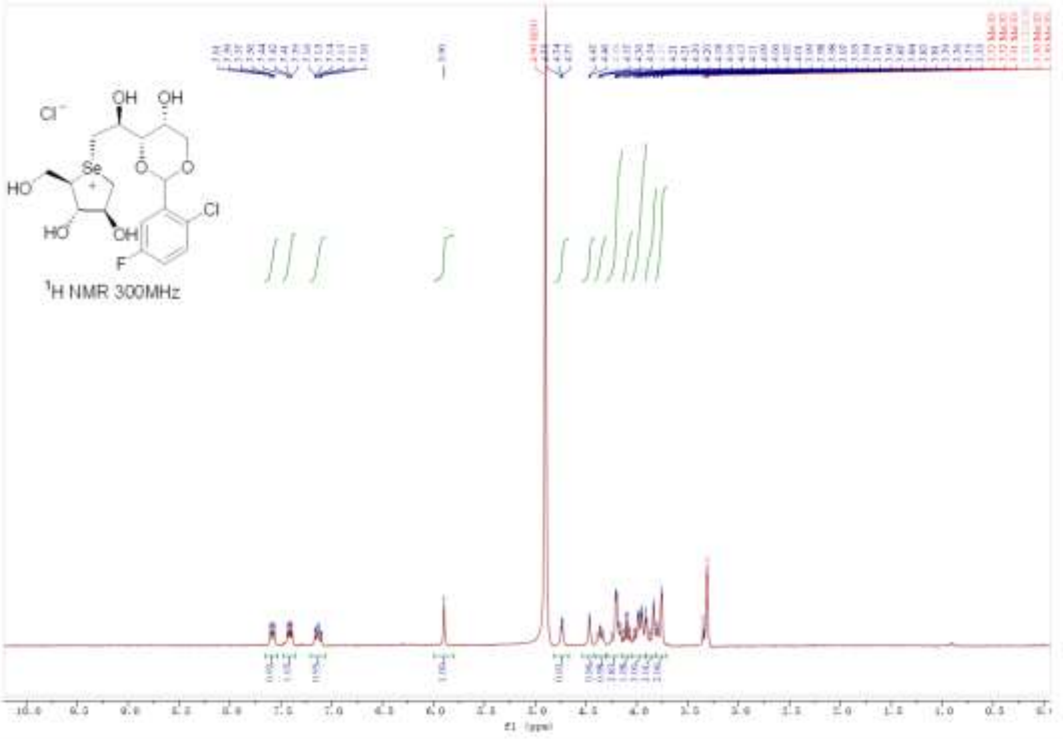

^{13}C-NMR spectrum of **20e**

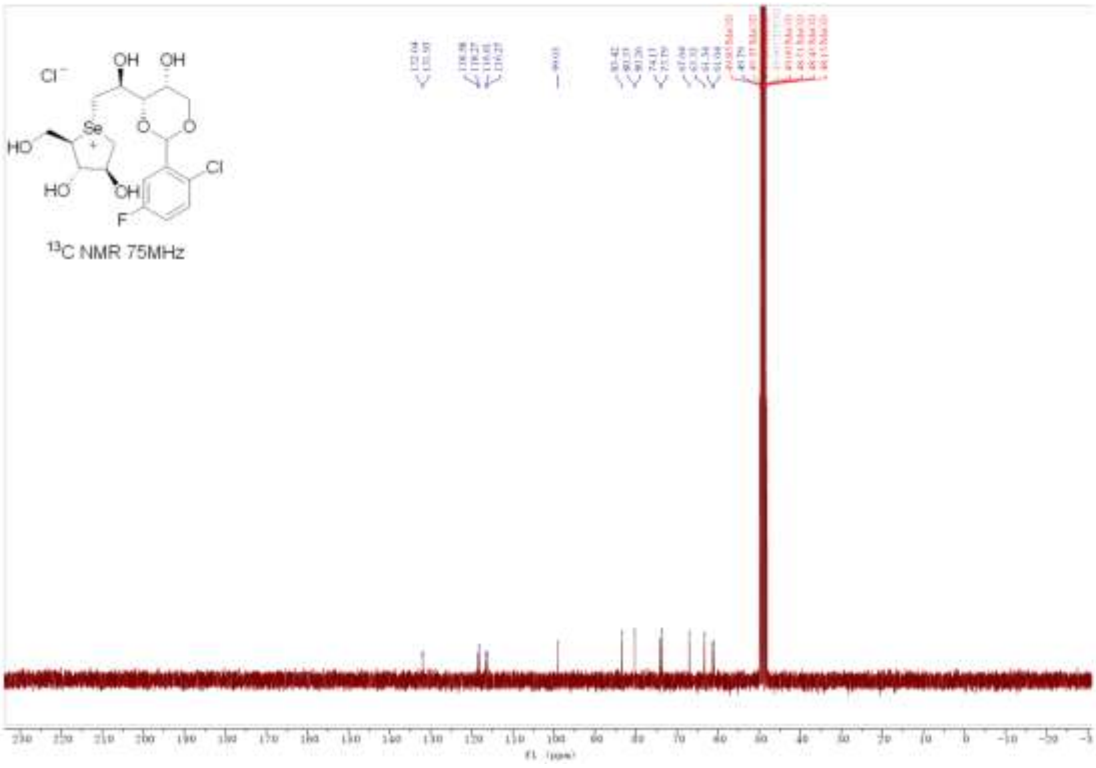

^{1}H-NMR spectrum of **20f**

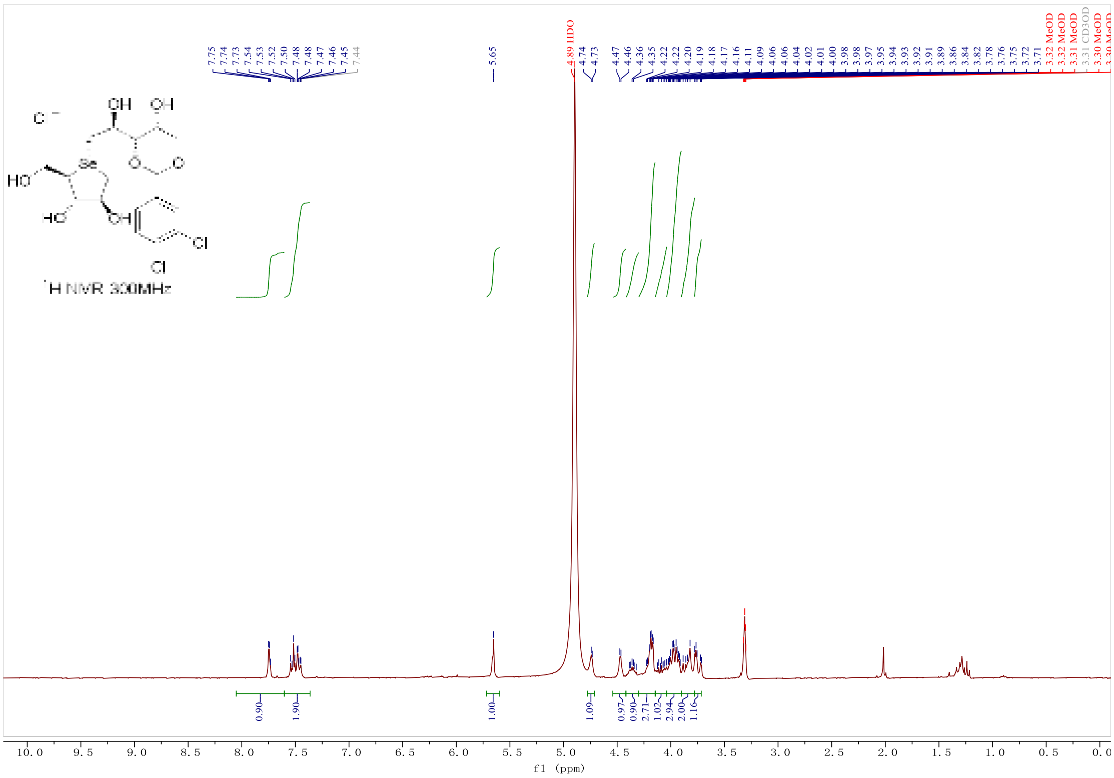

^{13}C-NMR spectrum of **20f**

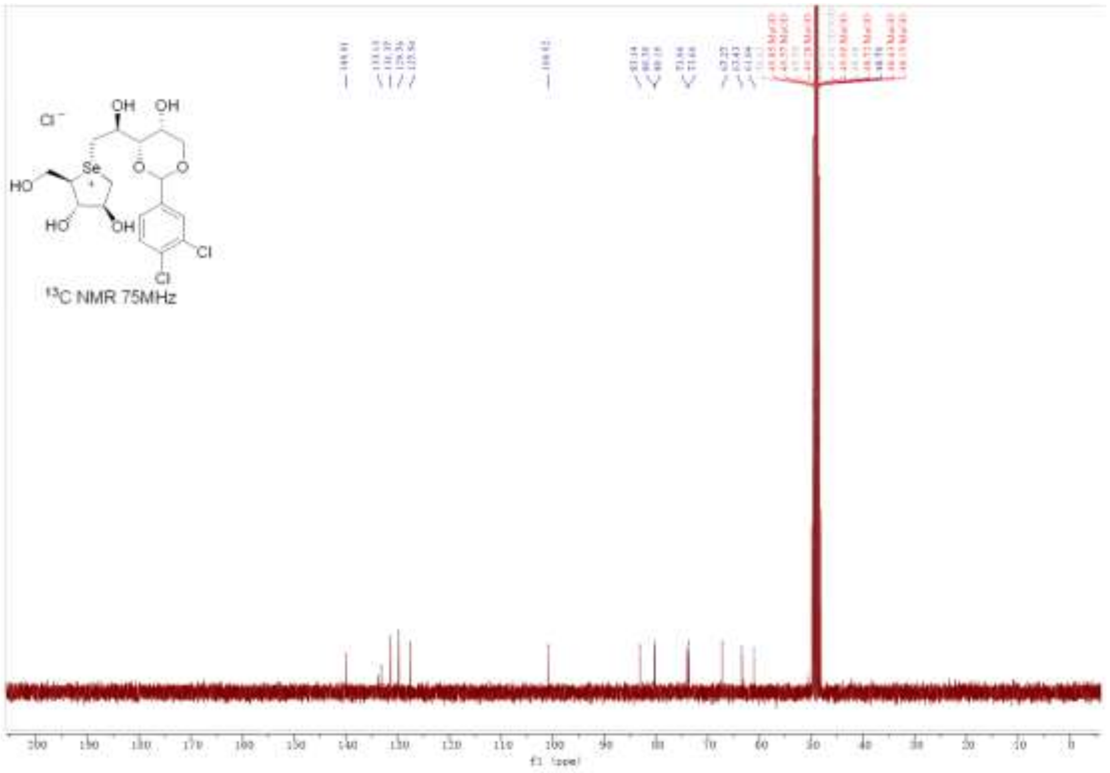

^{1}H-NMR spectrum of **20g**

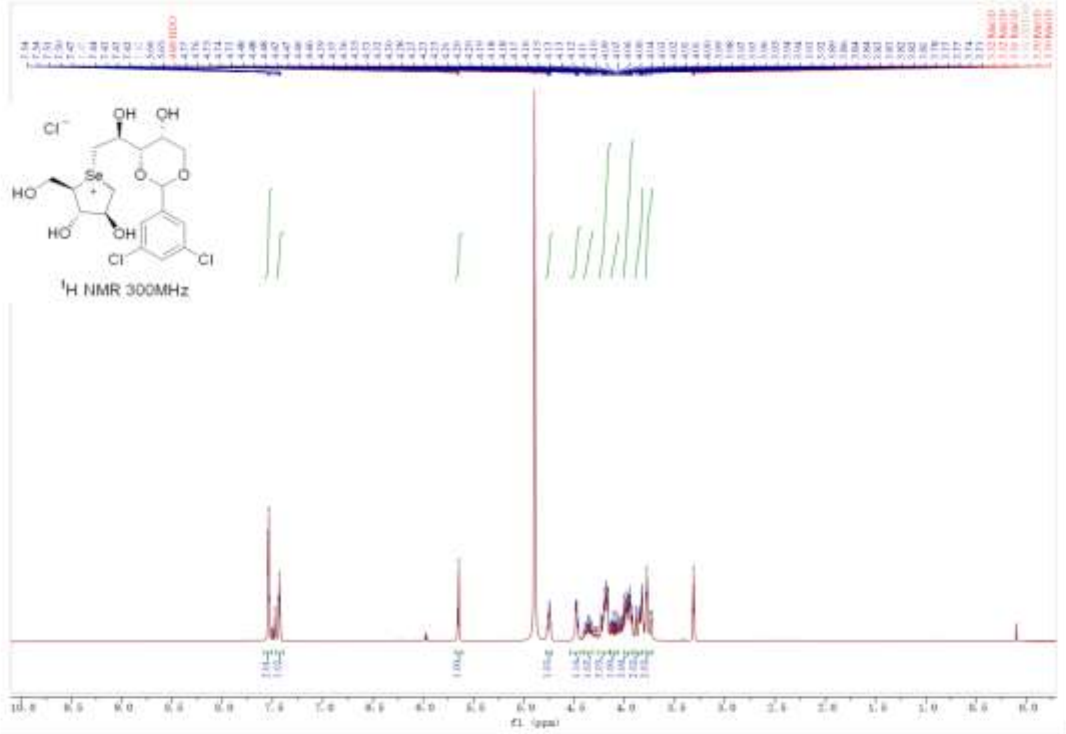

^{13}C-NMR spectrum of **20g**

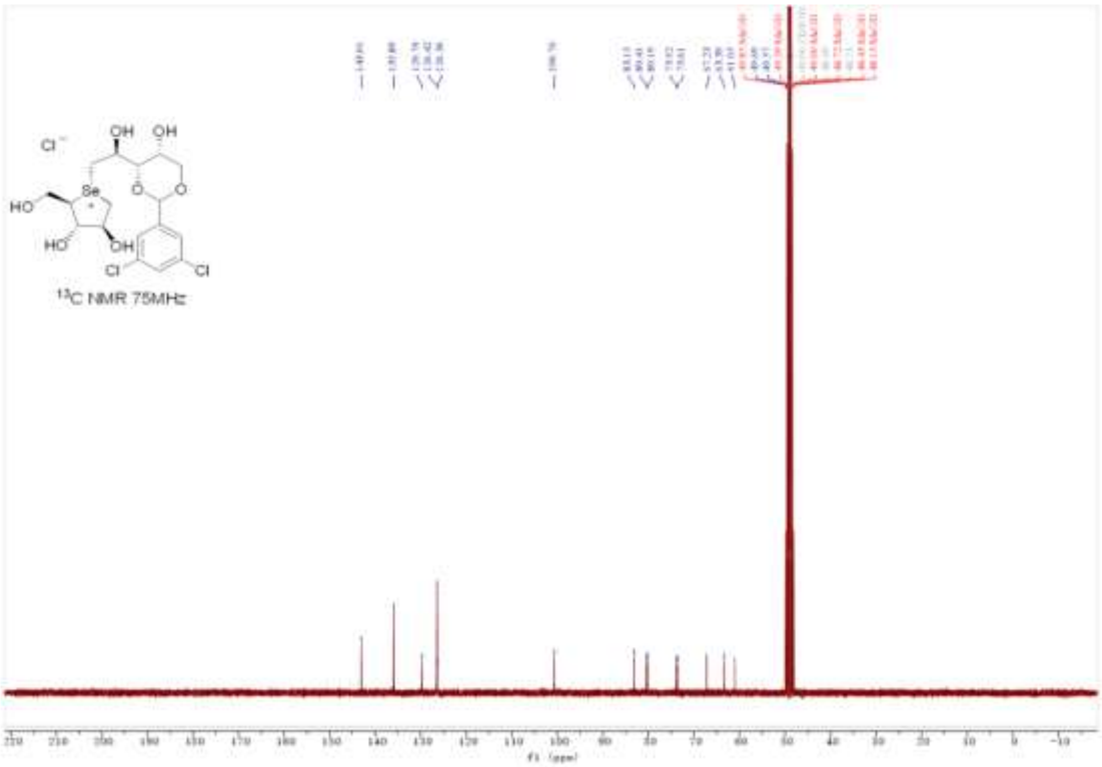

^{1}H-NMR spectrum of **20h**

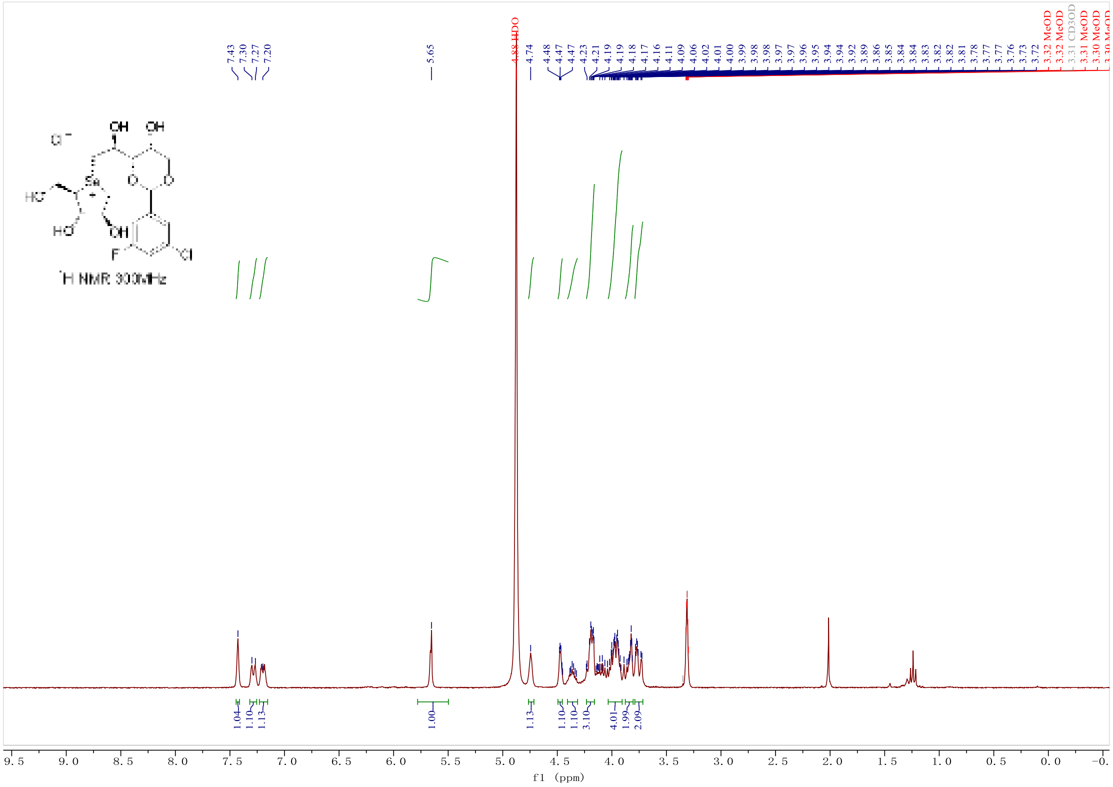

^{13}C-NMR spectrum of **20h**

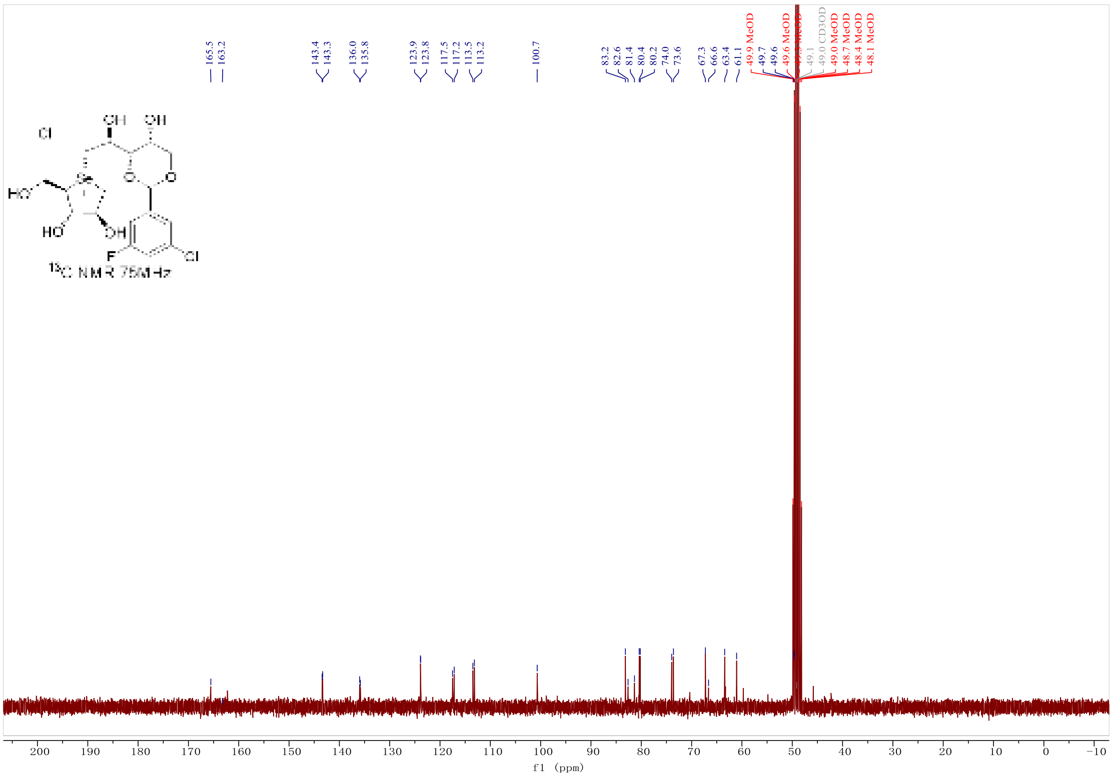

^{1}H-NMR spectrum of **20i**

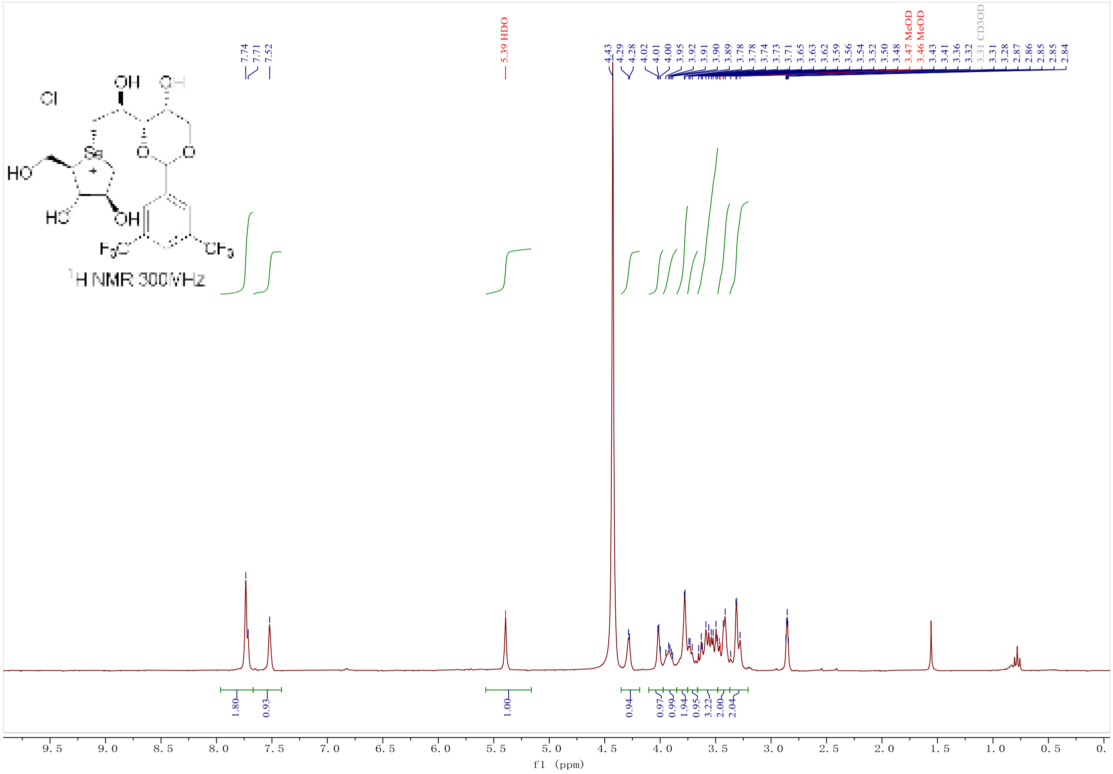

^{13}C-NMR spectrum of **20i**

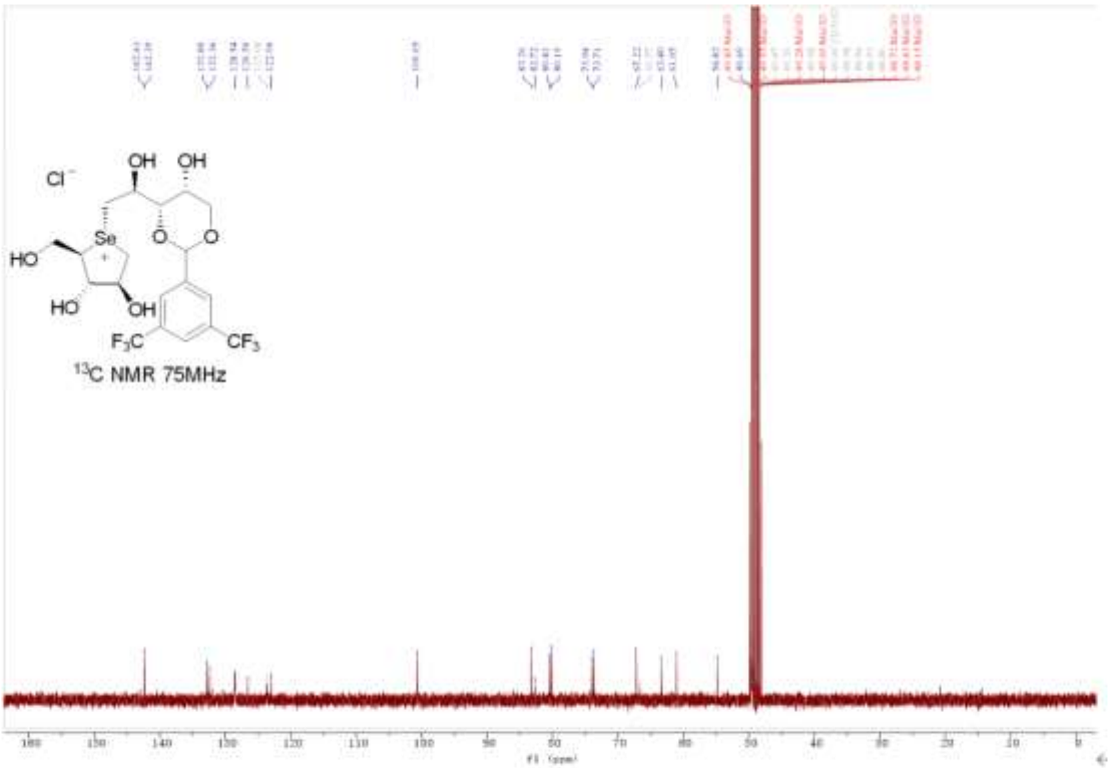

^{1}H-NMR spectrum of **20j**

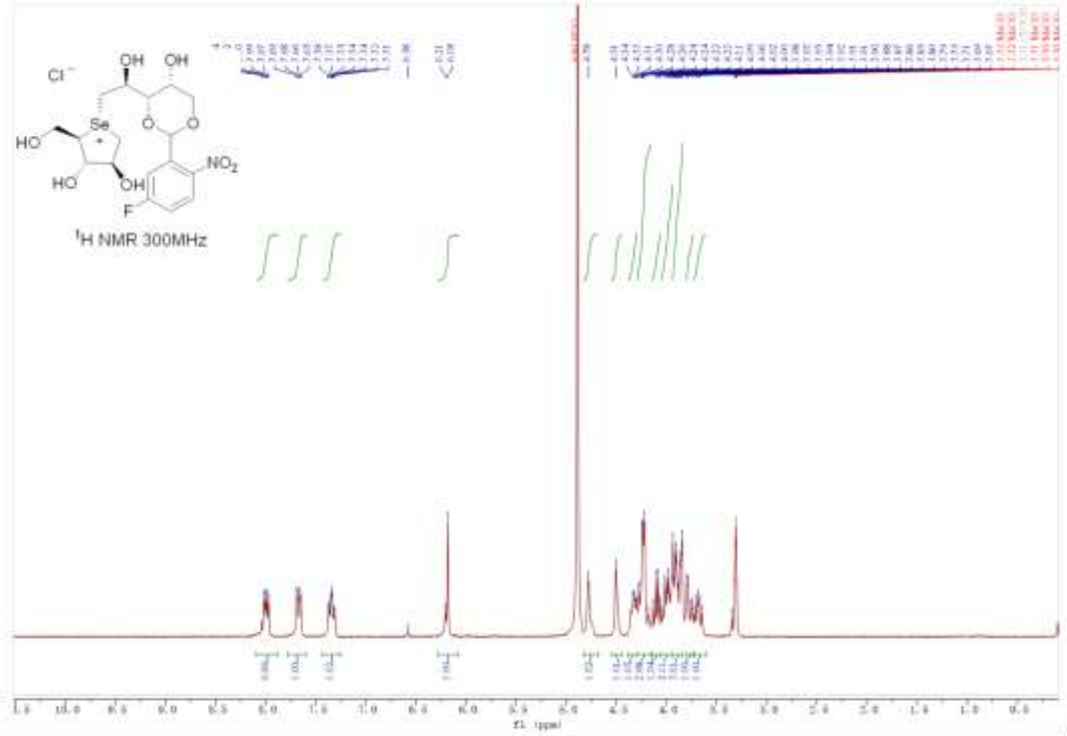

^{13}C-NMR spectrum of **20j**

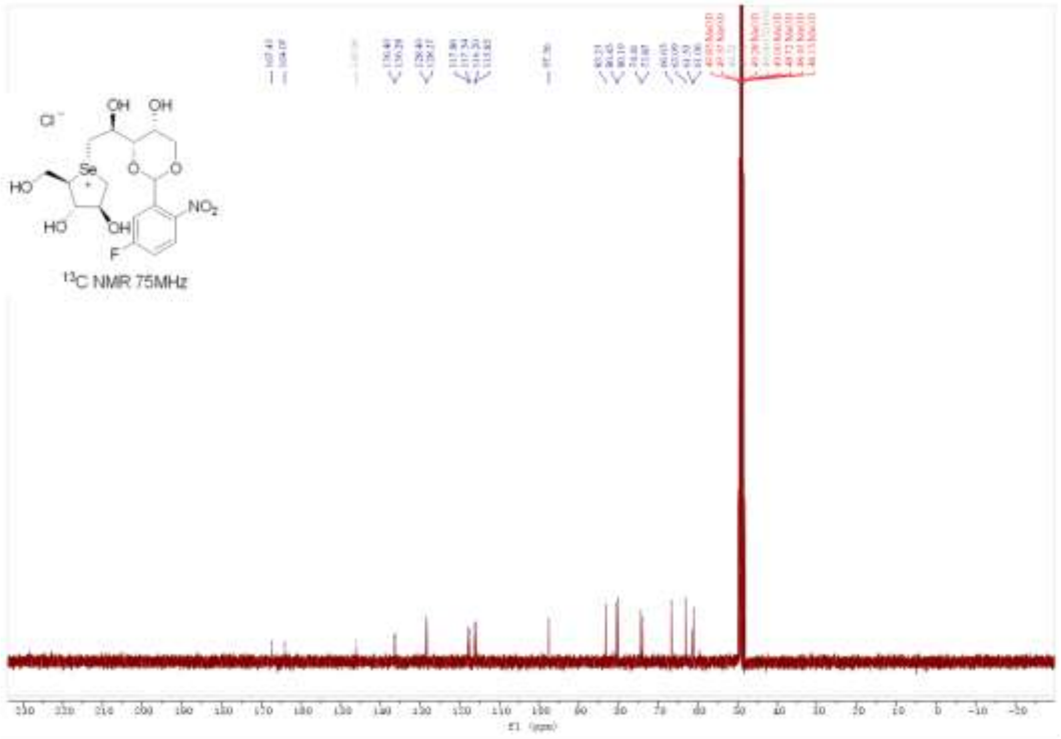

^{1}H-NMR spectrum of **20k**

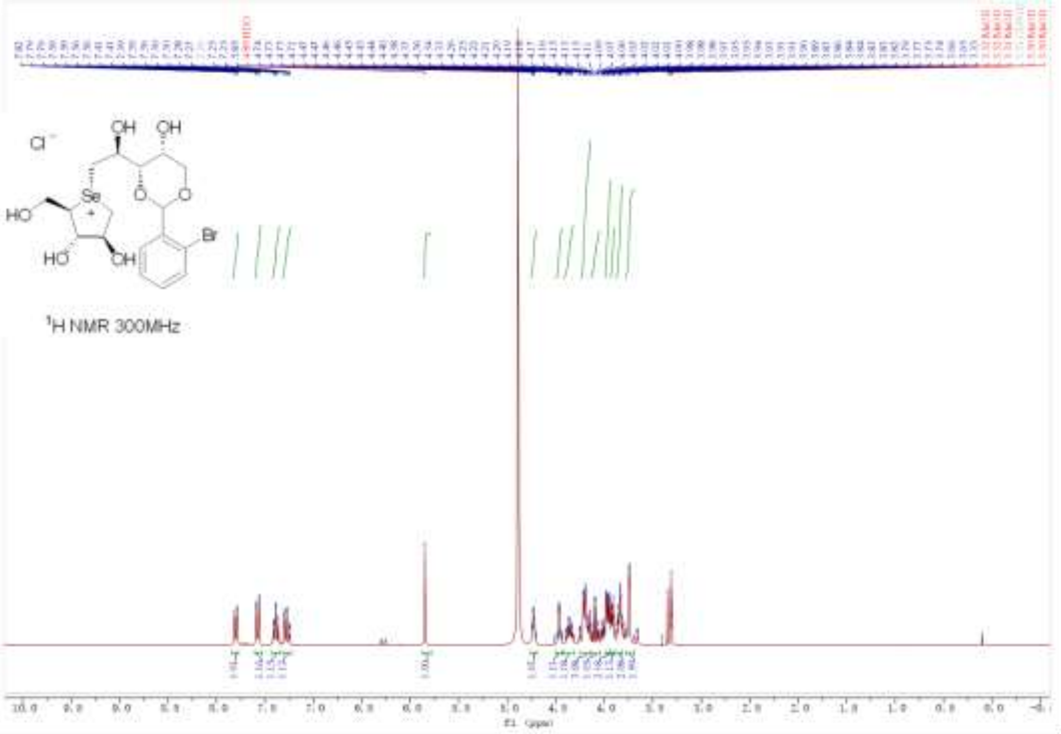

^{13}C-NMR spectrum of **20k**

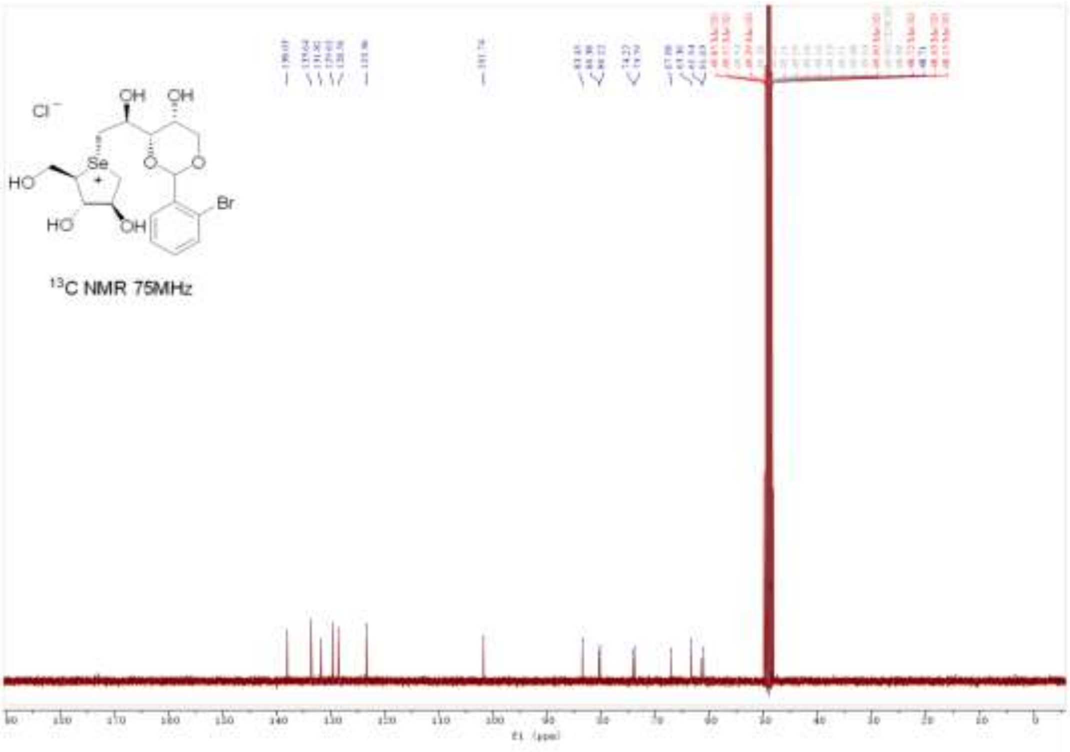

^{1}H-NMR spectrum of **20l**

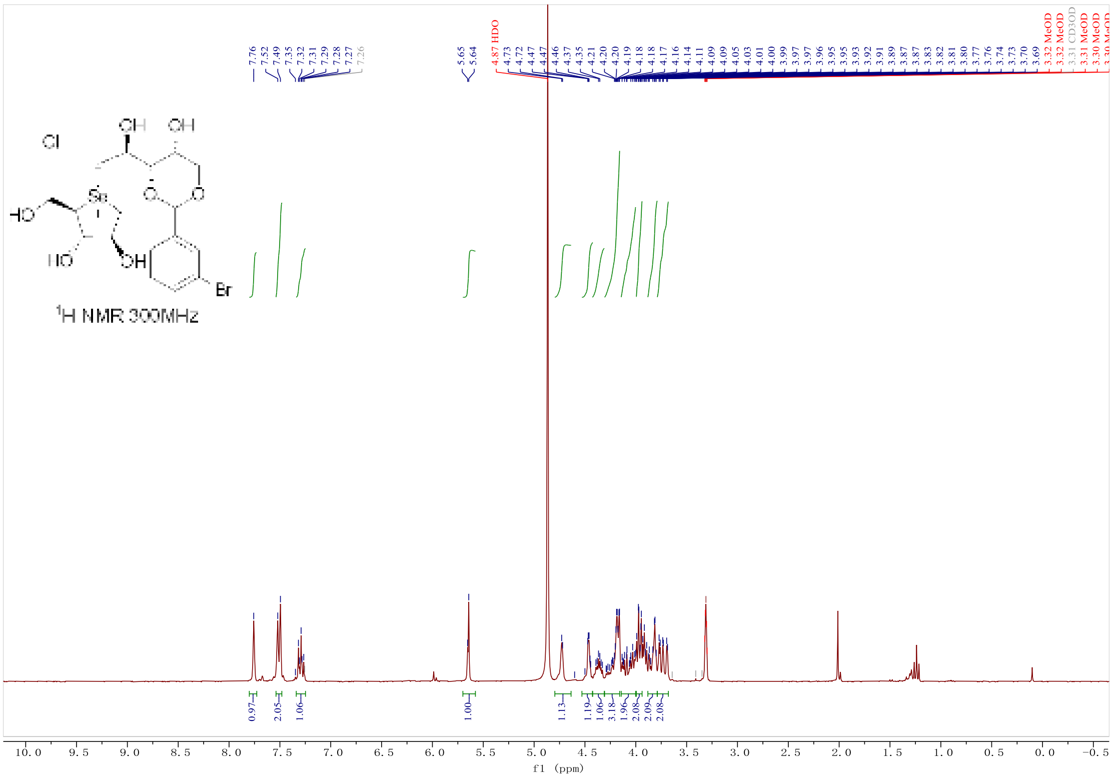

^{13}C-NMR spectrum of **20l**

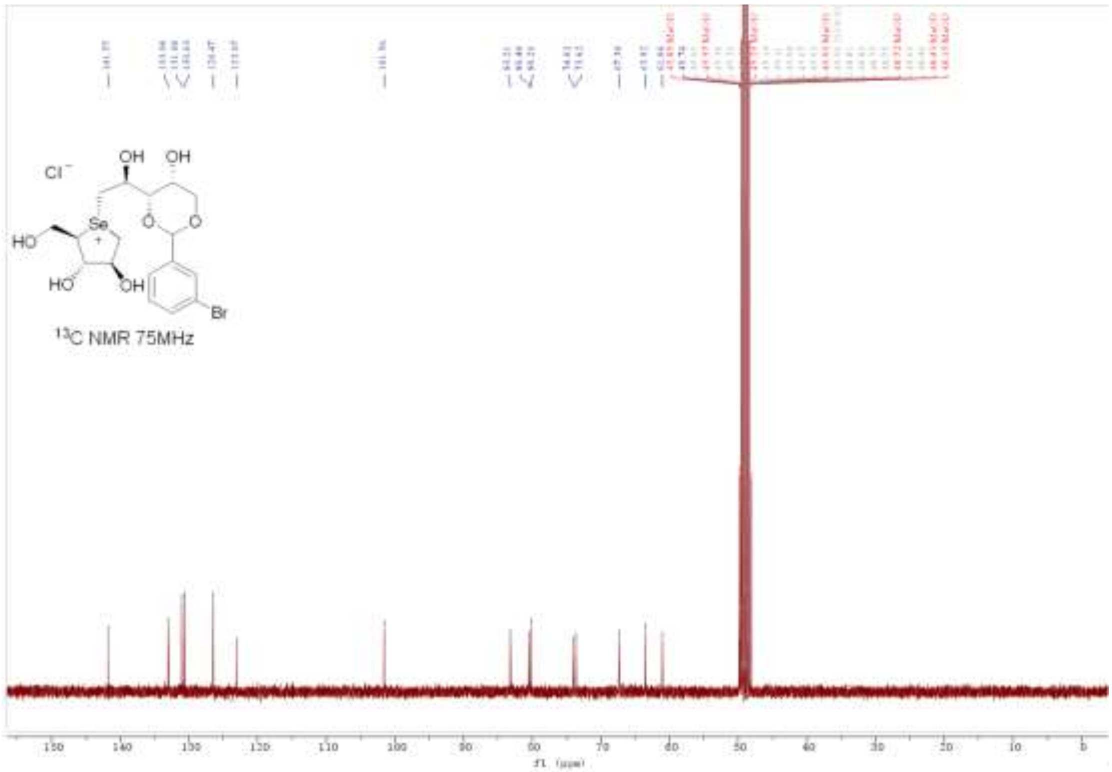

^{1}H-NMR spectrum of **20m**

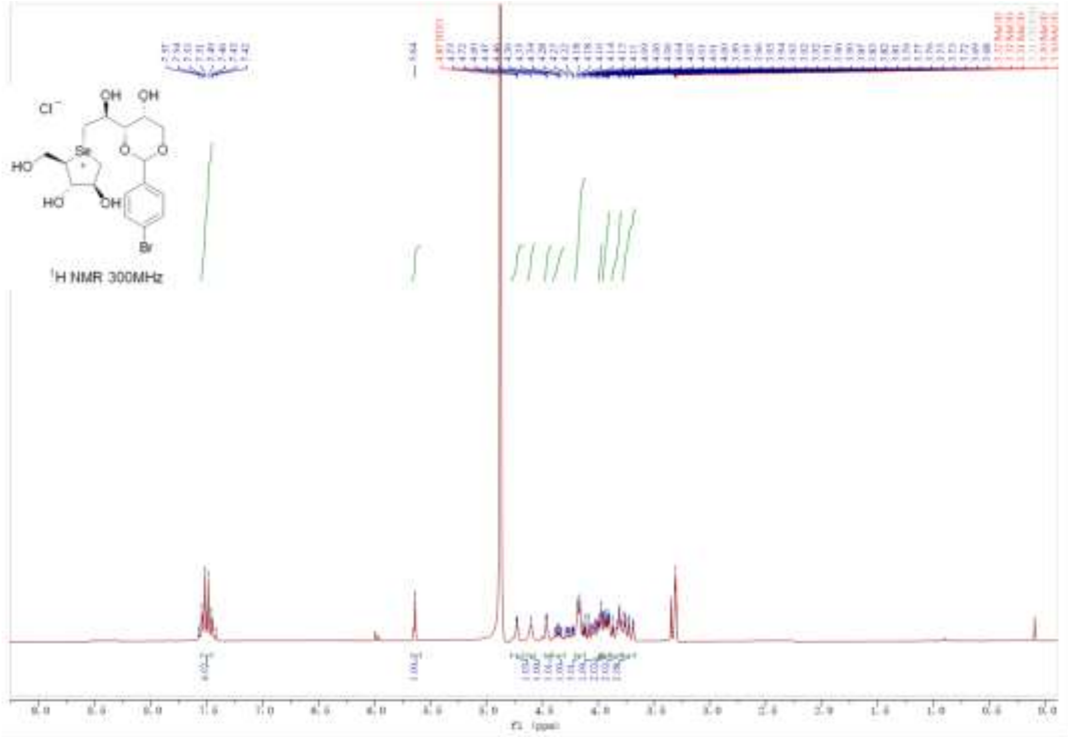

^{13}C-NMR spectrum of **20m**

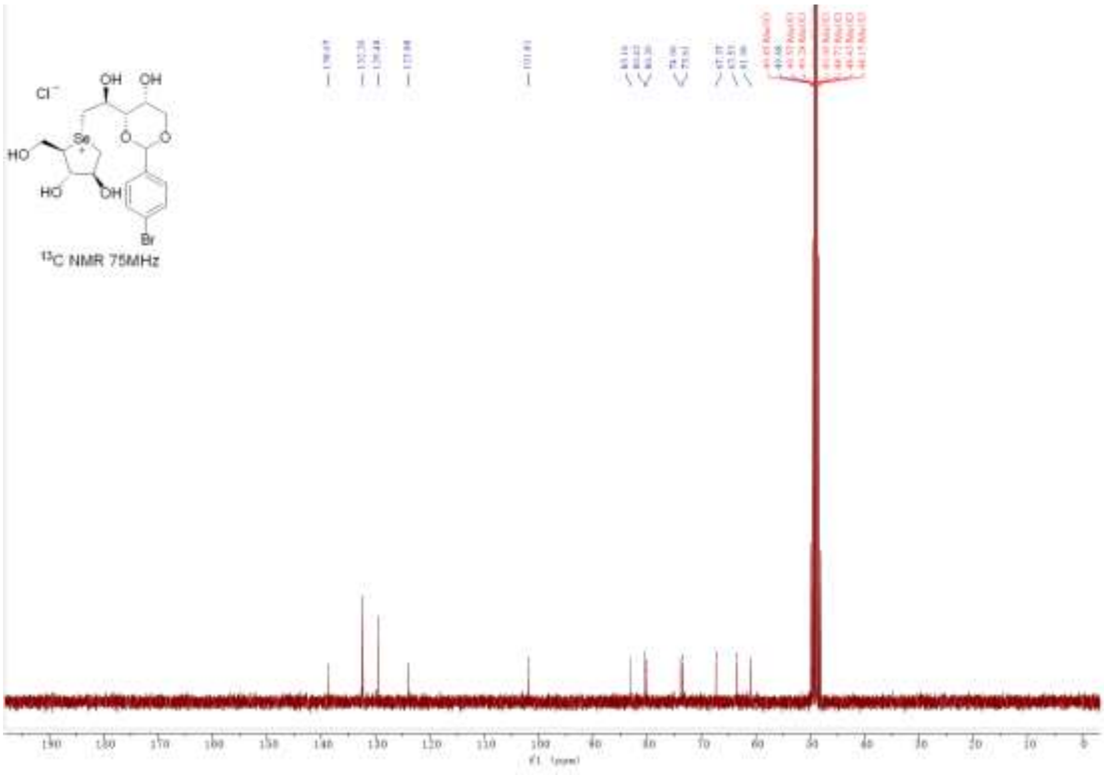

^{1}H-NMR spectrum of **20n**

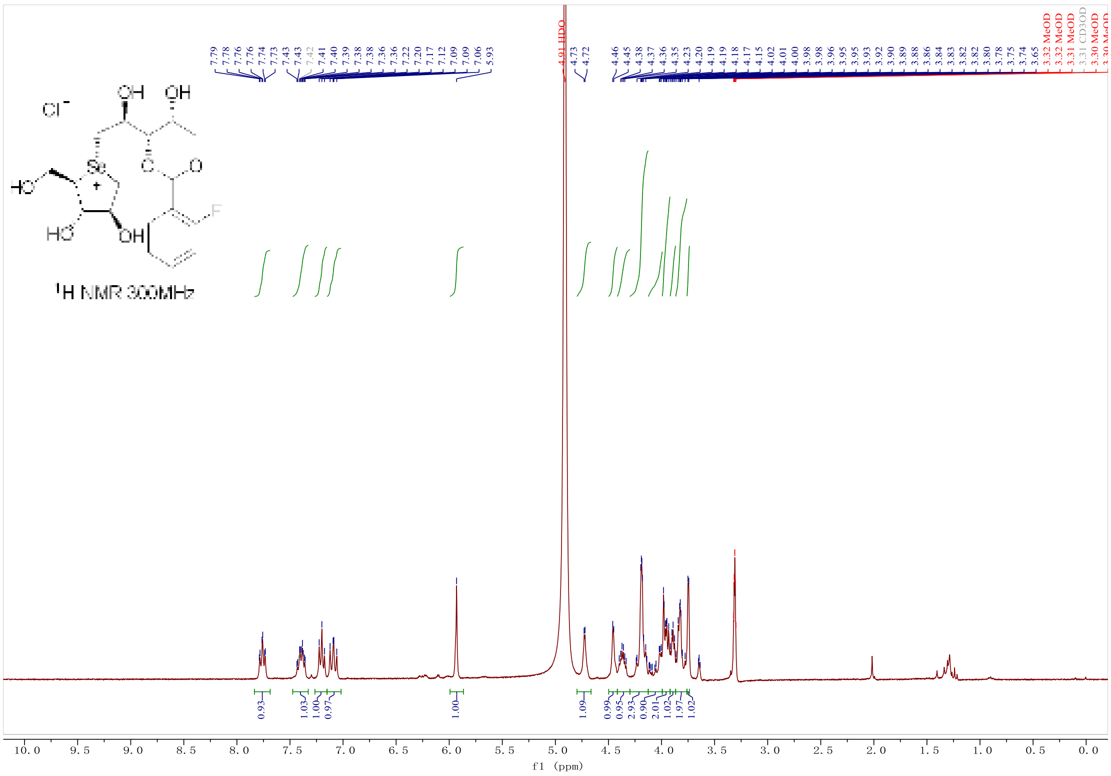

^{13}C-NMR spectrum of **20n**

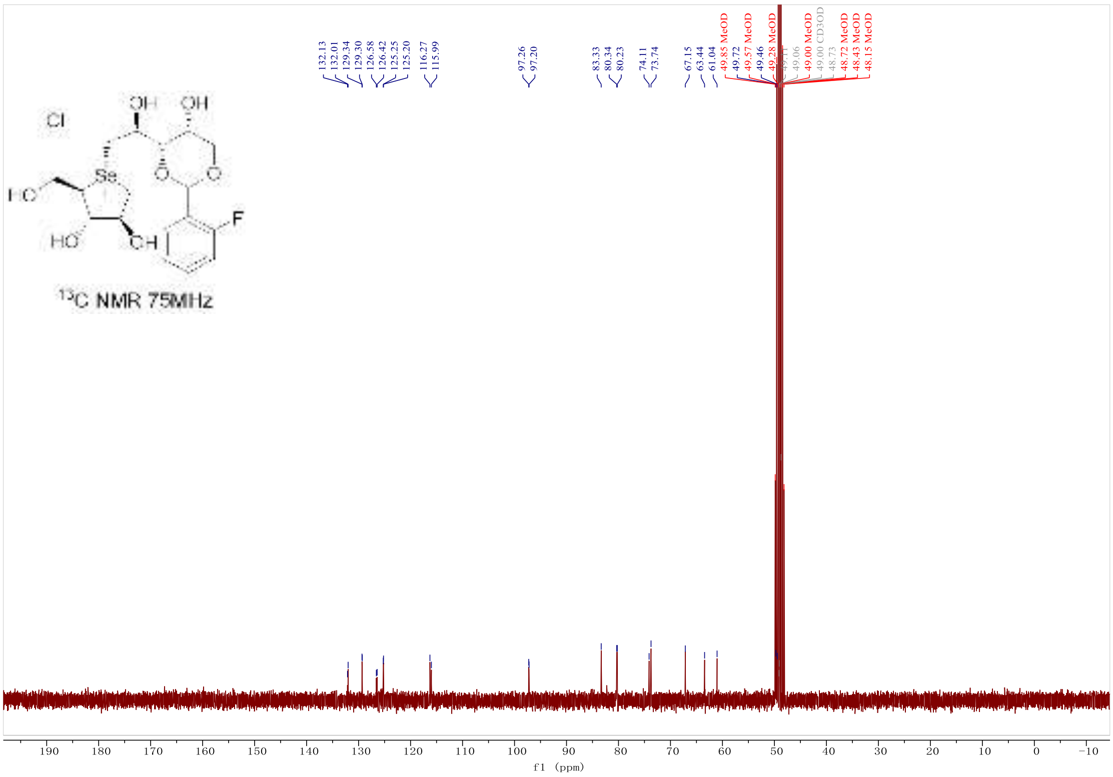

^{1}H-NMR spectrum of **20o**

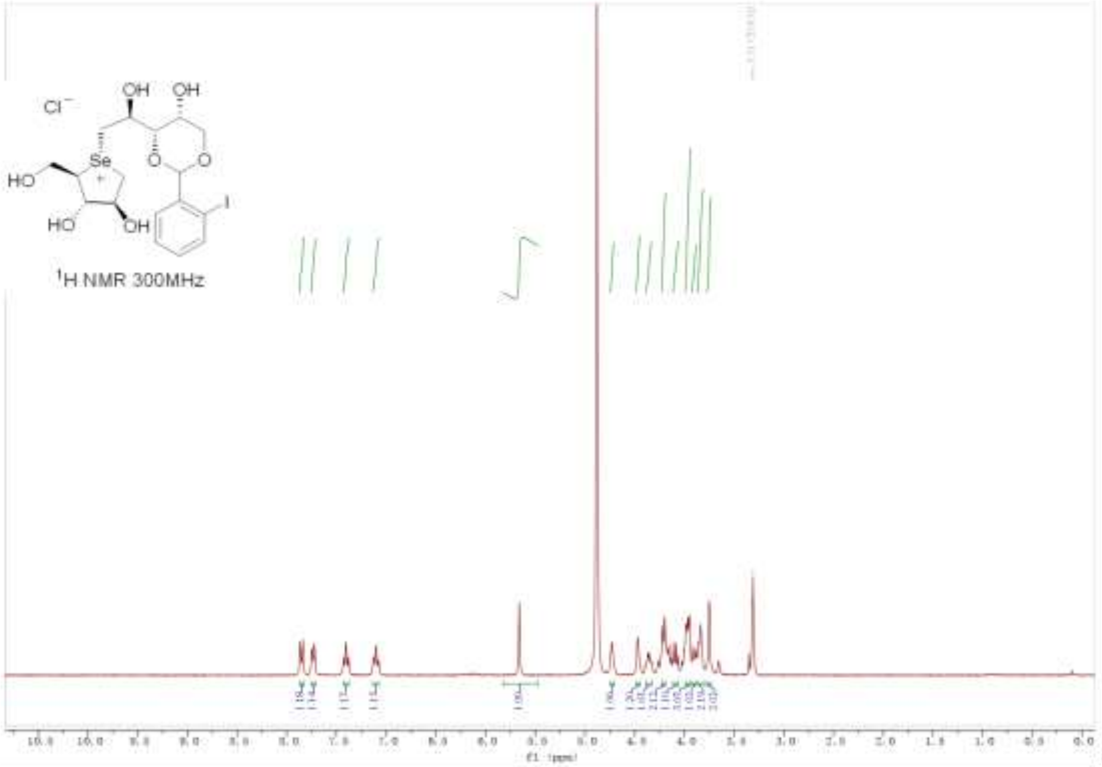

^{13}C-NMR spectrum of **20o**

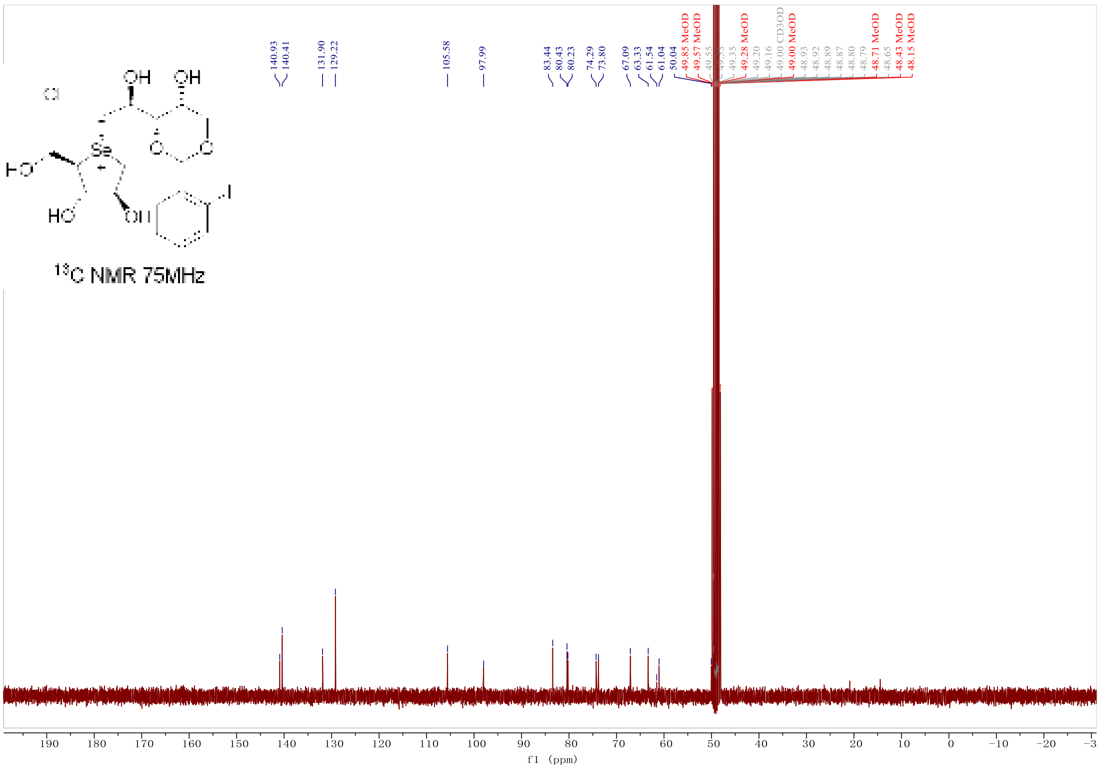

^{1}H-NMR spectrum of **20p**

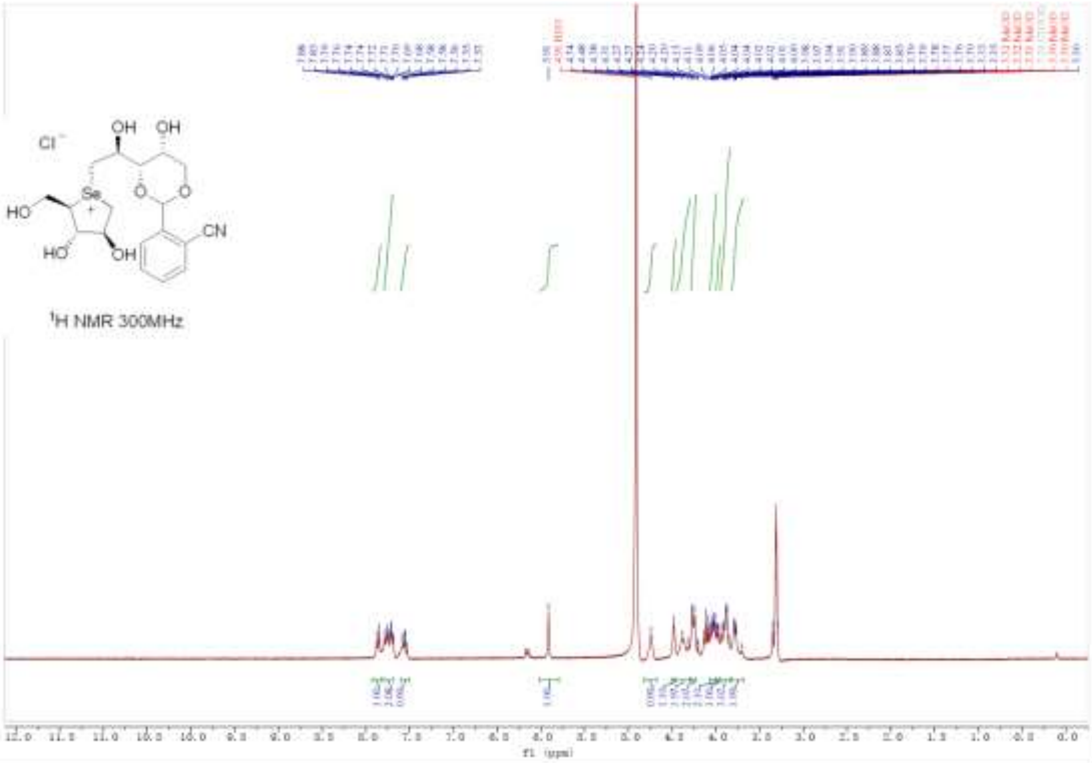

^{13}C-NMR spectrum of **20p**

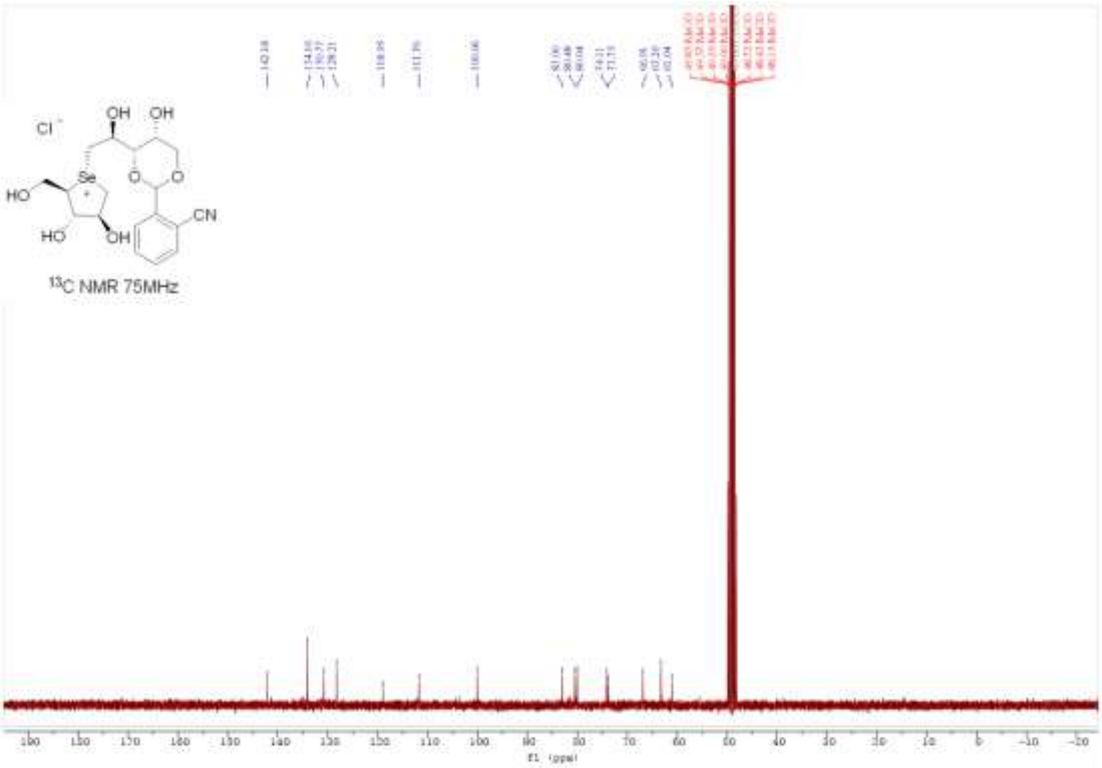

^{1}H-NMR spectrum of **20q**

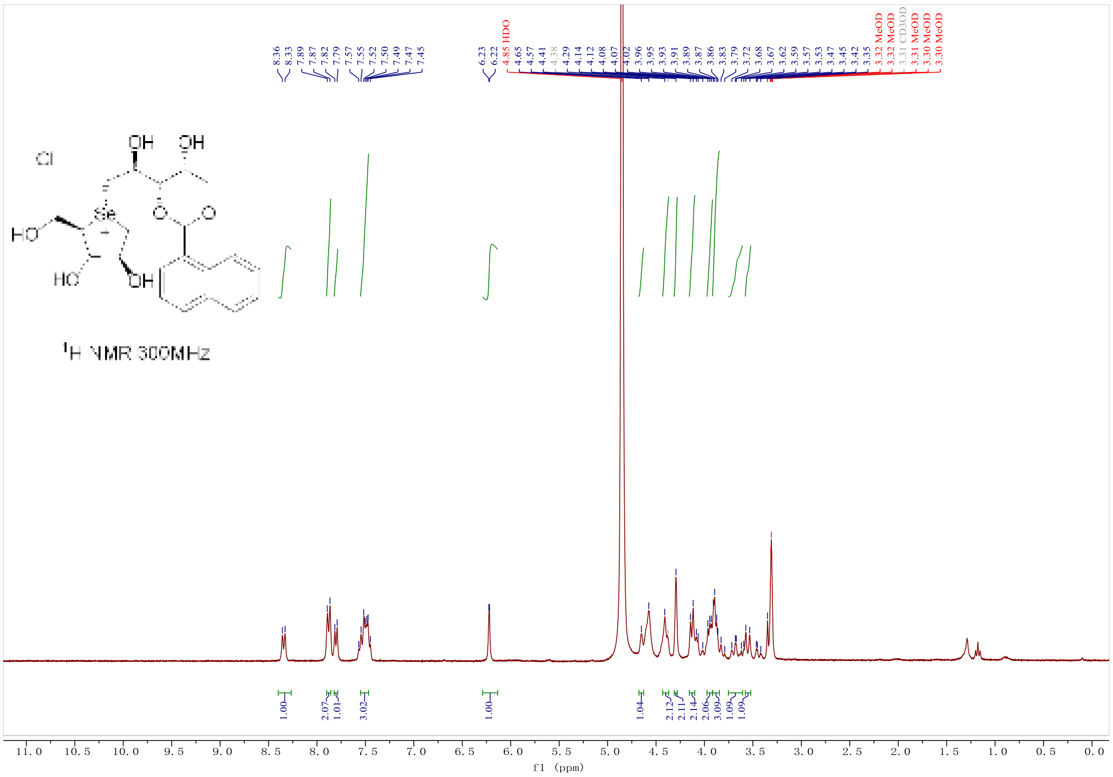

^{13}C-NMR spectrum of **20q**

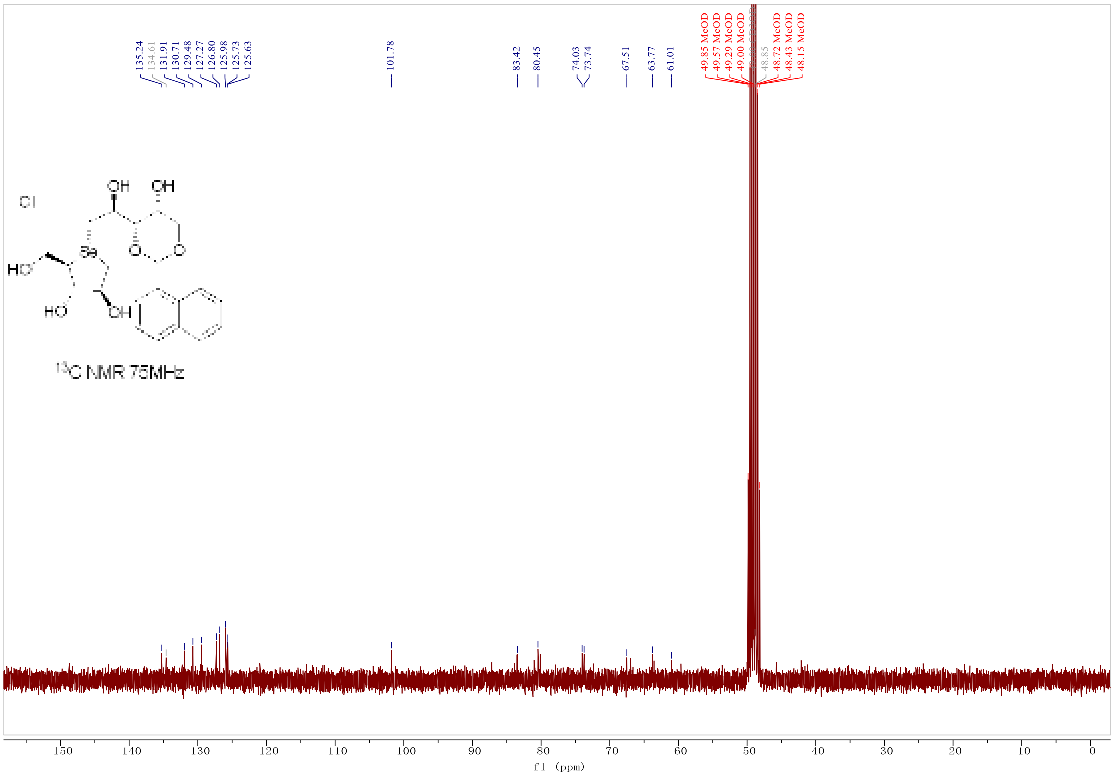

^{1}H-NMR spectrum of **20r**

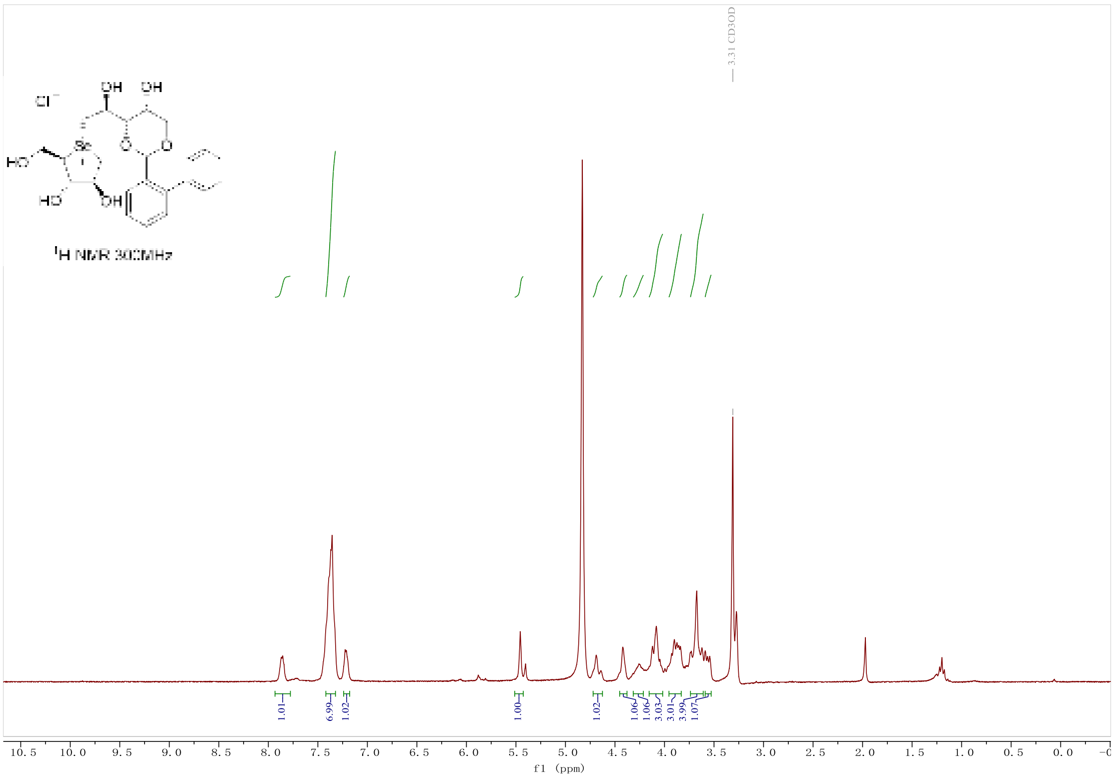

^{13}C-NMR spectrum of **20r**

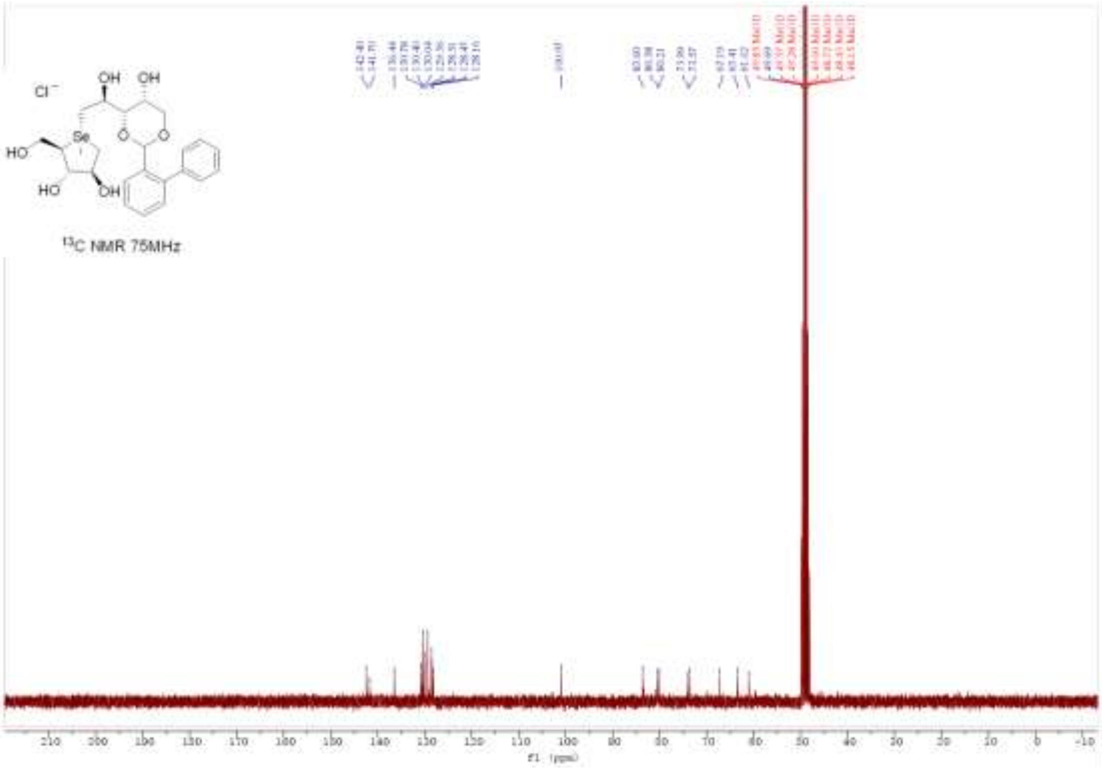

Curves of selected sulfonium and selenonium salts in enzyme inhibition assay

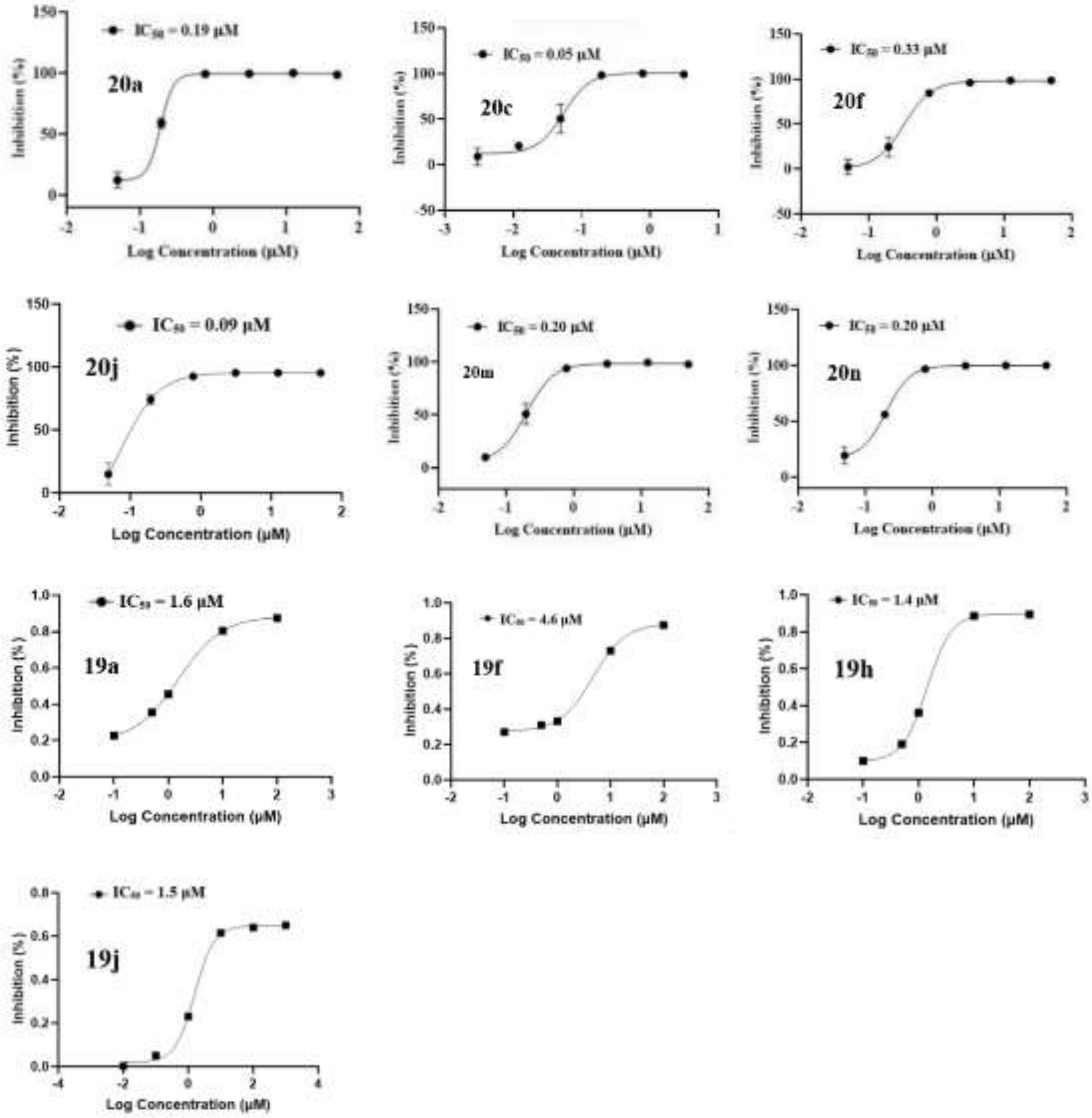

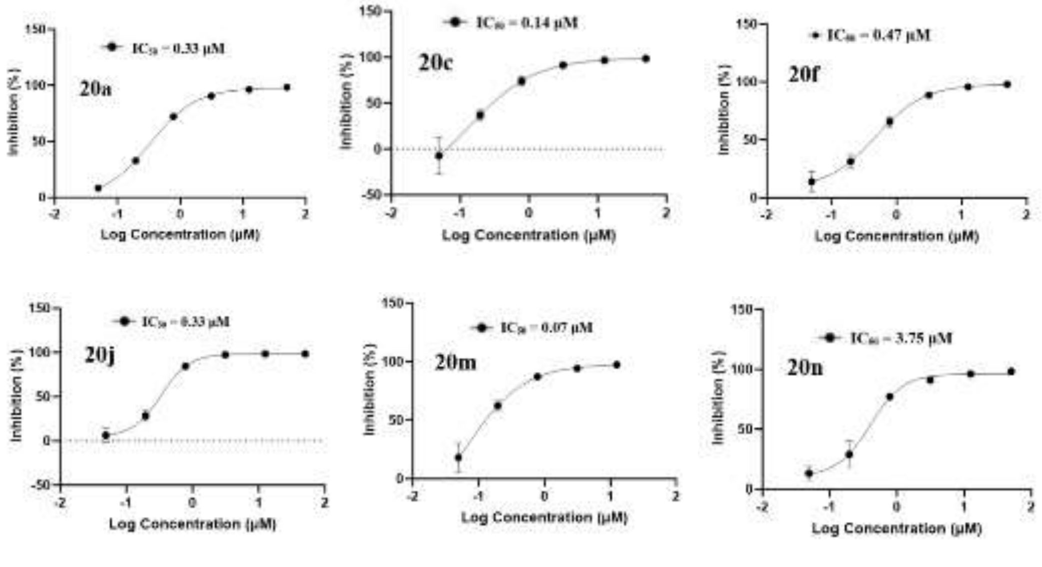

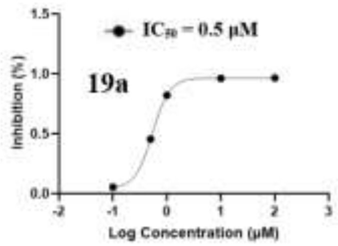
IC50 = 0.5 μM
19a
Inhibition (%)
Log Concentration (μM)

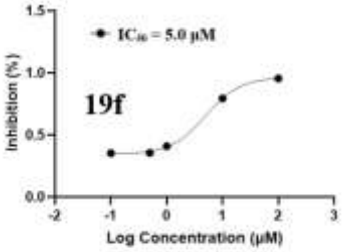
IC50 = 5.0 μM
19f
Inhibition (%)
Log Concentration (μM)

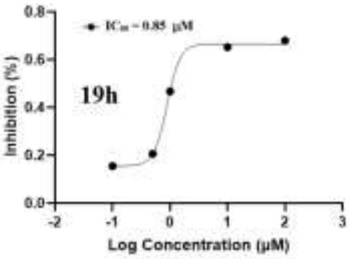
IC50 = 0.85 μM
19h
Inhibition (%)
Log Concentration (μM)

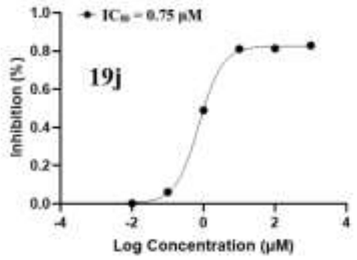
IC50 = 0.75 μM
19j
Inhibition (%)
Log Concentration (μM)

IC_{50} Values (μM) of the 20a and 20c designed in the present study against different normal cell lines.

Compounds	*HELF*	*LO2*	*Primary Hepatocyte*
**20a**	>500	>500	>500
**20c**	>500	>500	>500

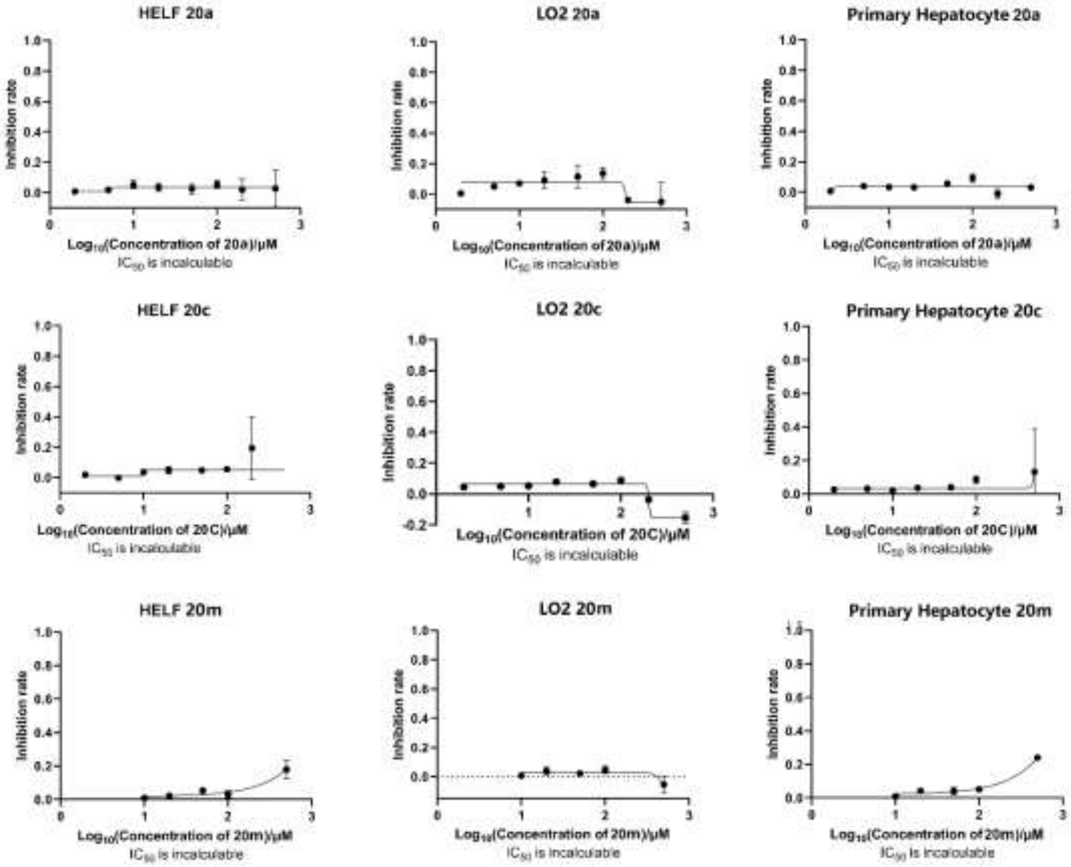

Supplement: Supplementary file 1 [file molecules-30-02856-s001.zip › molecules-3661752-supplementary.pdf]
